# An Efficient Synthesis of 3,5-Bis-Aminated Pyrazolo[1,5-*a*]Pyrimidines: Microwave-Assisted Copper Catalyzed C-3 Amination of 5-Amino-3-Bromo-Substituted Precursors

**DOI:** 10.3390/molecules30030458

**Published:** 2025-01-21

**Authors:** Terungwa H. Iorkula, Bryce A. Tolman, Latifat O. Ganiyu, Matt A. Peterson

**Affiliations:** Department of Chemistry and Biochemistry, Brigham Young University, Provo, UT 84602, USA

**Keywords:** pyrazolo[1,5-*a*]pyrimidines, copper catalyzed amination, microwave-assisted reactions, ullmann-type arylation

## Abstract

An efficient method has been developed for the rapid production of diverse arrays of 3,5-bis-aminated pyrazolo[1,5-*a*]pyrimidines. The method utilizes CuI (5 mol%) and carbazole-based ligand **L-1** (N-(9H-carbazol-9-yl)-1H-pyrrole-2-carboxamide) (10 mol%) for efficient Ullmann-type coupling of various amines to 5-amino-3-bromopyrazolo[1,5-*a*]pyrimidine precursors after heating in diethylene glycol (DEG) for only 1 h at 80 °C (microwave heating). 3,5-Bis-aminated products were obtained in good to excellent yields (60–93%, 83% average for 29 examples). 1° and 2° alkylamines, as well as a variety of aryl- or heteroarylamines coupled efficiently, and 1° and 2° alkyl (or aryl) amines at C-5 were well tolerated. The optimized conditions worked well on both the 50 mg and 1.0 g scales and gave products in only two steps from commercially available 3-bromo-5-chloropyrazolo[1,5-*a*]pyrimidine. Advantages provided by this method include short reaction time, excellent yields, broad substrate scope, and avoidance of toxic reagents commonly utilized for reductive aminations of C-3 NH_2_ substituted precursors.

## 1. Introduction

**Figure 1:**
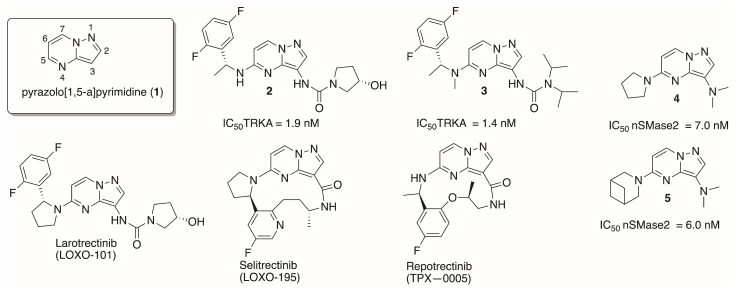
Clinical and preclinical biologically active pyrazolo[1,5-*a*]pyrimidines.

**Figure 2:**
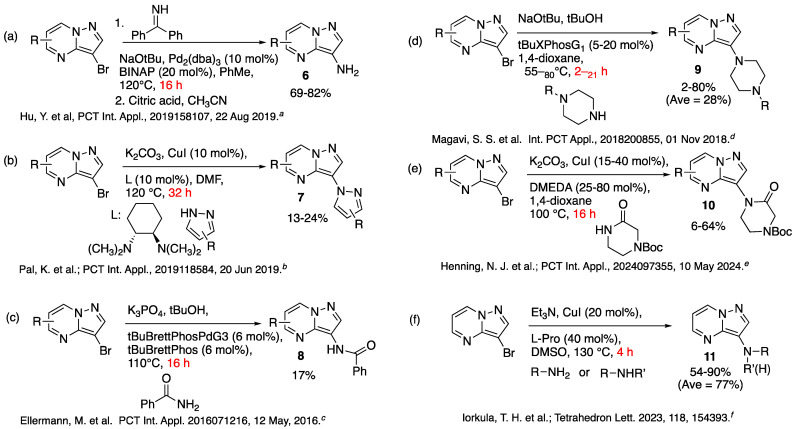
Buchwald-Hartwig or Ullmann-type coupling methods for C-3 amination. *^a^* [21], *^b^* [22], *^c^* [23], *^d^* [24], *^e^* [25], *^f^* [26].

**Figure 3:**
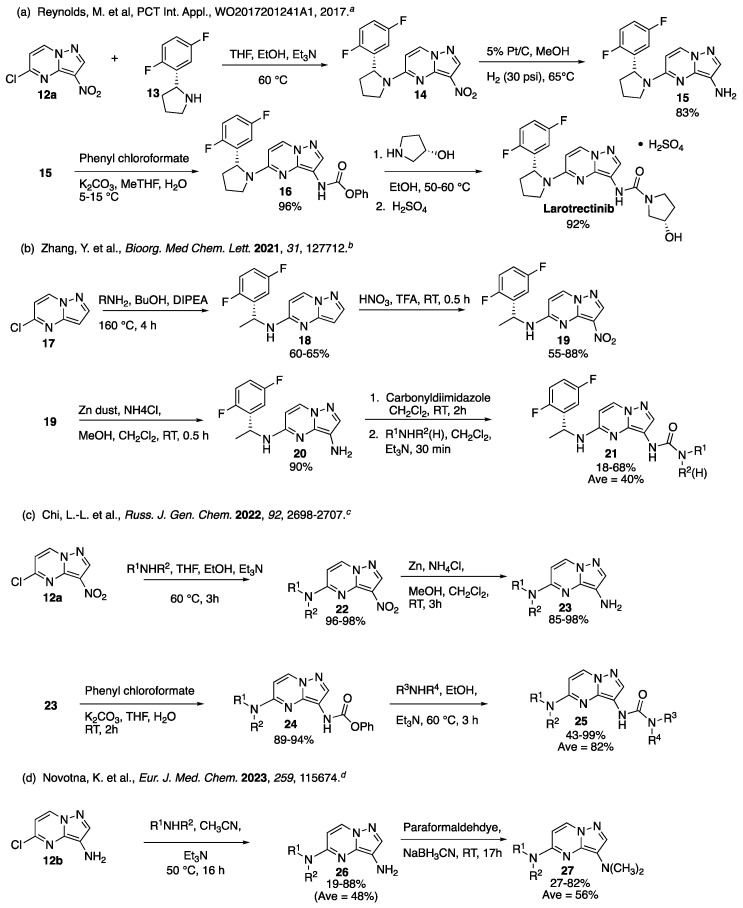
Synthesis of 3,5-bis-aminated pyrazolo[1,5-*a*]pyrimidines. *^a^* [27], *^b^* [13], *^c^* [29], *^d^* [20].

**Scheme 1:**
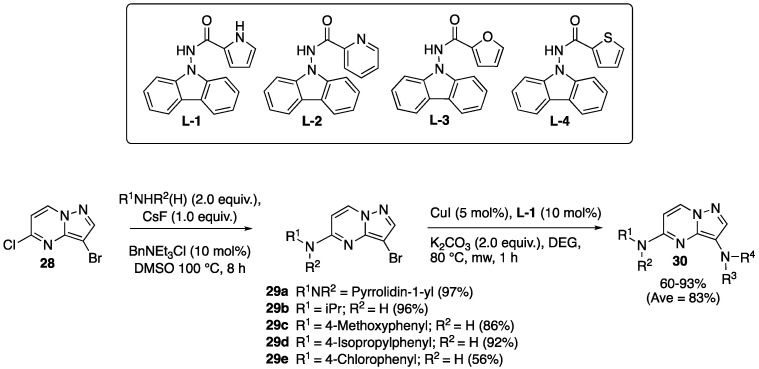
Two-step preparation of 3,5-bis-aminated pyrazolo[1,5-*a*]pyrimidines **30**.

The pyrazolo[1,5-*a*]pyrimidine scaffold (1) is a versatile template that has been extensively investigated for a number of important medicinal and biologically relevant applications. For example, pyrazolo[1,5-*a*]pyrimidine derivatives have been investigated as potential therapeutics for diabetes [1], cancer [2], neurodegenerative diseases [3], and various infectious diseases, including viral [4], protozoal [5], fungal [6], trypanosomal [7], and bacterial infections [8]. In addition, pyrazolo[1,5-*a*]pyrimidines have been used in dyes [9], fluorophores [10], chemosensors [11], tumor imaging [12], and as inhibitors of protein or lipid kinases [13]. Anti-inflammatory [14], anti-anxiolytic [15], and sedative-hypnotic [16] properties have also been reported. The clinical anticancer drugs Larotrectinib [17] and Reprotrectinib [18], along with several other structurally related clinical [19] or preclinical [13] candidates, as well as other promising disease-related enzyme inhibitors [20], have stimulated increased interest in the pyrazolo[1,5-*a*]pyrimidine scaffold (Figure 1). Of particular interest in recent years has been the synthesis and biological evaluation of C-3 aminated analogs, as underscored by the number of recently reported methodologies for converting C-3 brominated precursors to C-3 aminated derivatives (Figure 2) [21,22,23,24,25,26]. These methods involve transition-metal-catalyzed Buchwald-Hartwig or Ullmann-type coupling reactions, whereas the originally reported method for preparing Larotrectinib involved the reduction of a C-3 nitro group followed by a two-step formation of the urea (Figure 3) [27,28]. The most commonly reported methods for preparing 3,5-bis-aminated

pyrazolo[1,5-*a*]pyrimidine derivatives have either followed the route originally reported for Larotrectinib or related approaches varying essentially only in the order of C-3 nitration, reduction, and/or C-5 Nucleophilic Aromatic Substitution (SNAr) (Figure 3) [13,20,27,28,29]. Although these methods have provided compound libraries for SAR (Structure Activity Relationship) screening, they suffer from several drawbacks, including low yields, multiple steps, and/or limited synthetic versatility for functionalizing the C-3 NH_2_ moiety. Indeed, the C-3 NH_2_ of C-5 aminated derivatives has so far only been converted to ureas or thioureas via two-step carbamoylation/amination or to the N(CH_3_)_2_ group via reductive amination utilizing toxic reagents paraformaldehyde and NaBH_3_CN (Figure 3). Ideally, a synthetic method that avoids these limitations would provide efficient access to a broader array of analogs in short reaction times, high yields, and fewer steps, and it would avoid the use of toxic reagents. Given the considerable biological potential of such derivatives, methods meeting these criteria are highly desirable. Here, we report a new method that achieves these objectives, giving 3,5-bis-aminated products in 83% yield (average from 29 examples), utilizing 5 mol% of CuI catalyst and 10 mol% of carbazole-based ligand (**L**-**1**) after heating for only 1 h at 80 °C for C-3 amination. The reaction proceeded in only two steps from commercially available 3-bromo-5-chloropyrazolo[1,5-*a*]pyrimidine (Scheme 1) and provided a broad array of 3,5-bis-aminated products in good to excellent yields (Table 2). Substrates with both 1° alkyl (or aryl) and 2° alkylamines at C-5 were coupled effectively, and efficient C-3 amination was achieved using a variety of 1° or 2° alkylamine as well as aryl- and heteroarylamine C-3 coupling partners. With this method, rapid and efficient access to diverse libraries of 3,5-bis-aminated derivatives consisting of previously unavailable analogs is now readily attainable.

## 2. Results

### 2.1. Optimization

#### 2.1.1. Temperature, Solvent, and Base

From the recent report by Huang and co-workers wherein **L-1** was shown to promote the efficient N-arylation of cyclopropylamine with arylbromides and a CuI catalyst at room temperature [30], we became interested in the possibility that this novel ligand might prove useful for preparing 3,5-bis-aminated pyrazolo[1,5-*a*]pyrimidines that were inaccessible via previously published methods (see Figure 2 and Figure 3). Despite the fact that our previous L-proline/CuI method worked well for preparing C-3 mono-aminated derivatives from substrates that lacked a C-5 amino substituent (see Figure 2f) [26], the yields for converting C-5 amino substituted precursors (e.g., **29a** or **29b**) to 3,5-bis-aminated products **30** were consistently (and unacceptably) low (<35%), with much of the mass balance being attributed to the debrominated byproduct (e.g., **31**). In order to determine if the **L-1**/CuI catalyst system could overcome this limitation, we applied Huang’s conditions, utilizing **29a** and morpholine as our model system (Table 1, entry 1). Disappointingly, **L-1**/CuI failed to produce any detectable amount of **30e**, and only unreacted starting material **29a** (with trace amounts of **31**) were detected, even after 7 days at RT (Table 1, entry 1). Heating the reaction at incrementally higher temperatures (50–70 °C, using an oil bath) for 1–2 days gave a slightly better conversion, but substantial quantities of unreacted starting material (70–92%) remained (Table 1, entries 2–3). Heating at 80 °C was required for the complete conversion of **29a**; however, 18% of the debrominated byproduct (**31**) was also obtained (Table 1, entry 4). Next, microwave heating was examined. Encouragingly, our first attempt with microwave heating (Table 1, entry 5) gave 61.4% **30e**, with 1.4% of the debrominated byproduct **31** after heating for only 30 min at 70 °C. Increasing the reaction time (from 30 min to 3h) at 70 °C gave increased conversions, but debromination also increased from 1.8–2.7% (Table 1, entries 5–9). Microwave heating at 80 °C for 1 h gave approximately equivalent results to the reaction run at 70 °C for 3 h (Table 1, entries 9 and 11), while a reaction time of 30 min at 80 °C left 4.4% unreacted **29a** (Table 1, entries 10–11). A longer reaction time at 80 °C (1.5 h) gave product with essentially identical conversion to that obtained after only 1 h (Table 1, entries 11–12). At this point, it seemed reasonable to perform a solvent survey in order to confirm that, in keeping with Huang’s observations regarding arylation of cyclopropylamine with **L-1**/CuI, diethylene glycol (DEG) was the most suitable solvent [30]. Accordingly, six common polar solvents were evaluated (Table 1, entries 13–18). Of these solvents, ethylene glycol and 1,2-propanediol gave comparable results (90–94% conversion), but suffered from higher percentages of debromination (5.7–9.9%, Table 1, entries 13 and 18). NMP gave a lower yield (78%) with 19% debromination (Table 1, entry 17). The reaction in 1,4-dioxane failed to give any detectable C-3 aminated product (entry 16), while n-butanol and DMSO gave only 3.6–10.2% conversion, respectively (Table 1, entries 14–15). Next, a survey of four bases commonly used for transition-metal-catalyzed coupling was conducted (Table 1, entries 19–22). The inorganic base K_3_PO_4_ gave inferior conversion (94% compared to 97%), while tBuOK and TMSONa gave product **30e** in yields comparable to those obtained with K_2_CO_3_ (Table 1, entries 11 and 19–20). DBU afforded the least effective conversion (48.8%; Table 1, entry 22). Based on these data and the relative cost efficiency of K_2_CO_3_ compared to TMSONa (which is approx. 55-fold more expensive than K_2_CO_3_ on a 100 g basis), we opted to use the conditions in entry 11 (80 °C, 1 h, DEG, K_2_CO_3_) for further optimization and substrate scope determination (*vide infra*).

#### 2.1.2. Ligand and C-3 Halogenated Precursor

**Scheme 2:**
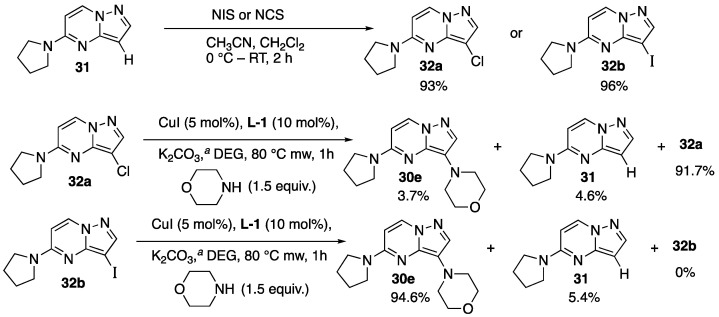
C-3 amination with 3-chloro or 3-iodo precursors. *^a^* (2.0 equiv).

A brief survey of structurally related ligands (**L-2**, **L-3**, and **L-4**) further confirmed the suitability of the **L-1**/CuI catalyst system (Table 1, entries 23–25). Notably, **L-2** (which was recently shown to promote the efficient CuI-catalyzed arylation of phenols) [31] gave a nearly 2:1 ratio of debrominated **31**:**30e**, while **L-3** and **L-4** gave substantially less efficient conversions (12–17%) under our optimized microwave conditions. In order to confirm the suitability of the C-3 brominated precursors, we also screened 3-chloro and 3-iodo derivatives (**32a–b**) using our optimized conditions (Scheme 2). The reaction for the 3-chloro derivative was markedly inferior (<4% conversion), while the 3-iodo derivative gave excellent yields but suffered from greater dehalogenated byproduct (**31**) compared to the 3-bromo derivative **29a** (5.4 and 2.9%, respectively).

## 3. Discussion

Using the optimized conditions established in our preliminary experiments (Table 1 and Scheme 2), we applied this methodology to the substrates listed in Table 2. We were pleased to note the broad substrate scope and functional group compatibility exemplified by a variety of 1° alkyl and 2° alkylamines and aryl- or heteroarylamines, with key functionalities such as azides or BOC groups being well tolerated (Table 2). However, amides and ureas did not couple efficiently with our method (and the coupling products from those substrates formed in only trace amounts under our optimized conditions). Notwithstanding, the substrate scope presented in Table 2 is the broadest and most comprehensive scope of 3,5-bis-aminated products yet to be reported for the pyrazolo[1,5-*a*]pyrimidine scaffold coupled with alkyl- or arylamines [21,22,23,24,25,26,27,28]. Previous methods have been restricted to only C-3 mono-aminated derivates or an extremely limited number of 3,5-bis-aminated products, as illustrated in Figure 3. (The reactions highlighted in Figure 3 are summarized from an exhaustive Scifinder Scholar search conducted in November 2024). These methods were limited to either C-3 ureas, urethanes, or the (CH_3_)_2_N group, each of which was obtained via a somewhat circuitous approach requiring C-3 nitration, followed by reduction, with a final step involving either acylation or reductive amination. In our case, C-5 alkylamine- or arylamine-substituted precursors were obtained directly in one step from commercially available 3-bromo-5-chloropyrazolo[1,5-*a*]pyrimidine (Scheme 1). C-3 amination of both C-5 alkylamine and C-5 arylamine substrates worked equally well, and **29a–b** and **29c–e** gave 3,5-bis-aminated products in 82.2% and 83.1% (average yields, respectively). Thus, a broad range of 3,5-bis-aminated products, consisting of both 3,5-bis arylamine, 3,5-bis alkylamine, or 3,5-bis alkyl/aryl amine combinations, could be efficiently obtained in only two steps utilizing our method. The functional group tolerance is noteworthy, given the significant potential that derivatives such as **30d**, **30g**, **30n**, and **30z–c’** have for further elaboration into more complex libraries of compounds. Facile TFA-promoted cleavage of the BOC protecting groups or reduction of the azide would provide the corresponding amine products for further functionalization, and the aryl chloride moieties of **30z–c’** could also be used for Suzuki-Miyaura coupling. An additional advantage of azide **30n** is its potential use in conventional “click” coupling chemistry for conjugating biologically relevant payloads for biological applications.

3,5-Bis-aminated derivatives with other C-5 amino groups could also be envisioned since model substrates **29a–e** establish that a variety of alkyl or aryl amines at this position are well tolerated. Advantages of this method include rapid reaction time (1 h for C-3 amination), high yield (83% average for all 29 substrates examined), avoidance of toxic reagents such as paraformaldehyde and NaBH_3_CN, and a short reaction sequence (only two steps from commercially available 3-bromo-5-chloropyrazolo[1,5-*a*]pyrimidine). In addition, our method avoids expensive air- and moisture-sensitive palladium catalysts and provides much greater synthetic versatility than any previously reported method (Figure 2 and Figure 3). Perhaps the most striking drawback of the previous methods for 3,5-bis-aminated pyrazolo[1,5-*a*]pyrimidines is their limited scope (limited to C-3 ureas or dimethylamine). Our method opens up the possibility of preparing much broader, diversity-rich compound libraries for screening novel and/or enhanced biological, medicinal, or fluorometric properties.

**Table 2:** Substrate scope for C-3 amination. ^a,b,c^

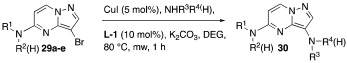
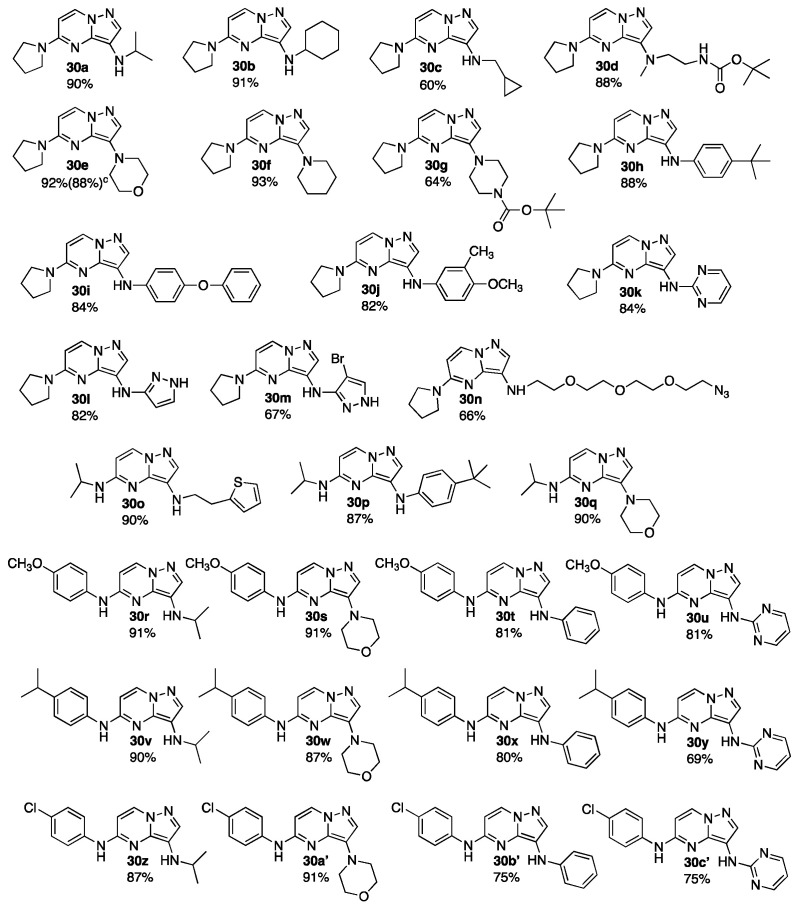

^a^ Reaction conditions: **29a–e** (50 mg, 0.15–0.20 mmol), amine (1.5 equiv.), K_2_CO_3_ (2.0 equiv.), CuI (5 mol%), **L-1** (10 mol%), diethyleneglycol (DEG) (1.0 mL); ^b^ Isolated yields; ^c^ Reaction performed on 1.0 g (3.80 mmol) scale.

## 4. Materials and Methods

### 4.1. General Experimental

All reactions were performed in either appropriately sized pressure vessels (conventional heating) or in 10 mL microreactor vessels (microwave heating) without previous drying, unless otherwise noted. Conventional heating was performed in an oil bath, and microwave heating was achieved using a CEM Microwave reactor. Reagent-grade solvents and all other reagents were used directly as supplied by commercial suppliers without additional drying or purification. ^1^H NMR and ^13^C NMR spectra were determined in CDCl_3_ or DMSO-*d*_6_ using internal references: *δ* 7.27 or 2.50 (^1^H) and *δ* 77.23 or 39.52 (^13^C), respectively. Abbreviations such as “d”, “t”, or “bs” refer to “apparent doublet”, “apparent triplet”, or “apparent broad singlet”, where such apparent multiplicities represent experimentally observed splitting patterns that result from line-broadening caused by conformational isomerism and/or concentration. High-resolution mass spectra were obtained using fast atom bombardment electrospray (ES) ionization techniques and had errors of less than ±5 ppm.

### 4.2. Ligand Synthesis

The method for preparing ligands **L-1**, **L-2**, **L-3**, and **L-4** is a modification of the method reported by Huang and co-workers [30] (Supplementary Materials, Scheme S1). In our hands, their method for preparing **L**-**1**, which employed SOCl_2_ for generating an acyl chloride from pyrrole 2-carboxylic acid, consistently gave yields <35%, even after rigorous drying of glassware, solvents, and reagents. We employed Ph_3_P/NIS to generate a reactive acyl iodide intermediate [32,33] and obtained the desired amidation of N-amino-9H-carbazole [34] in higher yields (38–67%; ave = 49%). (Scheme S1).

### 4.3. 5-Amino-3-Bromopyrazolo[1,5-a]Pyrimidines 29

General Procedure: A solution of **28** (200 mg, 0.86 mmol), pyrrolidine (123 mg, 1.73 mmol, 2.0 equiv), CsF (131 mg, 0.86 mmol, 1.0 equiv), and BnNEt_3_Cl (20 mg, 0.088 mmol, 10 mol%), in DMSO (6 mL) was stirred in a sealed pressure vessel (15 mL) and heated at 100 °C for 8 h [32,35]. After cooling to ambient temperature, H_2_O (60 mL) was added. The mixture was diluted with ethyl acetate and the organic layer was separated. The aqueous layer was partitioned with EtOAc (2 × 60 mL) and the combined organic layers were washed with saturated sodium bicarbonate solution (2 × 60 mL) and water (2 × 60 mL). The organic layer was dried over anhydrous magnesium sulfate, filtered, and evaporated under reduced pressure. The crude product was purified using flash chromatography (EtOAc/Hexanes) to give pure **29a** (222 mg, 0.83 mmol, 97%).

***3-Bromo-5-(pyrrolidin-1-yl)pyrazolo[1,5-a]pyrimidine (29a)***

3-Bromo-5-chloropyrazolo[1,5-*a*]pyrimidine (200 mg, 0.86 mmol), pyrrolidine (123 mg, 1.73 mmol, 2.0 equiv), CsF (131 mg, 0.86 mmol, 1.0 equiv), BnNEt_3_Cl (20 mg, 0.088 mmol,10 mol%), DMSO (6 mL); **29a** (222 mg, 0.83 mmol, 97%); ^1^H NMR (DMSO-*d*_6_, 500 MHz) δ 8.70 (d, *J* = 7.7 Hz, 1H), 8.01 (s, 1H), 6.53 (d, *J* = 7.7 Hz, 1H), 3.62 (“bs”, 4H), 2.06 (“bs”, 4H); ^13^C NMR (DMSO-*d*_6_, 125 MHz) δ 154.5, 145.5, 144.0, 136.2, 99.2, 76.9, 47.2, 25.7, 24.9; HRMS Calcd. for C_10_H_12_BrN_4_ [M + H]: 267.0245; Found: 267.0237 (∆ = 3.0 ppm).

***3-Bromo-5-(N-(isopropyl)amino)pyrazolo[1,5-a]pyrimidine (29b)***

3-Bromo-5-chloropyrazolo[1,5-*a*]pyrimidine (100 mg, 0.43 mmol), isopropylamine (51 mg, 0.86 mmol, 2.0 equiv), CsF (65 mg, 0.43 mmol, 1.0 equiv), BnNEt_3_Cl (10 mg, 0.044 mmol,10 mol%), DMSO (3 mL); **29b** (105 mg, 0.41 mmol, 96%); ^1^H NMR (DMSO-*d*_6_, 500 MHz) δ 8.42 (d, *J* = 7.5 Hz, 1H), 7.85 (s, 1H), 7.54 (d, *J* = 7.4 Hz, 1H), 6.23 (d, *J* = 7.4 Hz, 1H), 4.15 (“d”, *J* = 5.0 Hz, 1H), 1.19 (d, *J* = 6.5 Hz, 6H); ^13^C NMR (DMSO-*d*_6_, 125 MHz) δ 155.8, 145.6, 143.5, 135.6, 101.6, 77.5, 42.1, 22.5; HRMS Calcd. for C_9_H_12_BrN_4_ [M + H]: 255.0245; Found: 255.0237 (∆ = 3.1 ppm).

***3-Bromo-5-(4-(methoxy)anilin-1-yl)pyrazolo[1,5-a]pyrimidine (29c)***

3-Bromo-5-chloropyrazolo[1,5-*a*]pyrimidine (400 mg, 1.73 mmol), 4-methoxyaniline (426 mg, 3.46 mmol, 2.0 equiv), CsF (263 mg, 1.73 mmol, 1.0 equiv), BnNEt_3_Cl (39 mg, 0.172 mmol,10 mol%), DMSO (12 mL); **29c** (473 mg, 1.49 mmol, 86%); ^1^H NMR (DMSO-*d*_6_, 500 MHz) δ 9.73 (s, 1H), 8.62 (d, *J* = 7.6 Hz, 1H), 7.99 (s, 1H), 7.81 (d, *J* = 8.8 Hz, 2H), 6.96 (d, *J* = 9.0 Hz, 2H), 6.50 (d, *J* = 7.6 Hz, 1H), 3.76 (s, 3H); ^13^C NMR (DMSO-*d*_6_, 125 MHz) δ 155.3, 153.9, 144.9, 143.9, 136.4, 133.5, 121.2, 114.5, 102.1, 78.8, 55.7; HRMS Calcd. for C_13_H_12_BrN_4_O [M + H]: 319.0194; Found: 319.0181 (∆ = 4.1 ppm).

***3-Bromo-5-(4-(isopropyl)anilin-1-yl)pyrazolo[1,5-a]pyrimidine (29d)***

3-Bromo-5-chloropyrazolo[1,5-*a*]pyrimidine (400 mg, 0.43 mmol), 4-isopropylaniline (467 mg, 3.46 mmol, 2.0 equiv), CsF (263 mg, 1.73 mmol, 1.0 equiv), BnNEt_3_Cl (39 mg, 0.172 mmol,10 mol%), DMSO (12 mL); **29d** (525 mg, 1.59 mmol, 92%); ^1^H NMR (DMSO-*d*_6_, 500 MHz) δ 9.82 (s, 1H), 8.61 (d, *J* = 7.6 Hz, 1H), 7.99 (s, 1H), 7.83 (d, *J* = 8.5 Hz, 2H), 7.22 (d, *J* = 8.3 Hz, 2H), 6.53 (d, *J* = 7.6 Hz, 1H), 2.83 (sept, *J* = 6.9 Hz, 1H), 1.18 (d, *J* = 6.9 Hz, 6H); ^13^C NMR (DMSO-*d*_6_, 125 MHz) δ 153.9, 144.8, 143.9, 143.2, 138.0, 136.4, 127.0, 119.8, 102.3, 79.0, 33.3, 24.4; HRMS Calcd. for C_15_H_16_BrN_4_ [M + H]: 331.0558; Found: 331.0556 (∆ = 0.6 ppm).

***3-Bomo-5-(4-(chloro)anilin-1-yl)-pyrazolo[1,5-a]pyrimidine (29e)***

3-Bromo-5-chloropyrazolo[1,5-*a*]pyrimidine (400 mg, 1.73 mmol), 4-chloroaniline (439 mg, 3.46 mmol, 2.0 equiv), CsF (263 mg, 1.73 mmol, 1.0 equiv), BnNEt_3_Cl (39 mg, 0.172 mmol,10 mol%), DMSO (12 mL); **29e** (312 mg, 0.97 mmol, 56%); δ 10.04 (s, 1H), 8.67 (d, *J* = 7.5 Hz, 1H), 8.03 (s, 1H), 7.93 (d, *J* = 8.9 Hz, 2H), 7.41 (d, *J* = 8.9 Hz, 2H), 6.55 (d, *J* = 7.6 Hz, 1H); ^13^C NMR (DMSO-*d*_6_, 125 MHz) δ 153.7, 144.5, 144.1, 139.3, 136.8, 129.1, 126.5, 121.0, 102.3, 79.4; HRMS Calcd. for C_12_H_9_BrClN_4_ [M + H]: 322.9699; Found: 322.9694 (∆ = 1.5 ppm).

### 4.4. 3,5-Bis-Aminopyrazolo[1,5-a]Pyrimidines 30

General Procedure: A solution of 3-Bromo-5-(pyrrolidin-1-yl)pyrazolo[1,5-*a*]pyrimidine (**29a**) (50 mg, 0.19 mmol), morpholine (25 mg, 0.29 mmol, 1.5 equiv.), **L-1** (6 mg, 0.02 mmol, 10 mol%), CuI (2 mg, 0.01 mmol, 5 mol%), and K_2_CO_3_ (52 mg, 0.38 mmol, 2 equiv.) in diethyleneglycol (DEG, 1 mL) was stirred in a 10 mL microwave reactor vessel and heated at 80 °C for 1 h. After cooling to ambient temperature, H_2_O (5 mL) was added. The mixture was diluted with CH_2_Cl_2_ (5 mL) and the organic layer was separated. The aqueous layer was partitioned with CH_2_Cl_2_ (2 × 10 mL), and the combined organic layers were washed with brine (2 × 10 mL), followed by water (2 × 10 mL). The organic layer was dried over anhydrous magnesium sulfate, filtered, and removed under reduced pressure. The crude product was then purified using flash chromatography (EtOAc/Hexanes or MeOH/CH_2_Cl_2_) to give pure product **30e** (48 mg, 0.176 mmol, 92%).

***3-(N-(Isopropyl)amino)-5-(pyrrolidin-1-yl)pyrazolo[1,5-a]pyrimidine (30a)***

3-Bromo-5-(pyrrolidin-1-yl)pyrazolo[1,5-*a*]pyrimidine (50 mg, 0.19 mmol), isopropylamine (17 mg, 0.29 mmol, 1.5 equiv.), **L-1** (6 mg, 0.02 mmol, 10 mol%), CuI (2 mg, 0.01 mmol, 5 mol%), and K_2_CO_3_ (52 mg, 0.38 mmol, 2 equiv.); **30a** (42 mg, 0.171 mmol, 90%); ^1^H NMR (DMSO-*d*_6_, 500 MHz) δ 8.39 (d, *J* = 7.8 Hz, 1H), 7.51 (s, 1H), 6.23 (d, *J* = 7.7 Hz, 1H), 3.47–3.41 (m, 5H), 1.94 (t, *J* = 6.4 Hz, 4H), 1.07 (d, *J* = 6.3 Hz, 6H); ^13^C NMR (DMSO-*d*_6_, 125 MHz) δ 152.2, 138.7, 136.6, 135.6, 116.3, 97.7, 47.8, 47.0, 25.4, 23.7; HRMS Calcd. for C_13_H_20_N_5_ [M + H]: 246.1719; Found: 246.1712 (∆ = 2.8 ppm).

***3-(N-(Cyclohexyl)amino)-5-(pyrrolidin-1-yl)pyrazolo[1,5-a]pyrimidine (30b)***

3-Bromo-5-(pyrrolidin-1-yl)pyrazolo[1,5-*a*]pyrimidine (50 mg, 0.19 mmol), cyclohexylamine (29 mg, 0.29 mmol, 1.5 equiv.), **L-1** (6 mg, 0.02 mmol, 10 mol%), CuI (2 mg, 0.01 mmol, 5 mol%), and K_2_CO_3_ (52 mg, 0.38 mmol, 2 equiv.); **30b** (49 mg, 0.172 mmol, 91%); ^1^H NMR (DMSO-*d*_6_, 500 MHz) δ 8.38 (d, *J* = 7.7 Hz, 1H), 7.50 (s, 1H), 6.22 (d, *J* = 7.7 Hz, 1H), 3.47 (‘bs’, 4H), 3.12 (t, *J* = 9.7 Hz, 1H), 1.95 (t, *J* = 6.3 Hz, 4H), 1.89–1.87 (m, 2H), 1.70– 1.68 (m, 2H), 1.57–1.55 (m, 1H), 1.26–1.09 (m, 5H); ^13^C NMR (DMSO-*d*_6_, 125 MHz) δ 152.1, 138.5, 136.6, 135.6, 115.9, 97.7, 72.7, 60.7, 55.4, 47.0, 33.8, 26.2, 25.4, 25.1; HRMS Calcd. for C_16_H_24_N_5_ [M + H]: 286.2032; Found: 286.2024 (∆ = 2.8 ppm).

***3-(N-((Cyclopropyl)methyl)amino)-5-(pyrrolidin-1-yl)pyrazolo[1,5-a]pyrimidine (30c)***

3-Bromo-5-(pyrrolidin-1-yl)pyrazolo[1,5-*a*]pyrimidine (50 mg, 0.19 mmol), cyclopropylamine (21 mg, 0.29 mmol, 1.5 equiv.), **L-1** (6 mg, 0.02 mmol, 10 mol%), CuI (2 mg, 0.01 mmol, 5 mol%), and K_2_CO_3_ (52 mg, 0.38 mmol, 2 equiv.); **30c** (29 mg, 0.113 mmol, 60%); ^1^H NMR (CDCl_3_, 500 MHz) δ 8.11 (d, *J* = 7.7 Hz, 1H), 7.61 (s, 1H), 6.04 (d, *J* = 7.2 Hz, 1H), 3.55 (“bs”, 4H), 2.98 (d, *J* = 6.9 Hz, 2H), 2.02 (t, *J* = 6.6 Hz, 4H), 1.17–1.13 (m, 1H), 0.53 (dd, J = 12.6, 5.5 Hz, 2H), 0.23 (dd, J = 10, 4.9 Hz, 2H); ^13^C NMR (CDCl_3_, 125 MHz) δ 152.2, 138.7, 135.3, 134.7, 117.5, 97.2, 54.1, 46.9, 25.5, 11.6, 3.4; HRMS Calcd. for C_14_H_20_N_5_ [M + H]: 258.1719; Found: 258.1704 (∆ = 2.7 ppm).

***3-[2-(tert-Butyloxycarbonyl)amino)-1-N-methylamino)ethyl]-5-(pyrrolidin-1-yl)pyrazolo[1,5-a]pyrimidine (30d)***

3-Bromo-5-(pyrrolidin-1-yl)pyrazolo[1,5-*a*]pyrimidine (50 mg, 0.19 mmol), 1-(*tert*-butyloxycarbonylamino)-2-(*N*-methylamino)ethane (51 mg, 0.29 mmol, 1.5 equiv.), **L-1** (6 mg, 0.02 mmol, 10 mol%), CuI (2 mg, 0.01 mmol, 5 mol%), and K_2_CO_3_ (52 mg, 0.38 mmol, 2 equiv.); **30d** (60 mg, 0.167 mmol, 88%); ^1^H NMR (CDCl_3_, 500 MHz) δ 8.07 (d, *J* = 7.7 Hz, 1H), 7.67 (s, 1H), 6.42 (s, 1H), 6.02 (d, *J* = 7.7 Hz, 1H), 3.53 (“bs”, 4H), 3.12–3.09 (m, 2H), 2.97–2.95 (m, 2H), 2.77 (s, 3H), 1.97 (“bs”, 4H), 1.38 (s, 9H); ^13^C NMR CDCl_3_, 125 MHz) δ 156.2, 152.7, 141.5, 137.4, 135.1, 119.6, 97.6, 78.5, 57.6, 47.1, 43.0, 38.8, 28.6, 25.5; HRMS Calcd. for C_18_H_29_N_6_O_2_ [M + H]: 361.2352; Found: 361.2344 (∆ = 2.2 ppm).

***3-(Morpholin-1-yl)-5-(pyrrolidin-1-yl)pyrazolo[1,5-a]pyrimidine (30e)***

(a) 50 mg scale: 3-bromo-5-(pyrrolidin-1-yl)pyrazolo[1,5-*a*]pyrimidine **29a** (50 mg, 0.19 mmol), morpholine (25 mg, 0.29 mmol, 1.5 equiv.), **L-1** (6 mg, 0.02 mmol, 10 mol%), CuI (2 mg, 0.01 mmol, 5 mol%), and K_2_CO_3_ (52 mg, 0.38 mmol, 2 equiv.); **30e** (48 mg, 0.176 mmol, 92%); (b) 1.0 g scale: **29a** (1.0 g, 3.8 mmol), morpholine (496 mg, 5.7 mmol, 1.5 equiv.), **L-1** (105 mg, 0.38 mmol, 10 mol%), CuI (36 mg, 0.19 mmol, 5 mol%), and K_2_CO_3_ (1.04 g, 7.6 mmol, 2 equiv.); **30e** (913 mg, 3.34 mmol, 88%); ^1^H NMR (DMSO-*d*_6_, 500 MHz) δ 8.45 (d, *J* = 7.8 Hz, 1H), 7.58 (s, 1H), 6.30 (d, *J* = 7.7 Hz, 1H), 3.74 (t, *J* = 4.6 Hz, 4H), 3.47 (t, *J* = 6.6 Hz, 4H), 3.09 (t, *J* = 4.4 Hz, 4H), 1.95 (“bs”, 4H); ^13^C NMR (CDCl_3_, 125 MHz) δ 152.0, 139.4, 134.8, 134.3, 120.6, 97.4, 67.0, 51.5, 46.8, 25.4; HRMS Calcd. for C_14_H_20_N_5_O [M + H]: 274.1668; Found: 274.1659 (∆ = 3.3 ppm).

***3-(Piperidin-1-yl)-5-(pyrrolidin-1-yl)pyrazolo[1,5-a]pyrimidine (30f)***

3-Bromo-5-(pyrrolidin-1-yl)pyrazolo[1,5-*a*]pyrimidine (50 mg, 0.19 mmol), piperidine (25 mg, 0.29 mmol, 1.5 equiv.), **L-1** (6 mg, 0.02 mmol, 10 mol%), CuI (2 mg, 0.01 mmol, 5 mol%), and K_2_CO_3_ (52 mg, 0.38 mmol, 2 equiv.); **30f** (48 mg, 0.177 mmol, 93%); ^1^H NMR (DMSO-*d*_6_, 500 MHz) δ 8.42 (d, *J* = 7.8 Hz, 1H), 7.56 (s, 1H), 6.28 (d, *J* = 7.7 Hz, 1H), 3.47 (t, *J* = 6.5 Hz, 4H), 3.05 (t, *J* = 5.1 Hz, 4H), 1.95 (“bs”, 4H), 1.65–1.61 (m, 4H), 1.51– 1.46 (m, 2H); ^13^C NMR (DMSO-*d*_6_, 125 MHz) δ 151.9, 138.5, 135.7, 134.5, 121.7, 97.9, 52.2, 46.9, 25.9, 25.4, 24.4; HRMS Calcd. for C_15_H_22_N_5_ [M + H]: 272.1875; Found: 272.1875 (∆ = 2.9 ppm).

***tert-Butyl 4-(5-(pyrrolidin-1-yl)pyrazolo[1,5-a]pyrimidin-3-yl)piperazine-1-carboxylate (30g)***

3-Bromo-5-(pyrrolidin-1-yl)pyrazolo[1,5-*a*]pyrimidine (50 mg, 0.19 mmol), *tert*-butyl piperazine-1-carboxylate (54 mg, 0.29 mmol, 1.5 equiv.), **L-1** (6 mg, 0.02 mmol, 10 mol%), CuI (2 mg, 0.01 mmol, 5 mol%), and K_2_CO_3_ (52 mg, 0.38 mmol, 2 equiv.); **30g** (45 mg, 0.120 mmol, 64%); ^1^H NMR (DMSO-*d*_6_, 500 MHz) δ 8.44 (d, *J* = 7.7 Hz, 1H), 7.59 (s, 1H), 6.29 (d, *J* = 7.7 Hz, 1H), 3.48–3.30 (m, 8H), 3.04 (t, *J* = 4.7 Hz, 4H), 1.94 (“bs”, 4H), 1.42 (s, 9H); ^13^C NMR (DMSO-*d*_6_, 125 MHz) δ 154.4, 152.1, 138.8, 135.8, 134.8, 120.2, 98.1, 79.3, 50.9, 47.0, 28.5, 25.4; HRMS Calcd. for C_19_H_29_N_6_O_2_ [M + H]: 373.2352; Found: 373.2347 (∆ = 1.3 ppm).

***3-(4-(tert-Butyl)anilin-1-yl)-5-(pyrrolidin-1-yl)pyrazolo[1,5-a]pyrimidine (30h)***

3-Bromo-5-(pyrrolidin-1-yl)pyrazolo[1,5-*a*]pyrimidine (50 mg, 0.19 mmol), 4-*tert*-butylaniline (43 mg, 0.29 mmol, 1.5 equiv.), **L-1** (6 mg, 0.02 mmol, 10 mol%), CuI (2 mg, 0.01 mmol, 5 mol%), and K_2_CO_3_ (52 mg, 0.38 mmol, 2 equiv.); **30h** (56 mg, 0.167 mmol, 88%); ^1^H NMR (DMSO-*d*_6_, 500 MHz) δ 8.53 (d, *J* = 7.7 Hz, 1H), 7.78 (s, 1H), 7.07 (d, *J* = 8.7 Hz, 2H), 6.87 (s, 1H), 6.55 (dd, *J* = 6.8, 1.9 Hz, 2H), 6.35 (d, *J* = 7.8Hz, 1H), 3.45 (t, *J* = 6.4 Hz, 4H), 1.92 (“bs”, 4H), 1.21 (s, 9H); ^13^C NMR (DMSO-*d*_6_, 125 MHz) δ 153.4, 146.7, 143.3, 142.4, 138.8, 136.0, 125.8, 112.7, 108.4, 98.3, 47.2, 33.9, 32.0, 25.4; HRMS Calcd. for C_20_H_26_N_5_ [M + H]: 336.2188; Found: 336.2196 (∆ = 2.4 ppm).

***3-(4-(Phenoxy)anilin-1-yl)-5-(pyrrolidin-1-yl)pyrazolo[1,5-a]pyrimidine (30i)***

3-Bromo-5-(pyrrolidin-1-yl)pyrazolo[1,5-*a*]pyrimidine (50 mg, 0.19 mmol), 4-phenoxyaniline (54 mg, 0.29 mmol, 1.5 equiv.), **L-1** (6 mg, 0.02 mmol, 10 mol%), CuI (2 mg, 0.01 mmol, 5 mol%), and K_2_CO_3_ (52 mg, 0.38 mmol, 2 equiv.); **30i** (59 mg, 0.160 mmol, 84%); ^1^H NMR (DMSO-*d*_6_, 500 MHz) δ 8.54 (d, *J* = 7.7 Hz, 1H), 7.82 (s, 1H), 7.30 (dd, *J* = 8.4, 7.6 Hz, 2H), 7.10 (s, 1H), 7.00 (t, *J* = 7.4 Hz, 1H), 6.86 (d, *J* = 7.8 Hz, 2H), 6.82 (dd, *J* = 12.2, 3.3 Hz, 2H), 6.66–6.63 (m, 2H), 6.36 (d, *J* = 7.8 Hz, 1H), 3.46 (t, *J* = 6.4 Hz, 4H) 1.93 (“bs”, 4H); ^13^C NMR (DMSO-*d*_6_, 125 MHz) δ 159.4, 153.4, 146.3, 145.9, 143.2, 142.3, 136.1, 130.1, 122.2, 121.3, 116.9, 114.1, 108.3, 98.3, 47.2, 25.3; HRMS Calcd. for C_22_H_22_N_5_O [M + H]: 372.1824; Found: 372.1830 (∆ = 1.6 ppm).

***3-((4-Methoxy-3-methyl)anilin-1-yl)-5-(pyrrolidin-1-yl)pyrazolo[1,5-a]pyrimidine (30j)***

3-Bromo-5-(pyrrolidin-1-yl)pyrazolo[1,5-*a*]pyrimidine (50 mg, 0.19 mmol), 4-methoxy-3-methylaniline (40 mg, 0.29 mmol, 1.5 equiv.), **L-1** (6 mg, 0.02 mmol, 10 mol%), CuI (2 mg, 0.01 mmol, 5 mol%), and K_2_CO_3_ (52 mg, 0.38 mmol, 2 equiv.); **30j** (50 mg, 0.156 mmol, 82%); ^1^H NMR (DMSO-*d*_6_, 500 MHz) δ 8.52 (d, *J* = 7.7 Hz, 1H), 7.76 (s, 1H), 6.66 (d, *J* = 8.7 Hz, 1H), 6.60 (s, 1H), 6.48 (d, *J* = 2.4 Hz, 1H), 6.40 (dd, *J* = 8.7, 2.7 Hz, 1H), 6.34 (d, *J* = 7.8 Hz, 1H), 3.65 (s, 3H), 3.45 (t, *J* = 6.4Hz, 4H), 2.03 (s, 3H), 1.93 (“bs”, 4H); ^13^C NMR (DMSO-*d*_6_, 125 MHz) δ 153.3, 149.6, 143.0, 142.8, 142.1, 136.0, 126.3, 116.1, 112.0, 110.9, 109.2, 98.2, 56.1, 47.1, 25.4, 16.7; HRMS Calcd. for C_18_H_22_N_5_O [M + H]: 324.1824; Found: 324.1817 (∆ = 2.2 ppm).

***3-(N-(Pyrimidin-2-yl)amino)-5-(pyrrolidin-1-yl)pyrazolo[1,5-a]pyrimidine (30k)***

3-Bromo-5-(pyrrolidin-1-yl)pyrazolo[1,5-*a*]pyrimidine (50 mg, 0.19 mmol), 2-aminopyrimidine (28 mg, 0.29 mmol, 1.5 equiv.), **L-1** (6 mg, 0.02 mmol, 10 mol%), CuI (2 mg, 0.01 mmol, 5 mol%), and K_2_CO_3_ (52 mg, 0.38 mmol, 2 equiv.); **30k** (45 mg, 0.160 mmol, 84%); ^1^H NMR (DMSO-*d*_6_, 500 MHz) δ 8.53 (d, *J* = 7.7 Hz, 1H), 8.31 (t, *J* = 4.7 Hz, 3H), 7.95 (s, 1H), 6.66 (t, *J* = 4.8 Hz, 1H), 6.35 (d, *J* = 7.7 Hz, 1H), 3.47 (t, *J* = 6.6 Hz, 4H), 1.93 (“bs”, 4H); ^13^C NMR (DMSO-*d*_6_, 125 MHz) δ 162.3, 158.5, 153.4, 142.1, 141.6, 135.8, 111.4, 105.9, 98.3, 47.1, 25.4; HRMS Calcd. for C_14_H_16_N_7_ [M + H]: 282.1467; Found: 282.1459 (∆ = 2.8 ppm).

***3-(N-(Pyrazol-3-yl)amino)-5-(pyrrolidin-1-yl)pyrazolo[1,5-a]pyrimidine (30l)***

3-Bromo-5-(pyrrolidin-1-yl)pyrazolo[1,5-*a*]pyrimidine (50 mg, 0.19 mmol), 3-aminopyrazole (24 mg, 0.29 mmol, 1.5 equiv.), **L-1** (6 mg, 0.02 mmol, 10 mol%), CuI (2 mg, 0.01 mmol, 5 mol%), and K_2_CO_3_ (52 mg, 0.38 mmol, 2 equiv.); **30l** (42 mg, 0.156 mmol, 82%); ^1^H NMR (DMSO-*d*_6_, 500 MHz) δ 8.64 (d, *J* = 7.8 Hz, 1H), 8.00 (s, 1H), 7.26 (d, *J* = 1.8 Hz, 1H), 6.47 (d, *J* = 7.8 Hz, 1H), 5.65 (s, 2H), 5.41 (d, *J* = 1.8 Hz, 1H), 3.50 (“bs”, 4H), 1.97 (“bs”, 4H); ^13^C NMR (DMSO, 125 MHz) δ 153.7, 148.0, 140.4, 139.7, 139.4, 136.6, 108.1, 98.8, 88.7, 47.3, 25.8; HRMS Calcd. for C_13_H_16_N_7_ [M + H]: 270.1467; Found: 270.1460 (∆ = 2.6 ppm).

***3-(N-(5-Bromopyrazol-3-yl)amino)-5-(pyrrolidin-1-yl)pyrazolo[1,5-a]pyrimidine (30m)***

3-Bromo-5-(pyrrolidin-1-yl)pyrazolo[1,5-*a*]pyrimidine (50 mg, 0.19 mmol), 3-amino-5-bromopyrazole (47 mg, 0.29 mmol, 1.5 equiv.), **L-1** (6 mg, 0.02 mmol, 10 mol%), CuI (2 mg, 0.01 mmol, 5 mol%), and K_2_CO_3_ (52 mg, 0.38 mmol, 2 equiv.); **30m** (44 mg, 0.127 mmol, 67%); ^1^H NMR (DMSO-*d*_6_, 500 MHz) δ 8.65 (d, *J* = 7.8 Hz, 1H), 8.01 (s, 1H), 7.41 (s, 1H), 6.48 (d, *J* = 7.8 Hz, 1H), 5.79 (bs, 1H), 5.76 (s, 1H), 3.51 (“bs”, 4H), 1.98 (“bs”, 4H); ^13^C NMR (DMSO-*d*_6_, 125 MHz) δ 153.8, 145.4, 139.9, 139.6, 136.6, 107.6, 99.0, 74.0, 47.4, 25.8, 24.8; HRMS Calcd. for C_13_H_15_N_7_Br [M + H]: 348.0572; Found: 348.0566 (∆ = 1.7 ppm).

***N-(2-(2-(2-(2-Azidoethoxy)ethoxy)ethoxy)ethyl)-5-(pyrrolidin-1-yl)pyrazolo[1,5-a]pyrimidin-3-amine (30n)***

3-Bromo-5-(pyrrolidin-1-yl)pyrazolo[1,5-*a*]pyrimidine (50 mg, 0.19 mmol), 2-(2-(2-(2-azidoethoxy)ethoxy)ethoxy)ethan-1-amine (63 mg, 0.29 mmol, 1.5 equiv.), **L-1** (6 mg, 0.02 mmol, 10 mol%), CuI (2 mg, 0.01 mmol, 5 mol%), and K_2_CO_3_ (52 mg, 0.38 mmol, 2 equiv.); **30n** (51 mg, 0.126 mmol, 66%); ^1^H NMR (CDCl_3_, 500 MHz) δ 8.02 (d, *J* = 7.7 Hz, 1H), 7.60 (s, 1H), 6.04 (d, *J* = 7.7 Hz, 1H), 3.70–3.66 (m, 12H), 3.54 (“bs”, 4H), 3.37 (t, *J* = 5.0 Hz, 2H), 3.33 (t, *J* = 5.3 Hz, 2H), 2.03–2.00 (m, 4H); ^13^C NMR (CDCl_3_, 125 MHz) δ 152.1, 137.5, 135.6, 134.7, 117.6, 97.8, 70.30, 70.29, 70.23, 70.17, 70.13, 69.7, 50.4, 47.3, 47.0, 25.4; HRMS Calcd. for C_18_H_29_N_8_O_3_ [M + H]: 405.2363; Found: 405.2382 (∆ = 4.7 ppm).

***5-(N-(Isopropyl)amino)-3-(2-N-(thiophen-2-yl)ethylamino)pyrazolo[1,5-a]pyrimidine (30o)***

3-Bromo-5-(N-(isopropyl)amino)pyrazolo[1,5-*a*]pyrimidine (50 mg, 0.20 mmol), 2-(thiophen-2-yl)ethan-1-amine (38 mg, 0.30 mmol, 1.5 equiv.), **L-1** (6 mg, 0.02 mmol, 10 mol%), CuI (2 mg, 0.01 mmol, 5 mol%), and K_2_CO_3_ (52 mg, 0.38 mmol, 2 equiv.); **30o** (54 mg, 0.180 mmol, 90%); ^1^H NMR (DMSO-*d*_6_, 500 MHz) δ 8.22 (d, *J* = 7.6 Hz, 1H), 7.47 (s, 1H), 7.31 (dd, *J* = 5.1, 1.2 Hz, 1H), 7.02 (d, *J* = 7.3 Hz, 1H), 6.94 (dd, *J* = 5.1, 3.4 Hz, 1H), 6.90 (dd, *J* = 3.3 Hz, Jz = 1.0 Hz, 1H), 6.05 (d, *J* = 7.6 Hz, 1H), 4.12–4.06 (m, 1H), 3.82 (s, 1H), 3.32 (t, *J* = 7.1 Hz, 2H), 3.02 (t, *J* = 7.2 Hz, 2H), 1.17 (d, *J* = 6.5 Hz, 6H); ^13^C NMR (DMSO-*d*_6_, 125 MHz) δ 153.1, 142.8, 137.8, 135.0, 134.8, 127.4, 125.4, 124.1, 117.3, 100.3, 49.6, 42.0, 30.5, 22.7; HRMS Calcd. for C_15_H_20_N_5_S [M + H]: 302.1439; Found: 302.1428 (∆ = 3.6 ppm).

***3-(4-(tert-Butyl)anilin-1-yl)-5-(N-(isopropyl)amino)pyrazolo[1,5-a]pyrimidine (30p)***

3-Bromo-5-(N-(isopropyl)amino)pyrazolo[1,5-*a*]pyrimidine (50 mg, 0.20 mmol), 4-*tert*-butylaniline (45 mg, 0.30 mmol, 1.5 equiv.), **L-1** (6 mg, 0.02 mmol, 10 mol%), CuI (2 mg, 0.01 mmol, 5 mol%), and K_2_CO_3_ (52 mg, 0.38 mmol, 2 equiv.); **30p** (56 mg, 0.173 mmol, 87%); ^1^H NMR (DMSO-*d*_6_, 500 MHz) δ 8.35 (d, *J* = 7.6 Hz, 1H), 7.71 (s, 1H), 7.22 (d, *J* = 7.6 Hz, 1H), 7.06 (d, *J* = 8.7 Hz, 2H), 6.84 (s, 1H), 6.56 (d, *J* = 8.7 Hz, 2H), 6.16 (d, *J* = 7.6 Hz, 1H), 4.06–4.00 (m, 1H), 1.20 (s, 9H), 1.12 (d, *J* = 6.5 Hz, 6H); ^13^C NMR (DMSO-*d*_6_, 125 MHz) δ 154.5, 146.5, 143.1, 141.6, 138.9, 135.5, 125.7, 112.9, 108.9, 100.6, 41.9, 33.9, 31.9, 22.7; HRMS Calcd. for C_19_H_26_N_5_ [M + H]: 324.2188; Found: 324.2174 (∆ = 4.3 ppm).

***5-(N-(Isopropyl)amino)-3-(morpholin-1-yl)pyrazolo[1,5-a]pyrimidine (30q)***

3-Bromo-5-(N-(isopropyl)amino)pyrazolo[1,5-*a*]pyrimidine (50 mg, 0.20 mmol), morpholine (26 mg, 0.30 mmol, 1.5 equiv.), **L-1** (6 mg, 0.02 mmol, 10 mol%), CuI (2 mg, 0.01 mmol, 5 mol%), and K_2_CO_3_ (52 mg, 0.38 mmol, 2 equiv.); **30q** (47 mg, 0.180 mmol, 90%); ^1^H NMR (DMSO-*d*_6_, 500 MHz) δ 8.28 (d, *J* = 7.6 Hz, 1H), 7.53 (s, 1H), 7.14 (d, *J* = 7.0 Hz, 1H), 6.11 (d, *J* = 7.6 Hz, 1H), 4.06–4.00 (m, 1H), 3.72 (t, *J* = 4.6 Hz, 4H), 3.08 (“bs”, 4H), 1.17 (d, *J* = 6.5 Hz, 6H); ^13^C NMR (DMSO-*d*_6_, 125 MHz) δ 152.0, 137.5, 134.3, 133.0, 119.8, 99.3, 65.5, 50.6, 41.1, 21.5; HRMS Calcd. for C_13_H_20_N_5_O [M + H]: 262.1668; Found: 262.1656 (∆ = 4.6 ppm).

***3-(N-(Isopropyl)amino)-5-(4-(methoxy)anilin-1-yl)pyrazolo[1,5-a]pyrimidine (30r)***

3-Bromo-5-(*4-(methoxy)anilin-1-yl*)-pyrazolo[1,5-*a*]pyrimidine (50 mg, 0.16 mmol), isopropylamine (14 mg, 0.24 mmol, 1.5 equiv.), **L-1** (4.4 mg, 0.016 mmol, 10 mol%), CuI (1.5 mg, 0.008 mmol, 5 mol%), and K_2_CO_3_ (44 mg, 0.32 mmol, 2 equiv.); **30r** (43 mg, 0.145 mmol, 91%); ^1^H NMR (DMSO-*d*_6_, 500 MHz) δ 9.31 (s, 1H), 8.40 (d, *J* = 7.5 Hz, 1H), 7.78 (d, *J* = 8.9 Hz, 2H), 7.55 (s, 1H), 6.91 (d, *J* = 8.9 Hz, 2H), 6.29 (d, *J* = 7.6 Hz, 1H), 3.74 (s, 3H), 3.65–3.60 (bs, 1H), 3.53 (sept, *J* = 6.3 Hz, 1H), 1.12 (d, *J* = 6.2 Hz, 6H); ^13^C NMR (DMSO-*d*_6_, 125 MHz) δ 154.6, 150.7, 137.2, 136.3, 135.7, 134.4, 120.5, 117.8, 114.3, 100.7, 55.6, 47.9, 23.7; HRMS Calcd. for C_16_H_20_N_5_O [M + H]: 298.1668; Found: 298.1668 (∆ = 0 ppm).

***5-(4-(Methoxy)anilin-1-yl)-3-(morpholin-1-yl)pyrazolo[1,5-a]pyrimidine (30s)***

3-Bromo-5-(*4-(methoxy)anilin-1-yl*)-pyrazolo[1,5-*a*]pyrimidine (50 mg, 0.16 mmol), morpholine (21 mg, 0.24 mmol, 1.5 equiv.), **L-1** (4.4 mg, 0.016 mmol, 10 mol%), CuI (1.5 mg, 0.008 mmol, 5 mol%), and K_2_CO_3_ (44 mg, 0.32 mmol, 2 equiv.); **30s** (47 mg, 0.145 mmol, 91%); ^1^H NMR (DMSO-*d*_6_, 500 MHz) δ 9.41 (s, 1H), 8.47 (d, *J* = 7.6 Hz, 1H), 7.69 (d, *J* = 9.0 Hz, 2H), 7.65 (s, 1H), 6.92 (d, *J* = 9.1 Hz, 2H), 6.35 (d, *J* = 7.6 Hz, 1H), 3.78 (t, *J* = 4.6 Hz, 4H), 3.74 (s, 3H), 3.78 (t, *J* = 4.6 Hz, 4H); ^13^C NMR (DMSO-*d*_6_, 125 MHz) δ 154.9, 150.7, 137.3, 136.0, 134.3, 134.0, 121.9, 120.7, 114.4, 101.0, 66.6, 55.7, 51.7; HRMS Calcd. for C_17_H_20_N_5_O_2_ [M + H]: 326.1617; Found: 326.1611 (∆ = 1.8 ppm).

***5-(4-(Methoxy)anilin-1-yl)-3-(anilin-1-yl)pyrazolo[1,5-a]pyrimidine (30t)***

3-Bromo-5-(*4-(methoxy)anilin-1-yl*)-pyrazolo[1,5-*a*] pyrimidine (50 mg, 0.16 mmol), aniline (22 mg, 0.24 mmol, 1.5 equiv.), **L-1** (4.4 mg, 0.016 mmol, 10 mol%), CuI (1.5 mg, 0.008 mmol, 5 mol%), and K_2_CO_3_ (44 mg, 0.32 mmol, 2 equiv.); **30t** (43 mg, 0.13 mmol, 81%); ^1^H NMR (DMSO-*d*_6_, 500 MHz) δ 9.47 (s, 1H), 8.54 (d, *J* = 7.6 Hz, 1H), 7.86 (s, 1H), 7.64 (d, *J* = 9.0 Hz, 2H), 7.21 (s, 1H), 7.09 (t, *J* = 7.6 Hz, 2H), 6.75 (d, *J* = 9.0 Hz, 2H), 6.70 (d, *J* = 7.9 Hz, 2H), 6.61 (t, *J* = 7.3 Hz, 1H), 6.41 (d, *J* = 7.6 Hz, 1H), 3.69 (s, 3H); ^13^C NMR (DMSO-*d*_6_, 125 MHz) δ 154.8, 152.1, 148.5, 141.6, 141.5, 136.2, 133.9, 129.2, 120.9, 116.9, 114.2, 113.6, 109.9, 101.2, 55.6; HRMS Calcd. for C_19_H_18_N_5_O [M + H]: 332.1511; Found: 332.1524 (∆ = 3.9 ppm).

***5-(4-(Methoxy)anilin-1-yl)-3-(N-(pyrimidin-2-yl)amino)pyrazolo[1,5-a]pyrimidine (30u)***

3-Bromo-5-(*4-(methoxy)anilin-1-yl*)pyrazolo[1,5-*a*]pyrimidine (50 mg, 0.16 mmol), 2-aminopyrimidine (23 mg, 0.24 mmol, 1.5 equiv.), **L-1** (4.4 mg, 0.016 mmol, 10 mol%), CuI (1.5 mg, 0.008 mmol, 5 mol%), and K_2_CO_3_ (44 mg, 0.32 mmol, 2 equiv.); **30u** (42 mg, 0.13 mmol, 81%); ^1^H NMR (DMSO-*d*_6_, 500 MHz) δ 9.48 (s, 1H), 8.63 (s, 1H), 8.54 (d, *J* = 7.6 Hz, 1H), 8.35 (d, *J* = 4.7 Hz, 2H), 8.04 (s, 1H), 7.73 (d, *J* = 8.9 Hz, 2H), 6.83 (d, *J* = 9.0 Hz, 2H), 6.71 (t, *J* = 4.7 Hz, 1H), 6.41 (d, *J* = 7.6 Hz, 1H), 3.72 (s, 3H); ^13^C NMR (DMSO-*d*_6_, 125 MHz) δ 166.9, 163.2, 159.6, 156.9, 145.7, 145.4, 140.8, 138.7, 125.7, 119.0, 116.3, 112.4, 106.0; HRMS Calcd. for C_17_H_16_N_7_O [M + H]: 334.1416; Found: 334.1421 (∆ = 1.5 ppm).

***3-(N-(Isopropyl)amino)-5-(4-(isopropyl)anilin-1-yl)pyrazolo[1,5-a]pyrimidine (30v)***

3-Bromo-5-(*4-(isopropyl)anilin-1-yl*)pyrazolo[1,5-*a*]pyrimidine (50 mg, 0.15 mmol), isopropylamine (14 mg, 0.23 mmol, 1.5 equiv.), **L-1** (4 mg, 0.015 mmol, 10 mol%), CuI (1.4 mg, 0.007 mmol, 5 mol%), and K_2_CO_3_ (41 mg, 0.30 mmol, 2 equiv.); **30v** (42 mg, 0.136 mmol, 90%); ^1^H NMR (DMSO-*d*_6_, 500 MHz) δ 8.10 (d, *J* = 7.6 Hz, 1H), 7.61 (s, 1H), 7.34 (d, *J* = 8.4 Hz, 2H), 7.19 (s, 1H), 7.16 (d, *J* = 8.4 Hz, 2H), 6.58 (bs, 1H), 6.13 (d, *J* = 7.6 Hz, 1H), 3.38 (sept, *J* = 6.3 Hz, 1H), 2.84 (sept, *J* = 6.9 Hz, 1H), 1.20 (d, *J* = 6.9 Hz, 6H), 1.15 (d, *J* = 6.3 Hz, 6H); ^13^C NMR (DMSO-*d*_6_, 125 MHz) δ 150.8, 142.2, 138.8, 137.2, 136.5, 135.8, 126.9, 119.0, 117.9, 100.9, 48.0, 33.3, 24.5, 23.6; HRMS Calcd. for C_18_H_24_N_5_ [M + H]: 310.2032; Found: 310.2027 (∆ = 1.6 ppm).

***5-(4-(Isopropyl)anilin-1-yl)-3-(morpholin-1-yl)pyrazolo[1,5-a]pyrimidine (30w)***

3-Bromo-5-(*4-(isopropyl)anilin-1-yl*)pyrazolo[1,5-*a*]pyrimidine (50 mg, 0.15 mmol), morpholine (20 mg, 0.23 mmol, 1.5 equiv.), **L-1** (4 mg, 0.015 mmol, 10 mol%), CuI (1.4 mg, 0.007 mmol, 5 mol%), and K_2_CO_3_ (41 mg, 0.30 mmol, 2 equiv.); **30w** (44 mg, 0.13 mmol, 87%); ^1^H NMR (DMSO-*d*_6_, 500 MHz) δ 9.52 (s, 1H), 8.46 (d, *J* = 7.6 Hz, 1H), 7.68 (d, *J* = 8.4 Hz, 2H), 7.66 (s, 1H), 7.18 (d, *J* = 8.5 Hz, 2H), 6.39 (d, *J* = 7.6 Hz, 1H), 3.77 (t, *J* = 4.6 Hz, 4H), 3.13 (t, *J* = 4.5 Hz, 4H), 2.84 (sept, *J* = 6.9 Hz, 1H), 1.18 (d, *J* = 6.9 Hz, 6H); ^13^C NMR (DMSO-*d*_6_, 125 MHz) δ 150.7, 142.5, 138.5, 137.4, 136.0, 134.4, 126.9, 119.2, 101.2, 66.5, 51.6, 33.3, 24.5; HRMS Calcd. for C_19_H_24_N_5_O [M + H]: 338.1981; Found: 338.1984 (∆ = 0.9 ppm).

***5-(4-(Isopropyl)anilin-1-yl)-3-(anilin-1-yl)pyrazolo[1,5-a]pyrimidine (30x)***

3-Bromo-5-(*4*-(isopropyl)anilin-1-yl)pyrazolo[1,5-*a*]pyrimidine (50 mg, 0.15 mmol), aniline (21 mg, 0.23 mmol, 1.5 equiv.), **L-1** (4 mg, 0.015 mmol, 10 mol%), CuI (1.4 mg, 0.007 mmol, 5 mol%), and K_2_CO_3_ (41 mg, 0.30 mmol, 2 equiv.); **30x** (41 mg, 0.12 mmol, 80%); ^1^H NMR (DMSO-*d*_6_, 500 MHz) δ 9.56 (s, 1H), 8.54 (d, *J* = 7.5 Hz, 1H), 7.86 (s, 1H), 7.61 (d, *J* = 8.2 Hz, 2H), 7.20 (s, 1H), 7.10 (t, *J* = 7.2 Hz, 2H), 7.02 (d, *J* = 7.9 Hz, 2H), 6.70 (d, *J* = 8.1 Hz, 2H), 6.61 (t, *J* = 7.2 Hz, 1H), 6.44 (d, *J* = 7.5 Hz, 1H), 2.78 (sept, *J* = 6.8 Hz, 1H), 1.14 (d, *J* = 6.8 Hz, 6H); ^13^C NMR (DMSO-*d*_6_, 125 MHz) δ 152.1, 148.3, 142.5, 141.5, 141.4, 138.3, 136.2, 129.2, 126.7, 119.5, 117.0, 113.6, 110.1, 101.3, 33.2, 24.4; HRMS Calcd. for C_21_H_22_N_5_ [M + H]: 344.1875; Found: 344.1865 (∆ = 2.9 ppm).

***5-(4-(Isopropyl)anilin-1-yl)-3-(N-(pyrimidin-2-yl)amino)pyrazolo[1,5-a]pyrimidine (30y)***

3-Bromo-5-(*4*-(isopropyl)anilin-1-yl)pyrazolo[1,5-*a*]pyrimidine (50 mg, 0.15 mmol), 2-aminopyrimidine (22 mg, 0.23 mmol, 1.5 equiv.), **L-1** (4 mg, 0.015 mmol, 10 mol%), CuI (1.4 mg, 0.007 mmol, 5 mol%), and K_2_CO_3_ (41 mg, 0.30 mmol, 2 equiv.); **30y** (36 mg, 0.104 mmol, 69%); ^1^H NMR (DMSO-*d*_6_, 500 MHz) δ 9.54 (s, 1H), 8.64 (s, 1H), 8.56 (d, *J* = 7.5 Hz, 1H), 8.36 (d, *J* = 4.7 Hz, 2H), 8.05 (s, 1H), 7.71 (d, *J* = 8.5 Hz, 2H), 7.11 (d, *J* = 8.5 Hz, 2H), 6.71 (t, *J* = 4.7 Hz, 1H), 6.44 (d, *J* = 7.6 Hz, 1H), 2.83 (sept, *J* = 6.9 Hz, 1H), 1.18 (d, *J* = 6.9 Hz, 6H); ^13^C NMR (DMSO-*d*_6_, 125 MHz) δ 162.1, 158.5, 152.1, 142.4, 140.9, 140.6, 138.4, 136.1, 126.8, 119.5, 111.6, 107.8, 101.3, 33.3, 24.5; HRMS Calcd. for C_19_H_20_N_7_ [M + H]: 346.1780; Found: 346.1784 (∆ = 1.2 ppm).

***5-(4-(Chloro)anilin-1-yl)-3-(N-isopropylamino)pyrazolo[1,5-a]pyrimidine (30z)***

3-Bromo-5*-(4*-(chloro)anilin-1-yl)pyrazolo[1,5-*a*]pyrimidine (50 mg, 0.16 mmol), isopropylamine (14 mg, 0.24 mmol, 1.5 equiv.), **L-1** (4.4 mg, 0.016 mmol, 10 mol%), CuI (1.5 mg, 0.008 mmol, 5 mol%), and K_2_CO_3_ (44 mg, 0.32 mmol, 2 equiv.); **30z** (42 mg, 0.14 mmol, 87%); ^1^H NMR (DMSO-*d*_6_, 500 MHz) δ 9.60 (s, 1H), 8.47 (d, *J* = 7.5 Hz, 1H), 7.91 (d, *J* = 7.0 Hz, 2H), 7.59 (s, 1H), 7.36 (d, *J* = 8.9 Hz, 2H), 6.33 (d, *J* = 7.6 Hz, 1H), 3.70 (bs, 1H), 3.54 (m, 1H), 1.13 (d, *J* = 6.3 Hz, 6H); ^13^C NMR (DMSO-*d*_6_, 125 MHz) δ 150.3, 140.1, 136.5, 136.23, 136.18, 128.9, 125.3, 120.4, 118.5, 100.7, 47.8, 23.7; HRMS Calcd. for C_15_H_17_ClN_5_ [M + H]: 302.1172; Found: 302.1179 (∆ = 2.3 ppm).

***5-(4-(Chloro)anilin-1-yl)-3-(morpholin-1-yl)pyrazolo[1,5-a]pyrimidine (30a’)***

3-Bromo-5-(4-(isopropyl)anilin-1-yl)pyrazolo[1,5-*a*]pyrimidine (50 mg, 0.16 mmol), morpholine (21 mg, 0.24 mmol, 1.5 equiv.), **L-1** (4.4 mg, 0.016 mmol, 10 mol%), CuI (1.5 mg, 0.008 mmol, 5 mol%), and K_2_CO_3_ (44 mg, 0.32 mmol, 2 equiv.); **30a’** (48 mg, 0.146 mmol, 91%); ^1^H NMR (DMSO-*d*_6_, 500 MHz) δ 9.75 (s, 1H), 8.51 (d, *J* = 7.6 Hz, 1H), 7.80 (d, *J* = 8.9 Hz, 2H), 7.69 (s, 1H), 7.37 (d, *J* = 8.7 Hz, 2H), 6.40 (d, *J* = 7.6 Hz, 1H), 3.77 (t, *J* = 4.5 Hz, 4H), 3.12 (t, *J* = 4.4 Hz, 4H); ^13^C NMR (DMSO-*d*_6_, 125 MHz) δ 150.4, 139.7, 136.9, 136.4, 134.4, 129.0, 125.7, 122.4, 120.6, 101.2, 66.5, 51.6; HRMS Calcd. for C_16_H_17_ClN_5_O [M + H]: 130.1122; Found: 330.1118 (∆ = 1.2 ppm).

***5-(4-(Chloro)anilin-1-yl)-3-(anilin-1-yl)pyrazolo[1,5-a]pyrimidine (30b’)***

3-Bromo-5-(4-(isopropyl)anilin-1-yl)pyrazolo[1,5-*a*] (50 mg, 0.16 mmol), aniline (22 mg, 0.24 mmol, 1.5 equiv.), **L-1** (4.4 mg, 0.016 mmol, 10 mol%), CuI (1.5 mg, 0.008 mmol, 5 mol%), and K_2_CO_3_ (44 mg, 0.32 mmol, 2 equiv.); **30b’** (39 mg, 0.12 mmol, 75%); ^1^H NMR (DMSO-*d*_6_, 500 MHz) δ 9.77 (s, 1H), 8.63 (d, *J* = 7.5 Hz, 1H), 7.92 (s, 1H), 7.75 (d, *J* = 7.0 Hz, 2H), 7.29 (s, 1H), 7.16 (d, *J* = 7.0 Hz, 2H), 7.11 (dd, *J* = 8.3, 7.4 Hz, 2H), 6.72 (d, *J* = 7.7 Hz, 2H), 6.63 (t, *J* = 7.3 Hz, 1H), 6.46 (d, *J* = 7.6 Hz, 1H); ^13^C NMR (DMSO-*d*_6_, 125 MHz) δ 151.6, 148.1, 141.5, 140.8, 139.6, 136.7, 129.2, 128.8, 125.6, 120.7, 117.1, 113.8, 110.6, 101.3; HRMS Calcd. for C_18_H_15_ClN_5_ [M + H]: 336.1016; Found: 336.1009 (∆ = 2.1 ppm).

***5-(4-(Chloro)anilin-1-yl)-3-(N-(pyrimidin-2-yl)amino)pyrazolo[1,5-a]pyrimidine (30c’)***

3-Bromo-5-(4-(isopropyl)anilin-1-yl)pyrazolo[1,5-*a*]pyrimidine (50 mg, 0.16 mmol), 2-aminopyrimidine (23 mg, 0.24 mmol, 1.5 equiv.), **L-1** (4.4 mg, 0.016 mmol, 10 mol%), CuI (1.5 mg, 0.008 mmol, 5 mol%), and K_2_CO_3_ (44 mg, 0.32 mmol, 2 equiv.); **30c’** (40 mg, 0.12 mmol, 75%); ^1^H NMR (DMSO-*d*_6_, 500 MHz) δ 9.77 (s, 1H), 8.76 (s, 1H), 8.62 (d, *J* = 7.5 Hz, 1H), 8.37 (d, *J* = 4.7 Hz, 2H), 8.12 (s, 1H), 7.86 (d, *J* = 8.9 Hz, 2H), 7.26 (d, *J* = 8.9 Hz, 2H), 6.73 (t, *J* = 4.7 Hz, 1H), 6.46 (d, *J* = 7.6 Hz, 1H); ^13^C NMR (DMSO-*d*_6_, 125 MHz) δ 161.9, 158.5, 151.7, 140.8, 139.9, 139.7, 136.4, 128.8, 125.6, 120.8, 111.6, 108.4, 101.4; HRMS Calcd. for C_16_H_13_ClN_7_ [M + H]: 338.0921; Found: 338.0912 (∆ = 2.7 ppm).

***5-(Pyrrolidin-1-yl)pyrazolo[1,5-a]pyrimidine (31)***

Similar to the method for preparing **29a–e**. 5-chloropyrazolo[1,5-*a*]pyrimidine (1.0 g, 6.5 mmol), pyrrolidine (925 mg, 13 mmol, 2.0 equiv), CsF (988 mg, 6.5 mmol, 1.0 equiv), and BnNEt_3_Cl (148 mg, 0.65 mmol,10 mol%), in DMSO (30 mL) was stirred in a sealed pressure vessel (350 mL) and heated at 100 °C for 8 hrs. After cooling to ambient temperature, H_2_O (300 mL) was added. The mixture was diluted using ethyl acetate, and the organic layer was separated. The aqueous layer was partitioned with EtOAc (2 × 300 mL), and the combined organic layers were washed with saturated sodium bicarbonate solution (2 × 300 mL) and then water (2 × 300 mL). The organic layer was dried over anhydrous magnesium sulfate, filtered, and then evaporated under reduced pressure. The crude product was purified using flash chromatography (EtOAc/Hexanes) to give pure **31** (1.13 g, 6.0 mmol, 92%); ^1^H NMR (DMSO-*d*_6_, 500 MHz) δ 8.59 (dd, *J* = 7.7, 0.5 Hz, 1H), 7.81 (d, *J* = 2.1 Hz, 1H), 6.36 (d, *J* = 7.7 Hz, 1H), 5.98 (d, *J* = 1.5 Hz, 1H), 3.48 (t, *J* = 6.5 Hz, 4H), 1.95 (“bs”, 4H); ^13^C (DMSO, 125 MHz) δ 154.0, 148.8, 144.5, 135.6, 98.3, 91.1, 47.1, 25.4; HRMS Calcd. for C_10_H_13_N_4_ [M + H]: 189.1140; Found: 189.1132 (∆ = 4.2 ppm).

### 4.5. 3-Halo-5-(Pyrrolidin-1-yl)Pyrazolo[1,5-a]Pyrimidines 32

General Procedure: To a stirred solution of 5-(pyrrolidin-1-yl)pyrazolo[1,5-*a*]pyrimidine **(31)** (100 mg) in CH_3_CN:CH_2_Cl_2_ (1:3, 2 mL) chilled to 0 °C was added N-halosuccinimide (1.0 equiv in 0.5 mL CH_3_CN), over 30 min. The mixture was stirred while slowly warming to ambient temperature (approx. 2 h). The solvents were removed under reduced pressure, and the crude residue was purified by flash chromatography (20% EtOAc/CH_2_Cl_2_).

***3-Chloro-5-(pyrrolidin-1-yl)pyrazolo[1,5-a]pyrimidine (32a)***

5-(Pyrrolidin-1-yl)pyrazolo[1,5-*a*]pyrimidine (100 mg, 0.53 mmol) in 2 mL (CH_3_CN:CH_2_Cl_2_, 1:3), N-chlorosuccinimide (70 mg, 0.53 mmol) in 0.5 mL CH_3_CN; **32a** (110 mg, 0.49 mmol, 93%); ^1^H NMR (DMSO-*d*_6_, 500 MHz) δ 8.59 (d, *J* = 7.8 Hz, 1H), 7.93 (s, 1H), 6.44 (d, *J* = 7.7 Hz, 1H), 3.52 (“bs”, 4H), 1.96 (“bs”, 4H); ^13^C NMR (DMSO-*d*_6_, 125 MHz) δ 154.3, 144.2, 142.2, 136.2, 99.2, 92.1, 47.2, 25.7, 24.9; HRMS Calcd. for C_10_H_12_ClN_4_ [M + H]: 223.0750; Found: 223.0761 (∆ = 4.9 ppm).

***3-Iodo-5-(pyrrolidin-1-yl)pyrazolo[1,5-a]pyrimidine (32b)***

5-(Pyrrolidin-1-yl)pyrazolo[1,5-*a*]pyrimidine (100 mg, 0.53 mmol) in 2 mL (CH_3_CN:CH_2_Cl_2_, 1:3); N-iodosuccinimide (119 mg, 0.53 mmol) in 0.5 mL CH_3_CN; **32b** (160 mg, 0.51 mmol; 96%); ^1^H NMR (DMSO-*d*_6_, 500 MHz) δ 8.60 (d, *J* = 7.8 Hz, 1H), 7.87 (s, 1H), 6.40 (d, *J* = 7.7 Hz, 1H), 3.52 (“bs”, 4H), 1.96 (“bs”, 4H); ^13^C NMR (DMSO-*d*_6_, 125 MHz) δ 154.7, 148.2, 148.0, 136.2, 99.1, 47.2, 43.3, 25.7, 24.9; HRMS Calcd. for C_10_H_12_IN_4_ [M + H]: 315.0107; Found: 315.0096 (∆ = 3.5 ppm).

## 5. Conclusions

We have developed an efficient method for rapid generation of diverse arrays of 3,5-bis-aminated pyrazolo[1,5-*a*]pyrimidine derivatives. The reaction proceeds in only two steps from commercially available 3-bromo-5-chloropyrazolo[1,5-*a*]pyrimidine and features efficient copper catalyzed C-3 amination of C-5 aminated precursors. 3,5-bis-aminated products consisting of both 3,5-bis arylamine, 3,5-bis alkylamine, or 3,5-bis alkyl/aryl amine combinations were efficiently obtained in only two steps utilizing our method. This represents the most comprehensive substrate scope for 3,5-bis-aminated pyrazolo[1,5-*a*]pyrimidines ever reported. Advantages of this method include rapid reaction time (1 h for C-3 amination), high yield, avoidance of toxic reagents such as NaBH_3_CN commonly used for reductive amination, and utilization of inexpensive CuI catalyst (5 mol%) in place of more expensive and/or less efficient air and moisture-sensitive palladium catalysts previously utilized for C-3 amination (Figure 2). With these advantages now in hand, access to broad, diversity-rich libraries of 3,5-bis-aminated pyrazolo[1,5-*a*]pyrimidines for screening novel or enhanced biological, medicinal, or fluorometric properties is now readily available.

## Figures and Tables

**Table 1:** Optimization. ^a–e^

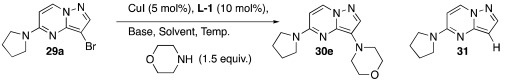						
Entry	Temp. (°C)	Ligand	Time	Solvent	Base ^c^	Products ^d^
1	RT	**L-1**	7 days	DEG	K_2_CO_3_	**30e** (---) **31** (---) ^e^ **29a** (100%)
2	50 ^a^	**L-1**	2 days	DEG	K_2_CO_3_	**30e** (7.7%) **31** (---) ^e^ **29a** (92.3%)
3	70 ^a^	**L-1**	24 h	DEG	K_2_CO_3_	**30e** (27.3%) **31** (2.8%); **29a** (69.9%)
4	80 ^a^	**L-1**	2 days	DEG	K_2_CO_3_	**30e** (82.4%) **31** (17.6%); **29a** (---)
5	70 ^b^	**L-1**	30 min	DEG	K_2_CO_3_	**30e** (61.4%) **31** (1.8%); **29a** (36.8%)
6	70 ^b^	**L-1**	1 h	DEG	K_2_CO_3_	**30e** (78.7%) **31** (2.4%); **29a** (18.9%)
7	70 ^b^	**L-1**	1.5 h	DEG	K_2_CO_3_	**30e** (87.2%) **31** (2.1%); **29a** (10.7%)
8	70 ^b^	**L-1**	2 h	DEG	K_2_CO_3_	**30e** (96.7%) **31** (2.5%); **29a** (0.8%)
9	70 ^b^	**L-1**	3 h	DEG	K_2_CO_3_	**30e** (97.3%) **31** (2.7%); **29a** (---)
10	80 ^b^	**L-1**	30 min	DEG	K_2_CO_3_	**30e** (93.7%) **31** (1.9%); **29a** (4.4%)
11	80 ^b^	**L-1**	1 h	DEG	K_2_CO_3_	**30e** (97.1%) **31** (2.9%); **29a** (---)
12	80 ^b^	**L-1**	1.5 h	DEG	K_2_CO_3_	**30e** (97.0%) **31** (3.0%); **29a** (---)
13	80 ^b^	**L-1**	1 h	Ethylene Glycol	K_2_CO_3_	**30e** (90.1%) **31** (9.9%); **29a** (---)
14	80 ^b^	**L-1**	1 h	DMSO	K_2_CO_3_	**30e** (10.2%) **31** (5.1%); **29a** (84.7%)
15	80 ^b^	**L-1**	1 h	^n^ Butanol	K_2_CO_3_	**30e** (3.6%) **31** (6.3%); **29a** (90.1%)
16	80 ^b^	**L-1**	1 h	Dioxane	K_2_CO_3_	**30e** (---) **31** (7.2%); **29a** (92.8%)
17	80 ^b^	**L-1**	1 h	NMP	K_2_CO_3_	**30e** (78%) **31** (19%); **29a** (3%)
18	80 ^b^	**L-1**	1 h	1,2-Propanediol	K_2_CO_3_	**30e** (94.3%) **31** (5.7%); **29a** (---)
19	80 ^b^	**L-1**	1 h	DEG	tBuOK	**30e** (95.2%) **31** (4.8%); **29a** (---)
20	80 ^b^	**L-1**	1 h	DEG	TMSONa	**30e** (97.1%) **31** (2.9%); **29a** (---)
21	80 ^b^	**L-1**	1 h	DEG	K_3_PO_4_	**30e** (94.3%) **31** (5.7%); **29a** (---)
22	80 ^b^	**L-1**	1 h	DEG	DBU	**30e** (48.8%) **31** (1.4%); **29a** (49.8%)
23	80 ^b^	**L-2**	1 h	DEG	K_2_CO_3_	**30e** (21%) **31** (39%); **29a** (40%)
24	80 ^b^	**L-3**	1 h	DEG	K_2_CO_3_	**30e** (17.4%) **31** (0.7%); **29a** (81.9%)
25	80 ^b^	**L-4**	1 h	DEG	K_2_CO_3_	**30e** (12.3%) **31** (---); **29a** (87.7%)

^a^ Conventional oil bath heating; ^b^ Microwave heating; ^c^ 2.0 equiv.; ^d^ Percent conversion as determined by ^1^HNMR; ^e^ Trace amounts (^1^H NMR signal detected, but barely distinguishable from baseline noise).

## Data Availability

The original contributions presented in this study are included in the article/Supplementary Material. Further inquiries can be directed to the corresponding author.

## Supplementary Materials

The following supporting information can be downloaded at: https://www.mdpi.com/article/10.3390/molecules30030458/s1, Experimental procedures for preparing **L**-**1**–**L**-**4**; Scheme S1: Synthesis of ligands **L-1**–**L-4**; spectral data for compounds **29a–e**, **30a–c’**, **31**, **32a–b**; and representative conversion-optimization data. References [30,32,33,34] are cited the supplementary materials.

## References

[1] Shen J., Deng X., Sun R., Tavallaie M.S., Wang J., Cai Q., Lam C., Lei S., Fu L., Jiang F. (2020). Structural optimization of pyrazolo [1,5-*a*]pyrimidine derivatives as potent and highly selective DPP-4 inhibitors. Eur. J. Med. Chem..

[2] Arias-Gomez A., Godoy A., Portilla J. (2021). Functional pyrazolo [1,5-a]pyrimidines: Current approaches in synthetic transformations and uses as an antitumor scaffold. Molecules.

[3] Jismy B., Guillaumet G., Akssira M., Tikad A., Abarbri M. (2021). Efficient microwave-assisted Suzuki–Miyaura cross-coupling reaction of 3-bromo pyrazolo [1,5-*a*]pyrimidin-5(4H)-one: Towards a new access to 3,5-diarylated 7-(trifluoromethyl)pyrazolo [1,5-*a*]pyrimidine derivatives. RSC Adv..

[4] Yamaguchi-Sasaki T., Tokura S., Ogata Y., Kawaguchi T., Sugaya Y., Takahashi R., Iwakiri K., Abe-Kumasaka T., Yoshida I., Arikawa K. (2020). Discovery of a potent dual inhibitor of wild-type and mutant respiratory syncytial virus fusion proteins. ACS Med. Chem. Lett..

[5] Fouda A.M., Abbas H.-A., Ahmed E.H., Shati A.A., Alfaifi M.Y., Elbehairi S.E.I. (2019). Synthesis, in vitro antimicrobial and cytotoxic activities of some new Pyrazolo [1,5-*a*]pyrimidine derivatives. Molecules.

[6] Hassan A.S., Masoud D.M., Sroor F.M., Askar A.A. (2017). Synthesis and biological evaluation of pyrazolo [1,5-*a*]pyrimidine-3-carboxamide as antimicrobial agents. Med. Chem. Res..

[7] Al-Adiwish W.M., Tahir M.I.M., Siti-Noor-Adnalizawati A., Hashim S.F., Ibrahim N., Yaacob W.A. (2013). Synthesis, antibacterial activity and cytotoxicity of new fused pyrazolo [1,5-*a*]pyrimidine and pyrazolo [5,1-*c*][1,2,4]triazine derivatives from new 5-aminopyrazoles. Eur. J. Med. Chem..

[8] Novinson T., Bhooshan B., Okabe T., Revankar G.R., Robins R.K., Senga K., Wilson H.R. (1976). Novel heterocyclic nitrofurfural hydrazones. In vivo antitrypanosomal activity. J. Med. Chem..

[9] Tsai P.C., Wang I.J. (2005). Synthesis and solvatochromic properties of some disazo dyes derived from pyrazolo [1,5-*a*]pyrimidine derivatives. Dye. Pigm..

[10] Tigreros A., Aranzazu S.-L., Bravo N.F., Zapata-Rivera J., Portilla J. (2020). Pyrazolo [1,5-*a*]pyrimidines-based fluorophores: A comprehensive theoretical-experimental study. RSC Adv..

[11] Tigreros A., Rosero H.A., Castillo J.-C., Portilla J. (2019). Integrated pyrazolo [1,5-*a*]pyrimidine-hemicyanine system as a colorimetric and fluorometric chemosensor for cyanide recognition in water. Talanta.

[12] Ding R., He Y., Xu J., Liu H., Wang X., Feng M., Qi C., Zhang J., Peng C. (2011). Preparation and bioevaluation of ^99m^Tc nitrido radiopharmaceuticals with pyrazolo [1,5-*a*]pyrimidine as tumor imaging agents. Med. Chem. Res..

[13] Zhang Y., Liu Y., Zhou Y., Zhang Q., Han T., Tang C., Fan W. (2021). Pyrazolo [1,5-*a*]pyrimidine based trk inhibitors: Design, synthesis, biological activity evaluation. Bioorg. Med. Chem. Lett..

[14] Kosugi T., Mitchell D.R., Fujino A., Imai M., Kambe M., Kobayashi S., Makino H., Matsueda Y., Oue Y., Komatsu K. (2012). Mitogen-activated protein kinase-activated protein kinase 2 (mapkap-k2) as an antiinflammatory target: Discovery and in vivo activity of selective pyrazolo [1,5-*a*]pyrimidine inhibitors using a focused library and structure-based optimization approach. J. Med. Chem..

[15] Loew G., Toll L., Lawson J., Uyeno E., Kaegi H. (1984). Pyrazolo [1,5-*a*]pyrimidines: Receptor binding and anxiolytic behavioral studies. Pharmacol. Biochem. Behav..

[16] Selleri S., Bruni F., Costagli C., Costanzo A., Guerrini G., Ciciani G., Gratteri P., Besnard F., Costa B., Montali M. (2005). A novel selective GABAAα1 receptor agonist displaying sedative and anxiolytic-like properties in rodents. J. Med. Chem..

[17] Farago A.F., Demetri G.D. (2020). Larotrectinib, a selective tropomyosin receptor kinase inhibitor for adult and pediatric tropomyosin receptor kinase fusion cancers. Future Oncol..

[18] Sava J. FDA Grants Breakthrough Therapy Designation to Repotrectinib for ROS1+ Metastatic NSCLC. https://www.targetedonc.com/view/fda-grants-breakthrough-therapy-designation-to-repotrectinib-for-ros1-metastatic-nsclc.

[19] A Study to Test the Safety of the Investigational Drug Selitrectinib in Children and Adults That May Treat Cancer. https://clinicaltrials.gov/ct2/show/NCT03215511.

[20] Novotna K., Thomas A.G., Stepanek O., Murphy B., Hin N., Skacel J., Mueller L., Tenora L., Pal A., Alt J. (2023). Neutral sphingomyelinase 2 inhibitors based on the pyrazolo [1,5-*a*]pyrimidin-3-amine scaffold. Eur. J. Med. Chem..

[21] Hu Y., Wu D., Peng W., Li X., Hu F., Huang B., Zhu J., Wu Y. (2019). Heterocyclic compound, application thereof and pharmaceutical composition comprising same. PCT Int. Appl..

[22] Pal K., Ciblat S., Albert V., Bruneau-Latour N., Boudreault J. (2019). Preparation of 5-(2-(2,5-difluorophenyl)pyrrolidin-1-yl)-3-(1H-pyrazol-1-yl)pyrazolo [1,5-*a*]pyrimidine derivatives and related compounds as Trk kinase inhibitors for treating cancer. PCT Int. Appl..

[23] Ellermann M., Valot G., Cancho Grande Y., Hassfeld J., Kinzel T., Koebberling J., Beyer K., Roehrig S., Sperzel M., Stampfuss J. (2016). Piperidinylpyrazolopyrimidinones as plasminogen inhibitors and their preparation. PCT Int. Appl..

[24] Magavi S.S., Parks D.J., Tait B.D., Cho J., Agrawal R., Shaw P.R. (2023). Preparation of substituted pyrazolopyridines as platelet-derived growth factor receptor (PDGFR) alpha inhibitors and uses thereof. PCT Int. Appl..

[25] Henning N.J., Nomura D.K., Boike L., Marquess D., Keitz P. (2024). Preparation of bifunctional compounds as protein-stabilizing deubiquitinase-targeting chimeras useful in treatment of cystic fibrosis and other diseases. PCT Int. Appl..

[26] Iorkula T.H., Tolman B.A., Singleton J.D., Peterson M.A. (2023). An efficient microwave assisted copper catalyzed C-3 amination of 3-bromopyrazolo [1,5-*a*]pyrimidine. Tetrahedron Lett..

[27] Reynolds M., Eary C.T., Spencer S., Juengst D., Hache B., Jiang Y., Haas J., Andrews S.W. (2022). Preparation of (S)-N-(5-((R)-2-(2,5-Difluorophenyl)pyrrolidin-1-yl)pyrazolo [1,5-a]pyrimidin-3-yl)-3-hydroxypyrrolidine-1-carboxamide. WO, 201241, 2017.

[28] Flick A.C., Leverett C.A., Ding H.X., McInturff E., Fink S.J., Helal C.J., DeForest J.C., Morse P.D., Mahapatra S., O’Donnell C.J. (2020). Synthetic approaches to new drugs approved during 2018. J. Med. Chem..

[29] Chi L.-L., Hao L.-L., Cai Z.-Q., Kong D.-L., Wang Y.-N., Qin W.-T., Gao Y., Qu Z.-Z. (2022). Design, synthesis, and biological evaluation of novel pyrazolo [1,5-*a*]pyrimidine and 1,3-Benzodiazine derivatives as potent antitumor agents. Russ. J. Gen. Chem..

[30] Hong P., Zhu X., Lai X., Gong Z., Huang M., Wan Y. (2024). Room-temperature CuI-catalyzed *N*-arylation of cyclopropylamine. J. Org. Chem..

[31] Hong P., Zhu X., Chen F., Huang M., Wan Y. (2024). CuSO_4_/*N*-(9*H*-carbazol-9-yl)picolinamide-catalyzed C-O coupling of (hetero)aryl chlorides with phenols on water. Org Lett..

[32] FrØyen P. (1995). Formation of acyl bromides from carboxylic acids and N-bromosuccinimide; some reactions of bromocyanotriphenylphosphorane. Phosphorus Sulfur Silicon Relat. Elem..

[33] Wangngae S., Duangkamol C., Pattarawarapana M., Phakhodee W. (2015). Significance of reagent addition sequence in the amidation of carboxylic acids mediated by PPh_3_ and I_2_. RSC Adv..

[34] Cui H., Wang L., Bai G., Li D., Lin P. (2006). Synthesis of N-Amino-Carbazole. Yingyong Huagong.

[35] Iorkula T.H., Tolman B.A., Burt S.R., Peterson M.A. (2024). An Efficient synthesis of C-6 aminated 3-bromoimidazo [1,2-*b*]pyridazines. Synth. Comm..

