# Supplementary material for: An Efficient Synthesis of 3,5-Bis-Aminated Pyrazolo[1,5-a]Pyrimidines: Microwave-Assisted Copper Catalyzed C-3 Amination of 5-Amino-3-Bromo-Substituted Precursors
Source: Molecules. 2025 Jan 21;30(3):458. doi: 10.3390/molecules30030458 (PMC11820075; doi:10.3390/molecules30030458)
Supplement: Supplementary file 1 [file molecules-30-00458-s001.zip › molecules-3347510-supplementary.pdf]

An Efficient Synthesis of 3,5-Bis-Aminated Pyrazolo[1,5-*a*]pyrimidines: Microwave Assisted Copper Catalyzed C-3 Amination of 5-Amino-3-Bromo-substituted Precursors

Terungwa H. Iorkula,[a] Bryce A. Tolman, Latifat Ganiyu, and Matt A. Peterson[a,] *

[a]*Department of Chemistry and Biochemistry, Brigham Young University, Provo, UT 84602*
* Corresponding author. Tel.: +0-801-422-6843; fax: +0-801-422-0153; email:mapeterson@chem.byu.edu

SUPPLEMENTARY INFORMATION

General Experimental... 2

Ligand Synthesis 2

Optimization of C-3 Amination... 5

NMR Spectra (^{1}H, ^{13}C)

N-Nitroso-9H-carbazole... 7
N-Amino-9H-carbazole... 11
L-1... 15
L-2... 20
L-3... 24
L-4... 28
29a... 32
29b... 34
29c... 36
29d... 39
29e... 43
30a... 46
30b... 50
30c... 54
30d... 58
30e... 62
30f... 66
30g... 70
30h... 72
30i... 76
30j... 80
30k... 84
30l... 88
30m... 91
30n... 94
30o... 99
30p... 103
30q... 107
30r... 111
30s... 115
30t... 119

30u.. 122
30v.. 125
30w..129
30x.. 135
30y.. 139
30z.. 143
30a'.. 147
30b'..151
30c'.. 154
31.. 157
32a.. 160
32b.. 162
Representative Optimization Spectra... 164

General Experimental

Reactions described in the Supplementary Materials section were performed in flame- or oven-dried round-bottom flasks using solvents directly as supplied by commercial vendors, or dried as needed over standard drying agents, depending on the moisture sensitivity of the particular reaction. All other reagents were used directly as supplied without additional drying or purification. ^{1}H NMR and ^{13}C NMR spectra were determined in $CDCl_3$ or DMSO-d_6 using internal references: δ 7.27 or 2.50 (^{1}H), δ 77.23 or 39.52 (^{13}C), respectively. High resolution mass spectra were obtained using fast atom bombardment electrospray (ES) ionization techniques and had errors of less than ± 5 ppm.

Ligand Synthesis

Ligands **L-1**, **L-2**, **L-3**, and **L-4** were prepared via a modification of the method of Huang and co-workers, where we employed Ph_3P/NIS to generate a reactive acyl iodide intermediate[1, 2] to achieve the desired amidation (Scheme S1). In our hands, the method of Huang and co-workers which employed $SOCl_2$ for generating an acyl chloride consistently gave yields <35%, even after rigorous drying of glassware, solvents, and reagents. The Ph_3P/NIS method was more reproducible, and ligands were obtained in 38-67% (ave = 49%).

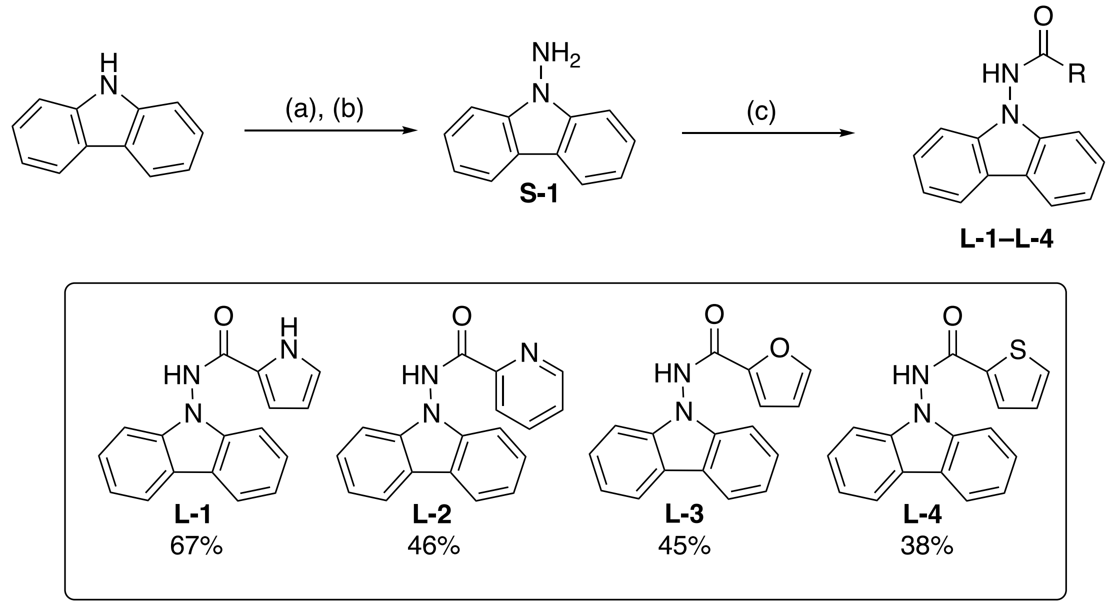

Scheme S1. Reagents and conditions: (a) $NaNO_2$, AcOH:DMSO (1:1); 0°C–RT; (b) Zn, EtOH:AcOH (4:1), 0°C–RT, 5 h; (c) RCO_2H (1.3 equiv.), Ph_3P (1.5 equiv.), NIS (1.5 equiv.), CH_3CN, 0°C – RT, 16 h.

N-Nitroso-9H-carbazole

N-Nitroso-9H-carbazole was prepared via the method of Huang and co-workers (1 g, 97%).[3] An exhaustive search of CAS SciFinder (Oct. 2024) revealed that although the ^1H characterization data for this compound has been reported, ^{13}C NMR data was missing. We report such data for archival purposes.

^1H NMR ($CDCl_3$, 500 MHz) δ 8.59–8.58 (m, 1H), 8.26 (d, J = 8.0 Hz; 1H), 7.97–7.93 (m, 2H), 7.57 (dt, J = 8.3, 1.0 Hz; 1H), 7.53–7.48 (m, 3H), ^{13}C NMR ($CDCl_3$, 125 MHz) δ 138.5, 132.7, 128.5, 128.1, 127.4, 126.2, 125.3, 124.8, 120.3, 119.8, 116.5, 112.3; HRMS Calcd. for $C_{12}H_9N_2O$ [M+H]: 197.0715; Found: 197.0721 (Δ = 3.0 ppm).

N-Amino-9H-carbazole **(S-1)**

N-Amino 9H-carbazole (**S-1**) was prepared following a modification of the method of Cui et al.[4] To a stirred solution of N-nitrosocarbazole (500 mg, 2.55 mmol), in EtOH:AcOH (4:1)(15 mL) at 0 °C was added Zn dust (670 mg, 10.5 mmol, 4 equiv.) in four aliquots over 1 hour (one aliquot every 15 min). The resulting mixture was allowed to warm to RT and stirred for 5 h. The crude reaction mixture was poured into EtOAc (20 mL), partitioned with H_2O (20 mL), and washed with $NaHCO_3$ (sat'd) (2 X 40 mL). The organic layer was dried over $MgSO_4$, filtered,

and then evaporated under reduced pressure. Purification via column chromatography (10% EtOAc/Hexanes) gave **S-1** (0.399 g, 2.2 mmol, 86%).

^{1}H NMR (DMSO-*d*$_6$, 500 MHz) δ 8.11 (d, *J* = 8.0 Hz, 2H), 7.60 (d, *J* = 8.5 Hz, 2H), 7.46 (dt, *J* = 8.0, 1.0 Hz, 2H), 7.17 ('t', *J* = 7.8 Hz, 2H), 5.83 (s, 2H); ^{13}C NMR (DMSO-*d*$_6$, 125 MHz) δ 141.6, 125.9, 120.4, 120.2, 119.0, 109.5; HRMS Calcd. for $C_{12}H_{10}N_2$ [M+H]: 183.0922; Found: 183.0929 (Δ = 3.8 ppm).

N-(9H-Carbazol-9-yl)-1H-pyrrole-2-carboxamide (L-1)

A solution of 1*H*-pyrrole-2-carboxylic acid (79 mg, 0.71 mmol, 1.3 equiv), N-iodosuccinimide (187 mg, 0.83 mmol, 1.5 equiv), and Ph_3P (218 mg, 0.83 mmol, 1.5 equiv), in CH_3CN (3 mL), was stirred under Argon at ambient temperature for 30 minutes. N-amino-9H-carbazole (100 mg, 0.55 mmol) was added, and the mixture stirred for an additional 16 h. Solvents were removed under reduced pressure and the crude residue purified via chromatography (20% EtOAc/Hexanes) to give **L-1** (101 mg, 0.37 mmol, 67%).

^{1}H NMR (DMSO-*d*$_6$, 500 MHz) δ 11.84 (s, 1H), 11.36 (s, 1H), 8.21 (d, *J* = 7.7 Hz, 2H), 7.46 (dt, *J* = 8.1, 1.0 Hz, 2H), 7.36 (d, *J* = 8.1 Hz, 2H), 7.28 ('t', *J* = 7.8 Hz, 2H), 7.20 (bs, 1H), 7.07 (bs, 1H), 6.27 (bs, 1H); ^{13}C NMR (DMSO-*d*$_6$, 125 MHz) δ 160.8, 140.7, 126.7, 123.8, 123.7, 121.1, 120.9, 120.4, 112.5, 109.8, 109.1; HRMS Calcd. for $C_{17}H_{14}N_3O$ [M+H]: 276.1137; Found: 276.1130 (Δ = 2.5 ppm).

N-(9H-Carbazol-9-yl)picolinamide (L-2)

Similar to **L-1**; N-aminocarbazole (100 mg, 0.55 mmol), picolinic acid (87 mg, 0.71 mmol, 1.3 equiv), N-iodosuccinimide (187 mg, 0.83 mmol, 1.5 equiv), Ph_3P (218 mg, 0.83 mmol, 1.5 equiv); **L-2** (73 mg, 0.25 mmol, 46%).

^{1}H NMR (DMSO-*d*$_6$, 500 MHz) δ 12.14 (s, 1H), 8.83 (d, *J* = 4.7 Hz, 1H), 8.20 (d, *J* = 7.7 Hz, 2H), 8.12–8.10 (m, 2H), 7.78–7.75 (m, 1H), 7.44 (t, *J* = 7.2 Hz, 2H), 7.33 (d, *J* = 8.0 Hz, 2H), 7.26 ('t', *J* = 7.5 Hz, 2H); ^{13}C NMR (DMSO-*d*$_6$, 125 MHz) δ 163.4, 148.4, 148.1, 139.2, 137.5, 127.0, 125.5, 122.3, 120.1, 119.8, 119.3, 108.1; HRMS Calcd. for $C_{18}H_{14}N_3O$ [M+H]: 288.1137; Found: 288.1134 (Δ = 1.0 ppm).

N-(9H-Carbazol-9-yl)furan-2-carboxamide (L-3)

Similar to **L-1**; N-aminocarbazole (100 mg, 0.55 mmol), furan-2-carboxylic acid (79 mg, 0.71 mmol, 1.3 equiv), N-iodosuccinimide (187 mg, 0.83 mmol, 1.5 equiv), Ph_3P (218 mg, 0.83 mmol, 1.5 equiv); **L-3** (68 mg, 0.25 mmol, 45%)

^{1}H NMR (DMSO-d_6, 500 MHz) δ 11.8 (bs, 1H), 8.20 (d, J = 7.8 Hz, 2H), 8.06 (d, J = 0.9 Hz, 2H), 7.47 - 7.44 (m, 3H), 7.37 (d, J = 8.1 Hz, 2H), 7.28 (ddd, J = 7.8, 7.1, 0.6 Hz, 2H), 6.80 (dd, J = 3.4, 1.7 Hz, 1H); ^{13}C NMR (DMSO-d_6, 125 MHz) δ 156.8, 145.9, 144.9, 139.3, 125.6, 120.1, 119.8, 119.5, 115.4, 111.7, 108.1; HRMS Calcd. for $C_{17}H_{13}N_2O_2$ [M+H]: 277.0977; Found: 277.0989 (Δ = 4.3 ppm).

N-(9H-Carbazol-9-yl)thiophene-2-carboxamide (L-4)

Similar to **L-1**; N-aminocarbazole (100 mg, 0.55 mmol), thiophene-2-carboxylic acid (91 mg, 0.71 mmol, 1.3 equiv), N-iodosuccinimide (187 mg, 0.83 mmol, 1.5 equiv), Ph_3P (218 mg, 0.83 mmol, 1.5 equiv); **L-4** (61 mg, 0.21 mmol, 38%).

^{1}H NMR (DMSO-d_6, 500 MHz) δ 11.9 (bs, 1H), 8.20 (d, J = 7.6 Hz, 2H), 8.14 (‘s’, 1H), 8.01 (d, J = 4.5 Hz, 1H), 7.47 (‘t’, J = 7.5 Hz, 2H), 7.39 (d, J = 8.0 Hz, 2H), 7.33 (d, J = 4.6 Hz, 1H), 7.28 (‘t’, J = 7.4 Hz, 1H); ^{13}C NMR (DMSO-d_6, 125 MHz) δ 160.4, 139.3, 135.4, 132.1, 129.5, 127.9, 125.7, 120.1, 119.9, 119.5, 108.1; HRMS Calcd. for $C_{17}H_{13}N_2OS$ [M+H]: 293.0749; Found: 293.0755 (Δ = 2.0 ppm).

Optimization of C-3 Amination

Reactions were set up according to the manuscript General Procedure for 3,5-bis-aminopyrazolo[1,5-*a*]pyrimidines **30**, reproduced below for clarity. After reacting at the specified temperatures, at timed intervals (as indicated in Table 1, see manuscript), the reactions were cooled to RT and analyzed by ^{1}H NMR. Various bases, solvents, and ligands were also evaluated (Table 1, manuscript).

General Procedure:

A solution of 3-Bromo-5-(pyrrolidin-1-yl)pyrazolo[1,5-*a*]pyrimidine (**29a**) (50 mg, 0.19 mmol), morpholine (25 mg, 0.29 mmol, 1.5 equiv.), **L-1** (6 mg, 0.02 mmol, 10 mol%), CuI (2 mg, 0.01 mmol, 5 mol%), and K_2CO_3 (52 mg, 0.38 mmol, 2 equiv.), in diethyleneglycol (DEG, 1 mL) was

stirred in a 10 mL microwave reactor vessel and heated at various temperatures and for varying lengths of time. Solvents, bases, and ligands were also varied (see Table 1, manuscript).

After the reaction had cooled to RT, an aliquot of reaction mix (approx. 200 µL) was directly mixed with DMSO-d_6 to prepare an NMR sample consisting of DEG:DMSO-d_6 (approx. 1:10, DEG:DMSO-d_6). Resonances Ha were normalized, then comparative integrations were used to determine the relative ratios of **29a**, **30e**, and **31** (see pp. 164-169).

---

[1] FrØyen, P. Formation of acyl bromides from carboxylic acids and N-bromosuccinimide; some reactions of bromocyanotriphenylphosphorane. *Phosphorus, Sulfur, and Silicon and the Related Elements*, **1995**, *102*, 253–259. https://doi.org/10.1080/10426509508042564

[2] Wangngae,S; Duangkamol, C.; Pattarawarapana, M.; Phakhodee; W. Significance of reagent addition sequence in the amidation of carboxylic acids mediated by PPh_3 and I_2. *RSC Adv.,* **2015**, *5*, 25789-25793 https://doi.org/10.1039/C5RA03184B

[3] Hong, P.; Zhu, X.; Lai, X.; Gong, Z.; Huang, M.; Wan, Y. Room-Temperature CuI-Catalyzed *N*-Arylation of Cyclopropylamine. *J. Org. Chem.* **2024**, *89*, 57–67. https://doi.org/10.1021/acs.joc.3c01357.

[4] Cui, H. et al. Synthesis of N-Amino-Carbazole, *Yingyong Huagong* **2006**, *35*, 295-297.

JI-II-43 10 1 /home/nmruser/networkdata/Bruker500Data/terungwa@chem.byu.edu

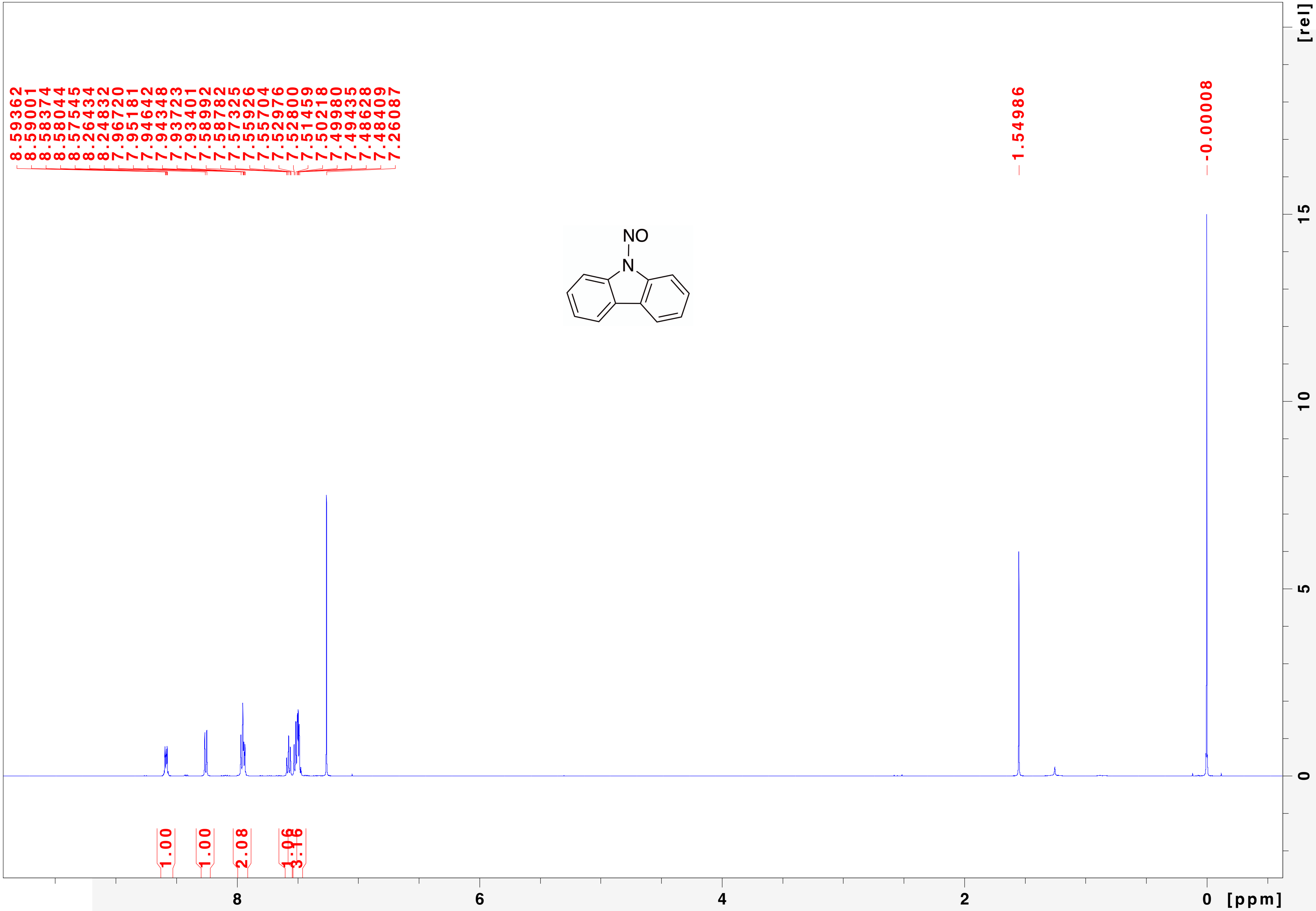

JI-II-43 10 1 /home/nmruser/networkdata/Bruker500Data/terungwa@chem.byu.edu

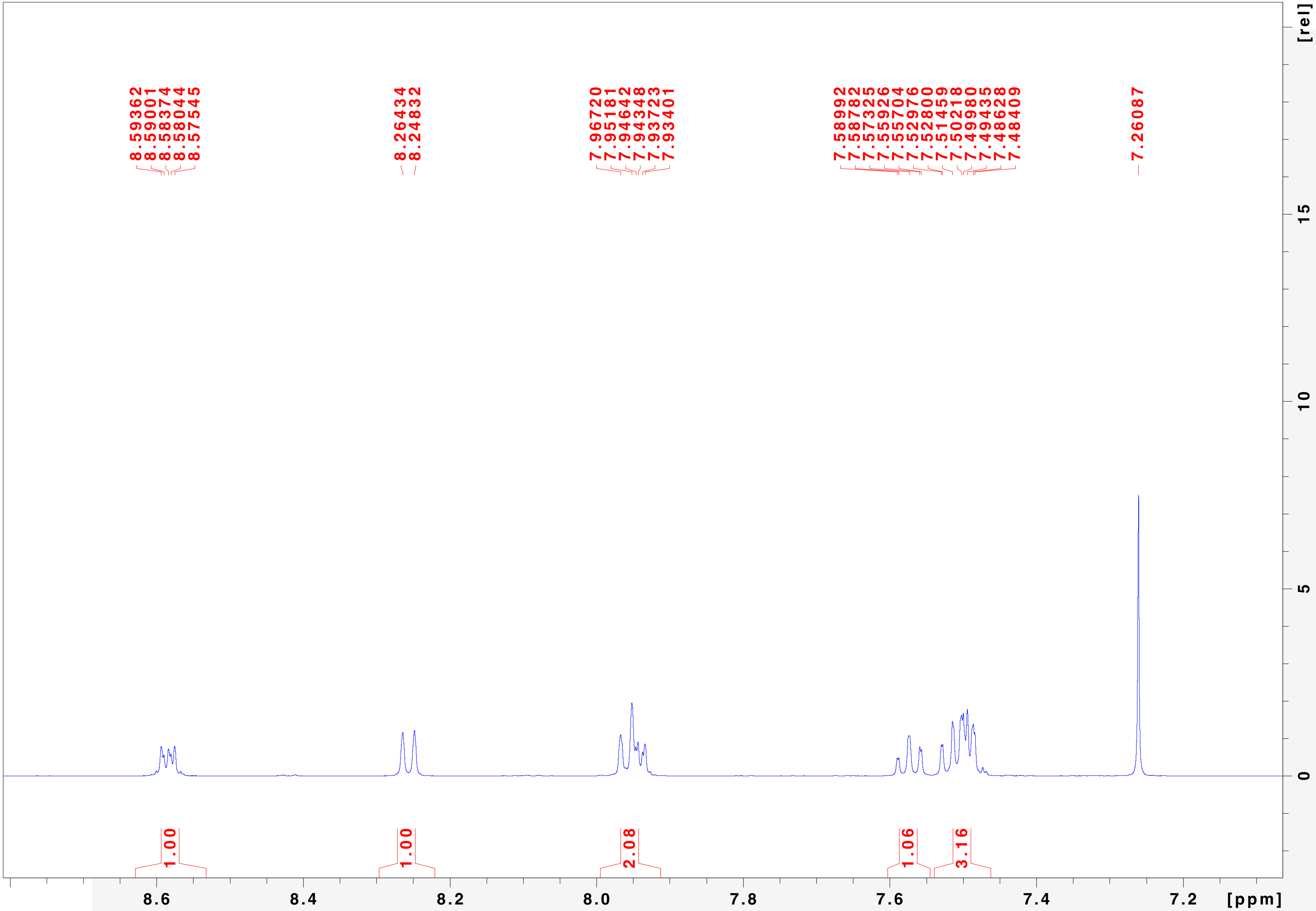

JI-II-43 20 1 /home/nmruser/networkdata/Bruker500Data/terungwa@chem.byu.edu

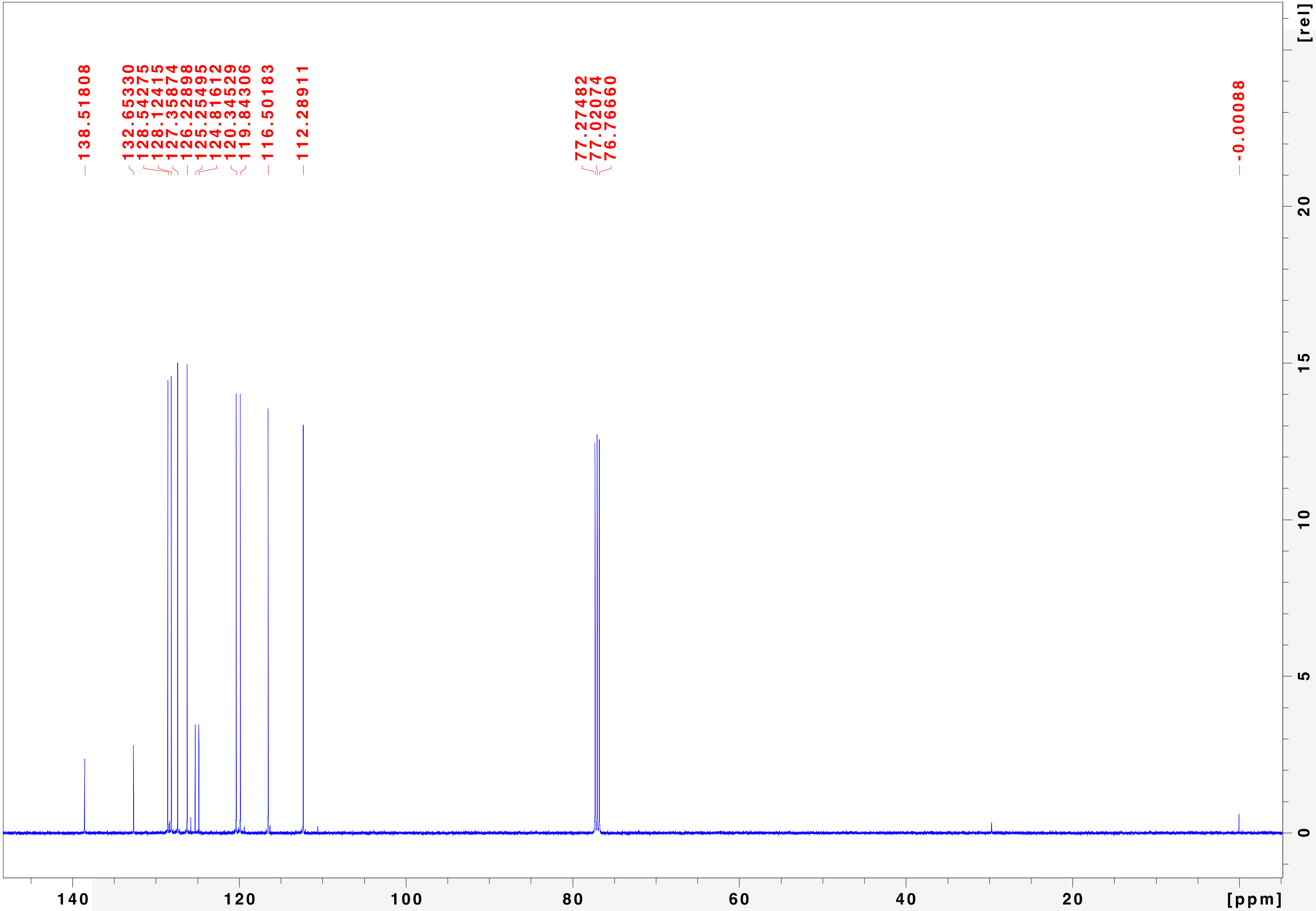

JI-II-43 20 1 /home/nmruser/networkdata/Bruker500Data/terungwa@chem.byu.edu

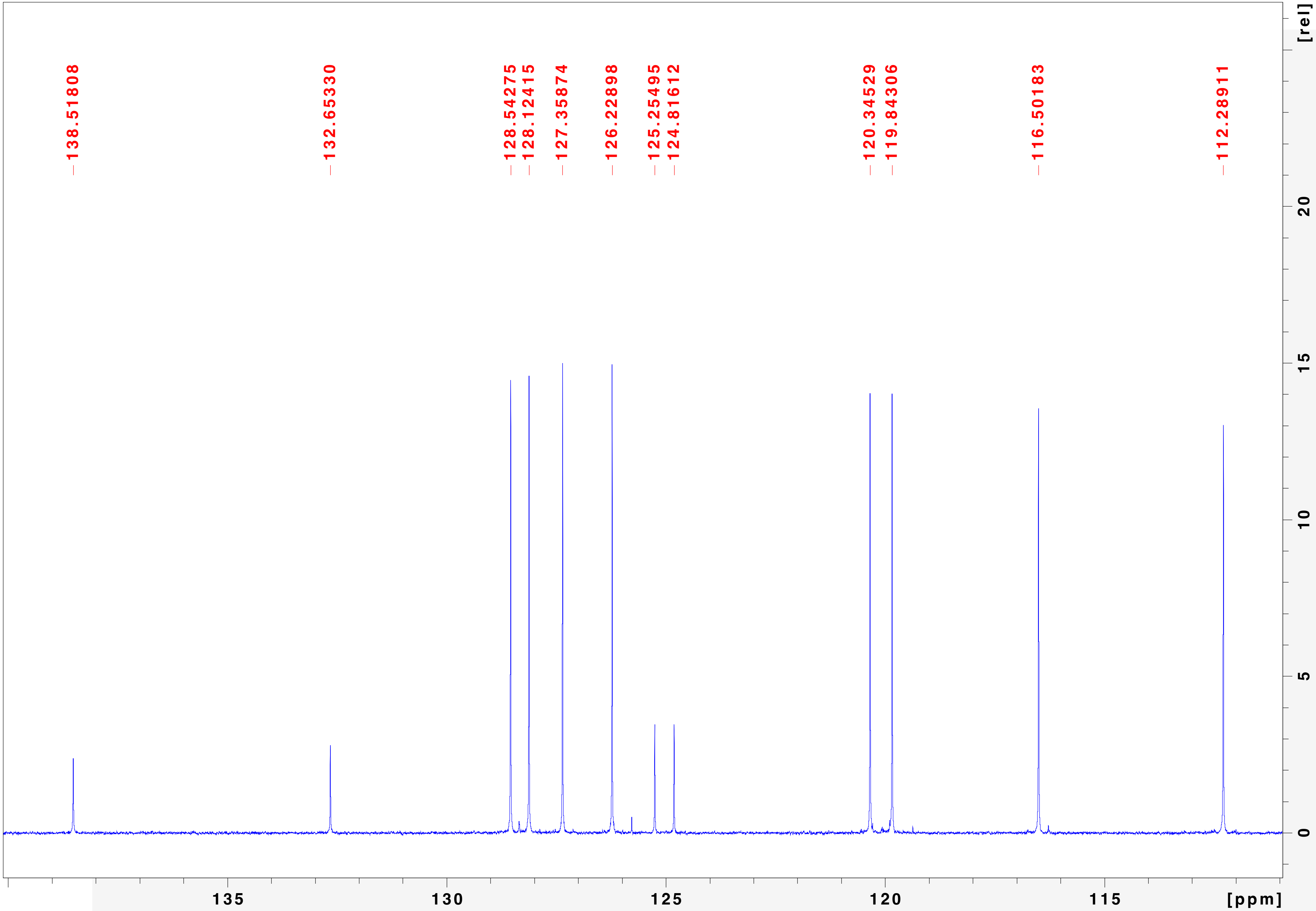

JI-II-45 20 1 /home/nmruser/networkdata/Bruker500Data/terungwa@chem.byu.edu

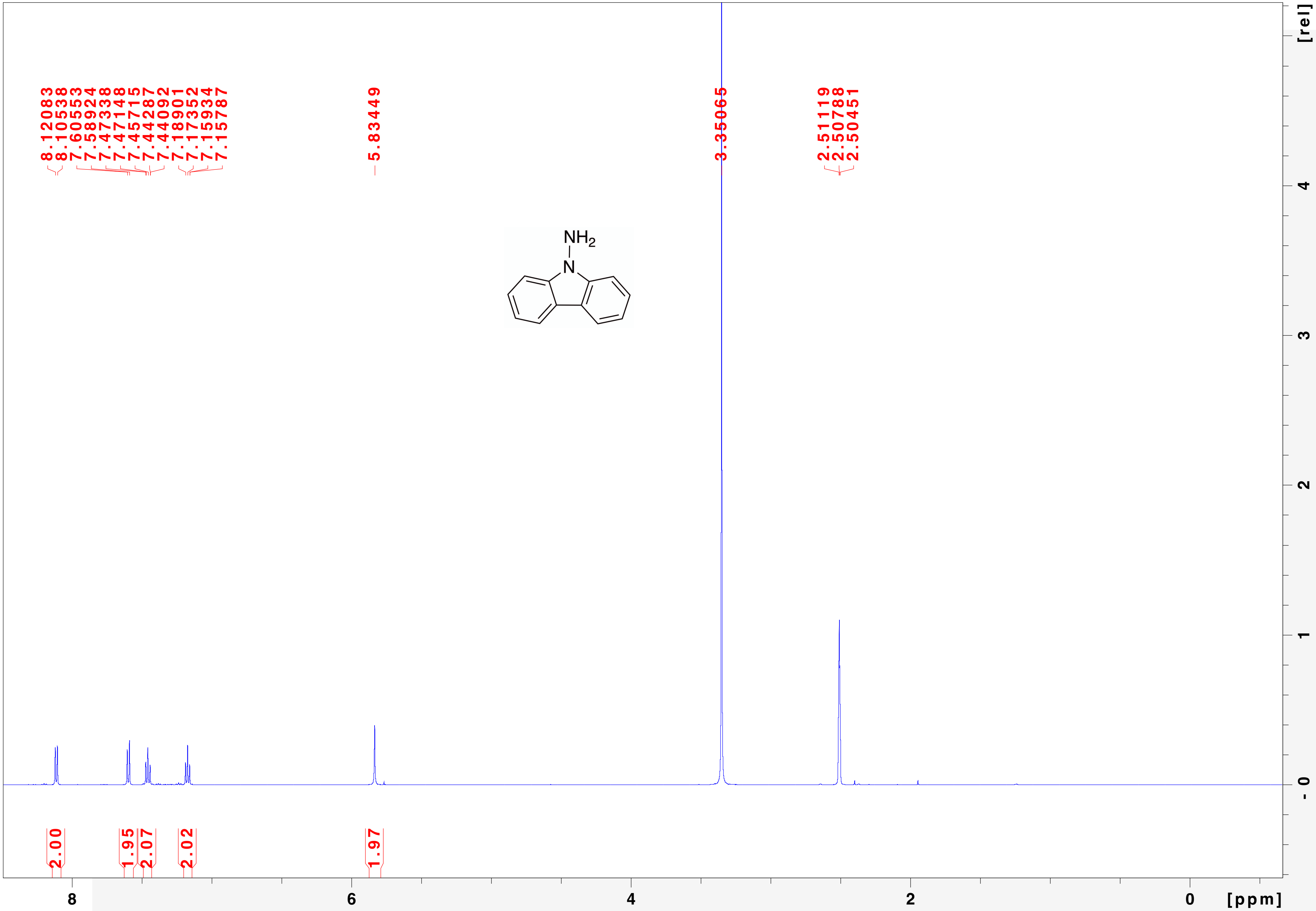

JI-II-45 20 1 /home/nmruser/networkdata/Bruker500Data/terungwa@chem.byu.edu

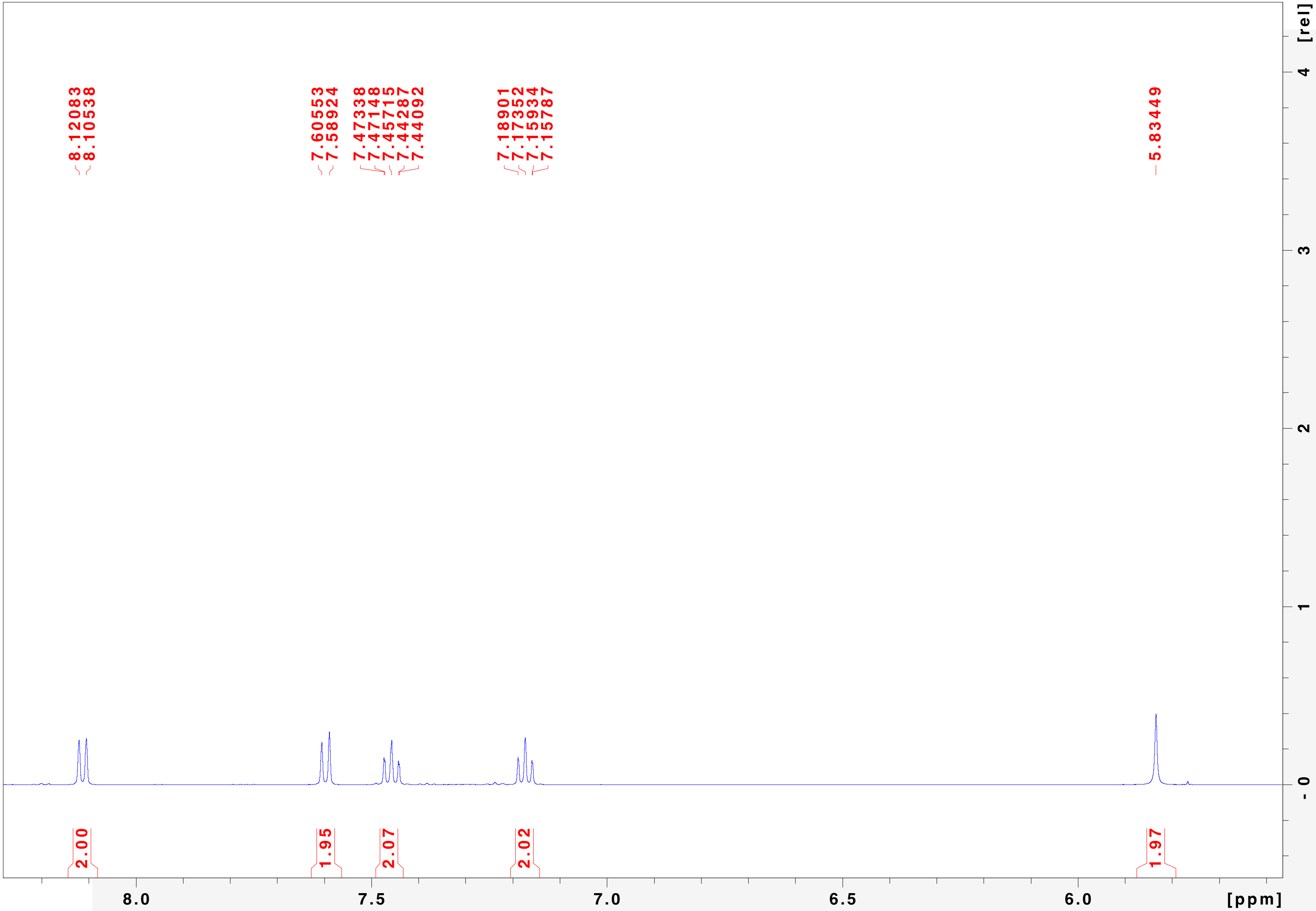

JI-II-45 21 1 /home/nmruser/networkdata/Bruker500Data/terungwa@chem.byu.edu

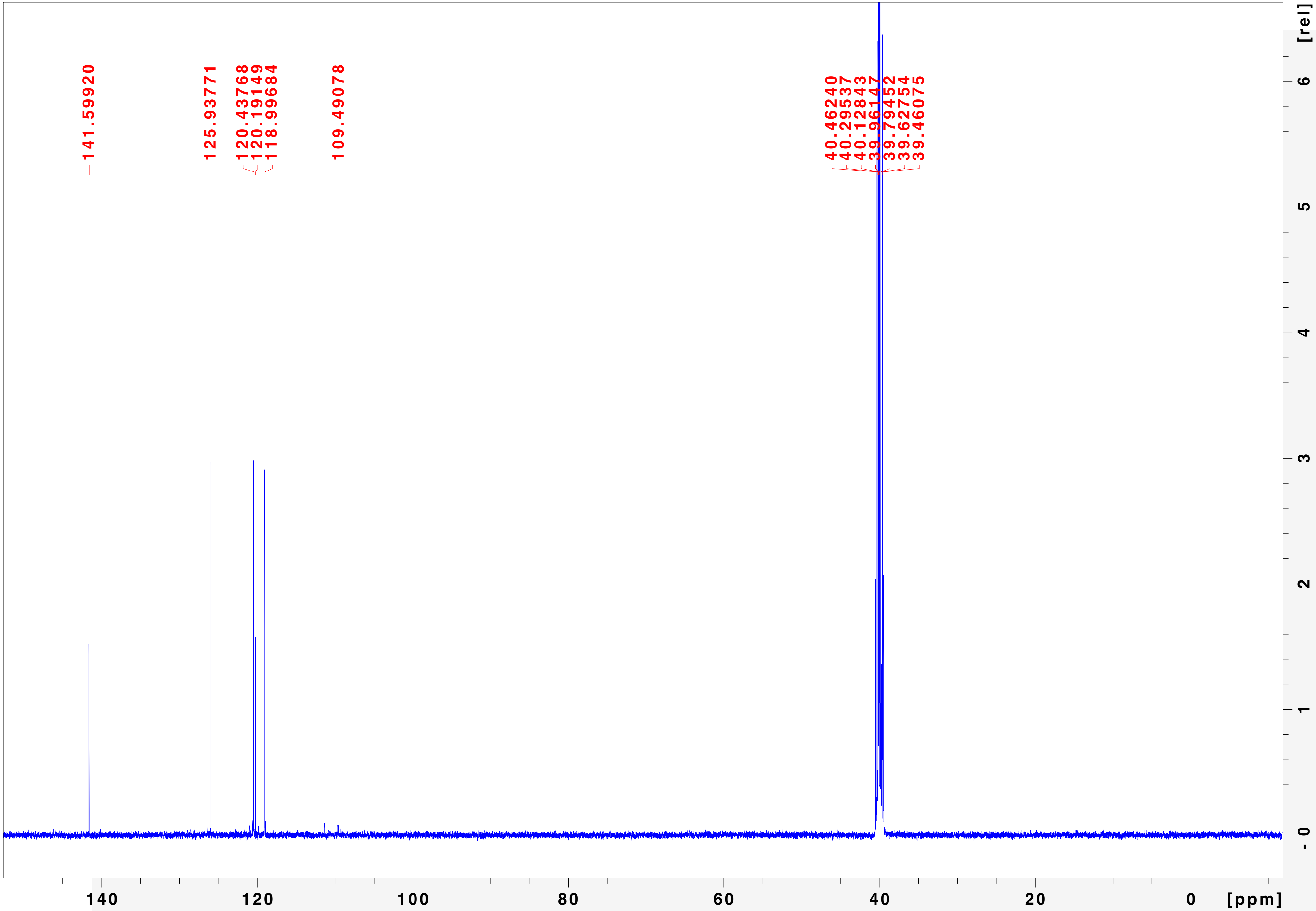

JI-II-45 21 1 /home/nmruser/networkdata/Bruker500Data/terungwa@chem.byu.edu

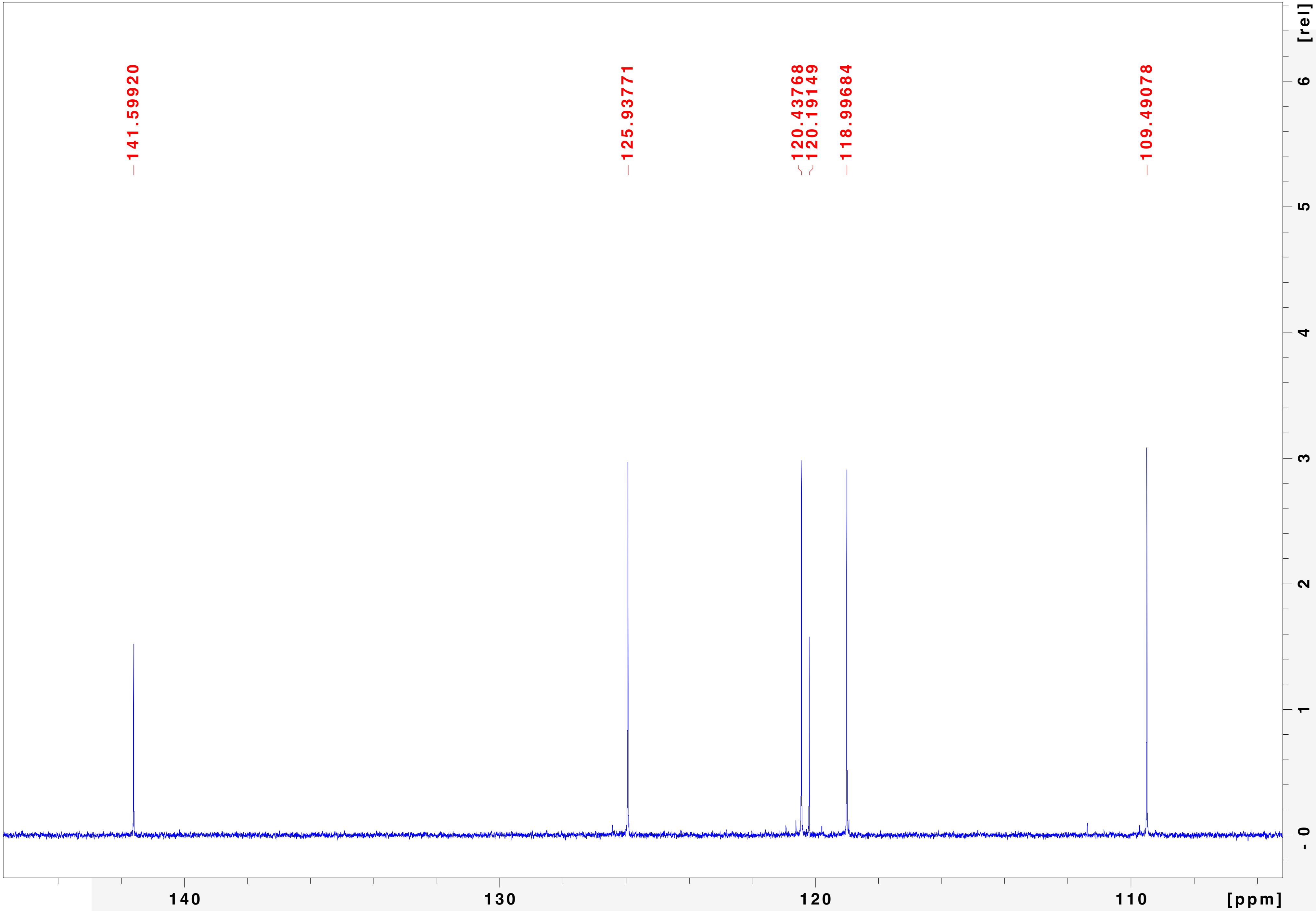

JI-II-71 20 1 /home/nmruser/networkdata/Bruker500Data/terungwa@chem.byu.edu

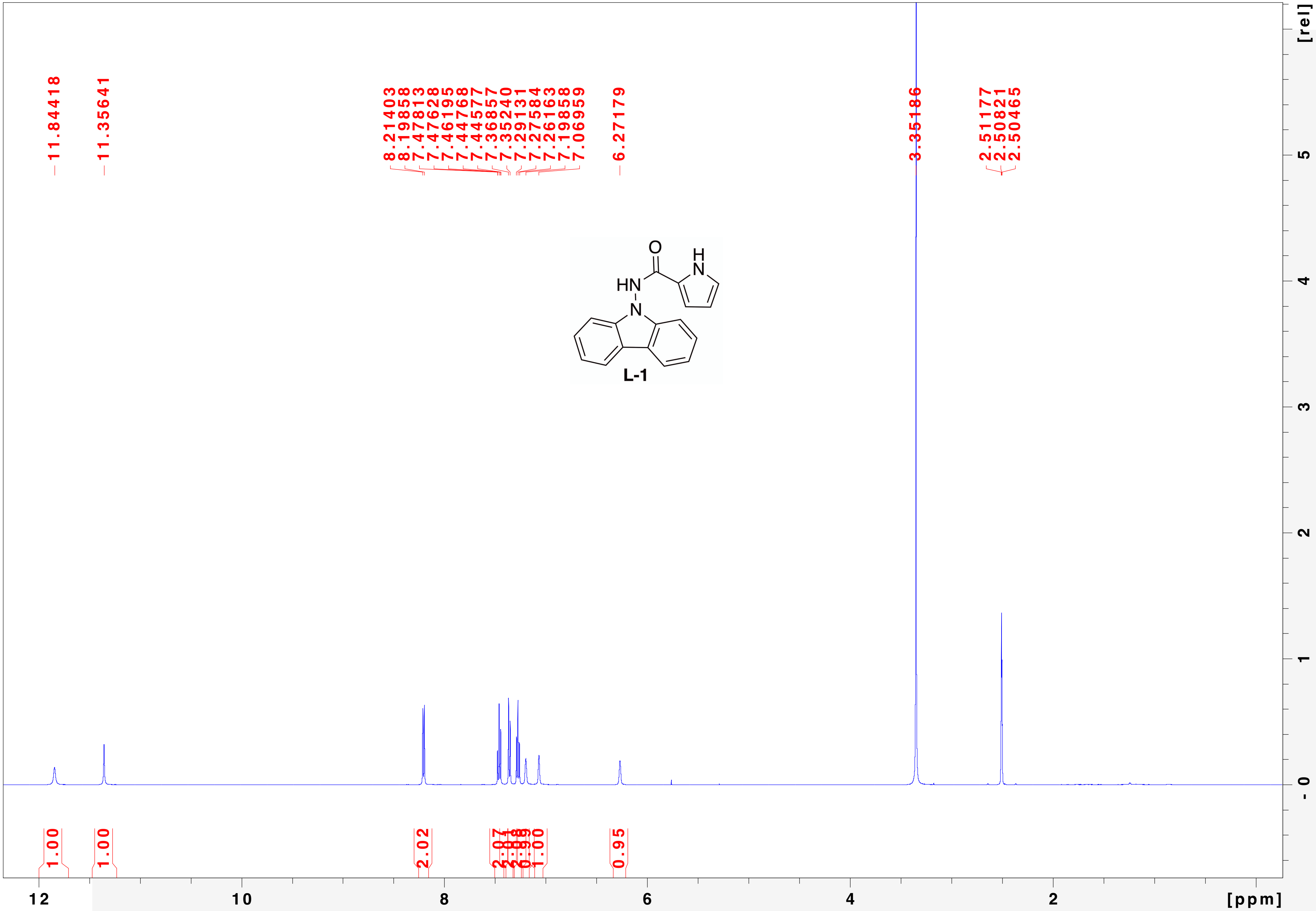

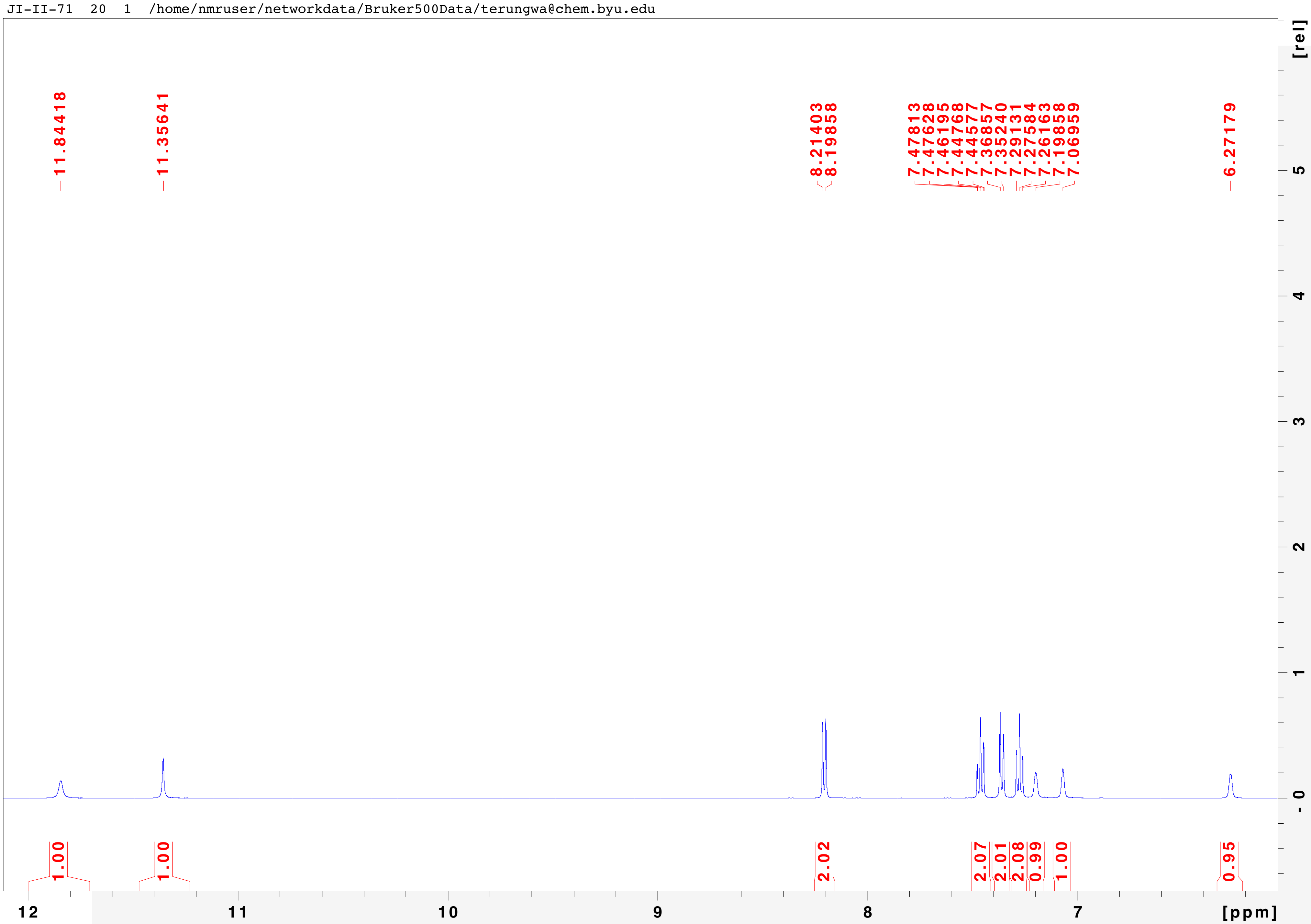
JI-II-71 20 1 /home/nmruser/networkdata/Bruker500Data/terungwa@chem.byu.edu
11.84418
11.35641
8.21403
8.19858
7.47813
7.47628
7.46195
7.44768
7.44577
7.36857
7.35240
7.29131
7.27584
7.26163
7.19858
7.06959
6.27179
1.00
1.00
2.02
2.07
2.01
2.08
0.99
1.00
0.95
12
11
10
9
8
7
[ppm]
[rel]
0
1
2
3
4
5

JI-II-71 20 1 /home/nmruser/networkdata/Bruker500Data/terungwa@chem.byu.edu

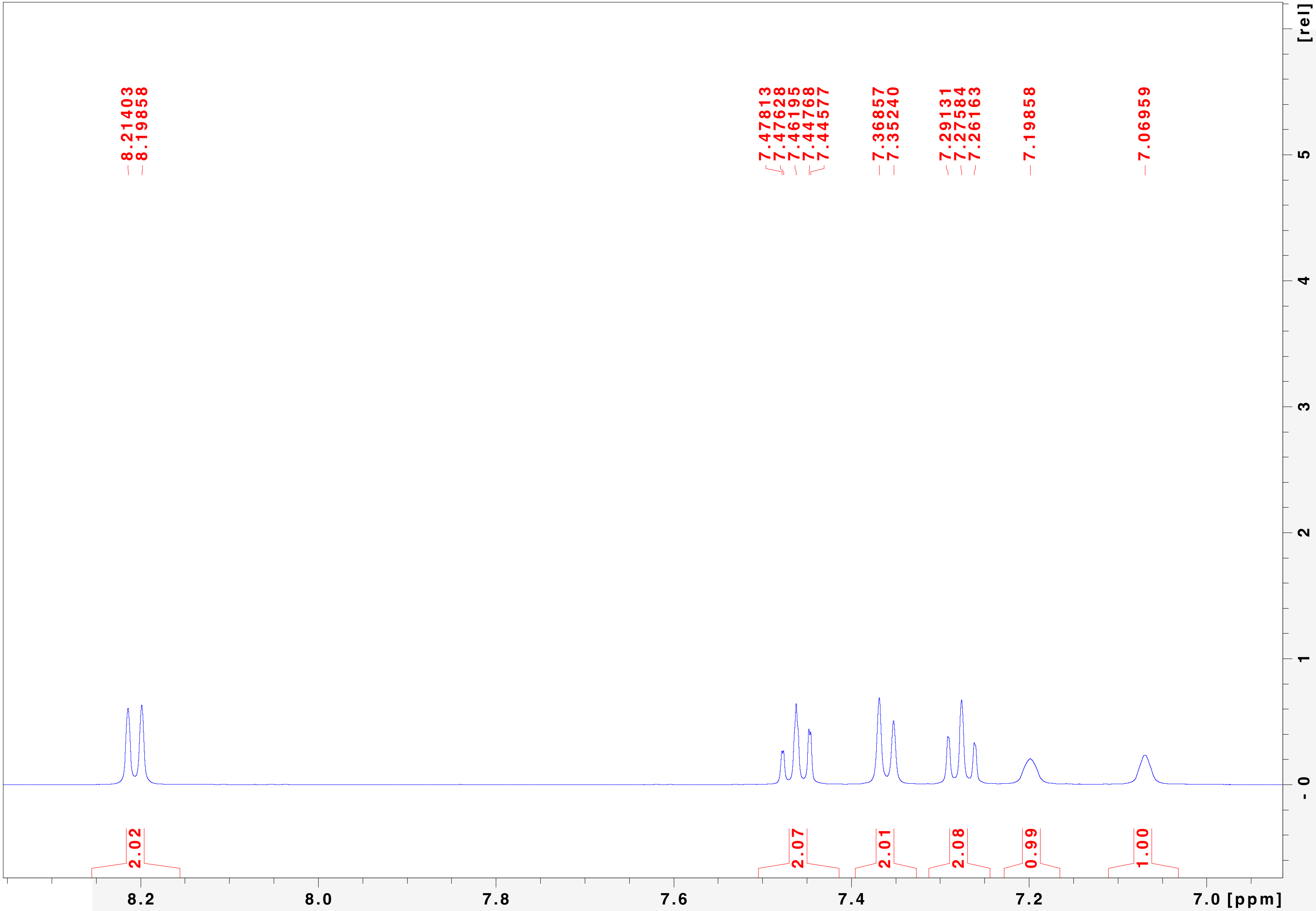

JI-II-71 21 1 /home/nmruser/networkdata/Bruker500Data/terungwa@chem.byu.edu

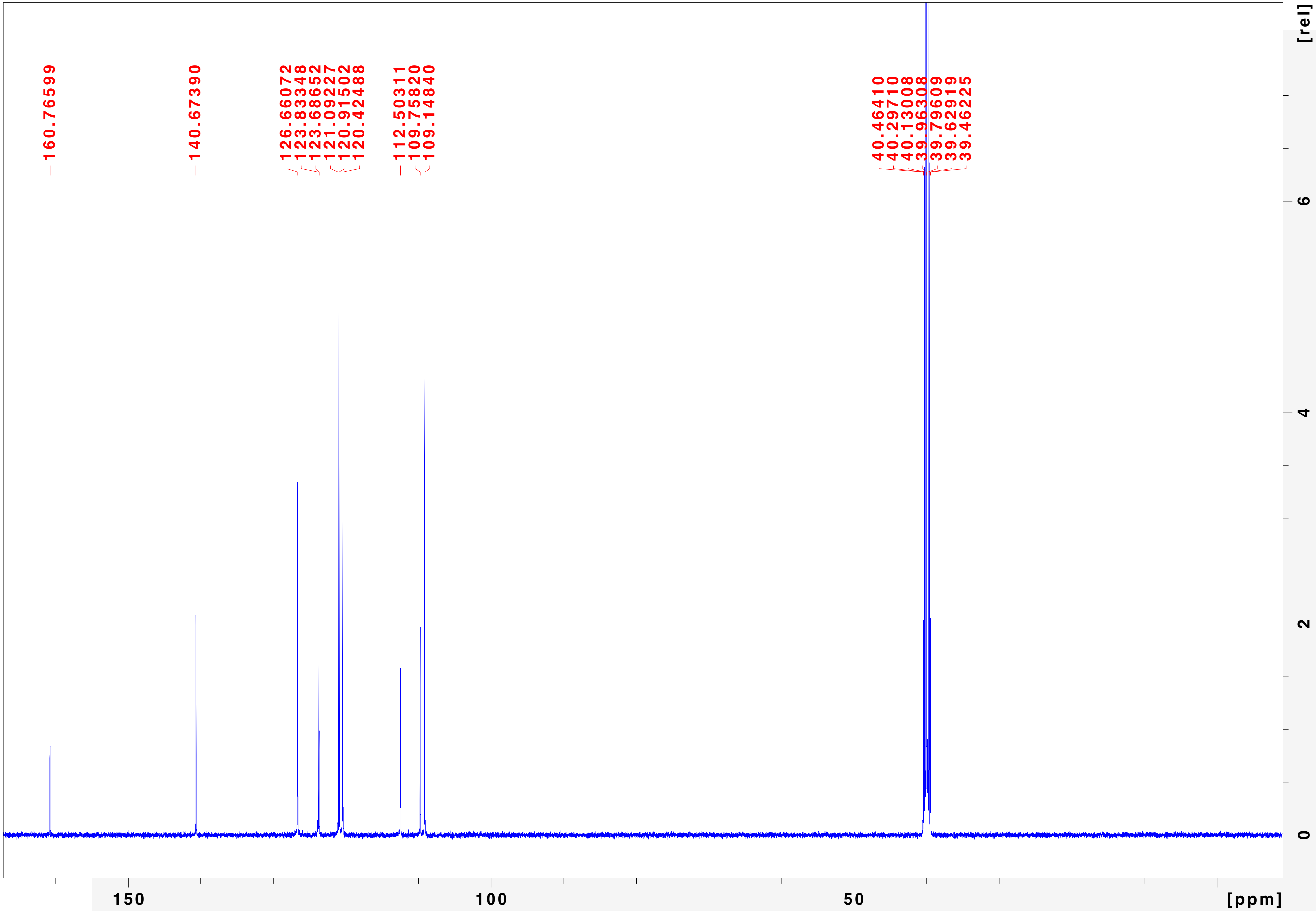

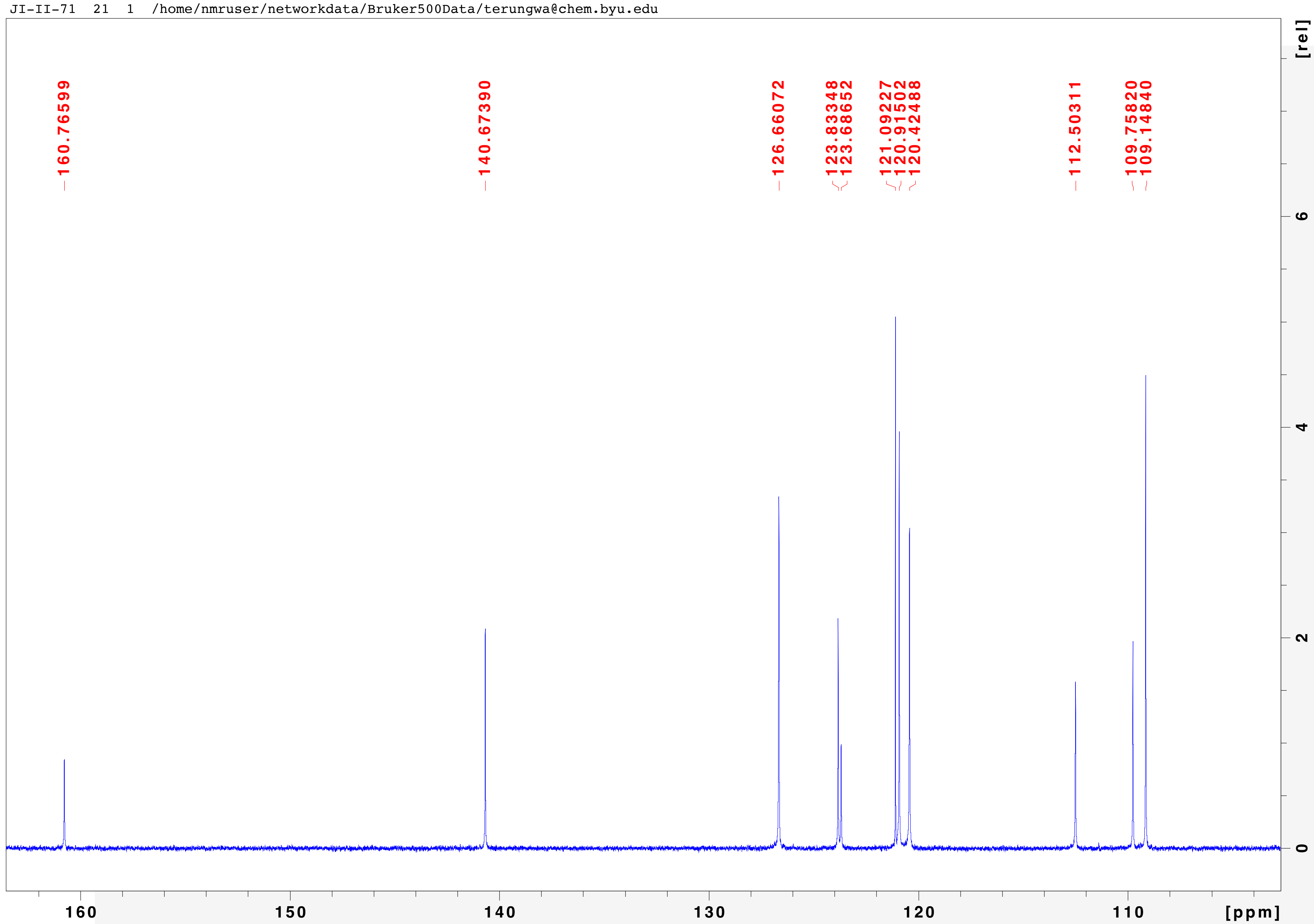
JI-II-71 21 1 /home/nmruser/networkdata/Bruker500Data/terungwa@chem.byu.edu
160.76599
140.67390
126.66072
123.83348
123.68652
121.09227
120.91502
120.42488
112.50311
109.75820
109.14840
[rel]
[ppm]

LG-I-39 40 1 /home/nmruser/networkdata/Bruker500Data/log96@chem.byu.edu

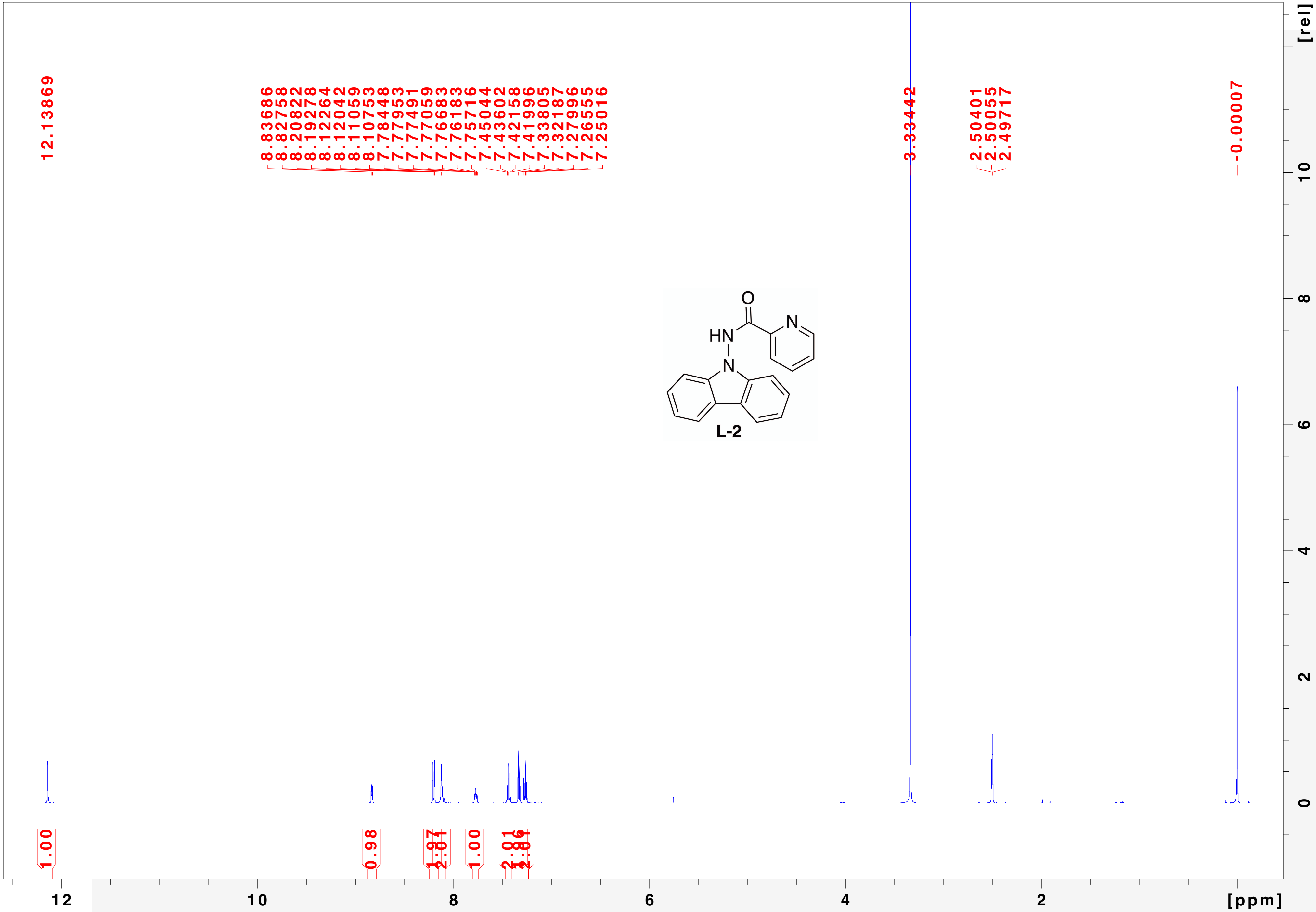

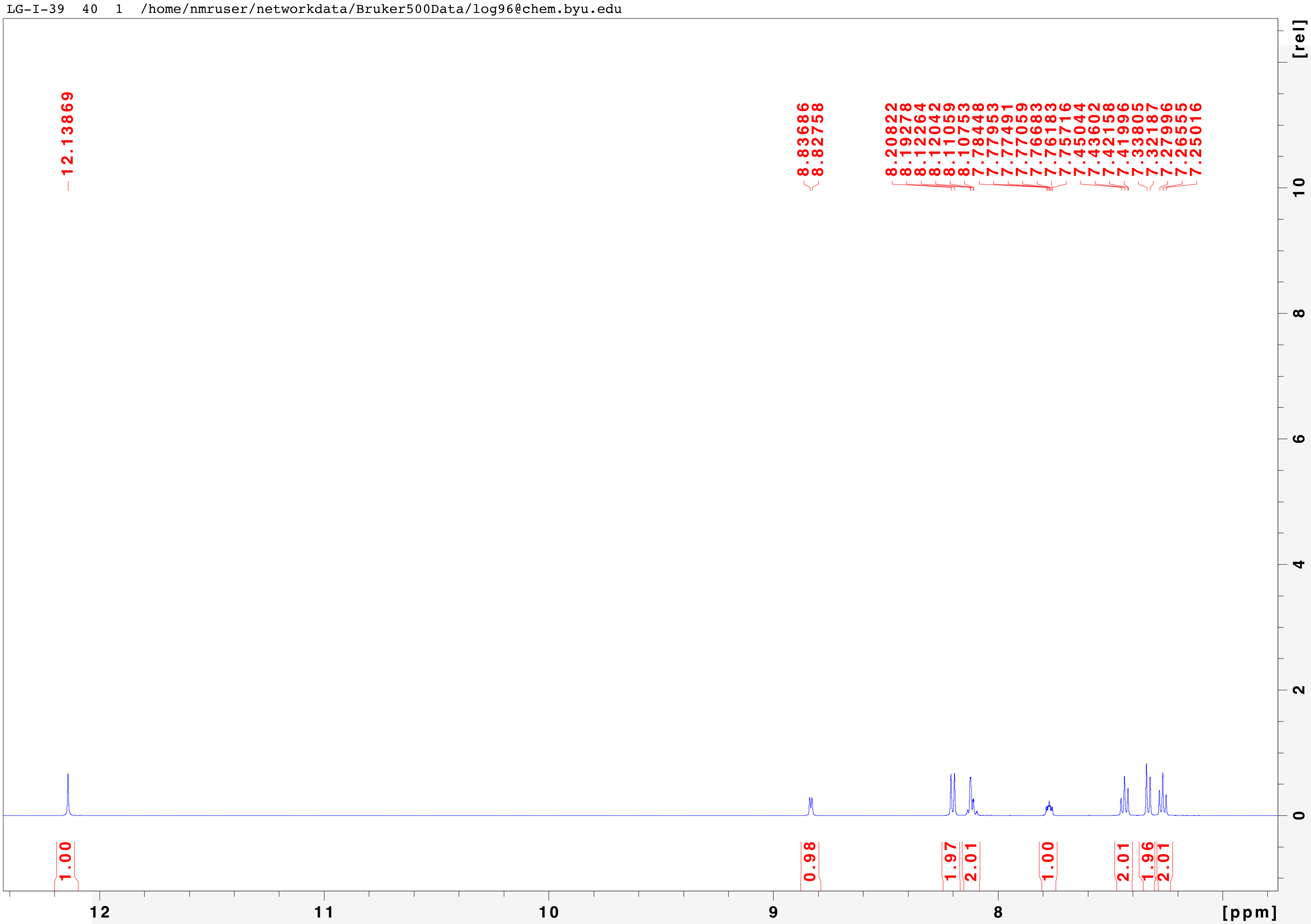
LG-I-39 40 1 /home/nmruser/networkdata/Bruker500Data/log96@chem.byu.edu
12.13869
8.83686
8.82758
8.20822
8.19278
8.12264
8.12042
8.11059
8.10753
7.78448
7.77953
7.77491
7.77059
7.76683
7.76183
7.75716
7.45044
7.43602
7.42158
7.41996
7.33805
7.32187
7.27996
7.26555
7.25016
1.00
0.98
1.97
2.01
1.00
2.01
1.96
2.01
[rel]
[ppm]

LG-I-39 50 1 /home/nmruser/networkdata/Bruker500Data/log96@chem.byu.edu

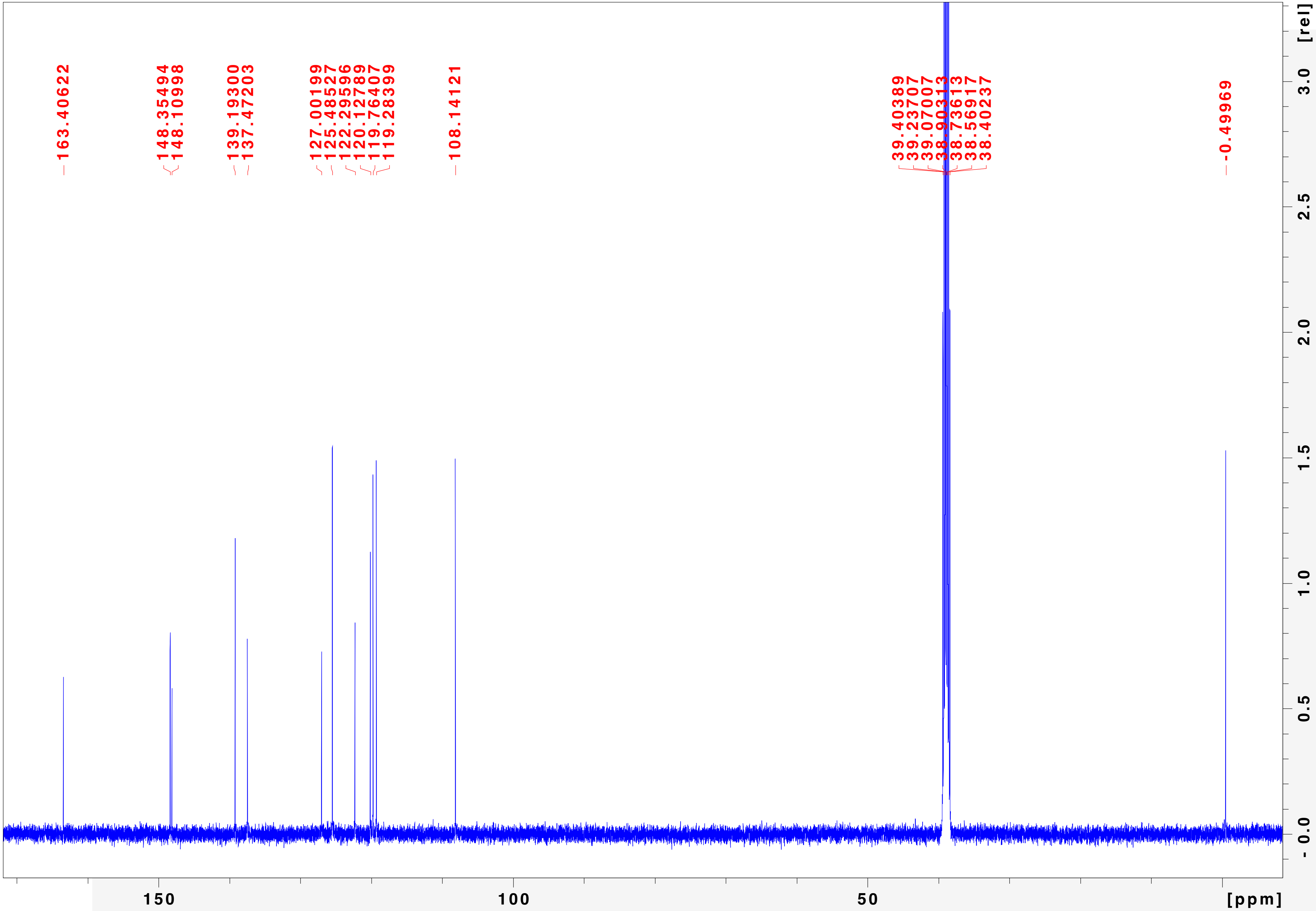

LG-I-39 50 1 /home/nmruser/networkdata/Bruker500Data/log96@chem.byu.edu

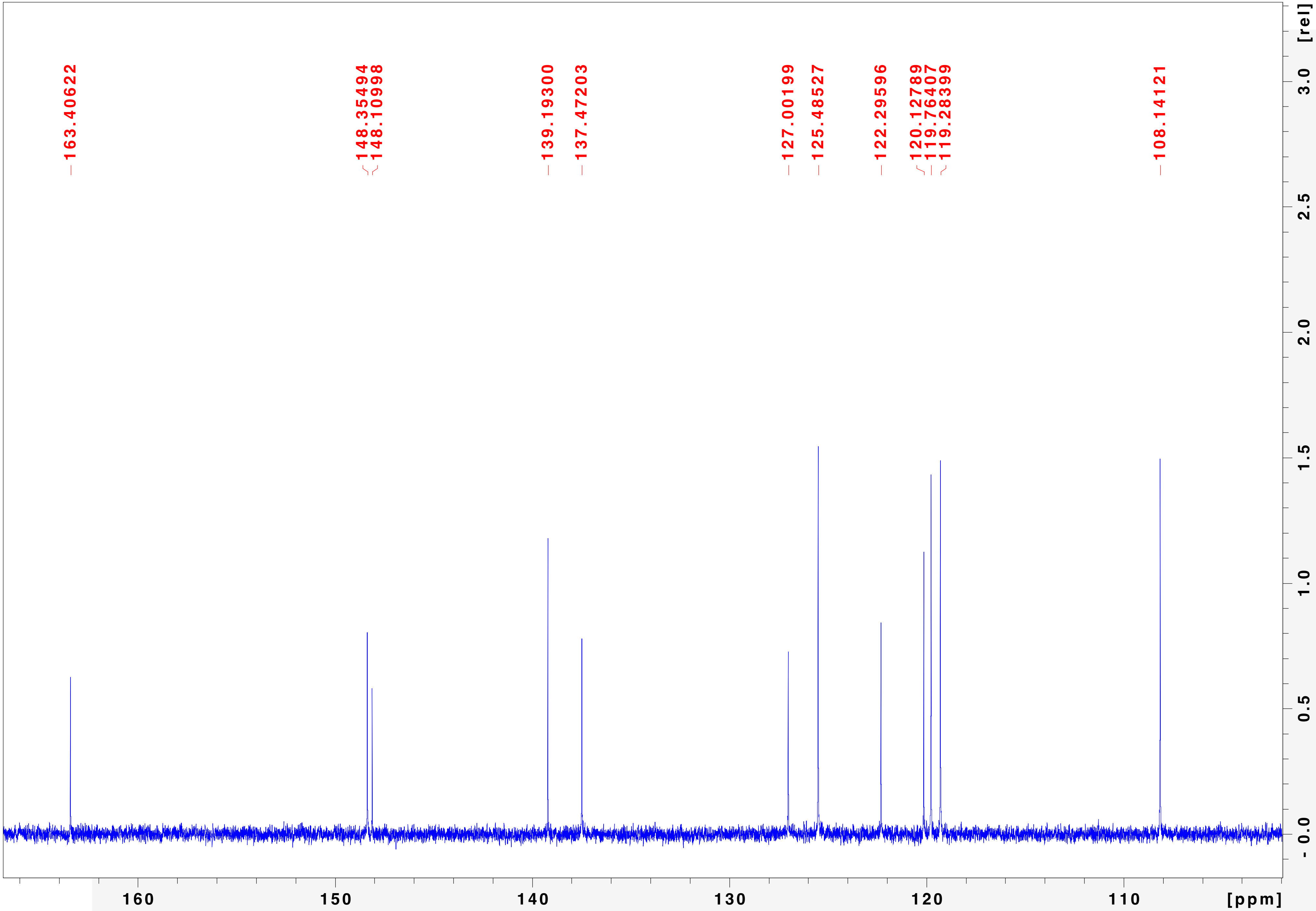

74 10 1 /home/nmruser/networkdata/Bruker500Data/log96@chem.byu.edu

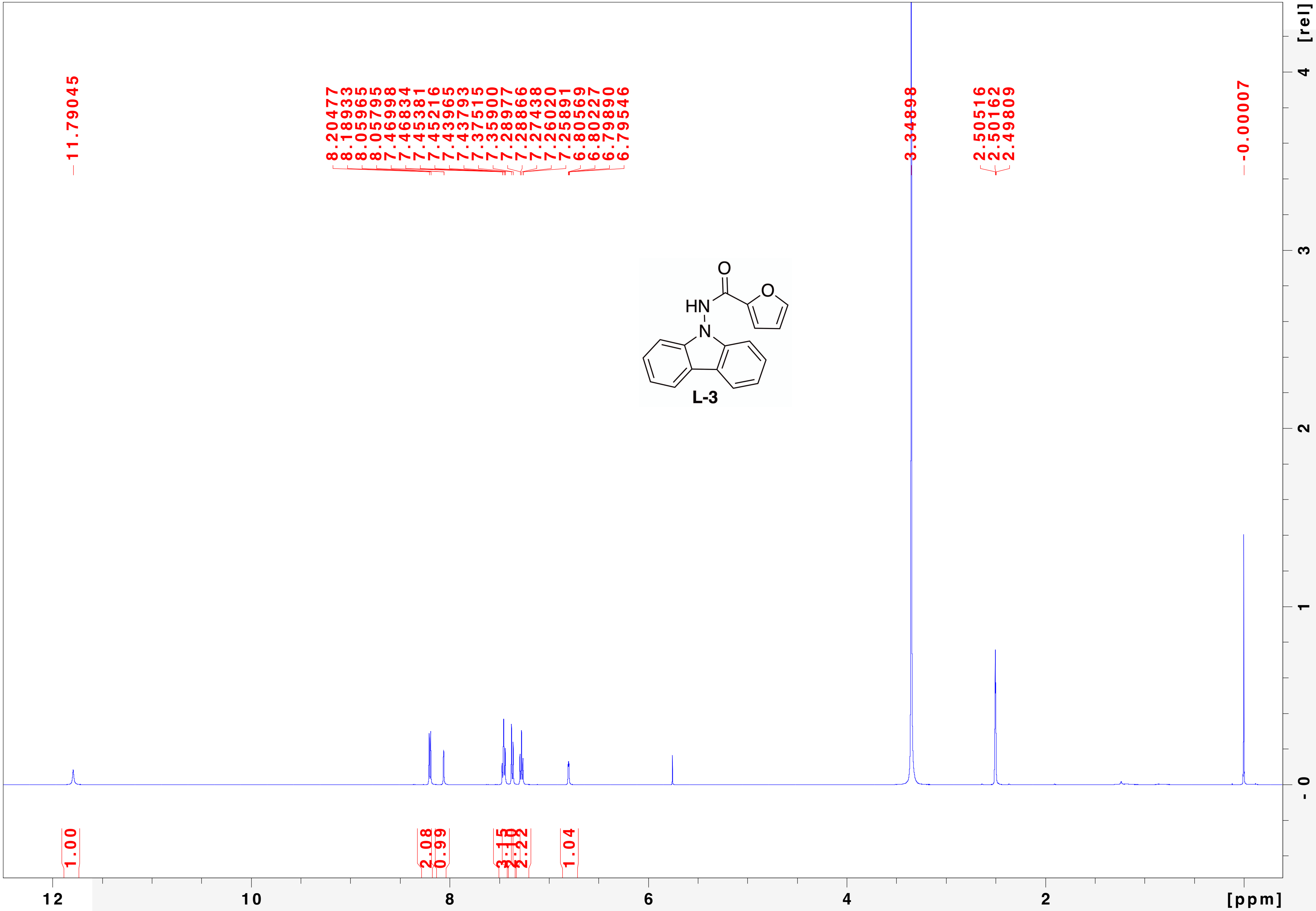

74 10 1 /home/nmruser/networkdata/Bruker500Data/log96@chem.byu.edu

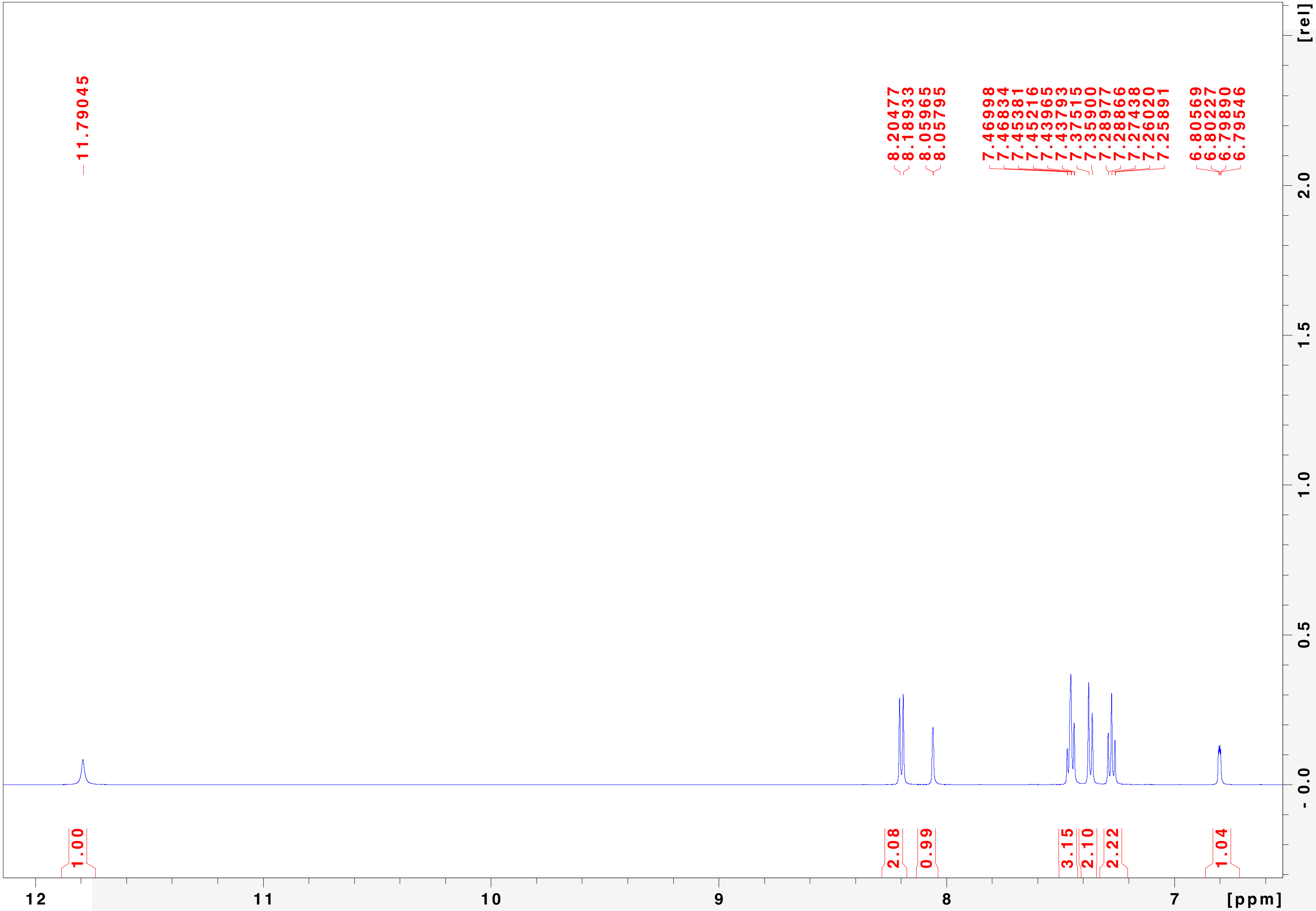

"Compund 74C" 10 1 /home/nmruser/networkdata/Bruker500Data/log96@chem.byu.edu

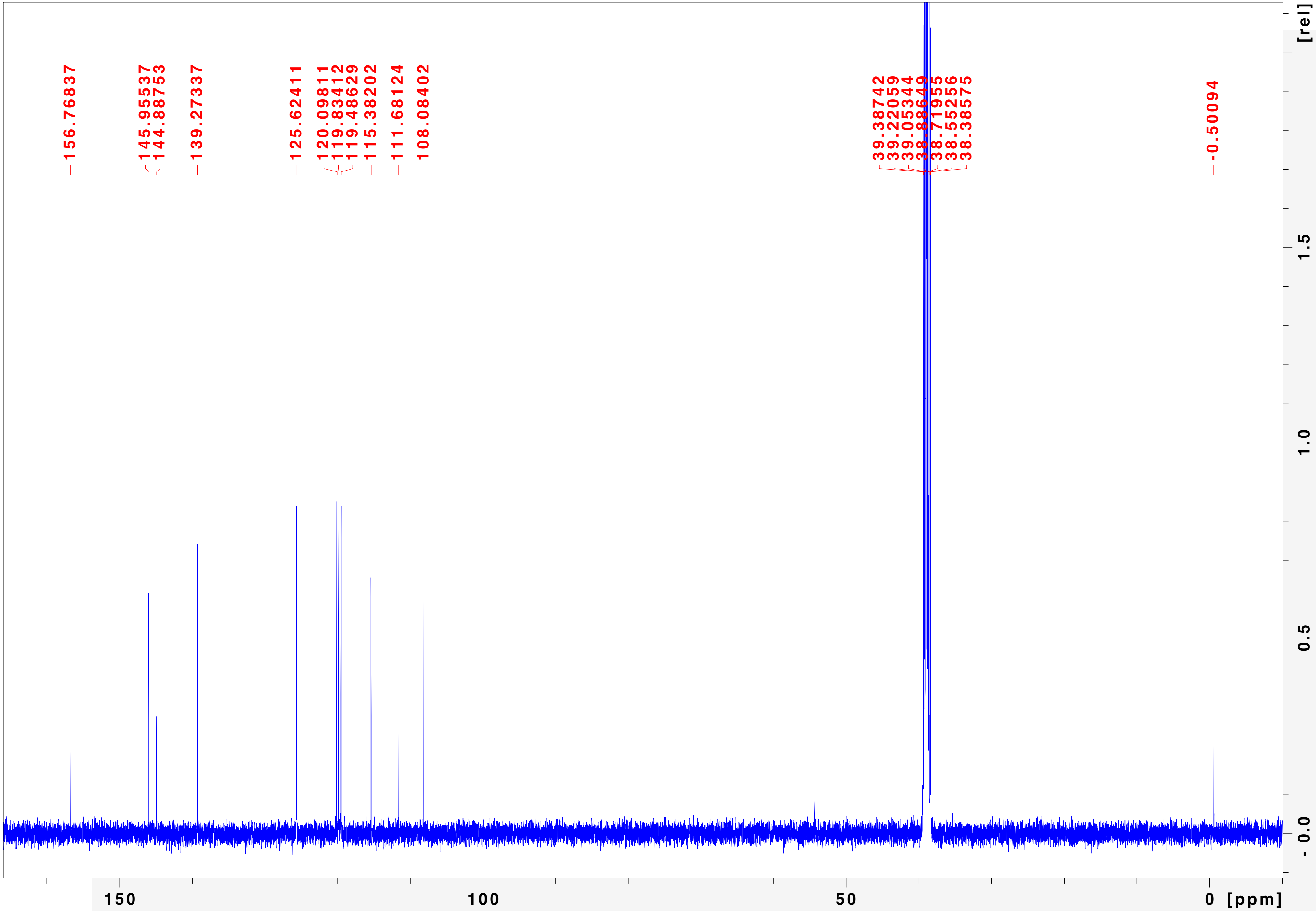

"Compund 74C"   10 1 /home/nmruser/networkdata/Bruker500Data/log96@chem.byu.edu

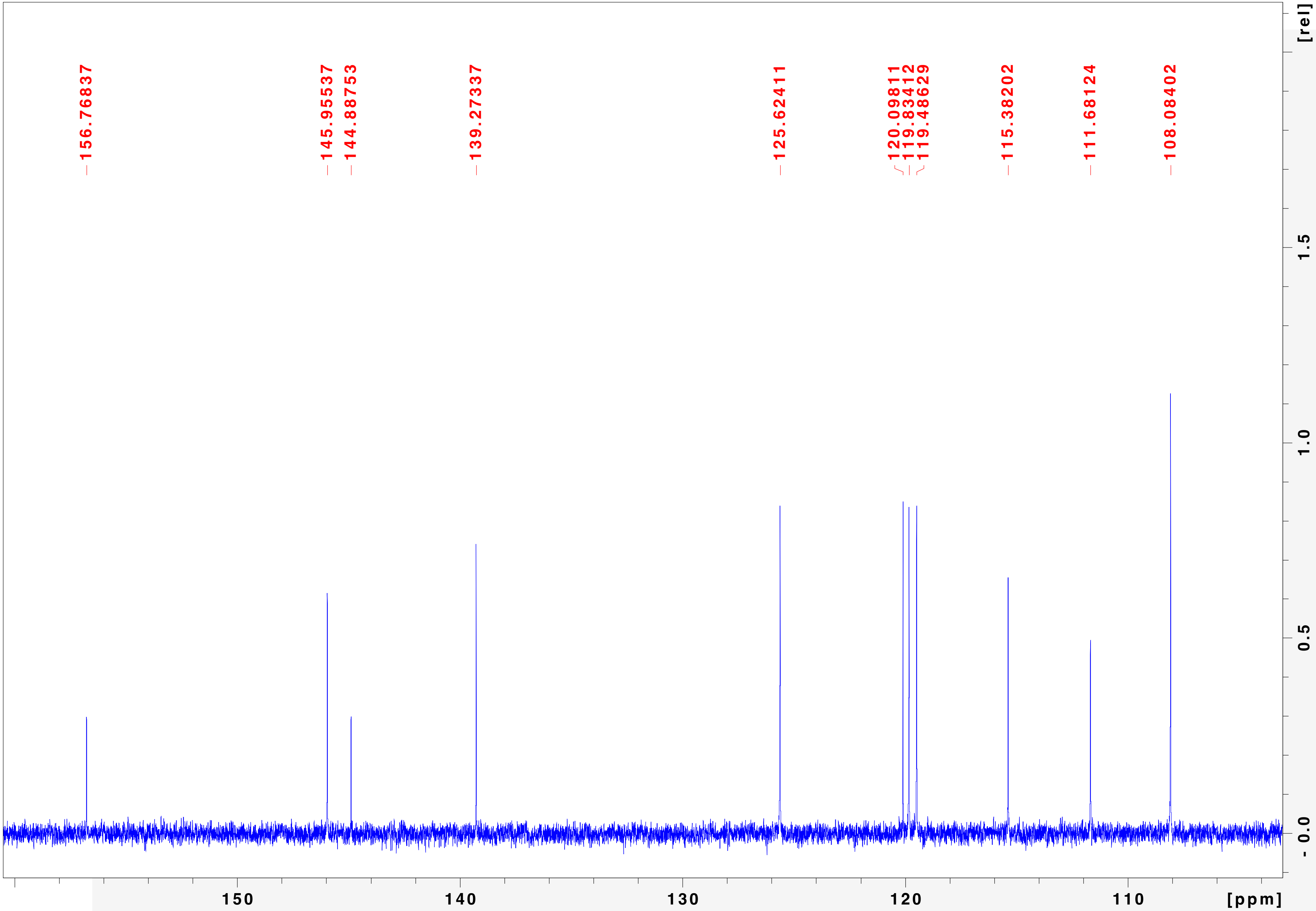

LG-I-38 20 1 /home/nmruser/networkdata/Bruker500Data/log96@chem.byu.edu

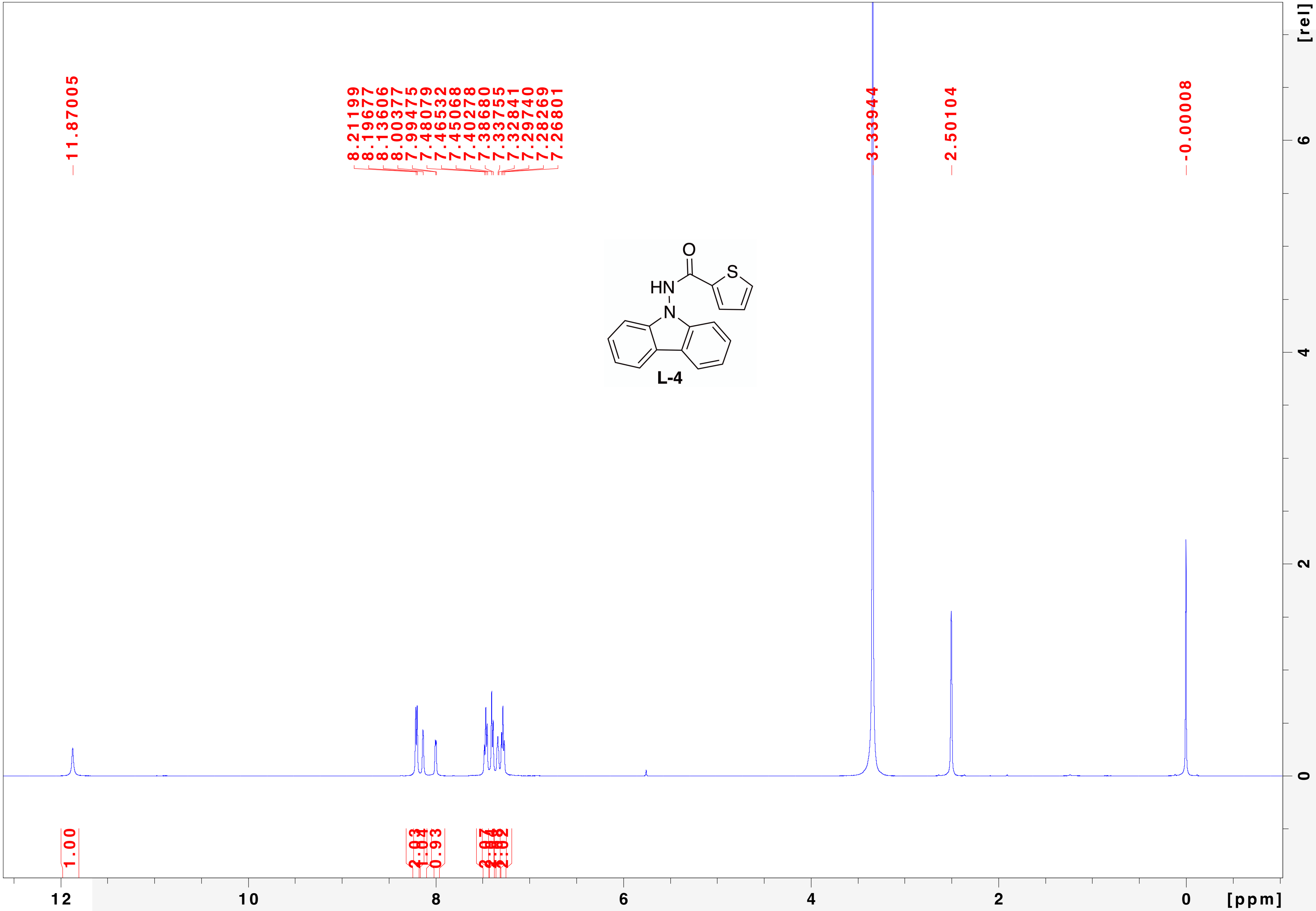

LG-I-38 20 1 /home/nmruser/networkdata/Bruker500Data/log96@chem.byu.edu

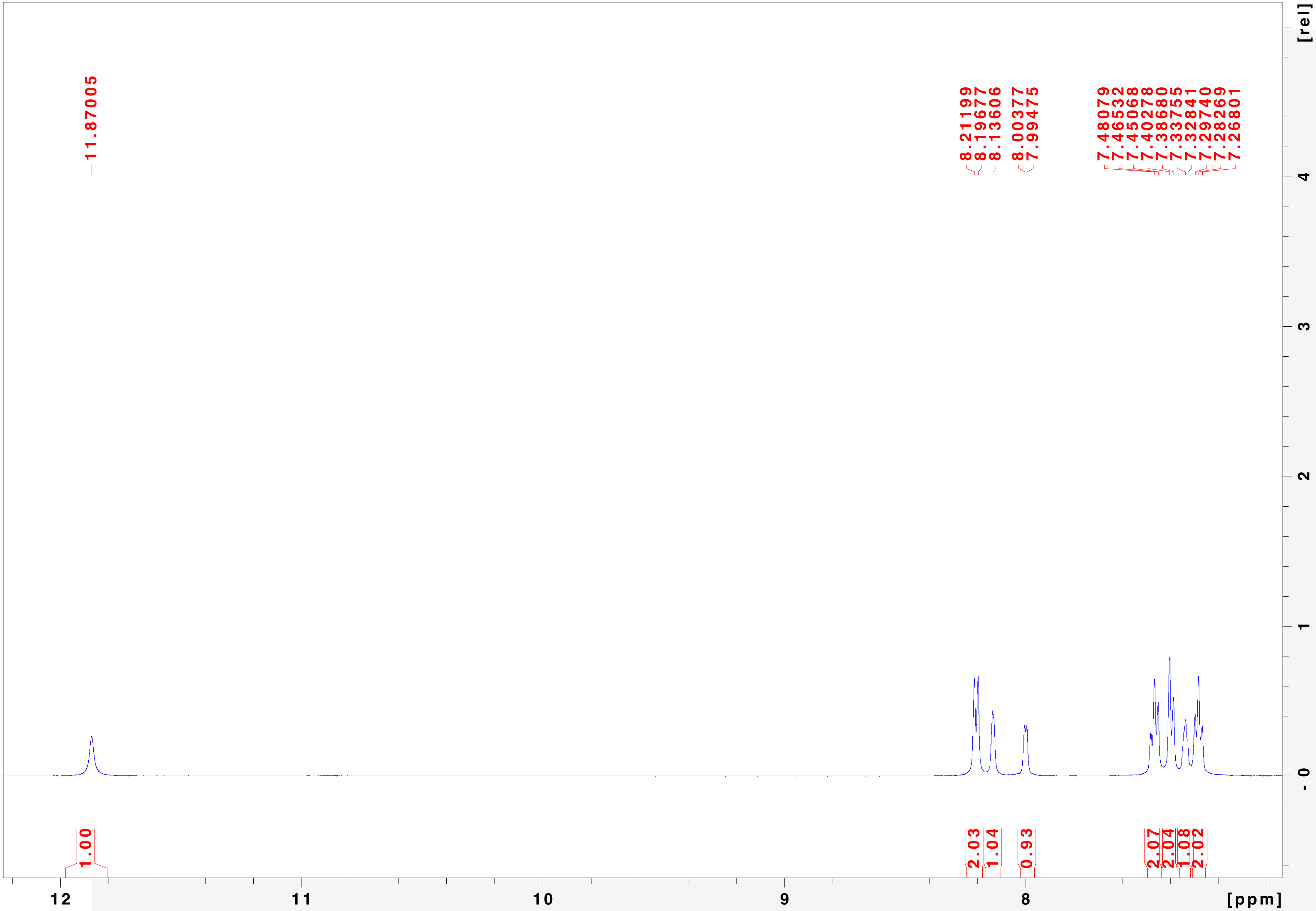

LG-I-38 30 1 /home/nmruser/networkdata/Bruker500Data/log96@chem.byu.edu

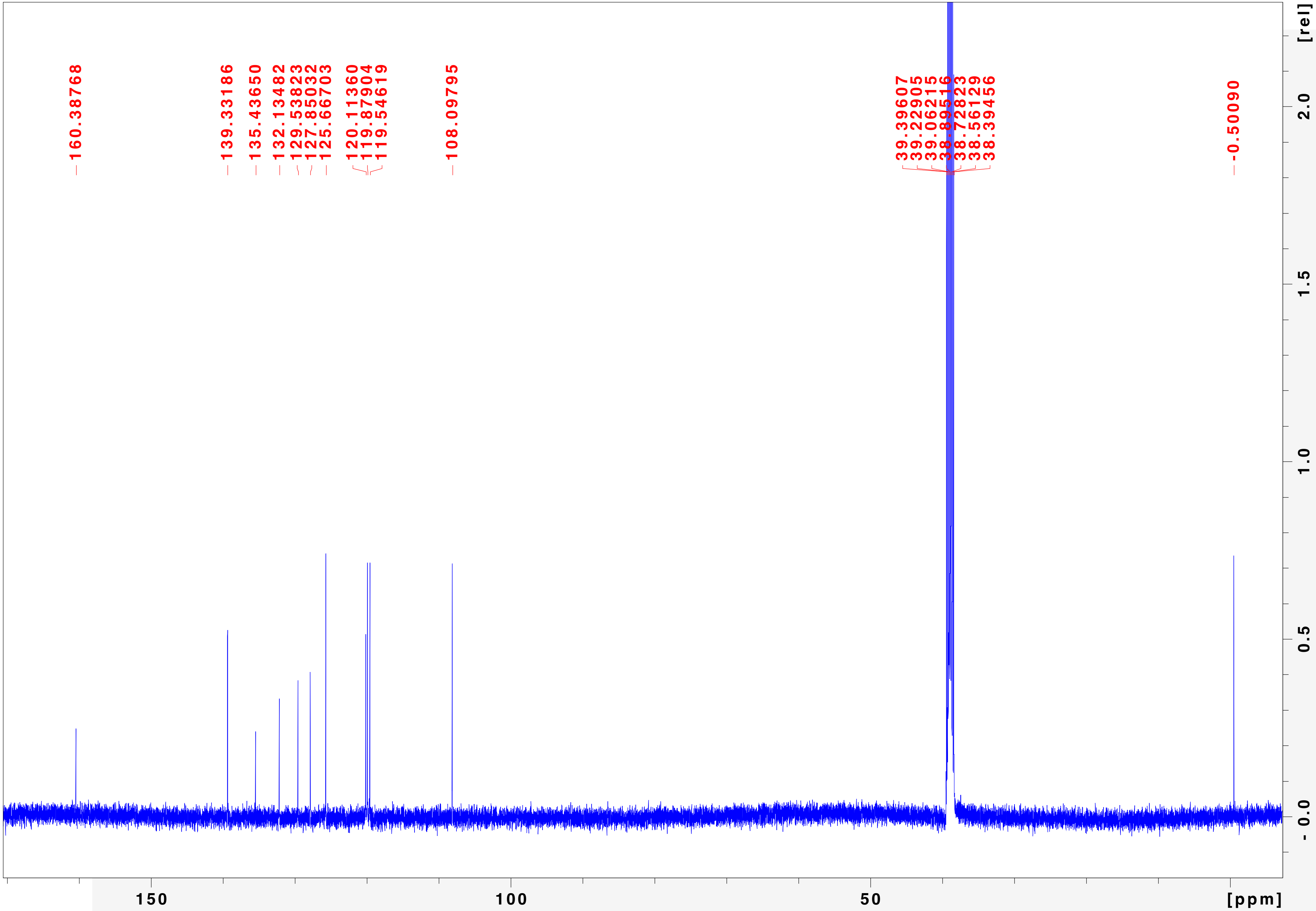

LG-I-38 30 1 /home/nmruser/networkdata/Bruker500Data/log96@chem.byu.edu

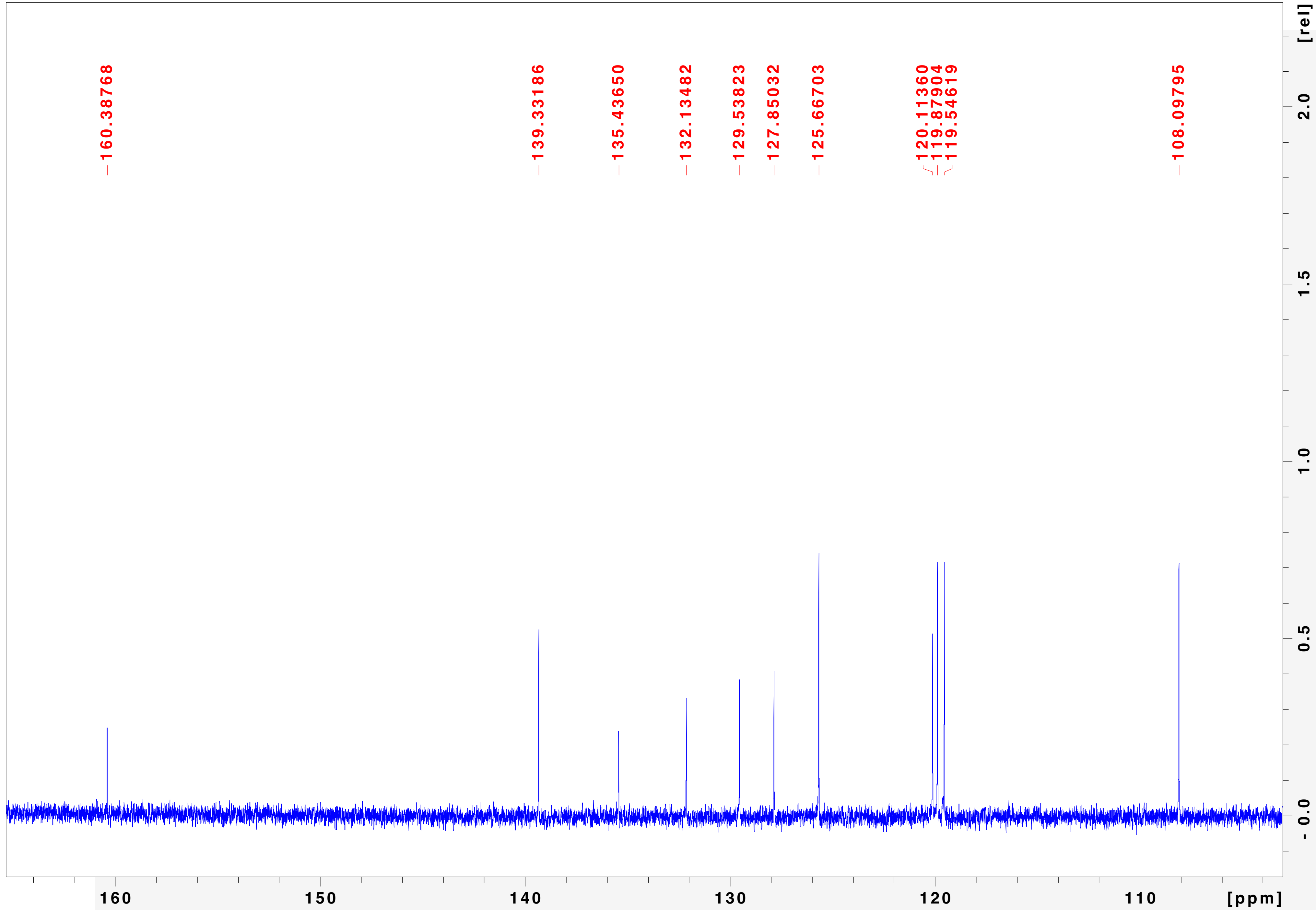

JI-II-44   10   1   /home/nmruser/networkdata/Bruker500Data/terungwa@chem.byu.edu

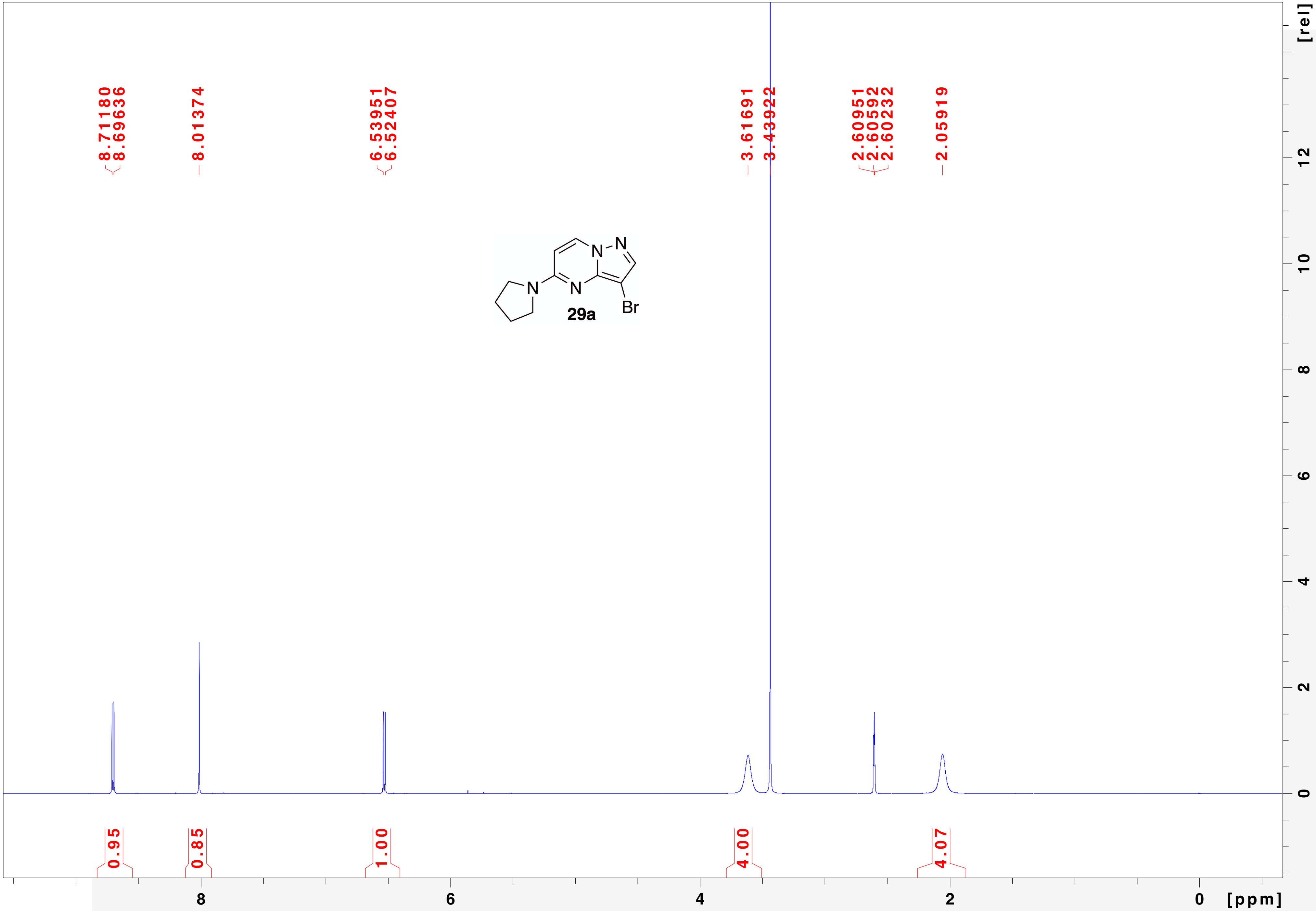

JI-II-44 20 1 /home/nmruser/networkdata/Bruker500Data/terungwa@chem.byu.edu

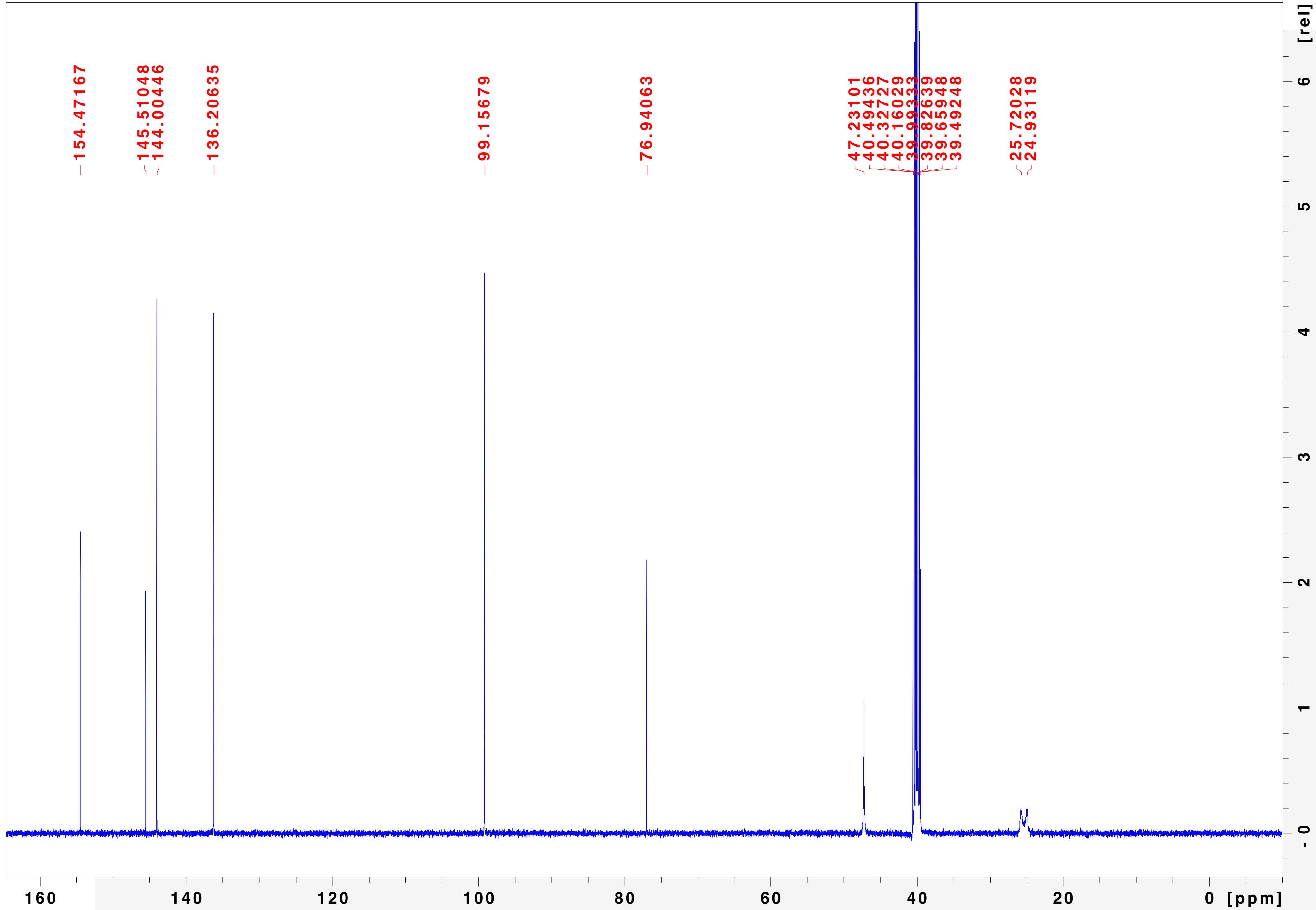

"BAT - II - 58" 2 1 /home/nmruser/networkdata/Bruker500Data/bat54@chem.byu.edu

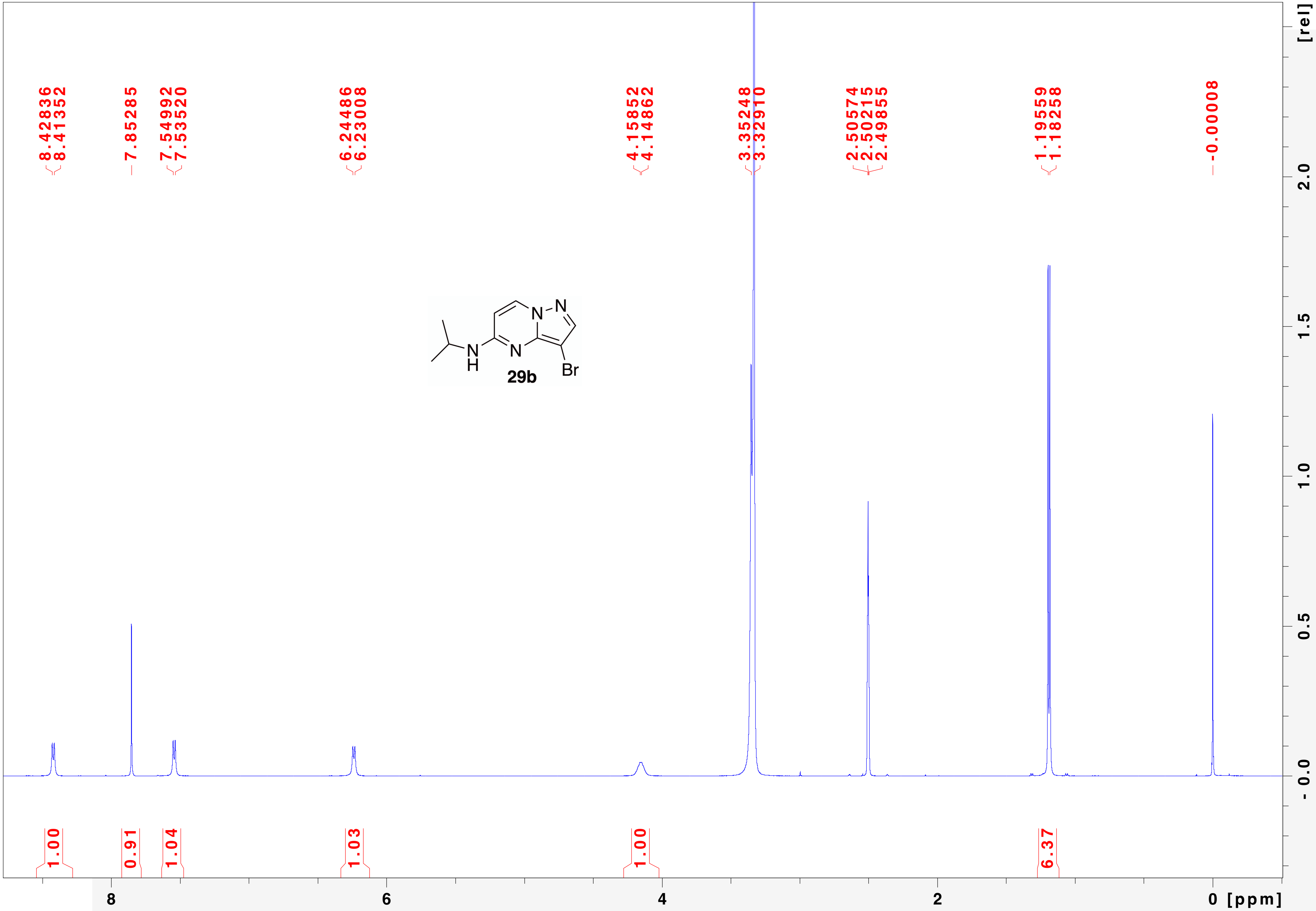

"BAT - II - 58" 1 1 /home/nmruser/networkdata/Bruker500Data/bat54@chem.byu.edu

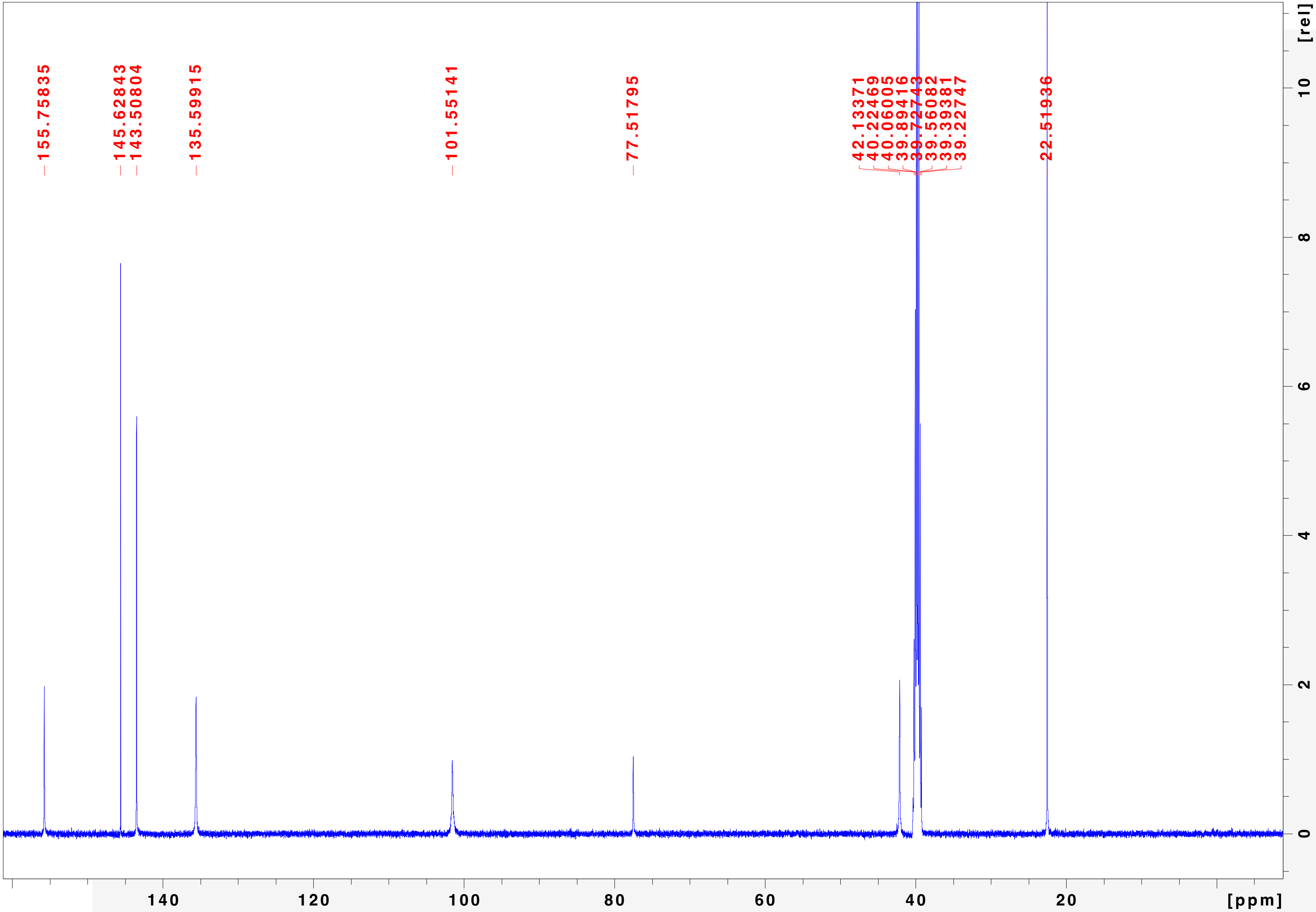

JI-III-41 10 1 /home/nmruser/networkdata/Bru500/terungwa@chem.byu.edu

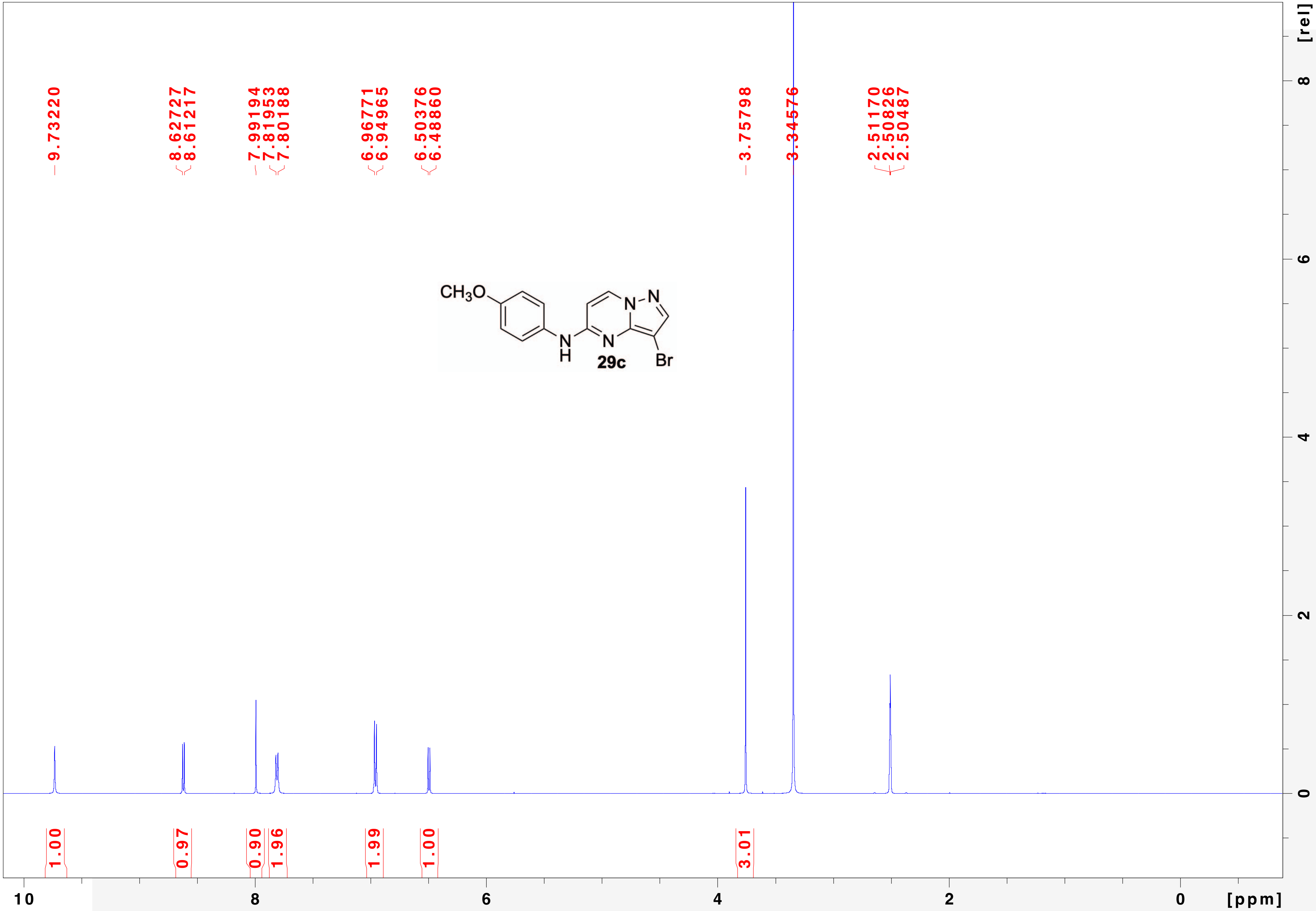

JI-III-41 10 1 /home/nmruser/networkdata/Bru500/terungwa@chem.byu.edu

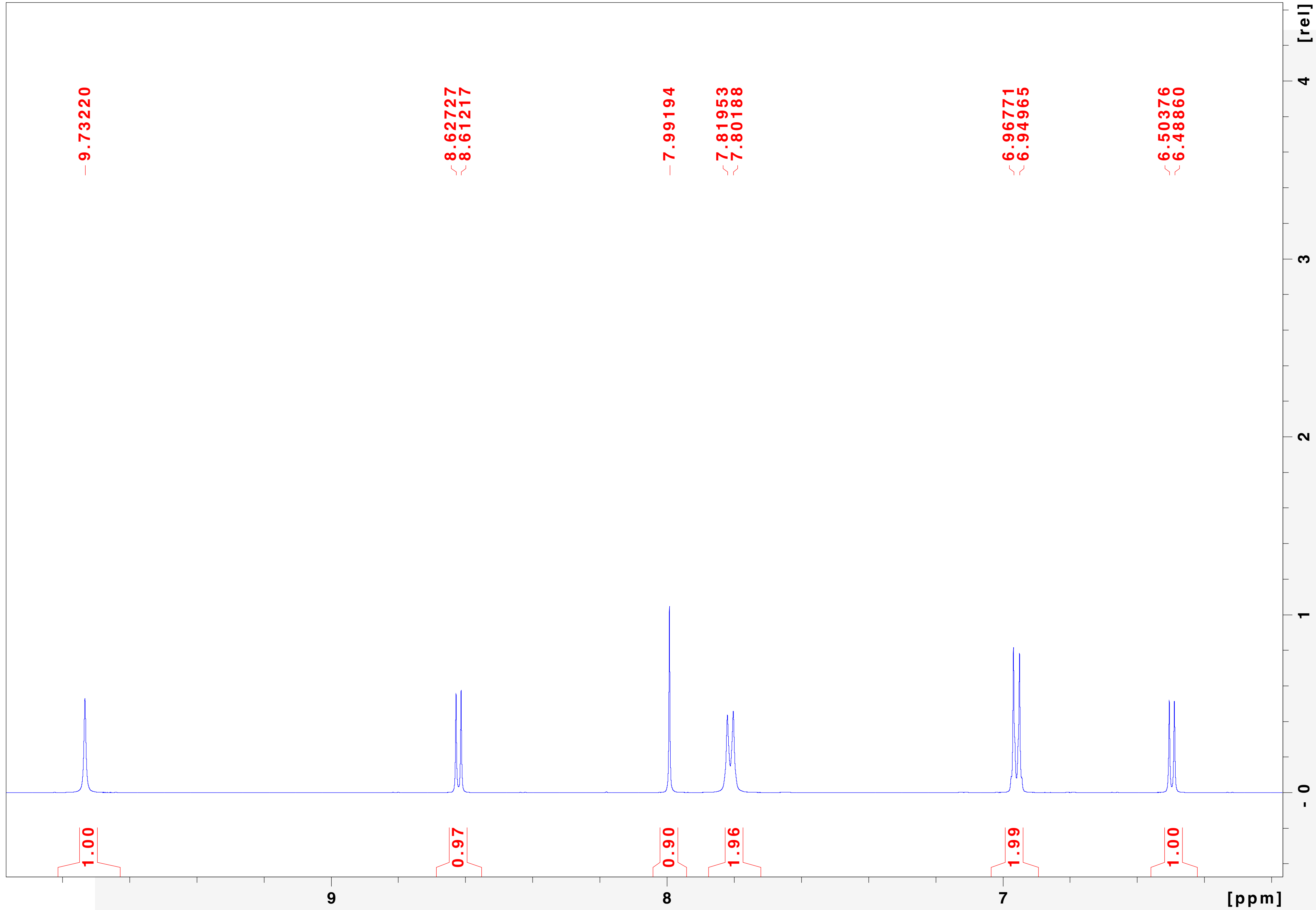

JI-III-41 20 1 /home/nmruser/networkdata/Bru500/terungwa@chem.byu.edu

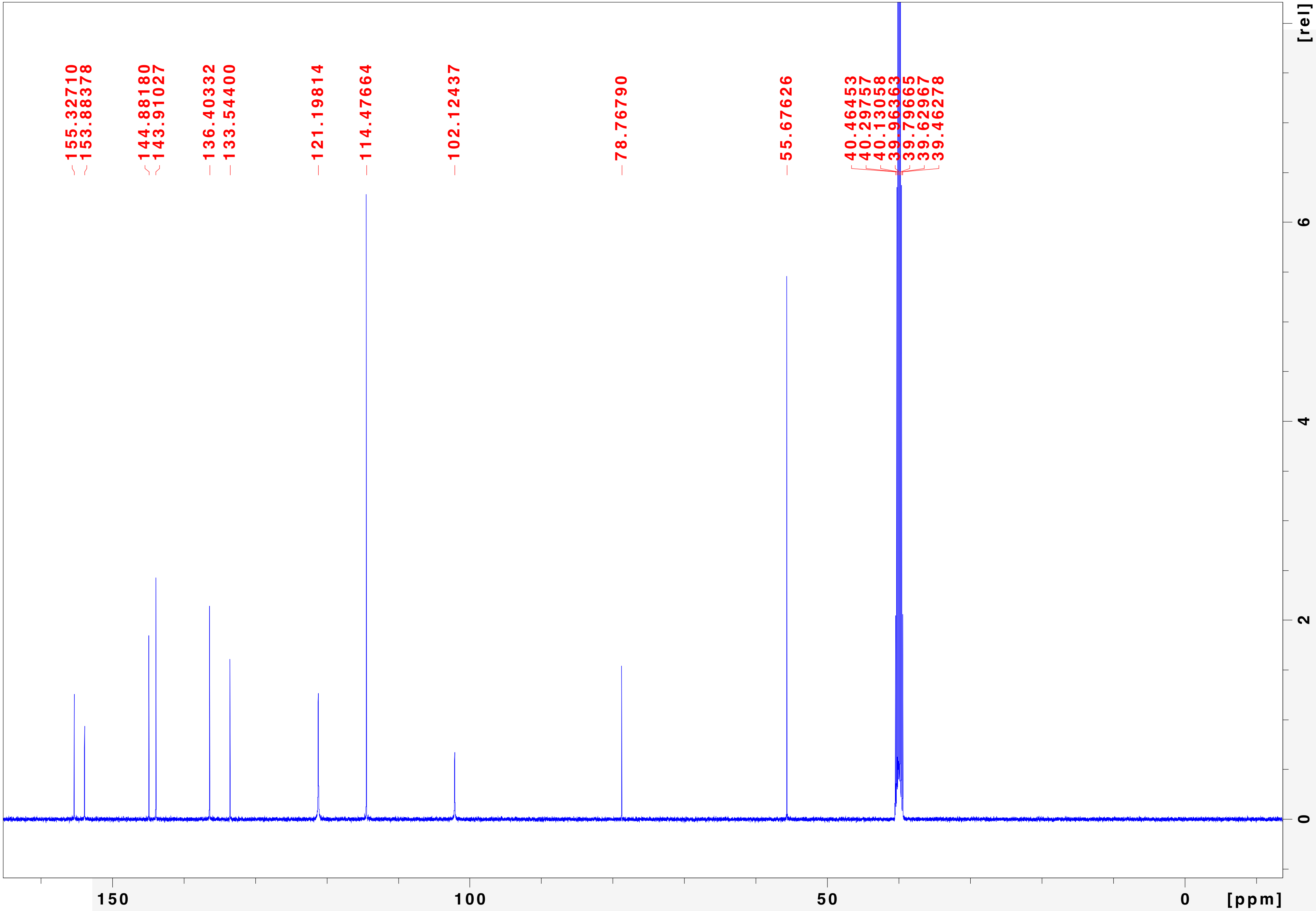

JI-III-40 10 1 /home/nmruser/networkdata/Bru500/terungwa@chem.byu.edu

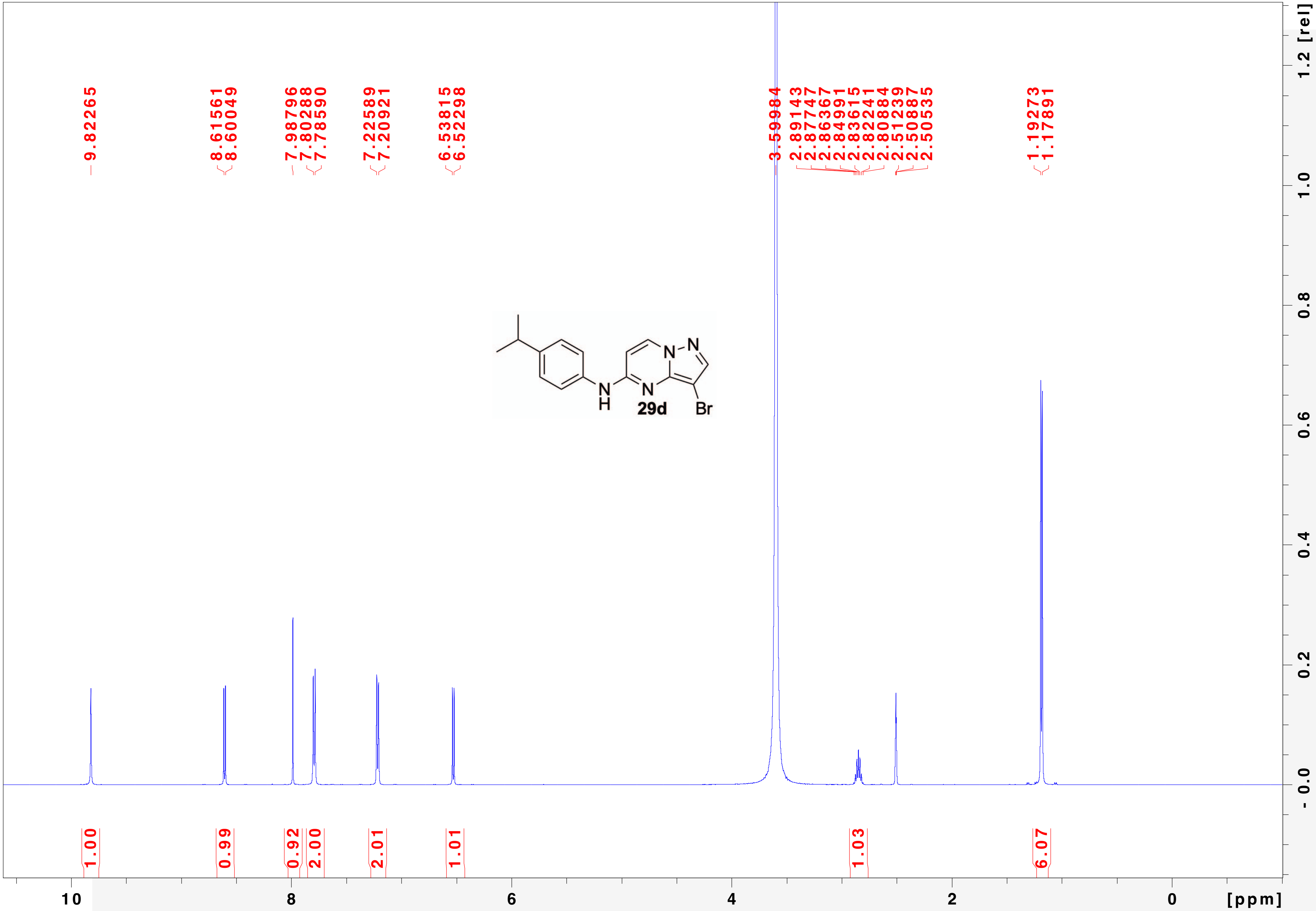

JI-III-40   10   1   /home/nmruser/networkdata/Bru500/terungwa@chem.byu.edu

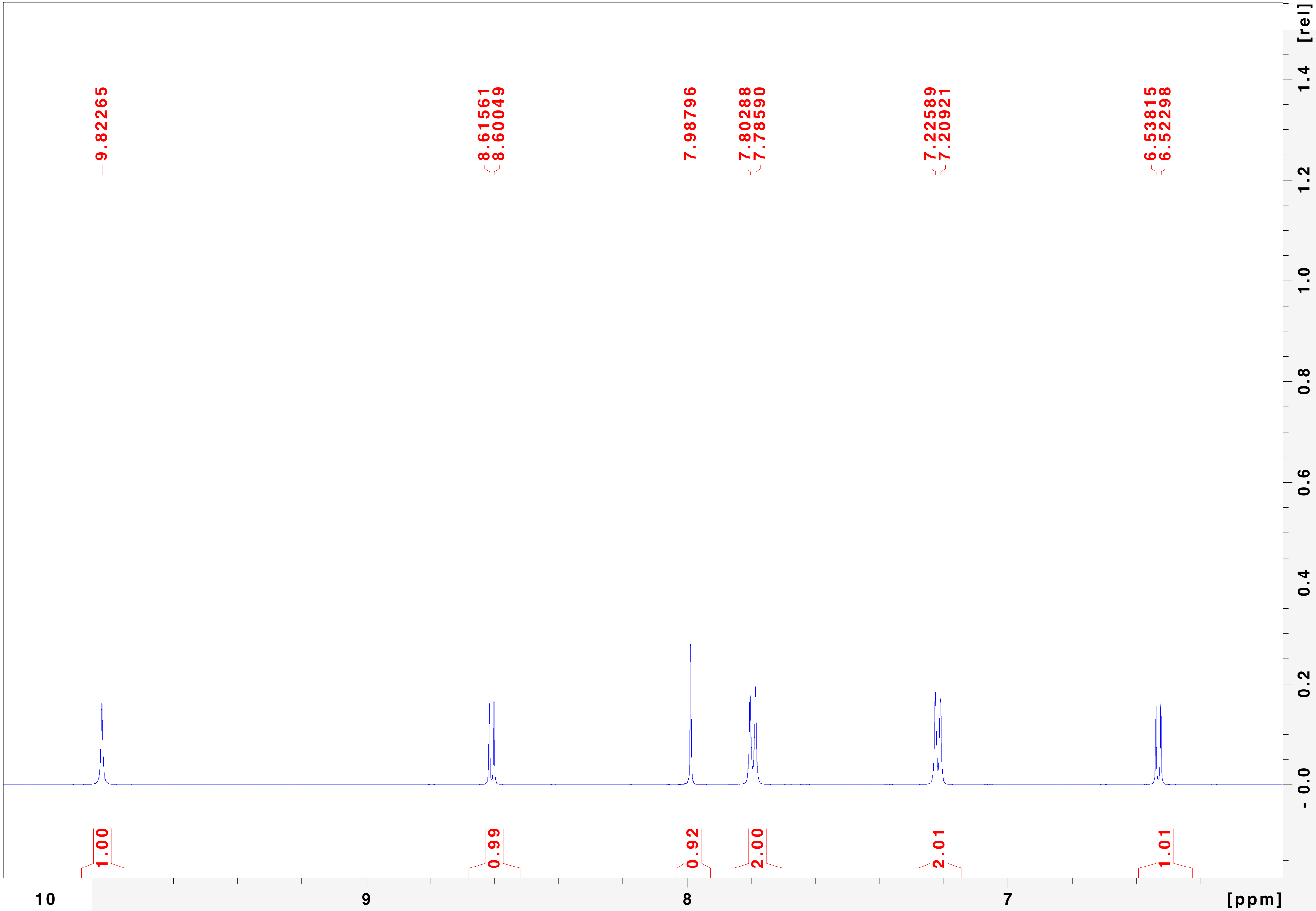

JI-III-40 10 1 /home/nmruser/networkdata/Bru500/terungwa@chem.byu.edu

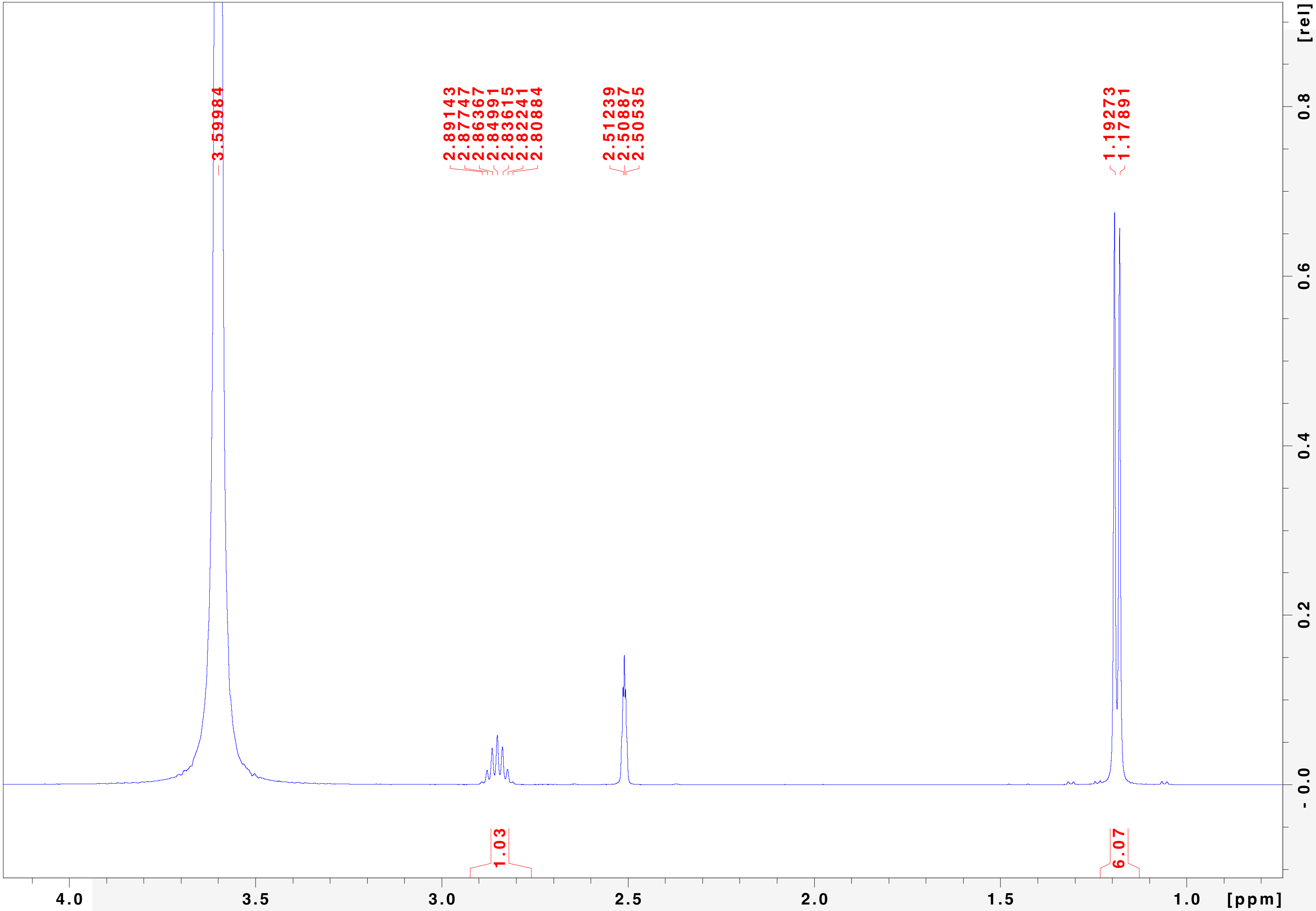

JI-III-40 20 1 /home/nmruser/networkdata/Bru500/terungwa@chem.byu.edu

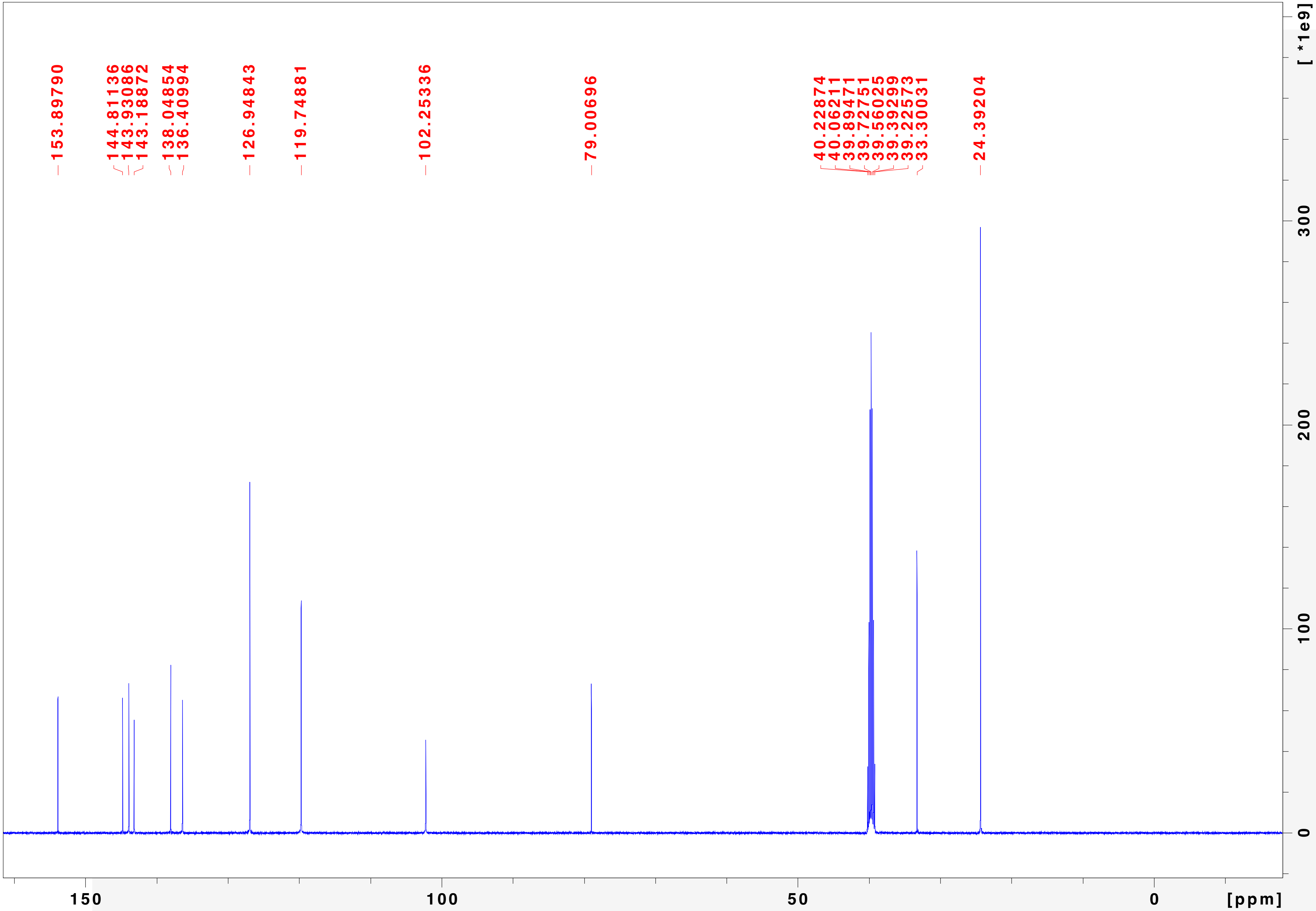

JI-III-38 10 1 /home/nmruser/networkdata/Bru500/terungwa@chem.byu.edu

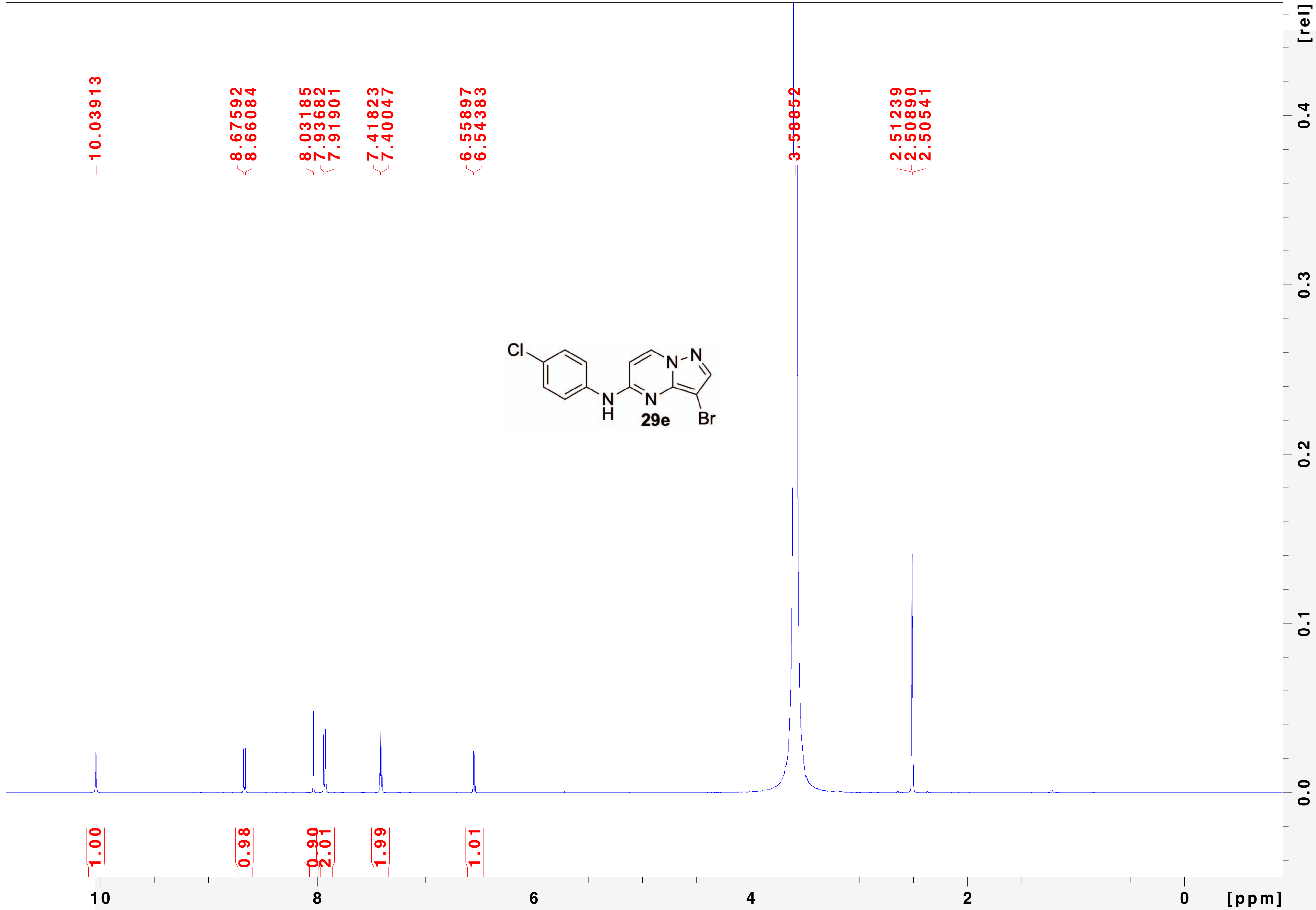

JI-III-38 10 1 /home/nmruser/networkdata/Bru500/terungwa@chem.byu.edu

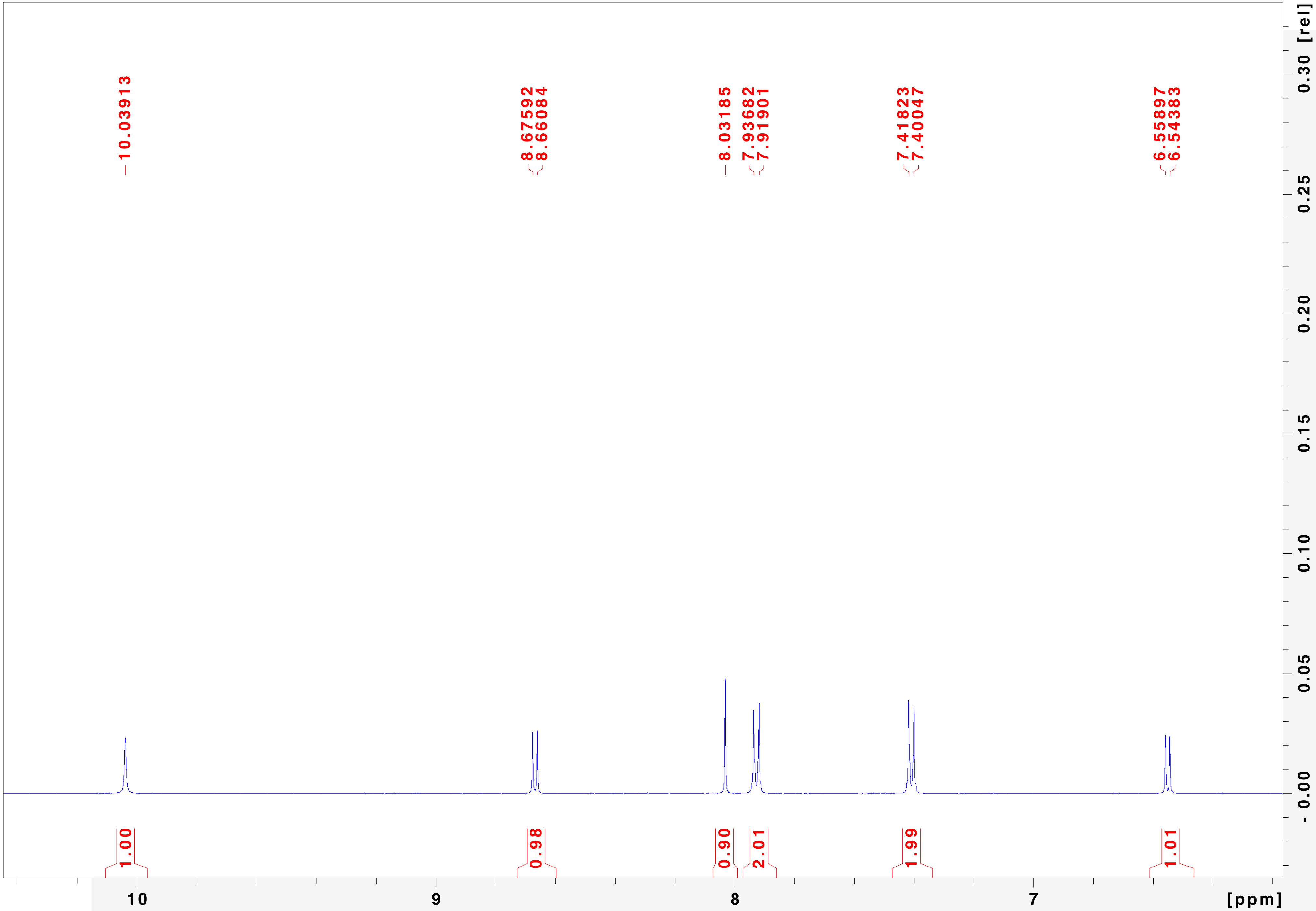

JI-III-38 20 1 /home/nmruser/networkdata/Bru500/terungwa@chem.byu.edu

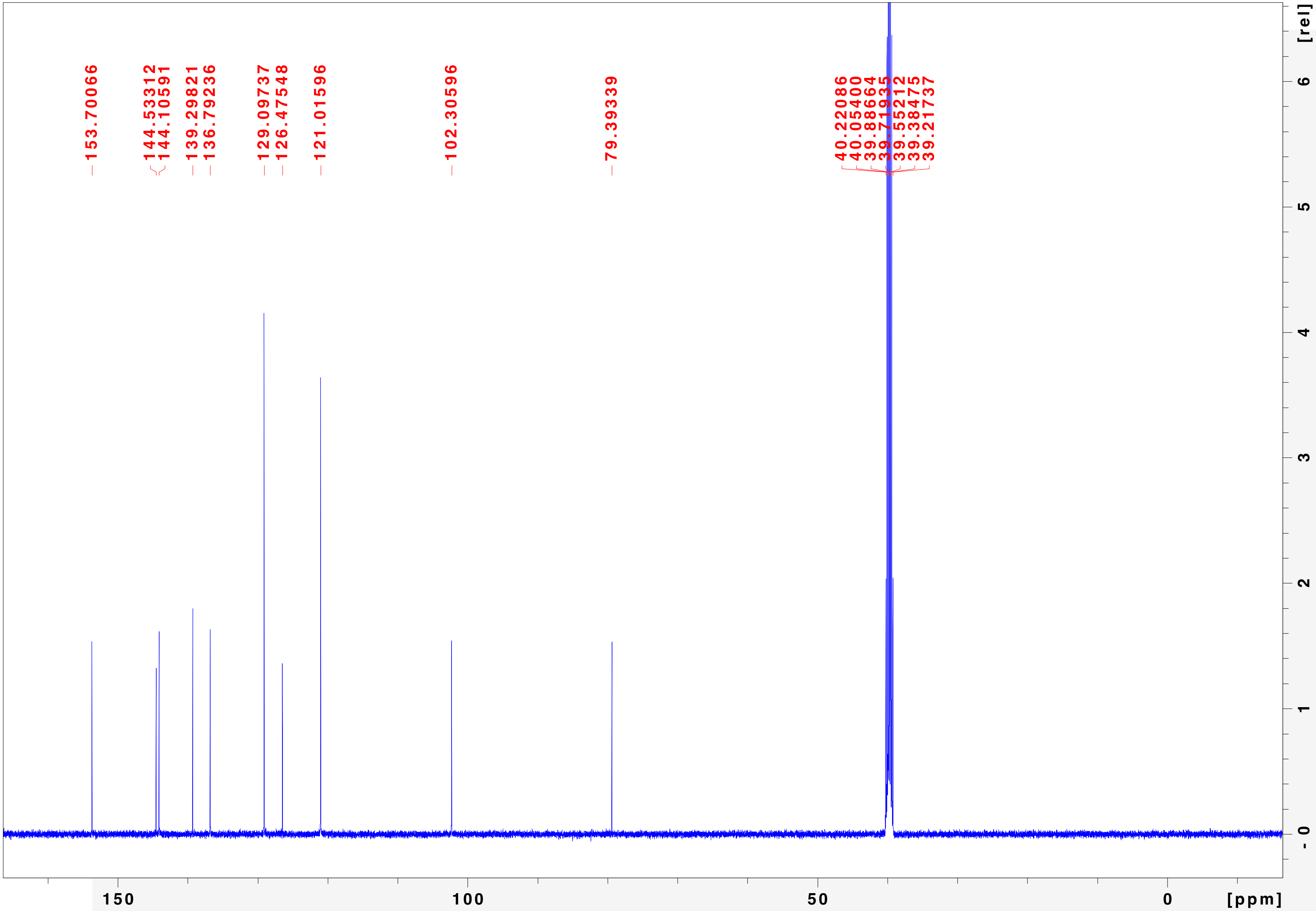

JI-II-120 20 1 /home/nmruser/networkdata/terungwa@chem.byu.edu

PROTON DMSO /opt/nmrdata terungwa@chem.byu.edu 1

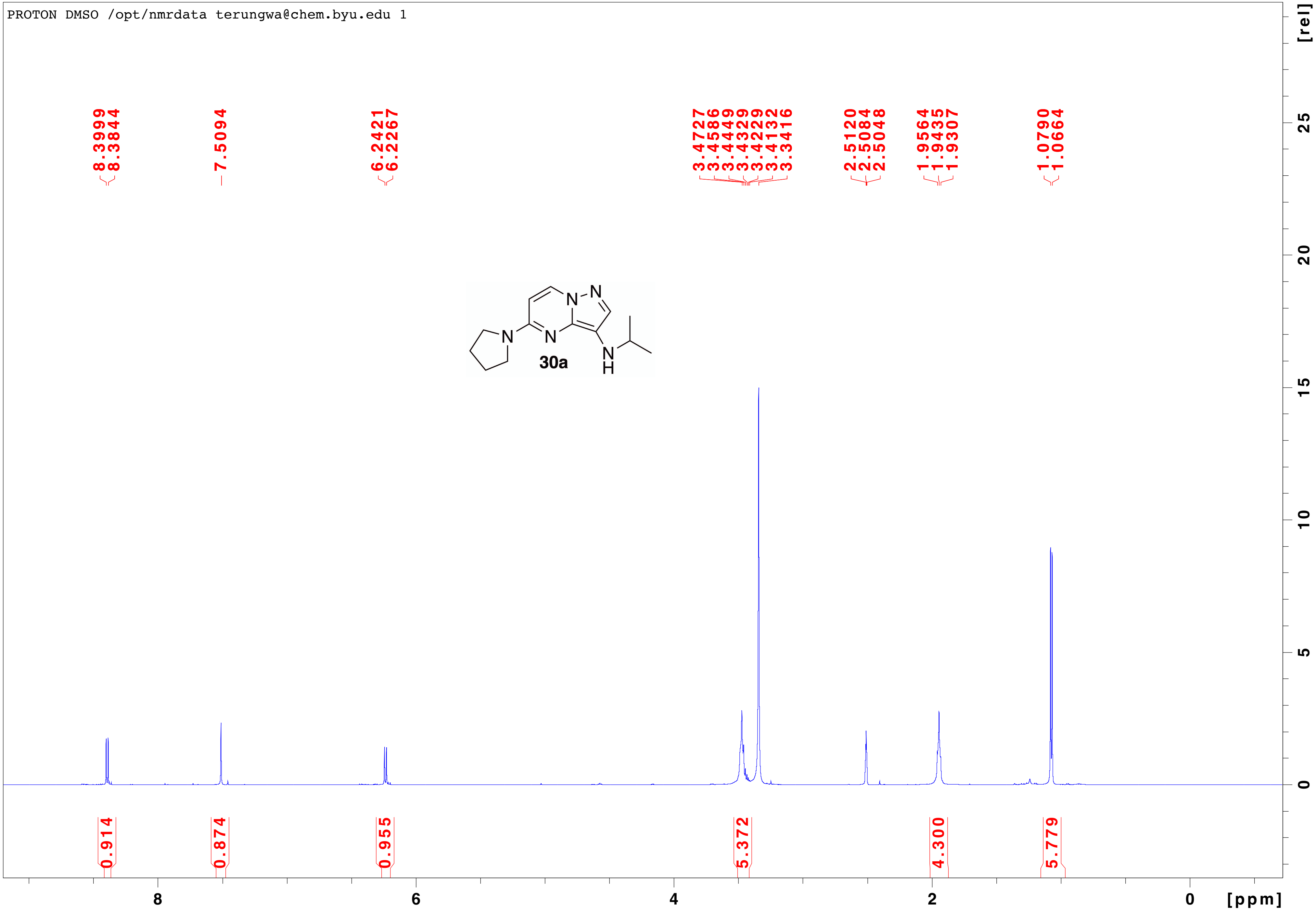

JI-II-120 20 1 /home/nmruser/networkdata/terungwa@chem.byu.edu

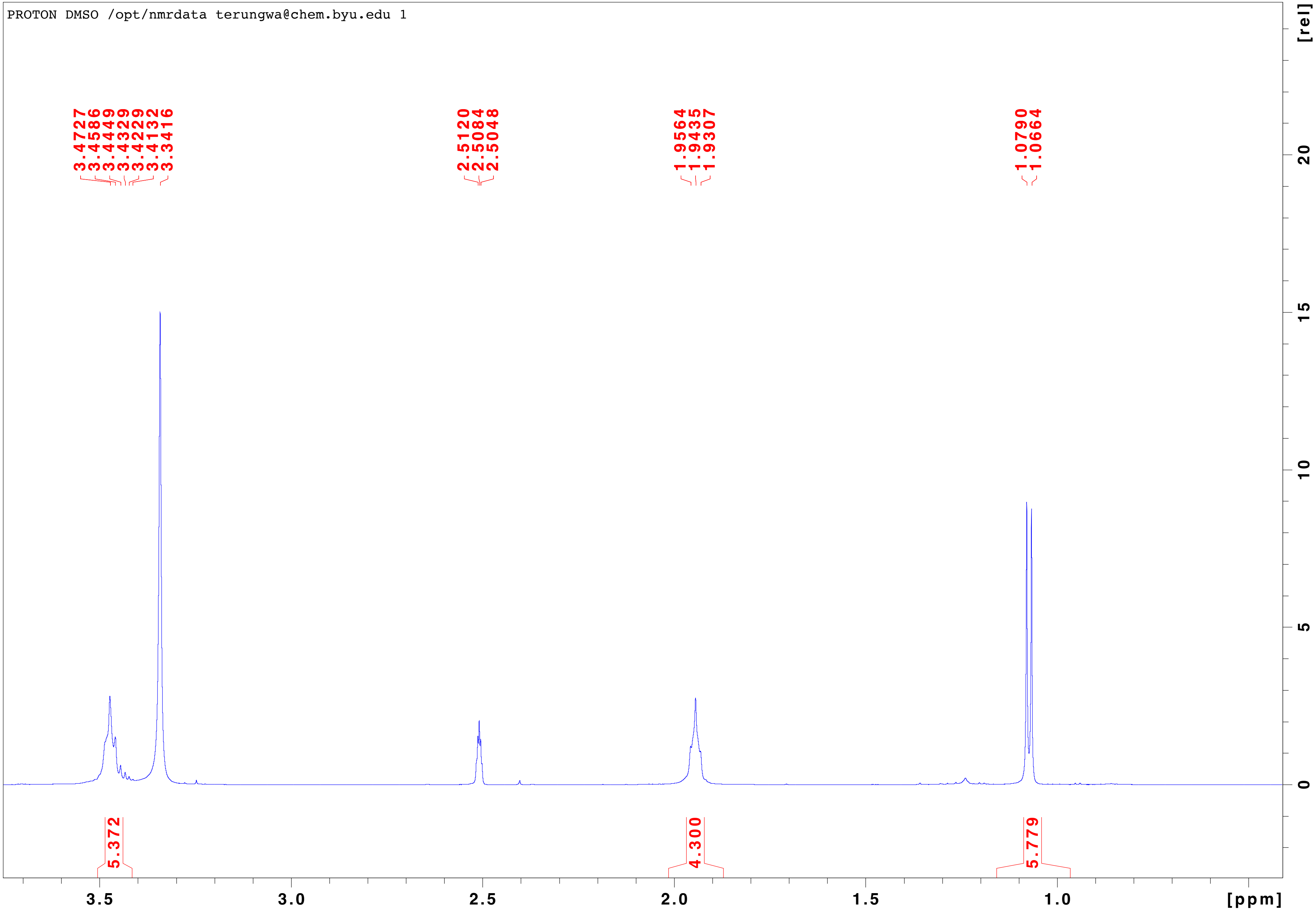

JI-II-120 20 1 /home/nmruser/networkdata/terungwa@chem.byu.edu

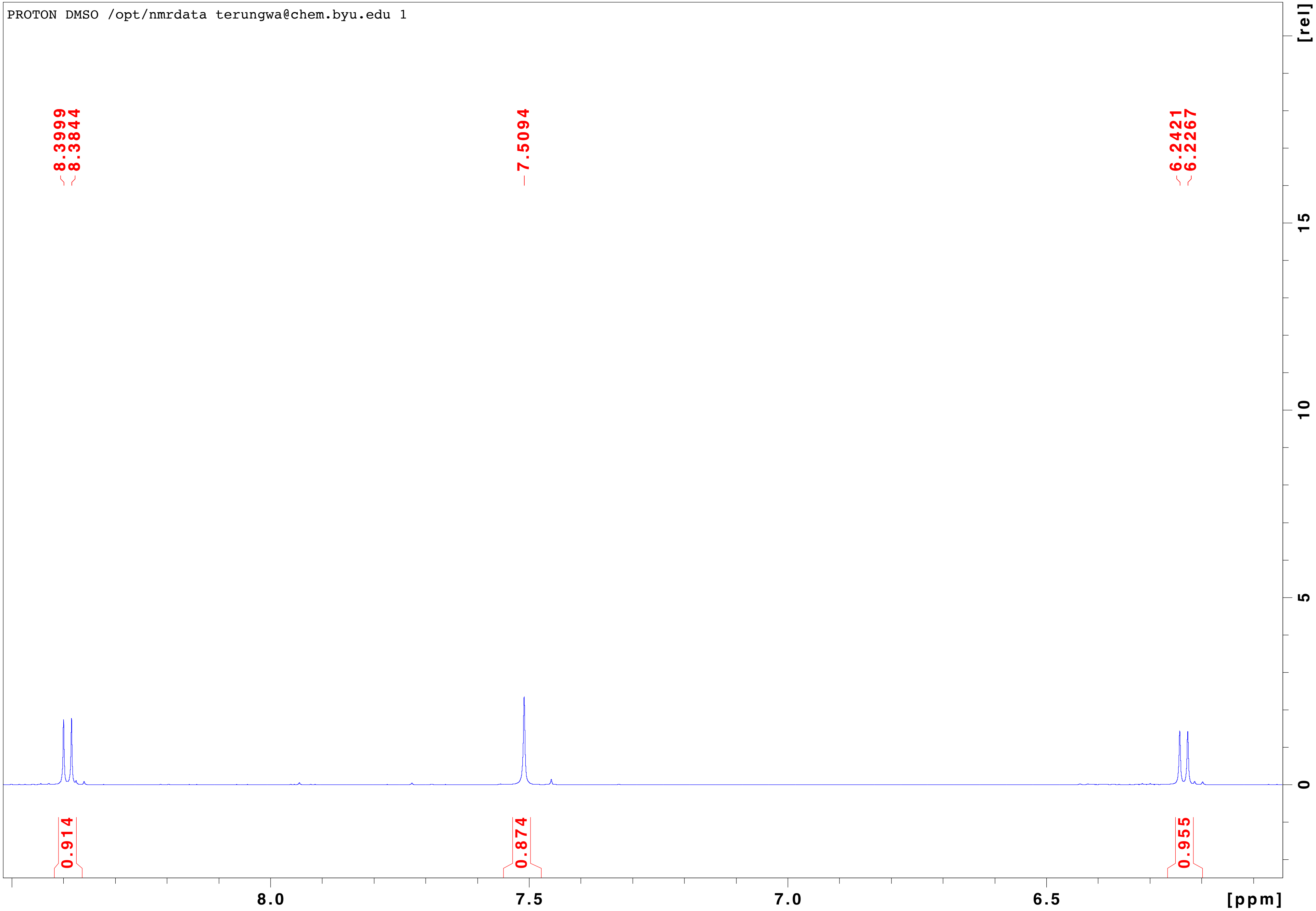

JI-II-120 40 1 /home/nmruser/networkdata/terungwa@chem.byu.edu

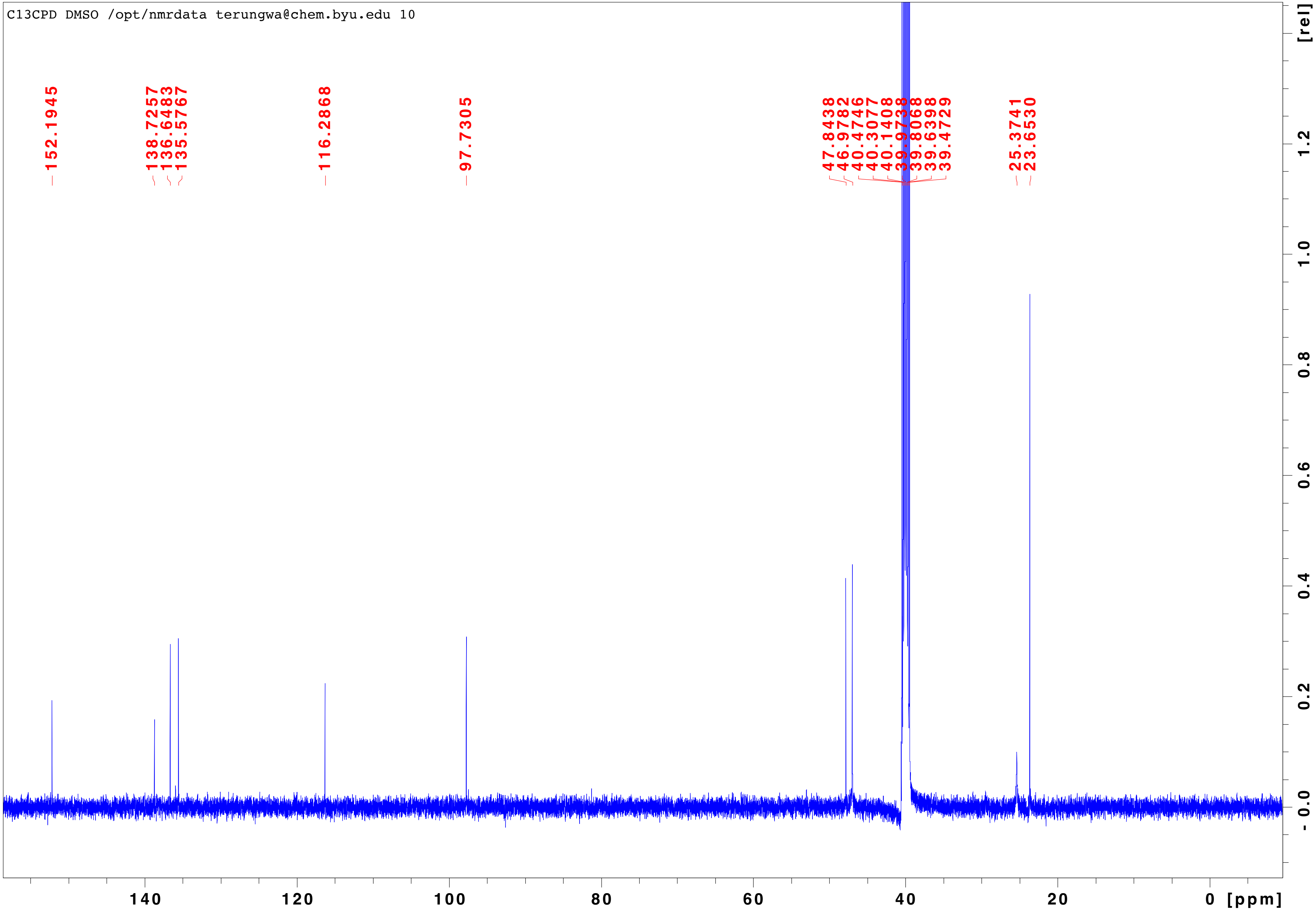

JI-II-132 10 1 /home/nmruser/networkdata/terungwa@chem.byu.edu

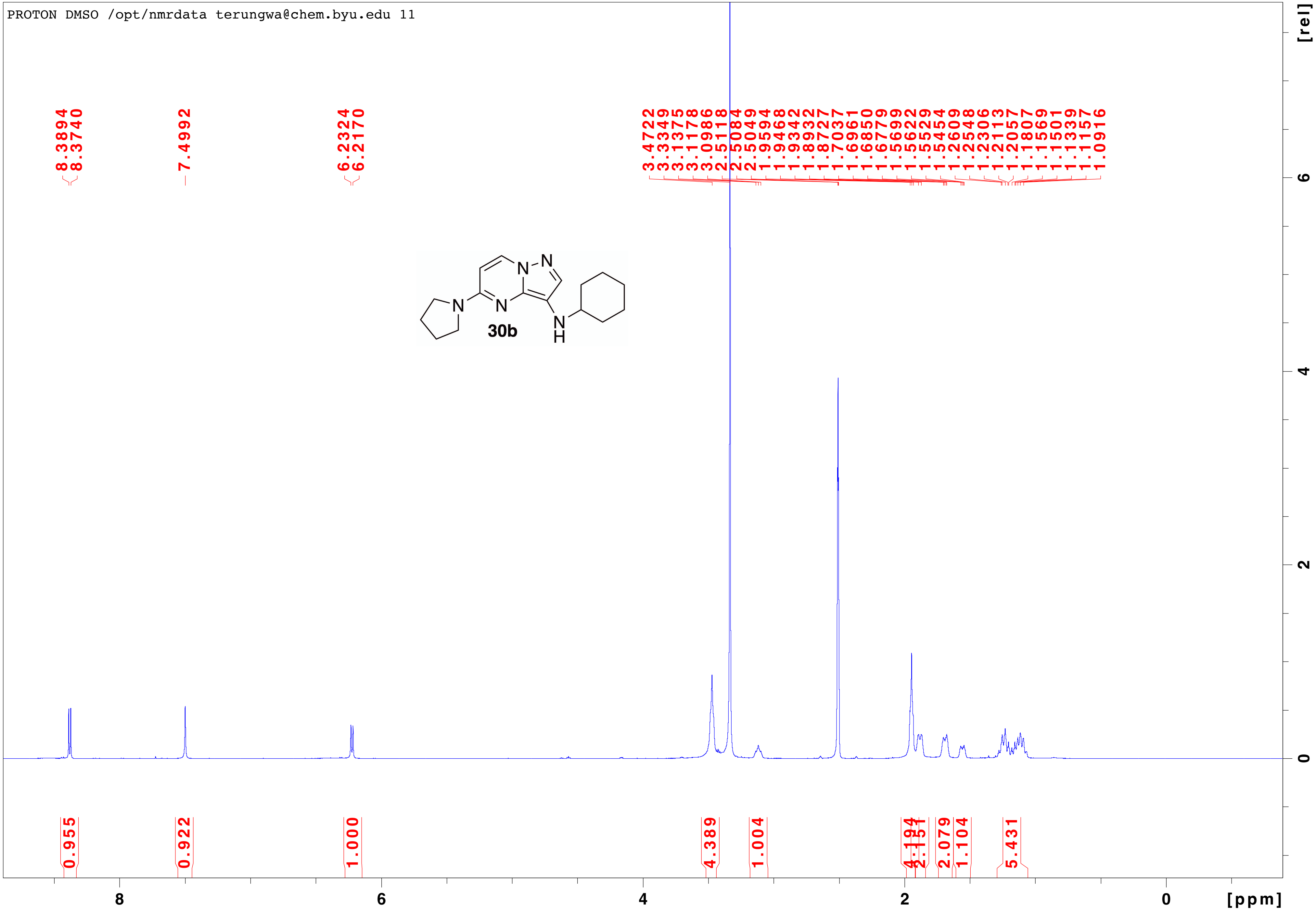

JI-II-132 10 1 /home/nmruser/networkdata/terungwa@chem.byu.edu

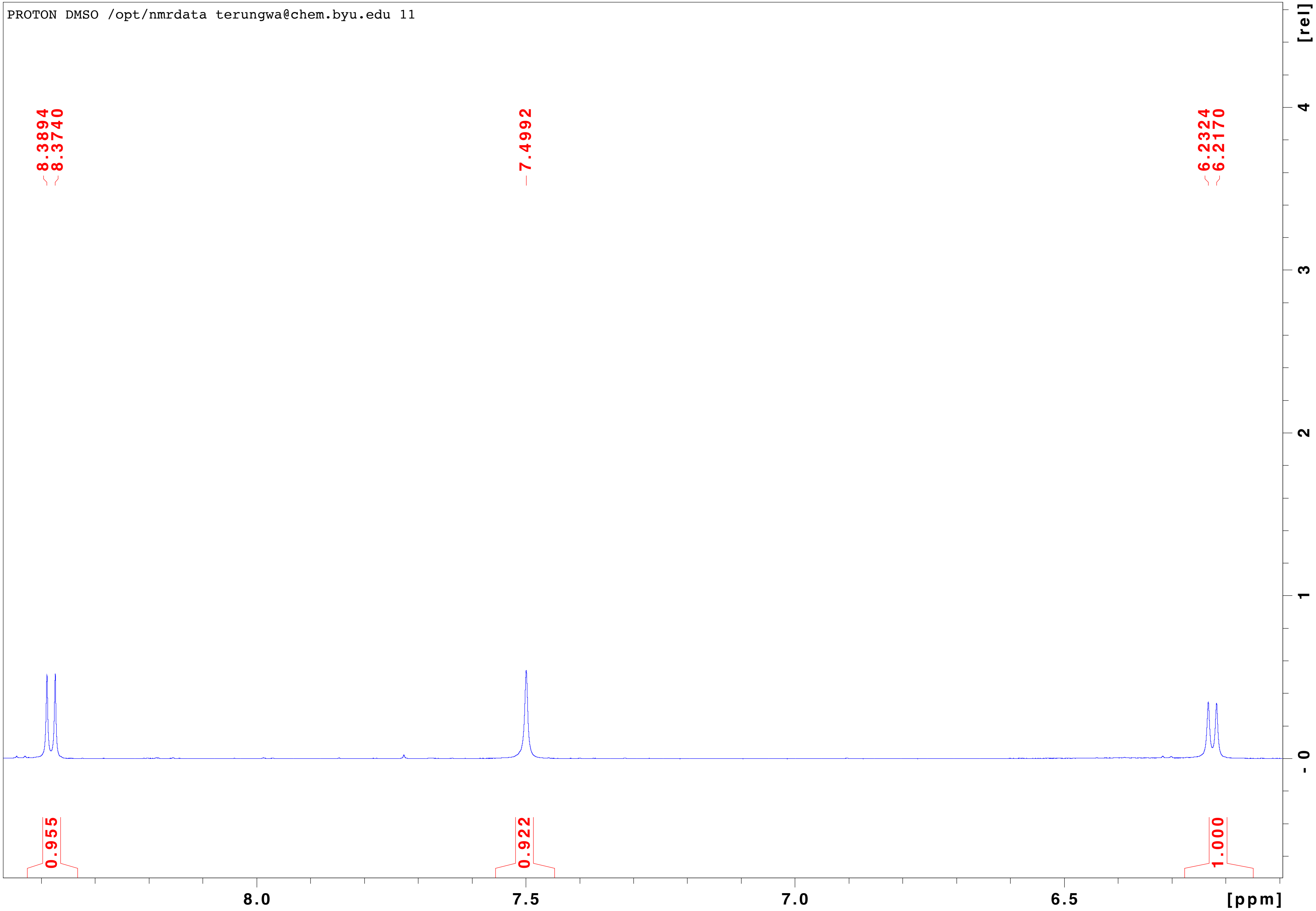

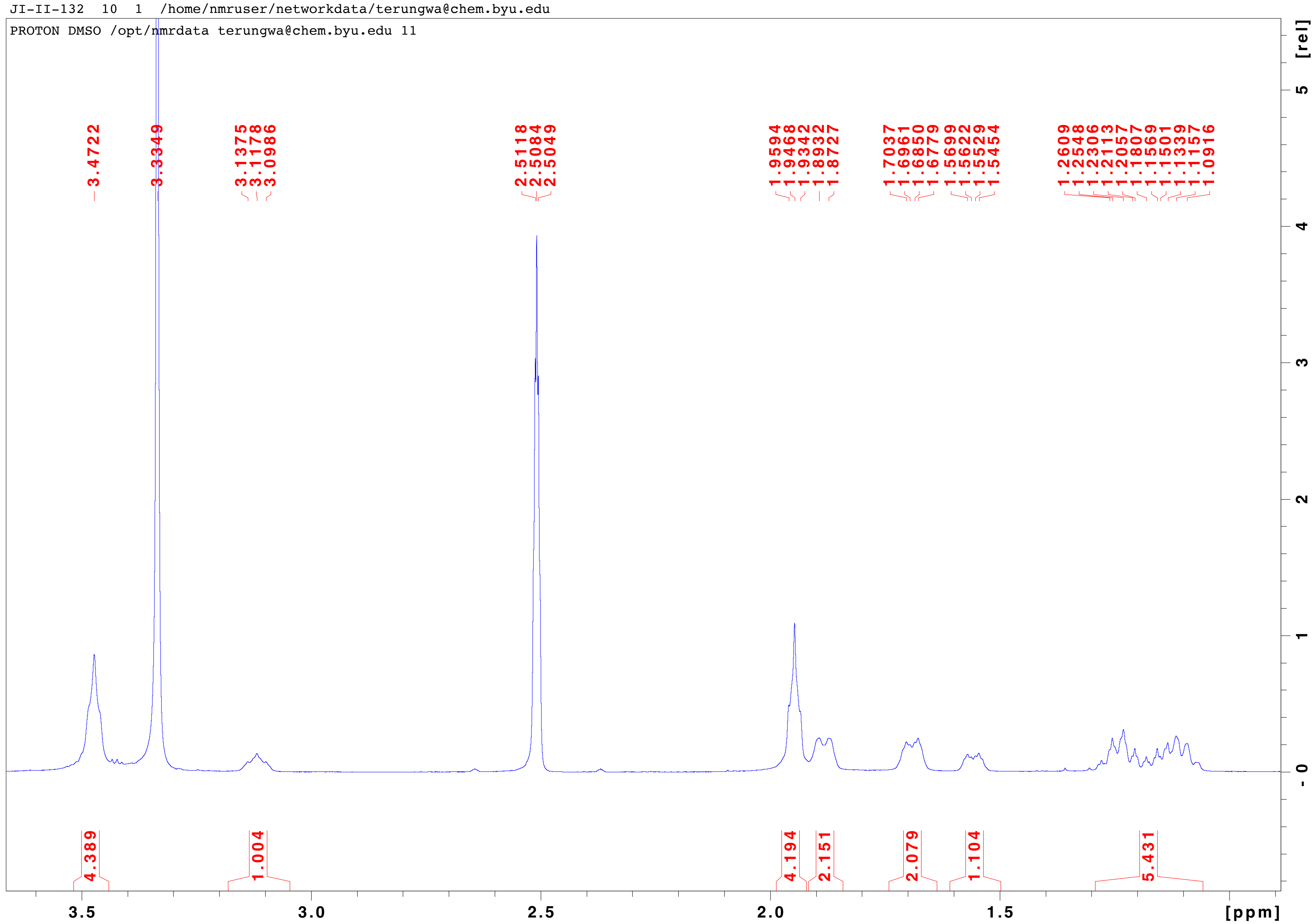
JI-II-132 10 1 /home/nmruser/networkdata/terungwa@chem.byu.edu
PROTON DMSO /opt/nmrdata terungwa@chem.byu.edu 11
3.4722
3.3349
3.1375
3.1178
3.0986
2.5118
2.5084
2.5049
1.9594
1.9468
1.9342
1.8932
1.8727
1.7037
1.6961
1.6850
1.6779
1.5699
1.5622
1.5529
1.5454
1.2609
1.2548
1.2306
1.2113
1.2057
1.1807
1.1569
1.1501
1.1339
1.1157
1.0916
4.389
1.004
4.194
2.151
2.079
1.104
5.431
[rel]
[ppm]

JI-II-132 20 1 /home/nmruser/networkdata/terungwa@chem.byu.edu

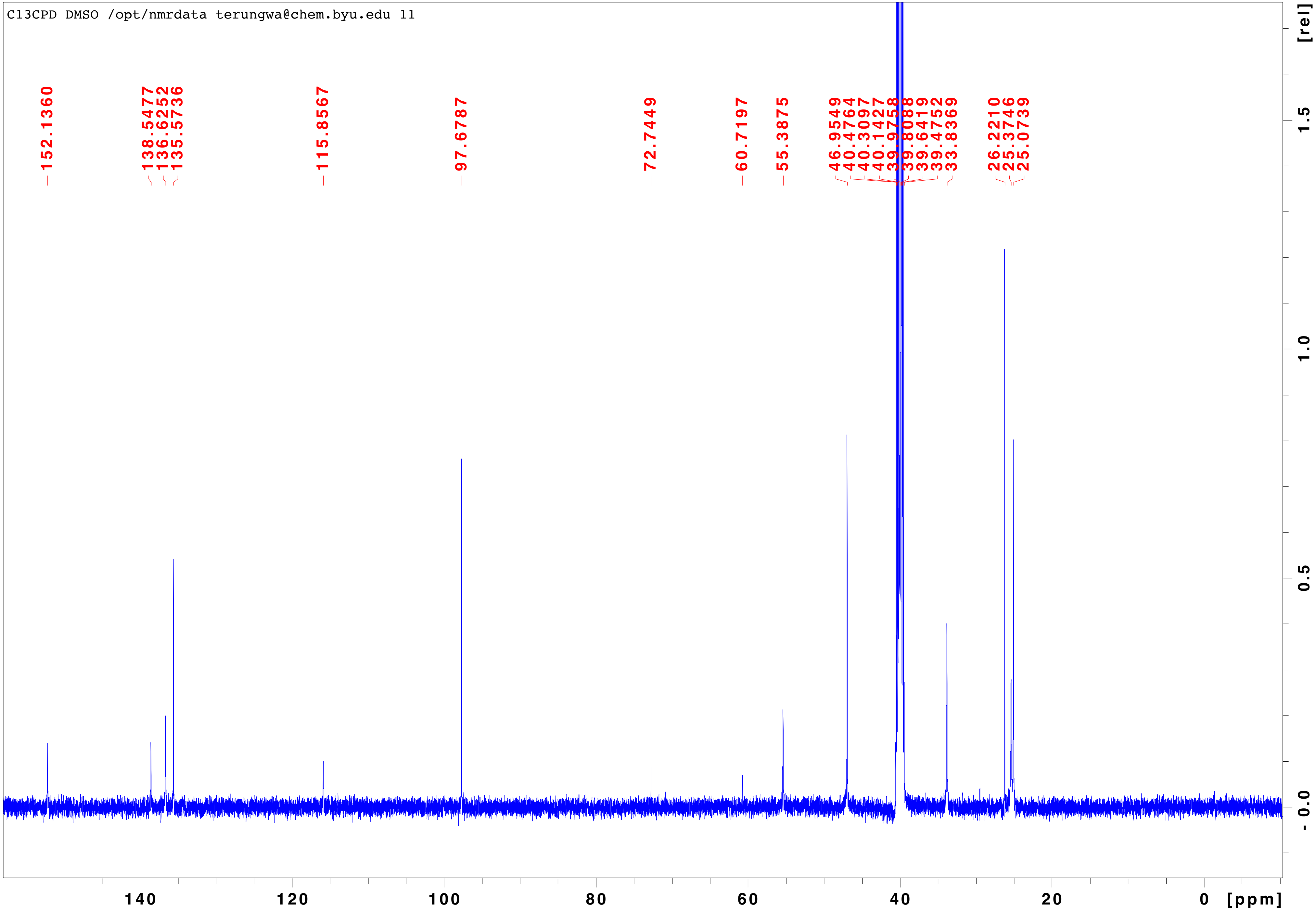

LG-I-46 30 1 /home/nmruser/networkdata/Bruker500Data/log96@chem.byu.edu

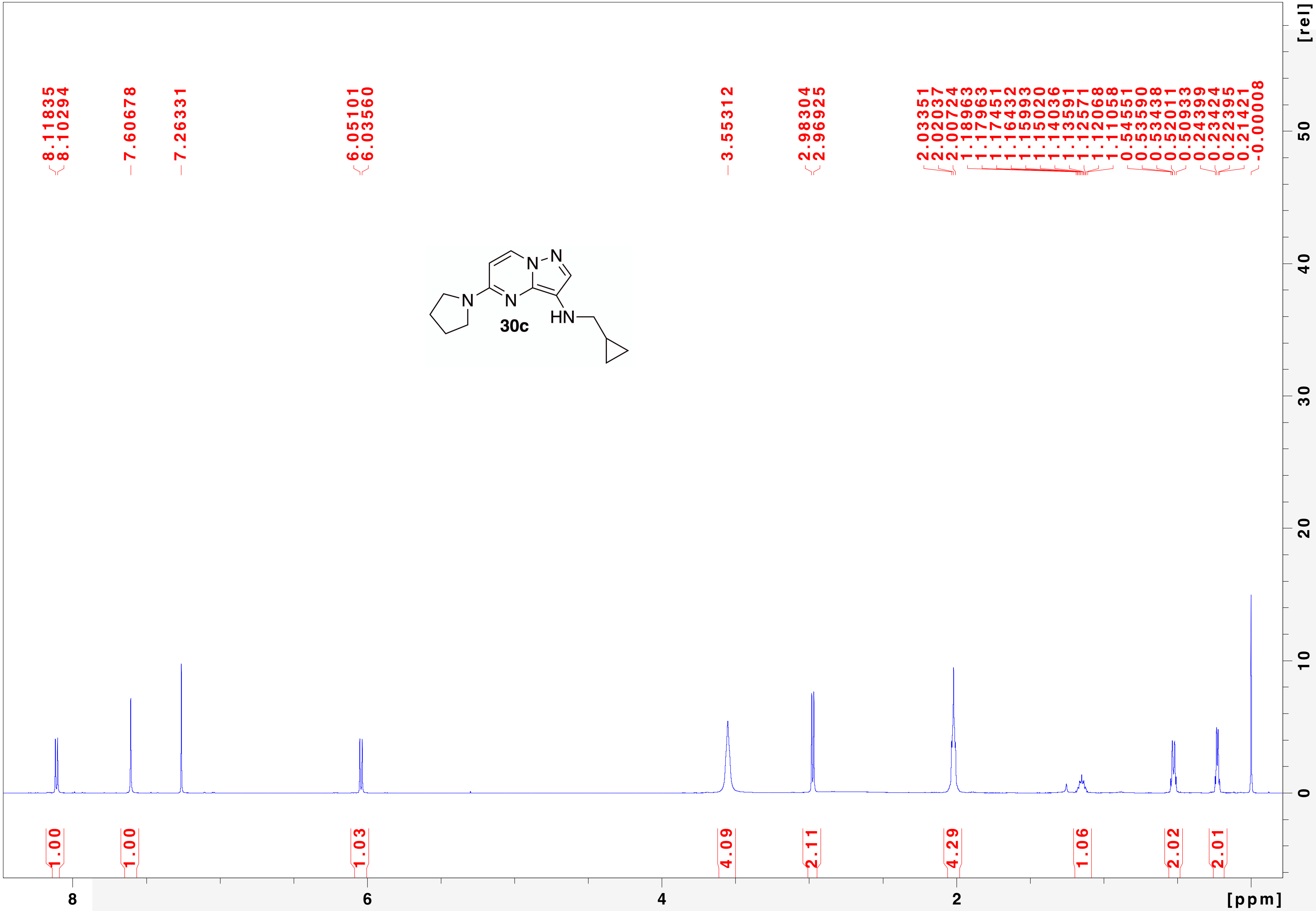

LG-I-46   30   1   /home/nmruser/networkdata/Bruker500Data/log96@chem.byu.edu

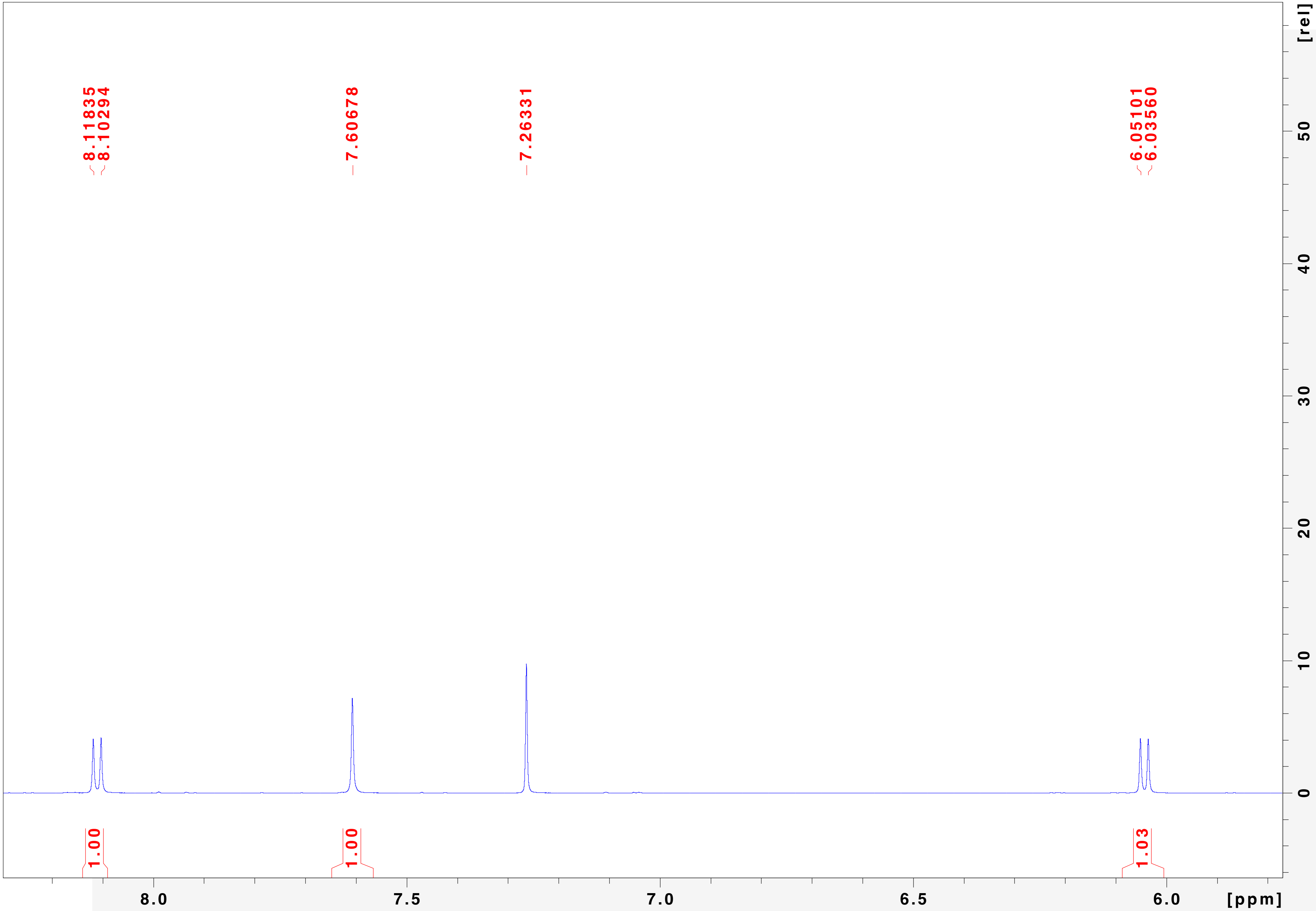

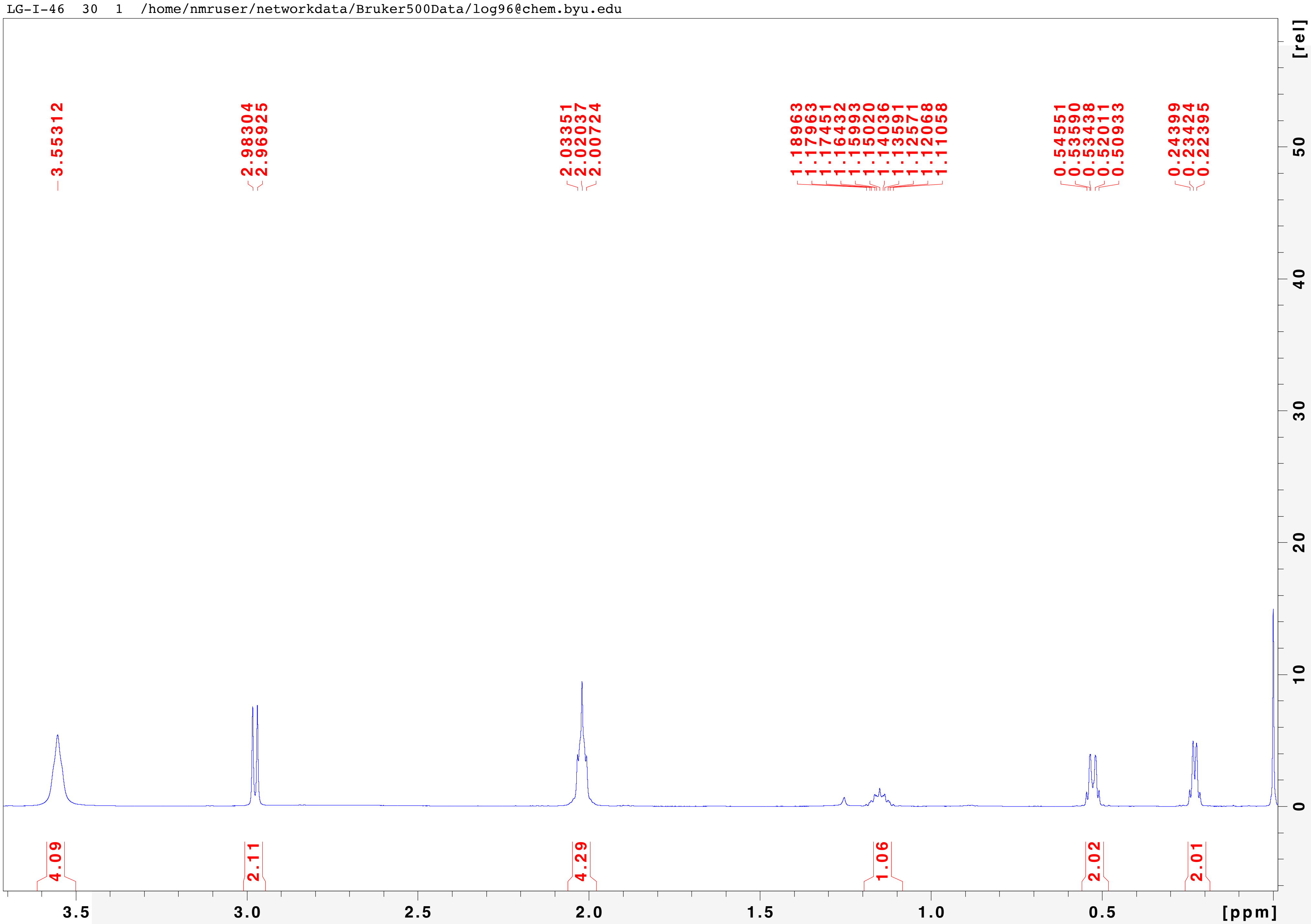

LG-I-46 30 1 /home/nmruser/networkdata/Bruker500Data/log96@chem.byu.edu
3.55312
2.98304
2.96925
2.03351
2.02037
2.00724
1.18963
1.17963
1.17451
1.16432
1.15993
1.15020
1.14036
1.13591
1.12571
1.12068
1.11058
0.54551
0.53590
0.53438
0.52011
0.50933
0.24399
0.23424
0.22395
4.09
2.11
4.29
1.06
2.02
2.01
[rel]
[ppm]

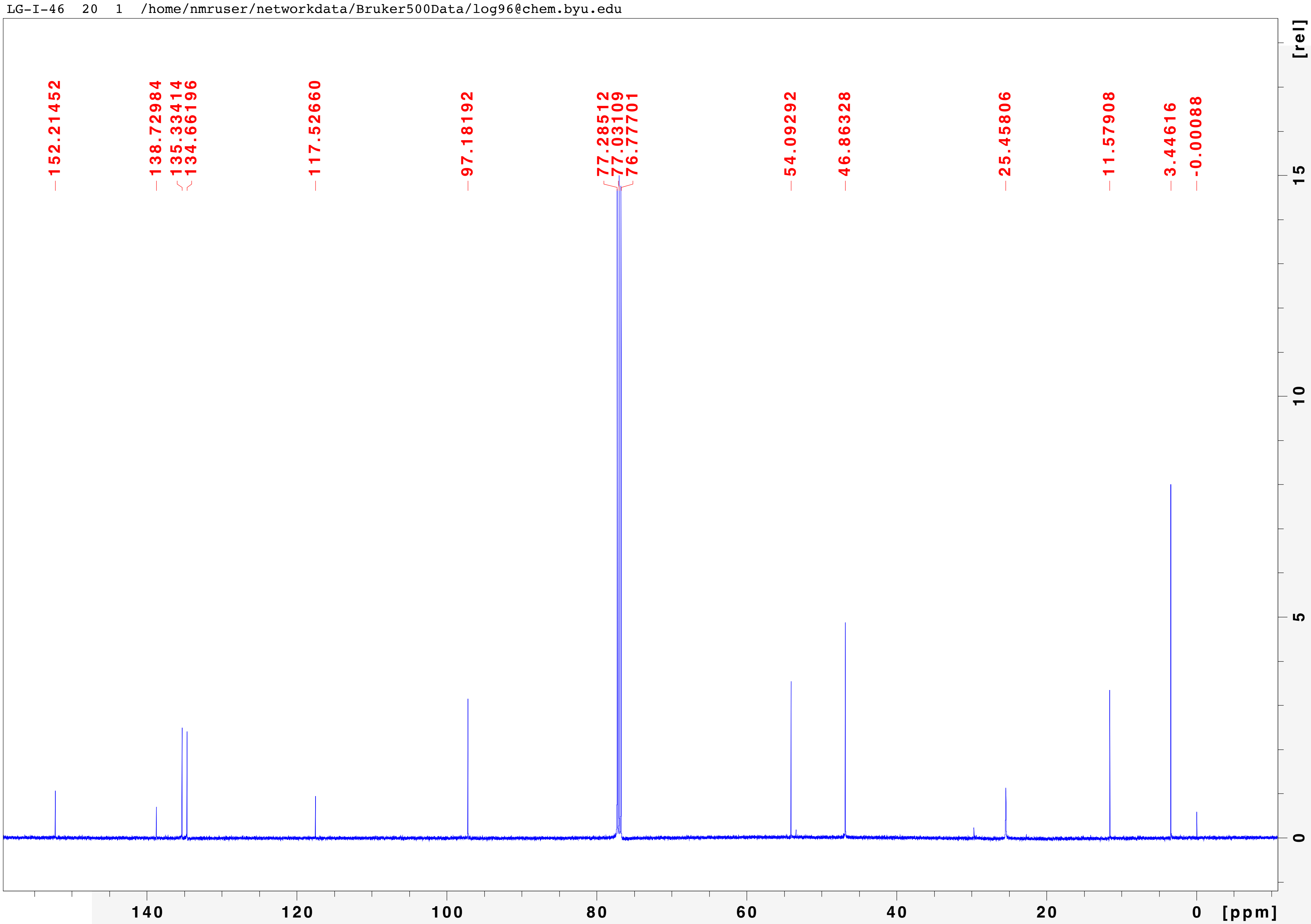
LG-I-46 20 1 /home/nmruser/networkdata/Bruker500Data/log96@chem.byu.edu
152.21452
138.72984
135.33414
134.66196
117.52660
97.18192
77.28512
77.03109
76.77701
54.09292
46.86328
25.45806
11.57908
3.44616
-0.00088
[rel]
15
10
5
0
140
120
100
80
60
40
20
0
[ppm]

JI-II-131 40 1 /home/nmruser/networkdata/terungwa@chem.byu.edu

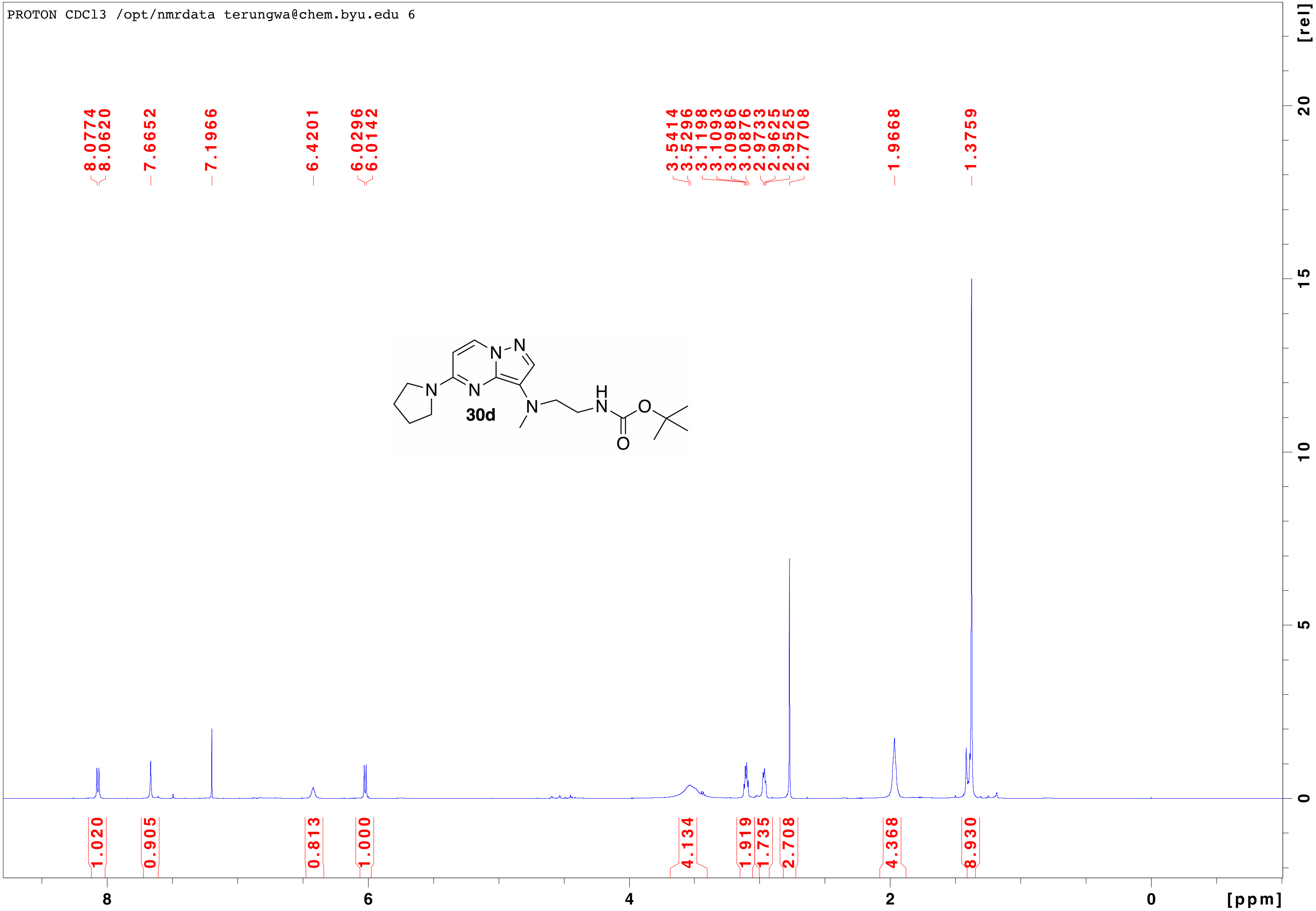

JI-II-131 40 1 /home/nmruser/networkdata/terungwa@chem.byu.edu

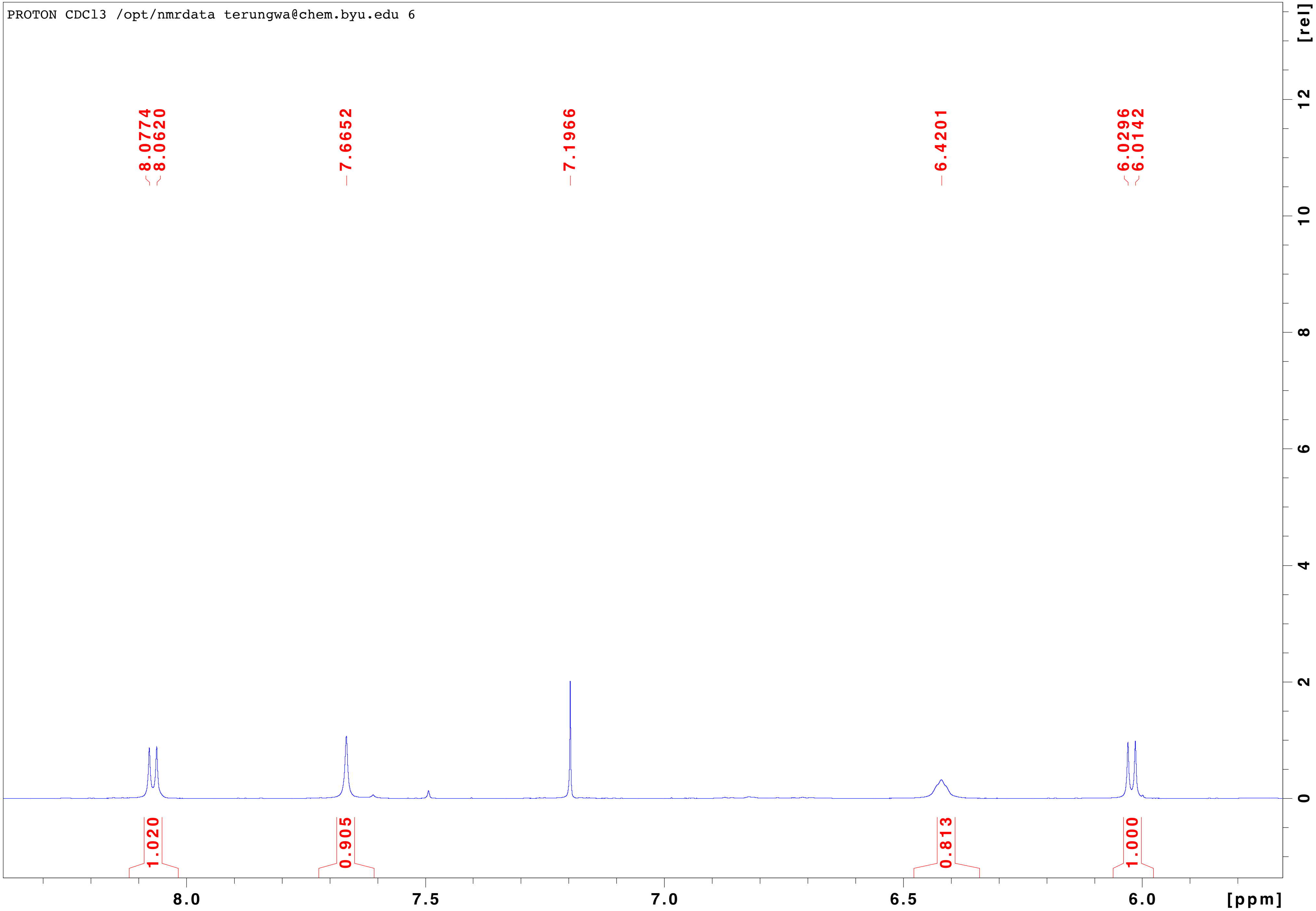

JI-II-131 40 1 /home/nmruser/networkdata/terungwa@chem.byu.edu

PROTON CDCl3 /opt/nmrdata terungwa@chem.byu.edu 6

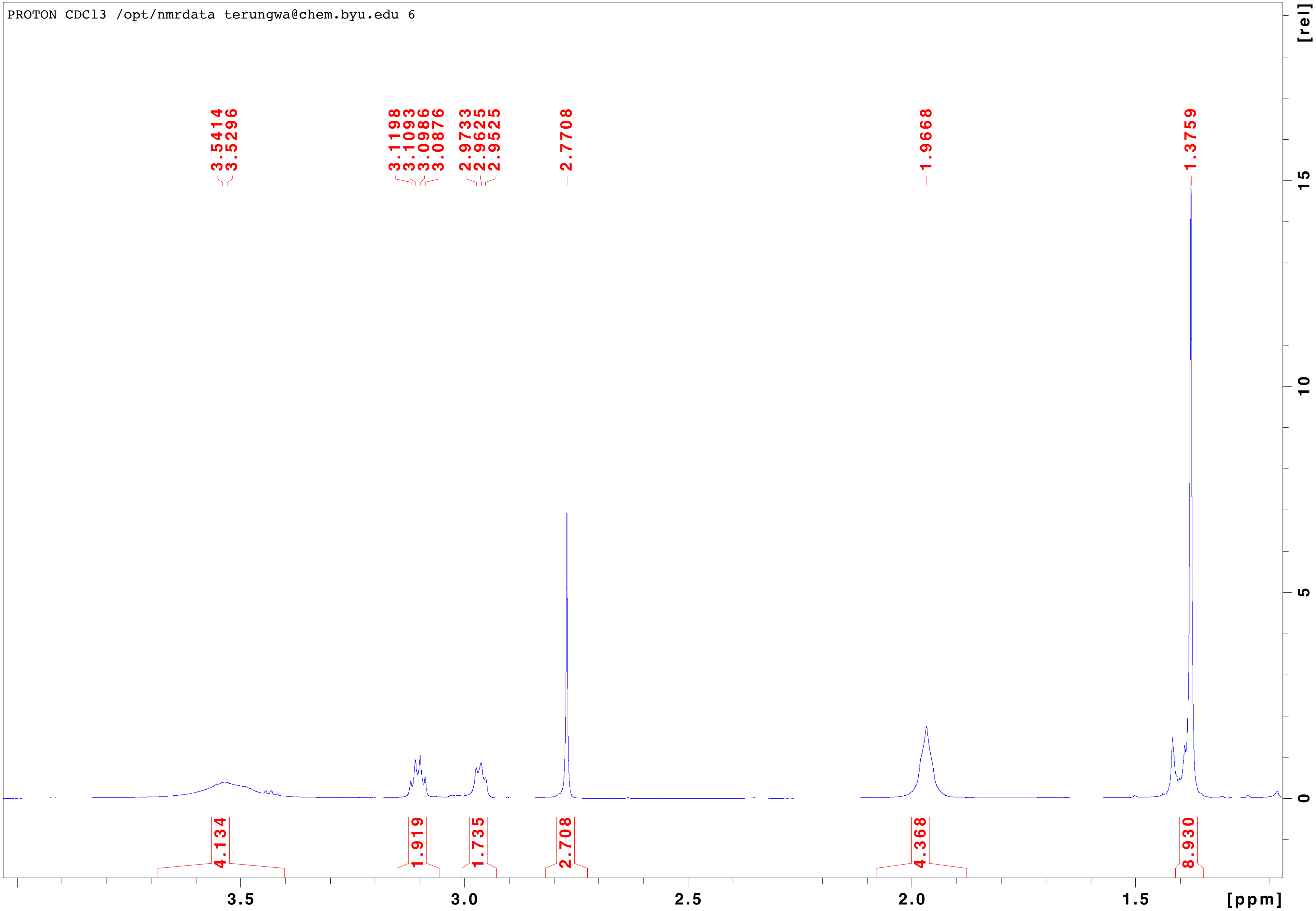

JI-II-131 50 1 /home/nmruser/networkdata/terungwa@chem.byu.edu

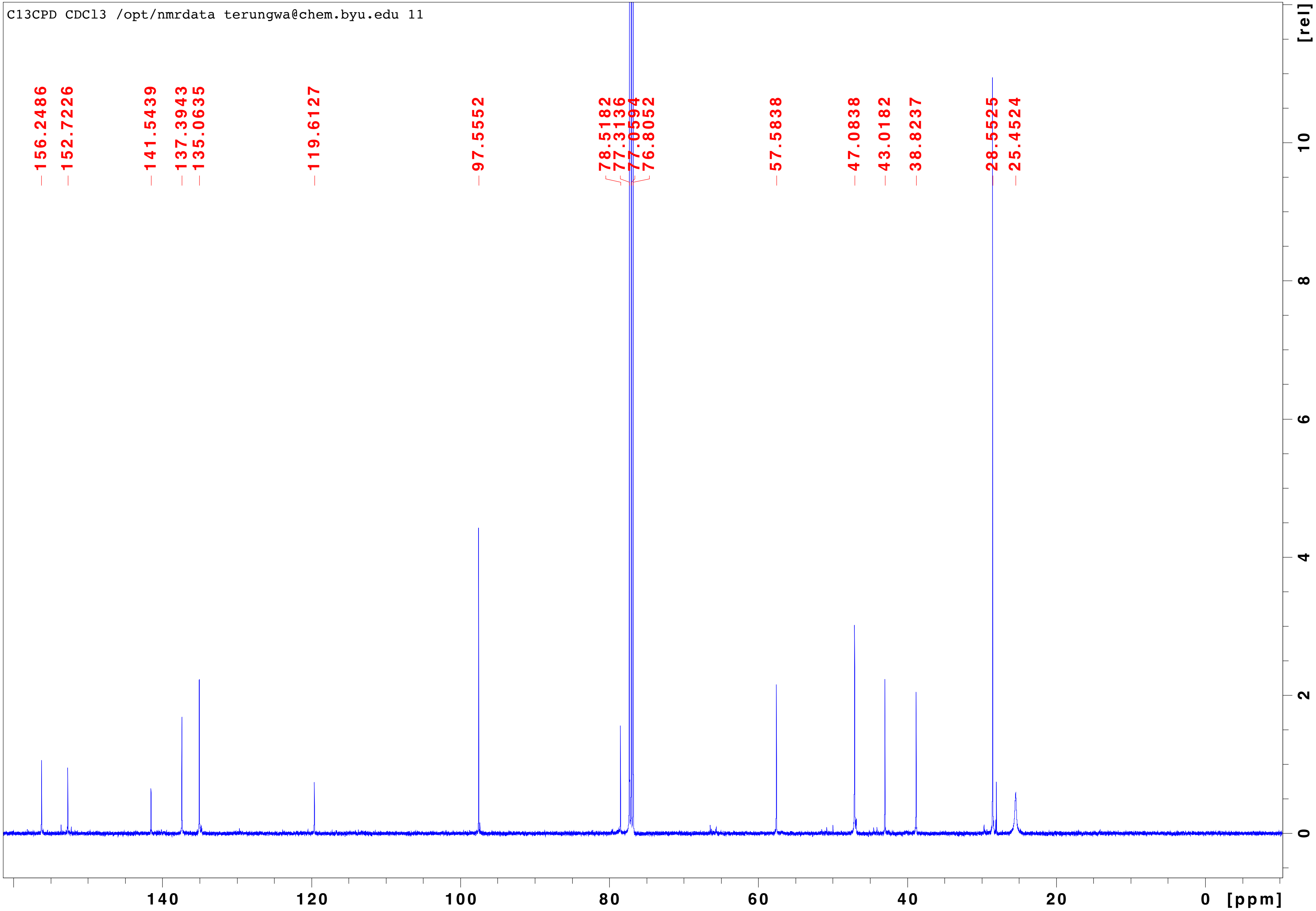

JI-II-49 40 1 /home/nmruser/networkdata/terungwa@chem.byu.edu

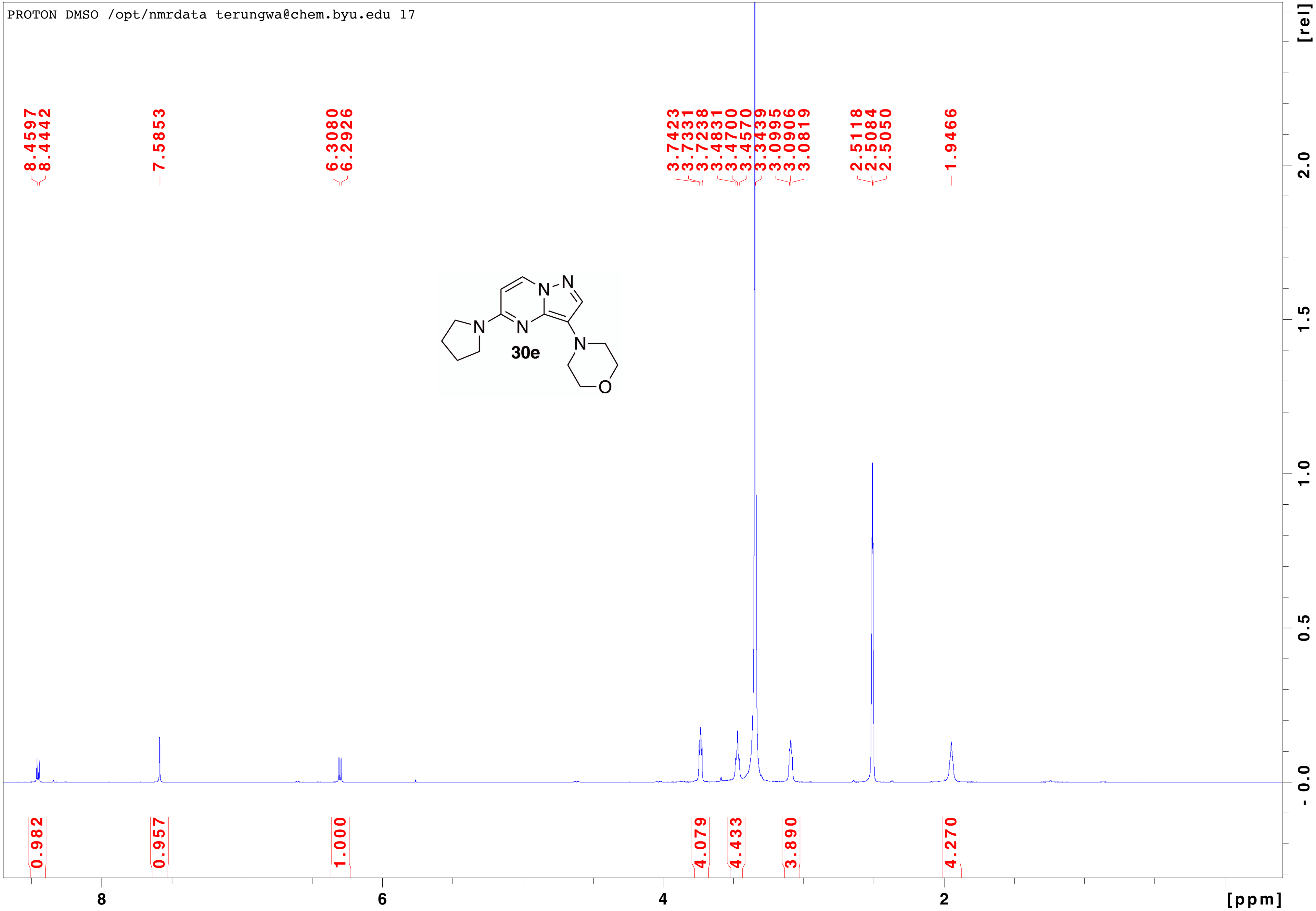

JI-II-49 40 1 /home/nmruser/networkdata/terungwa@chem.byu.edu

PROTON DMSO /opt/nmrdata terungwa@chem.byu.edu 17

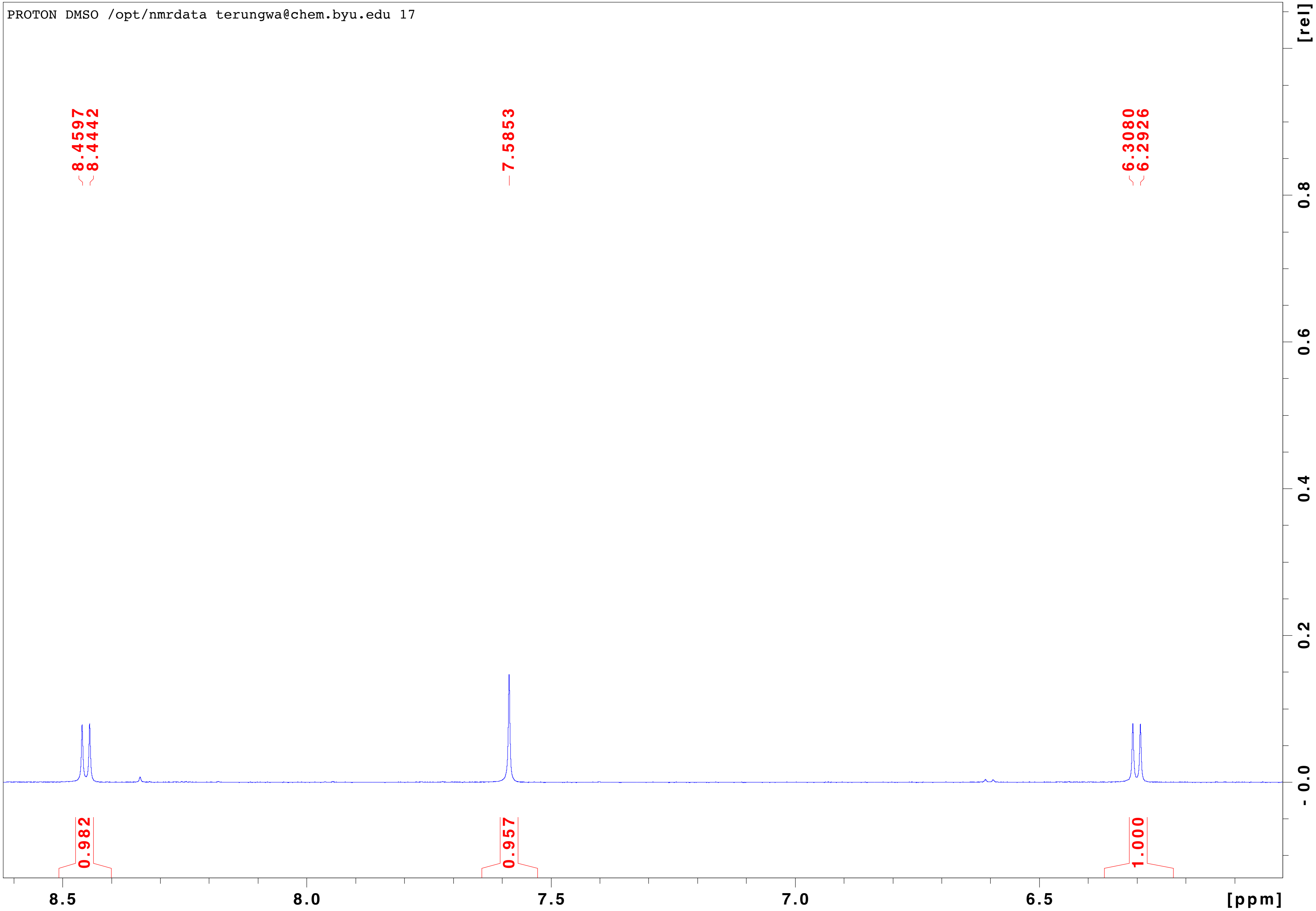

JI-II-49 40 1 /home/nmruser/networkdata/terungwa@chem.byu.edu

PROTON DMSO /opt/nmrdata terungwa@chem.byu.edu 17

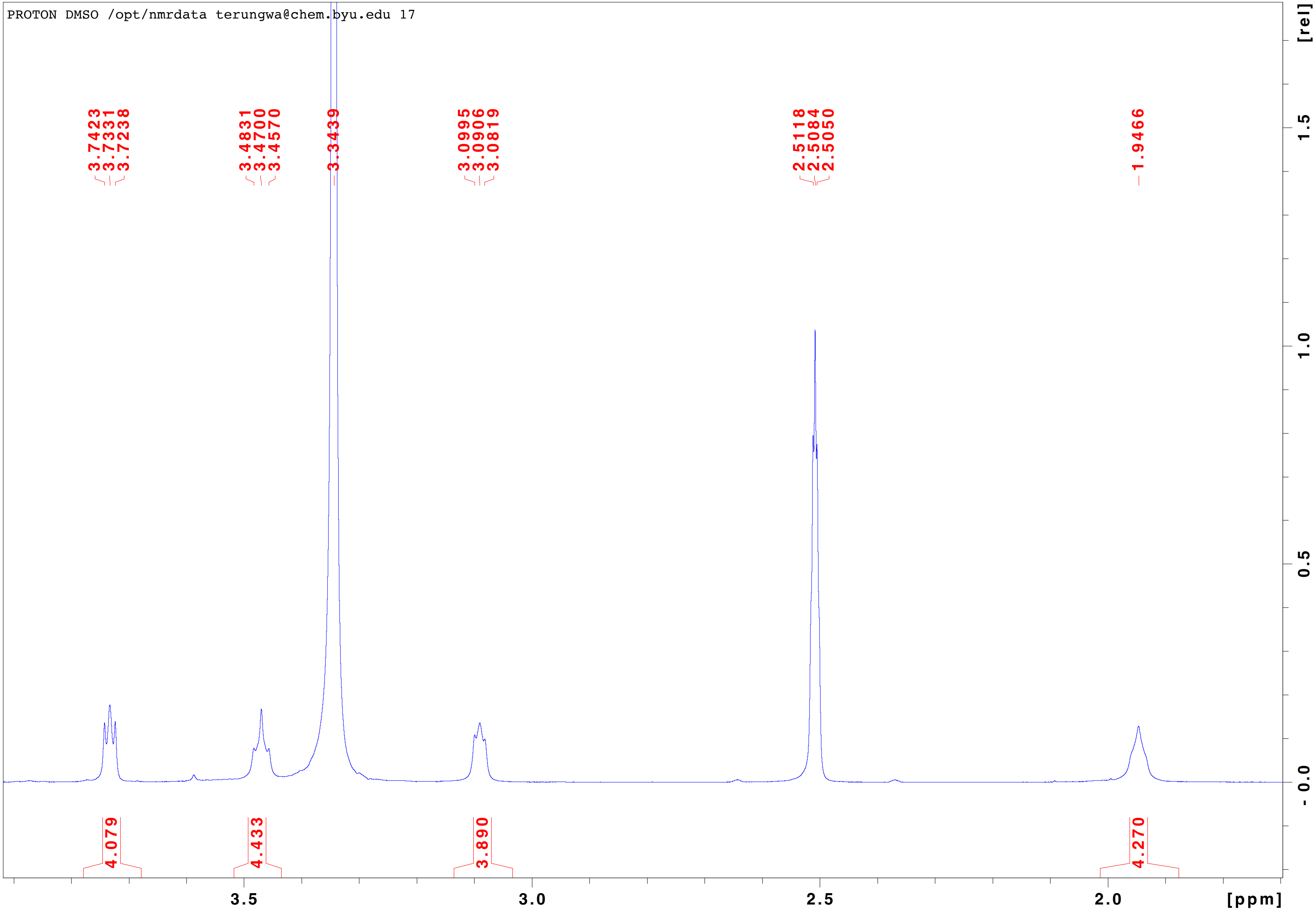

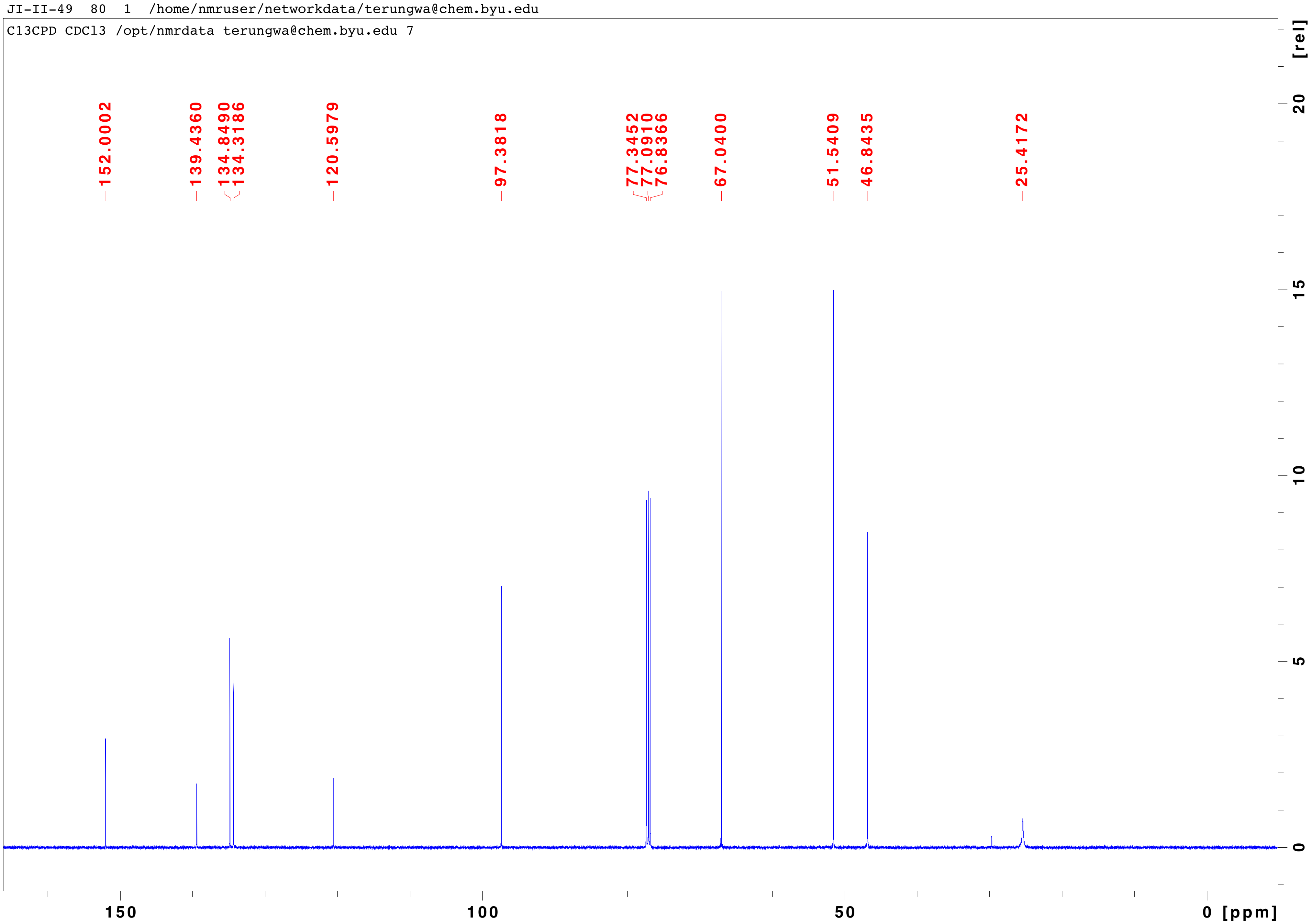
JI-II-49 80 1 /home/nmruser/networkdata/terungwa@chem.byu.edu
C13CPD CDCl3 /opt/nmrdata terungwa@chem.byu.edu 7
152.0002
139.4360
134.8490
134.3186
120.5979
97.3818
77.3452
77.0910
76.8366
67.0400
51.5409
46.8435
25.4172
[rel]
150
100
50
0 [ppm]

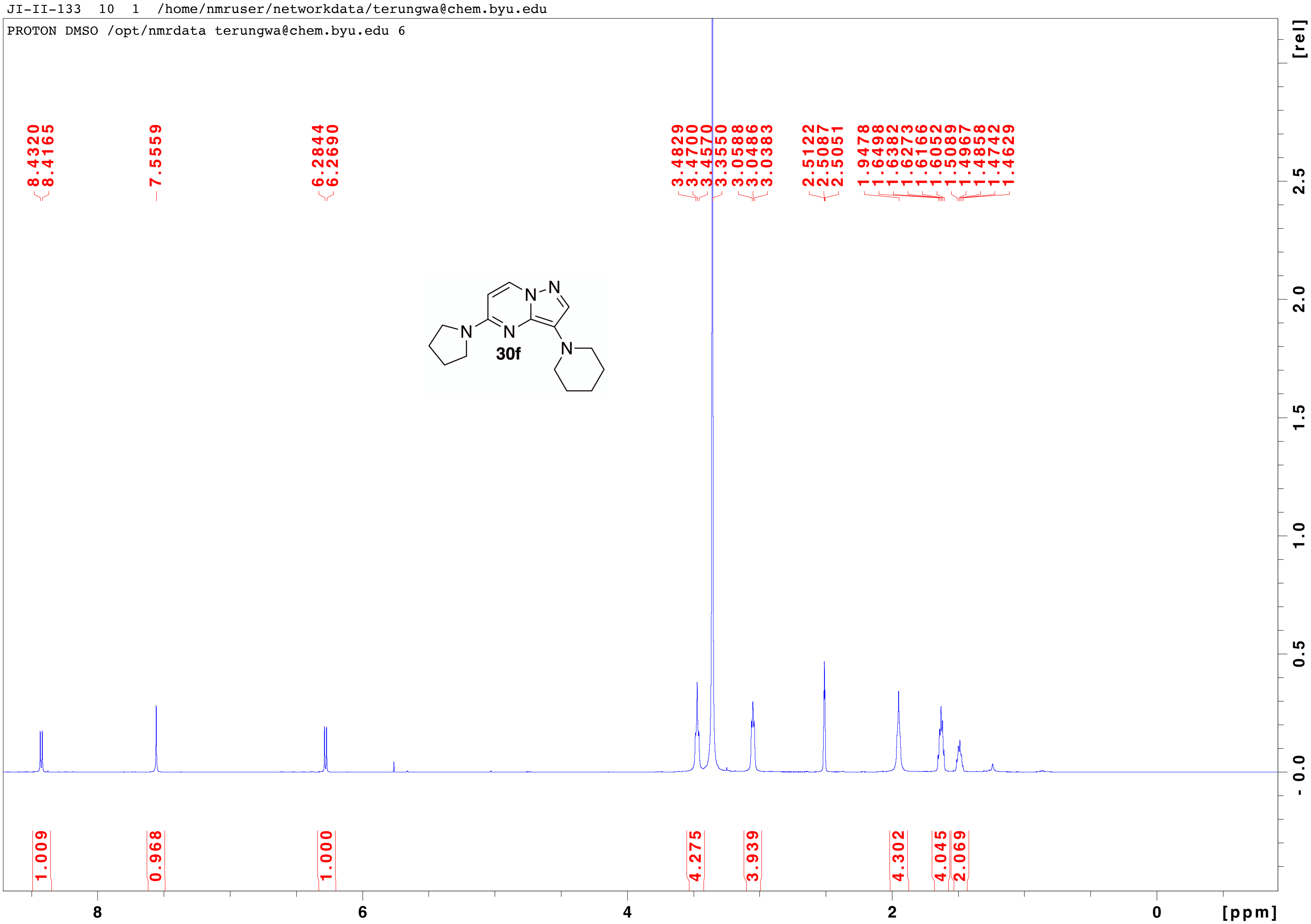

JI-II-133 10 1 /home/nmruser/networkdata/terungwa@chem.byu.edu
PROTON DMSO /opt/nmrdata terungwa@chem.byu.edu 6
8.4320
8.4165
7.5559
6.2844
6.2690
3.4829
3.4700
3.4570
3.3550
3.0588
3.0486
3.0383
2.5122
2.5087
2.5051
1.9478
1.6498
1.6382
1.6273
1.6166
1.6052
1.5089
1.4967
1.4858
1.4742
1.4629
30f
1.009
0.968
1.000
4.275
3.939
4.302
4.045
2.069
[rel]
[ppm]

JI-II-133 10 1 /home/nmruser/networkdata/terungwa@chem.byu.edu

PROTON DMSO /opt/nmrdata terungwa@chem.byu.edu 6

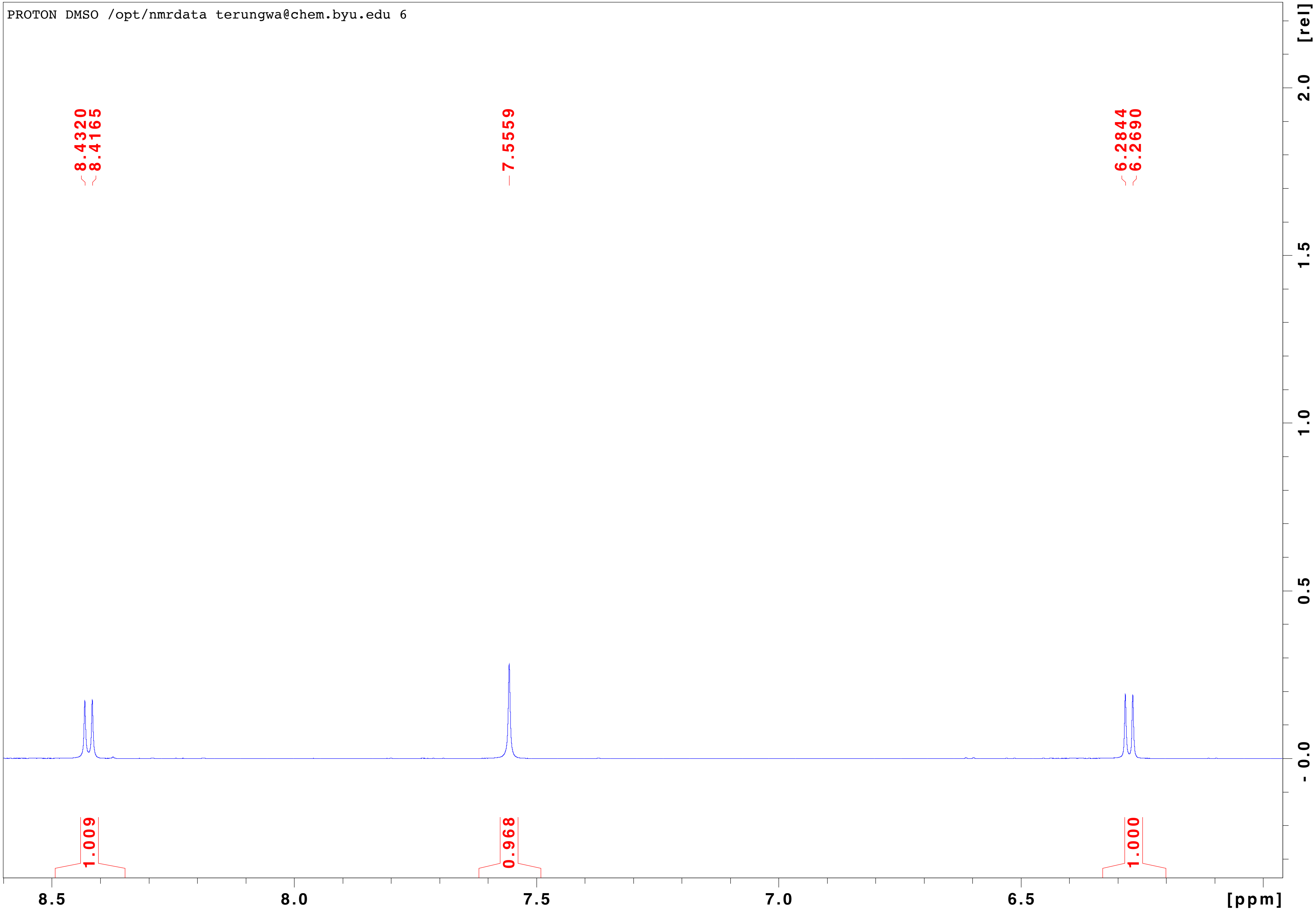

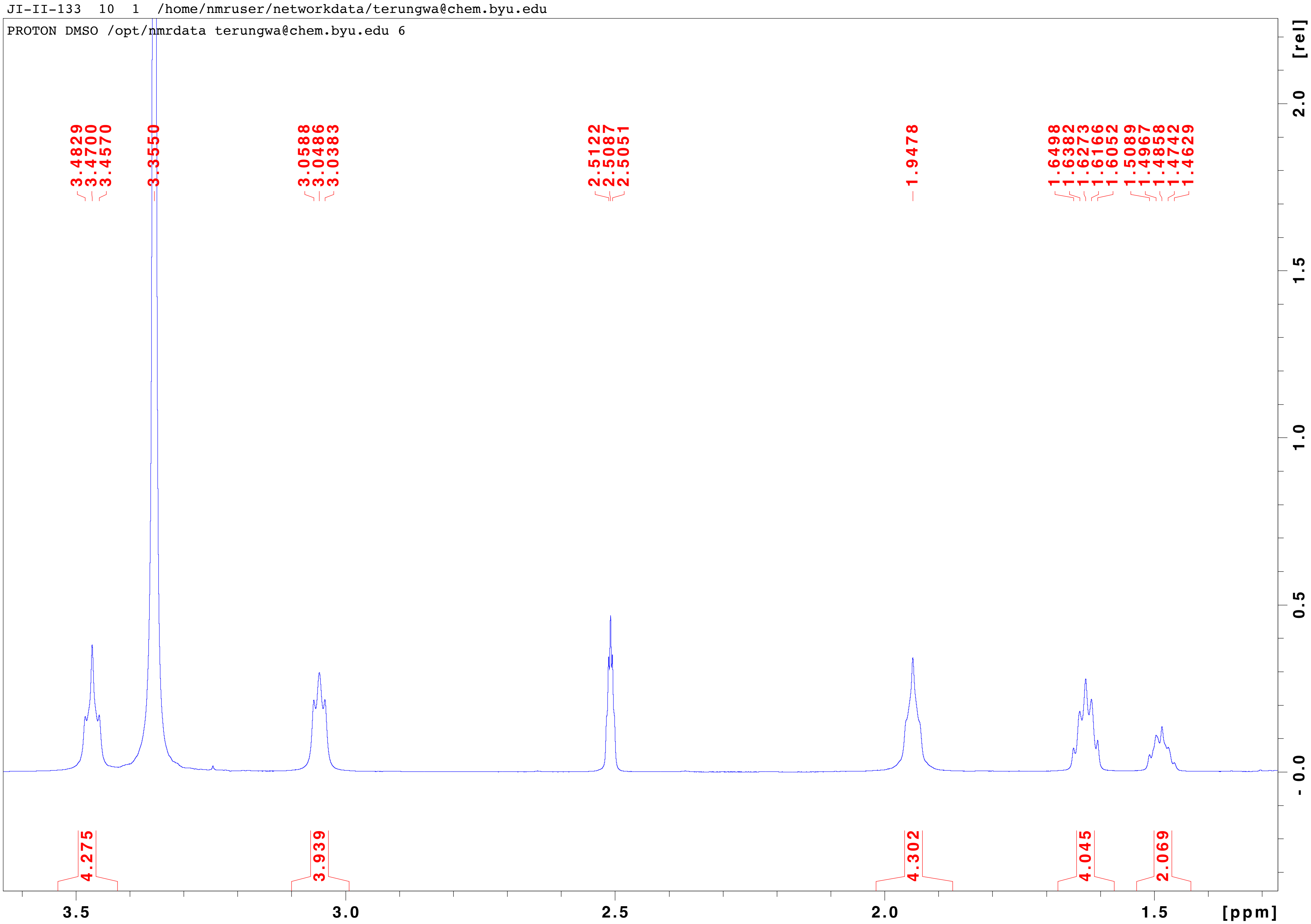

JI-II-133 10 1 /home/nmruser/networkdata/terungwa@chem.byu.edu
PROTON DMSO /opt/nmrdata terungwa@chem.byu.edu 6
3.4829
3.4700
3.4570
3.3550
3.0588
3.0486
3.0383
2.5122
2.5087
2.5051
1.9478
1.6498
1.6382
1.6273
1.6166
1.6052
1.5089
1.4967
1.4858
1.4742
1.4629
4.275
3.939
4.302
4.045
2.069
3.5
3.0
2.5
2.0
1.5
[ppm]
[rel]
2.0
1.5
1.0
0.5
- 0.0

JI-II-133 20 1 /home/nmruser/networkdata/terungwa@chem.byu.edu

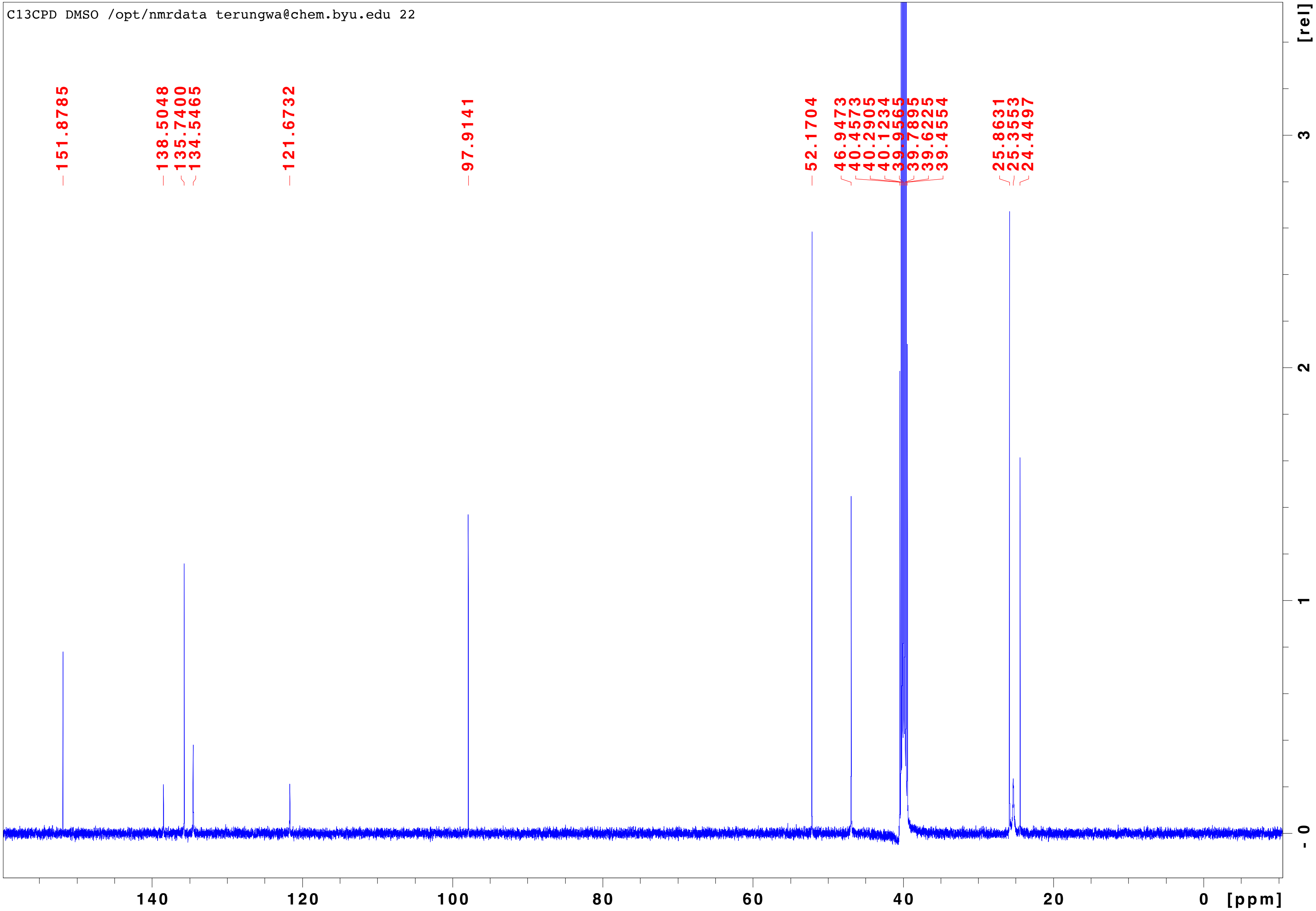

LG-I-55 30 1 /home/nmruser/networkdata/Bruker500Data/log96@chem.byu.edu

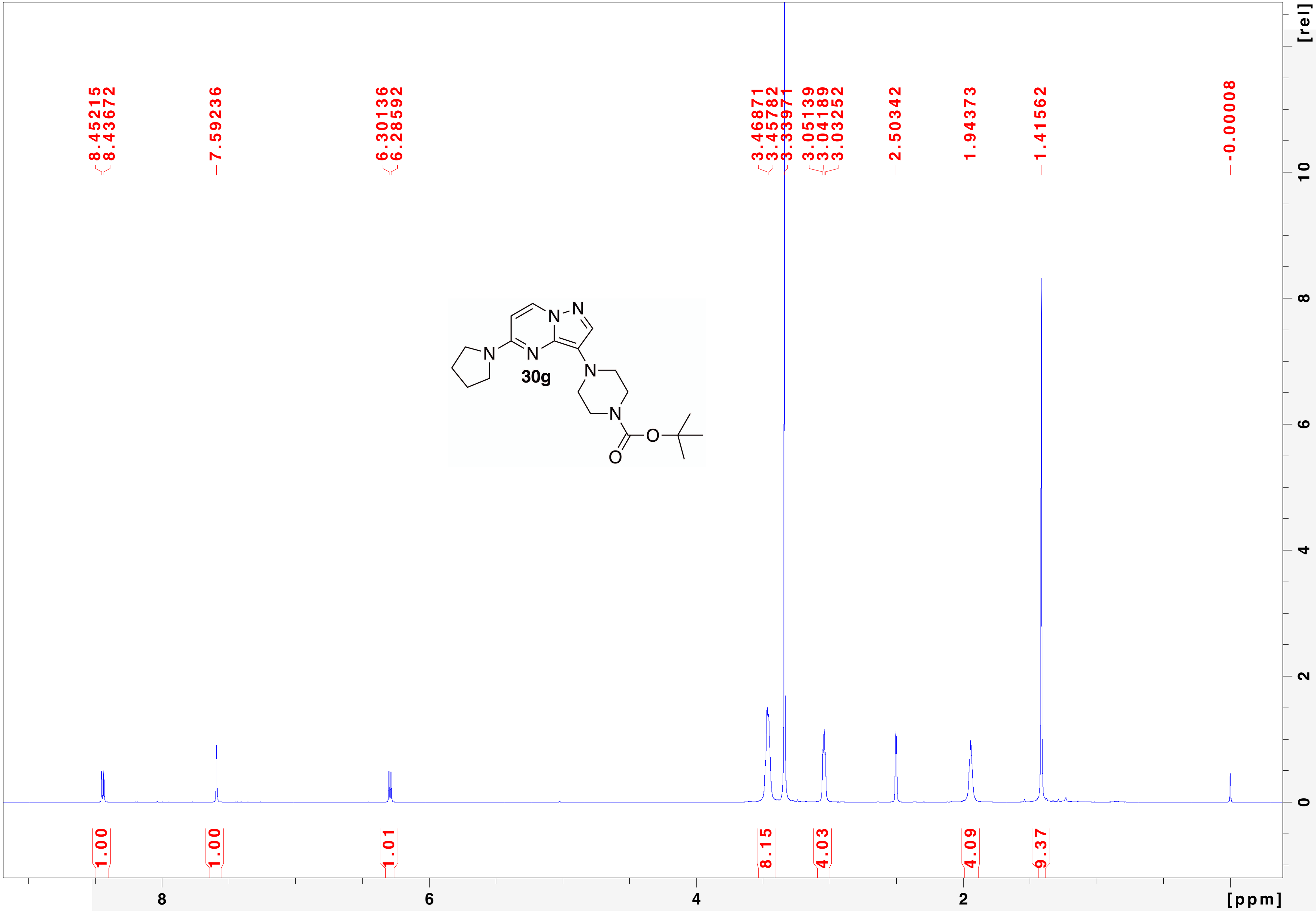

LG-I-55 20 1 /home/nmruser/networkdata/Bruker500Data/log96@chem.byu.edu

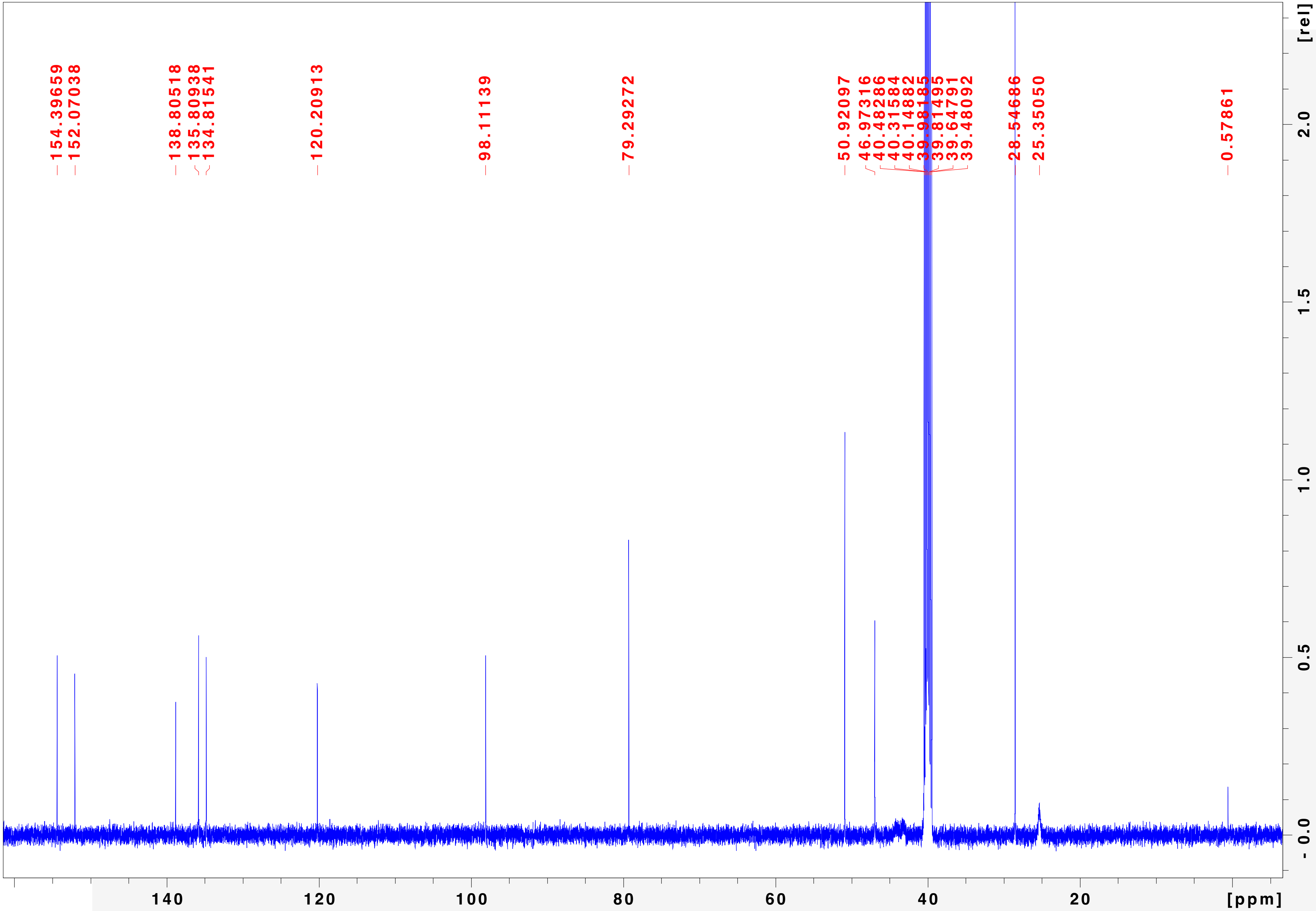

JI-II-116 20 1 /home/nmruser/networkdata/terungwa@chem.byu.edu

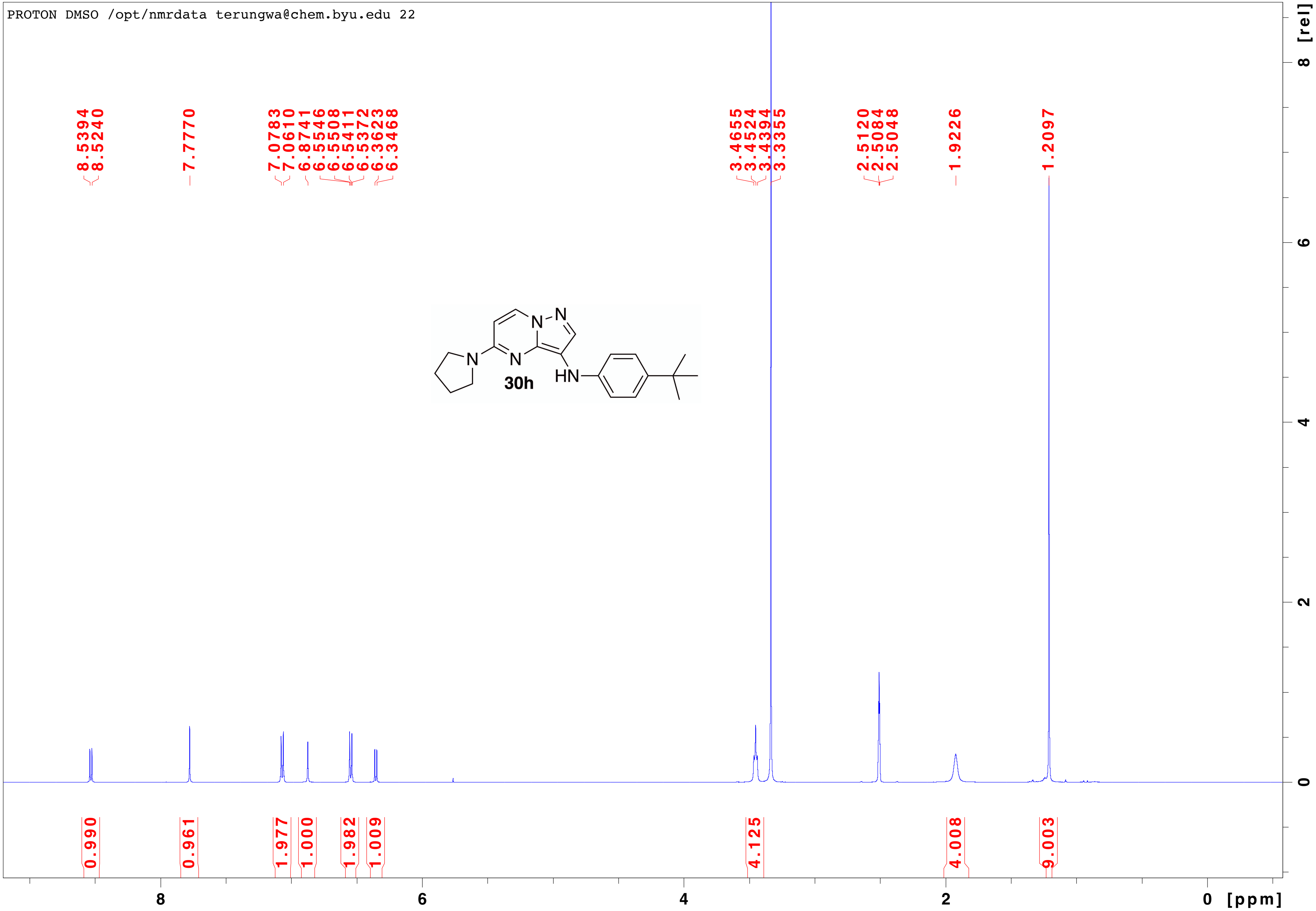

JI-II-116 20 1 /home/nmruser/networkdata/terungwa@chem.byu.edu

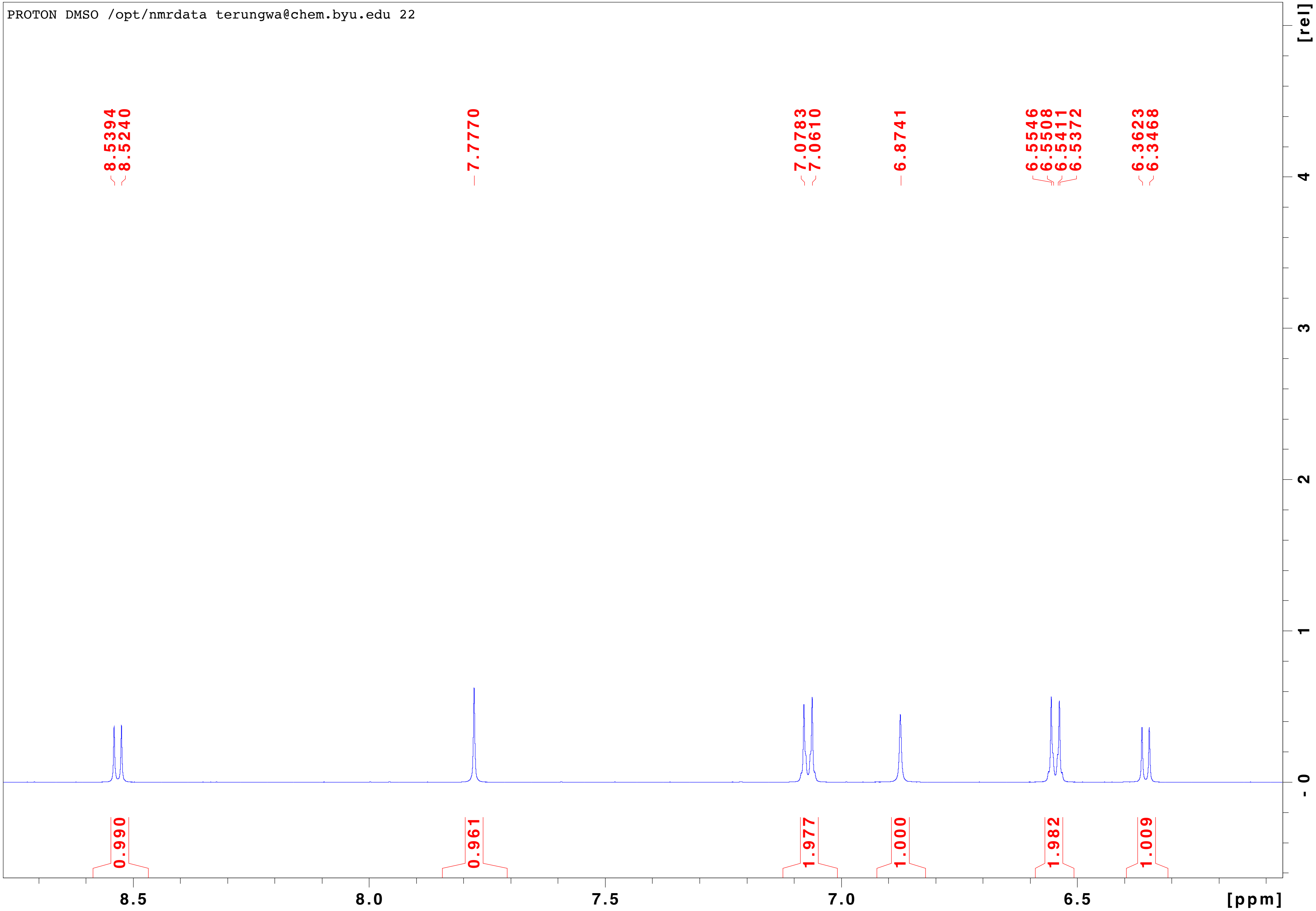

JI-II-116 20 1 /home/nmruser/networkdata/terungwa@chem.byu.edu

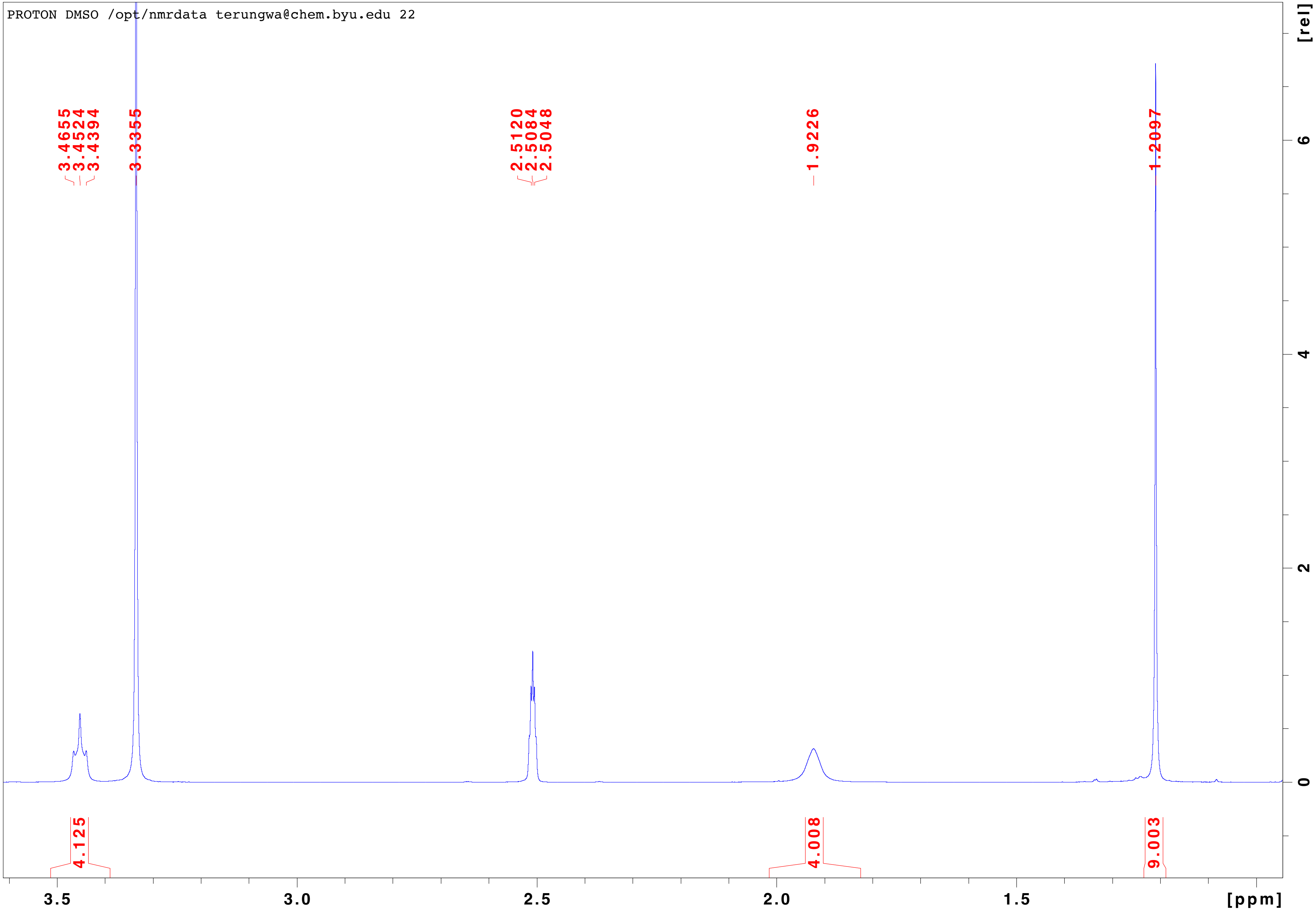

JI-II-116 30 1 /home/nmruser/networkdata/terungwa@chem.byu.edu

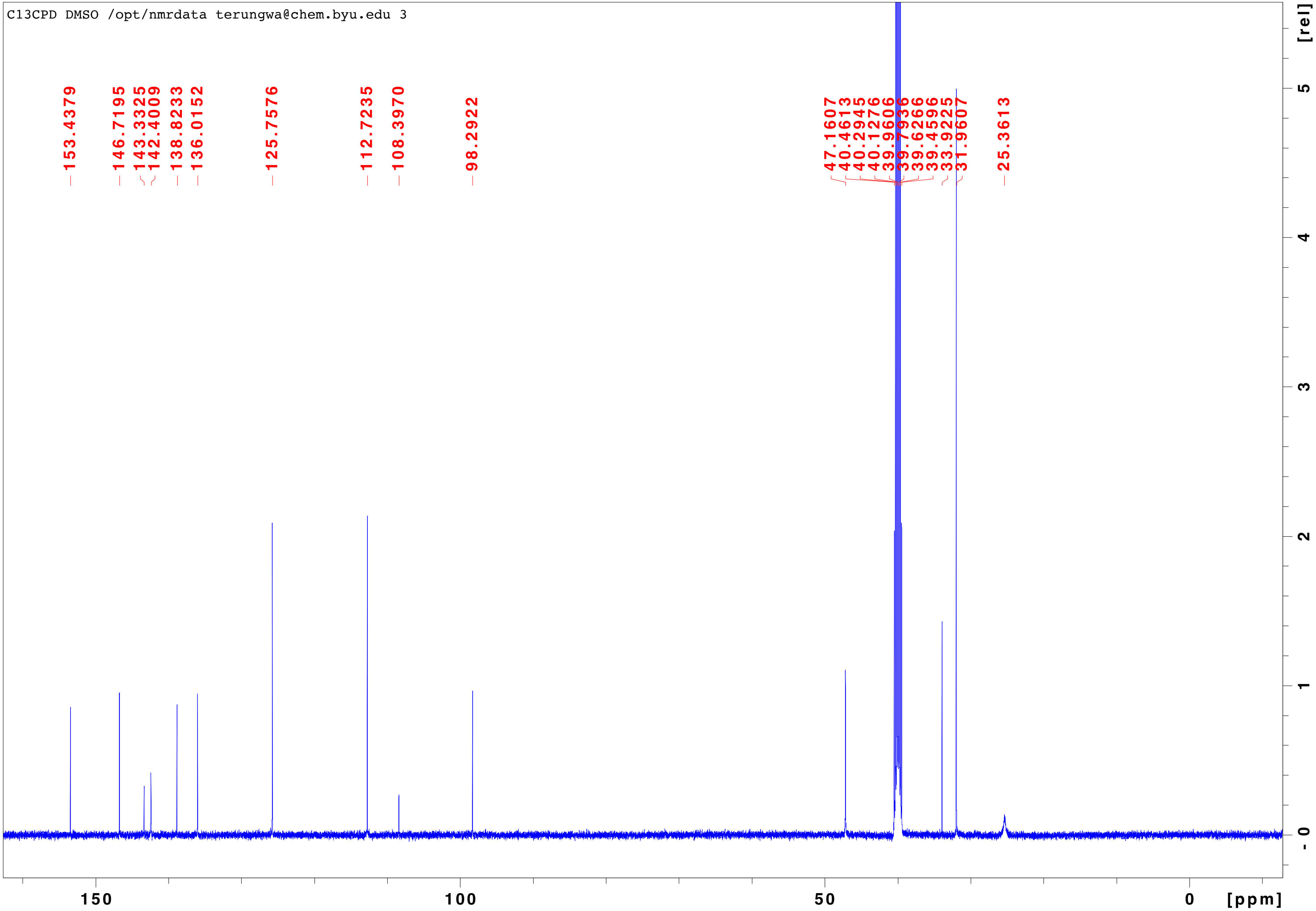

JI-II-115 20 1 /home/nmruser/networkdata/terungwa@chem.byu.edu

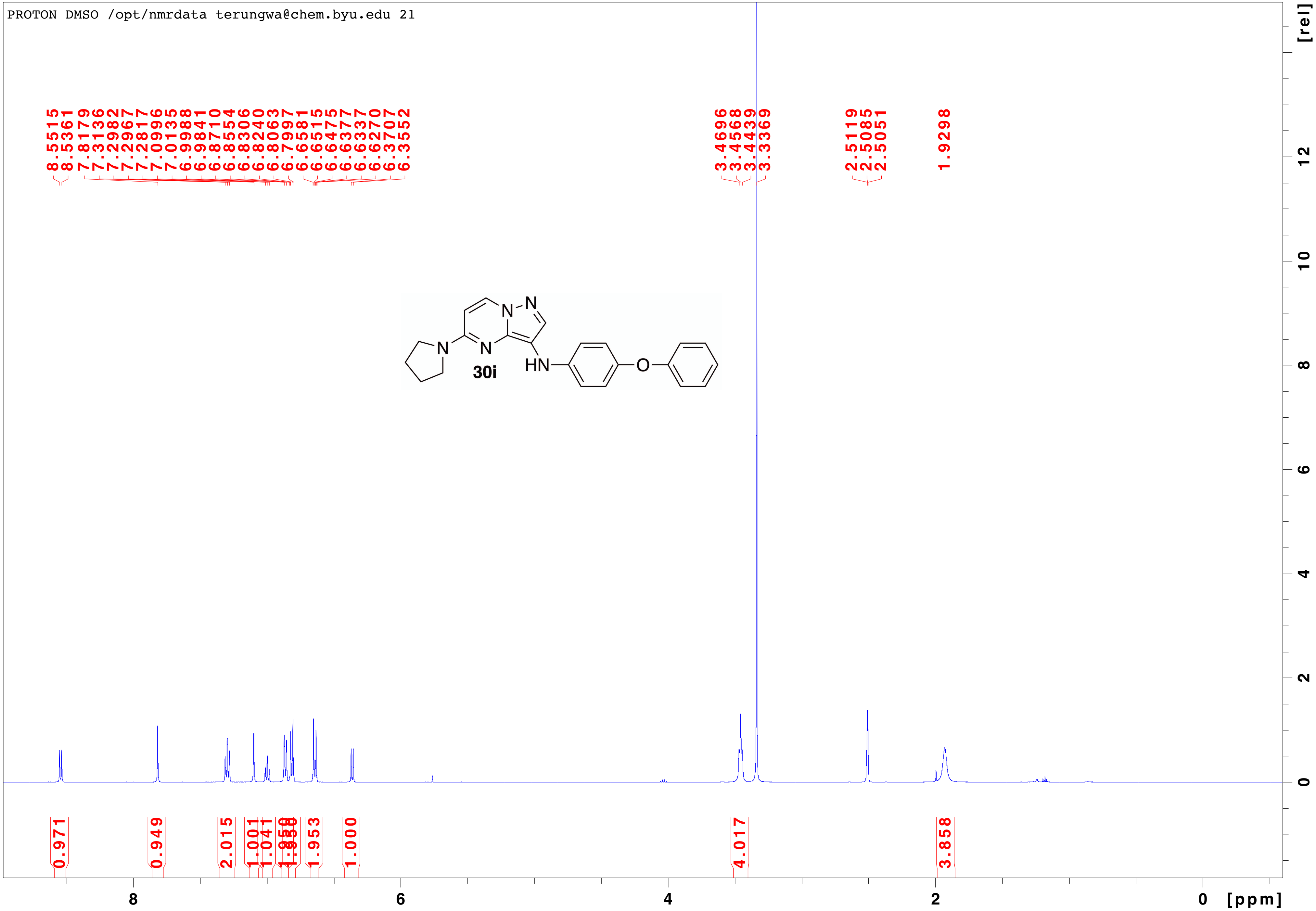

JI-II-115 20 1 /home/nmruser/networkdata/terungwa@chem.byu.edu

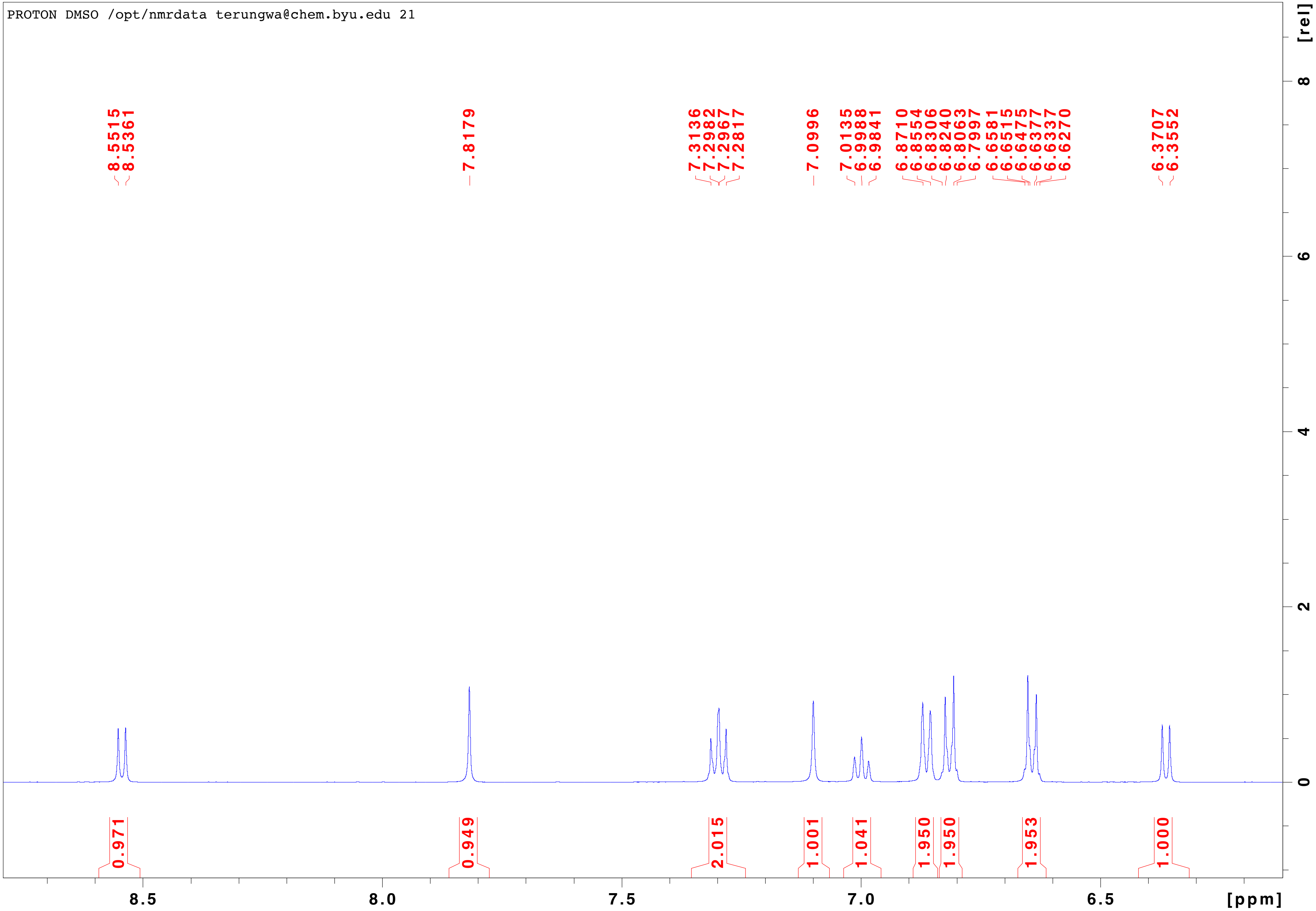

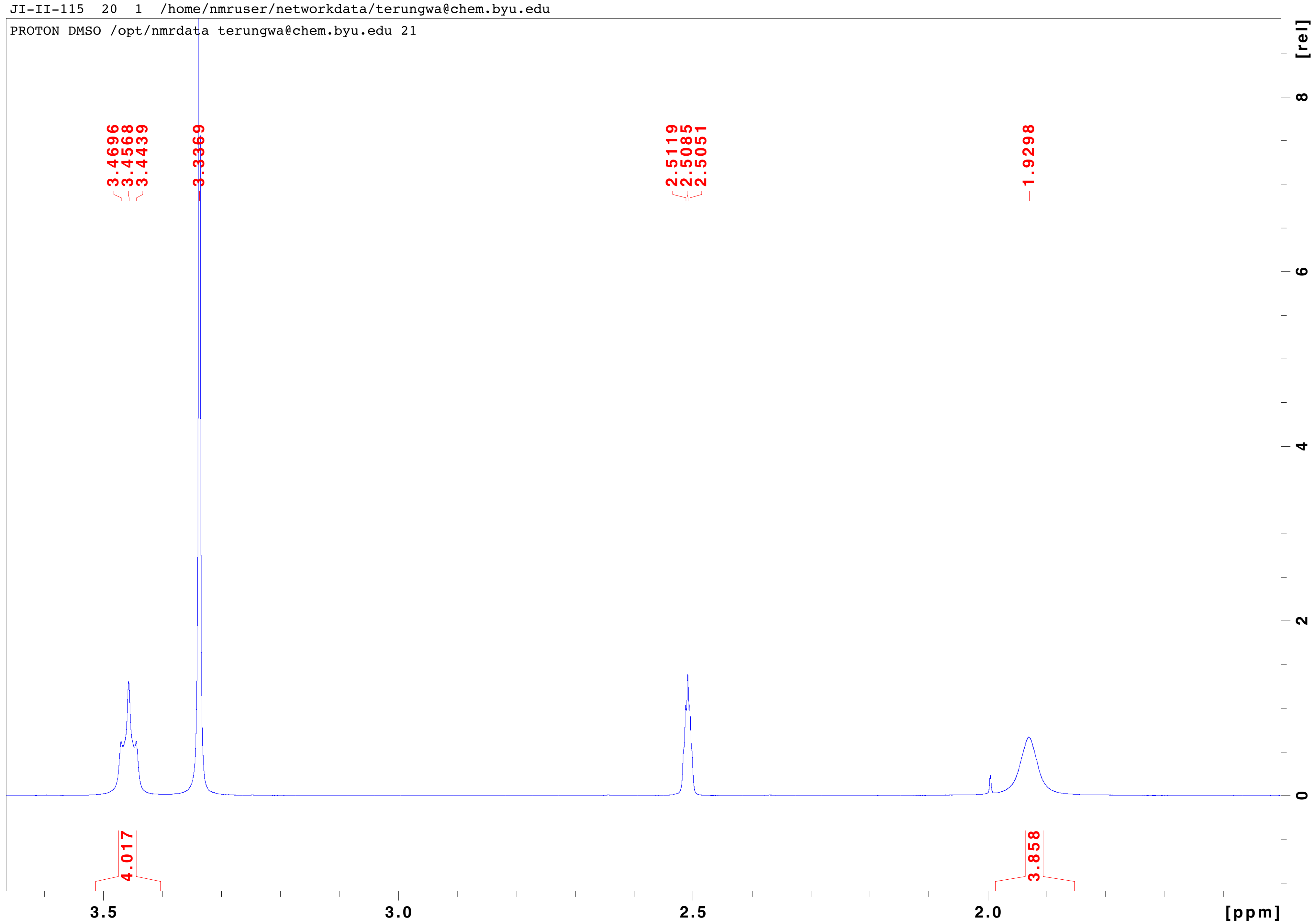
JI-II-115 20 1 /home/nmruser/networkdata/terungwa@chem.byu.edu
PROTON DMSO /opt/nmrdata terungwa@chem.byu.edu 21
3.4696
3.4568
3.4439
3.3369
2.5119
2.5085
2.5051
1.9298
4.017
3.858
[rel]
8
6
4
2
0
3.5
3.0
2.5
2.0
[ppm]

JI-II-115 30 1 /home/nmruser/networkdata/terungwa@chem.byu.edu

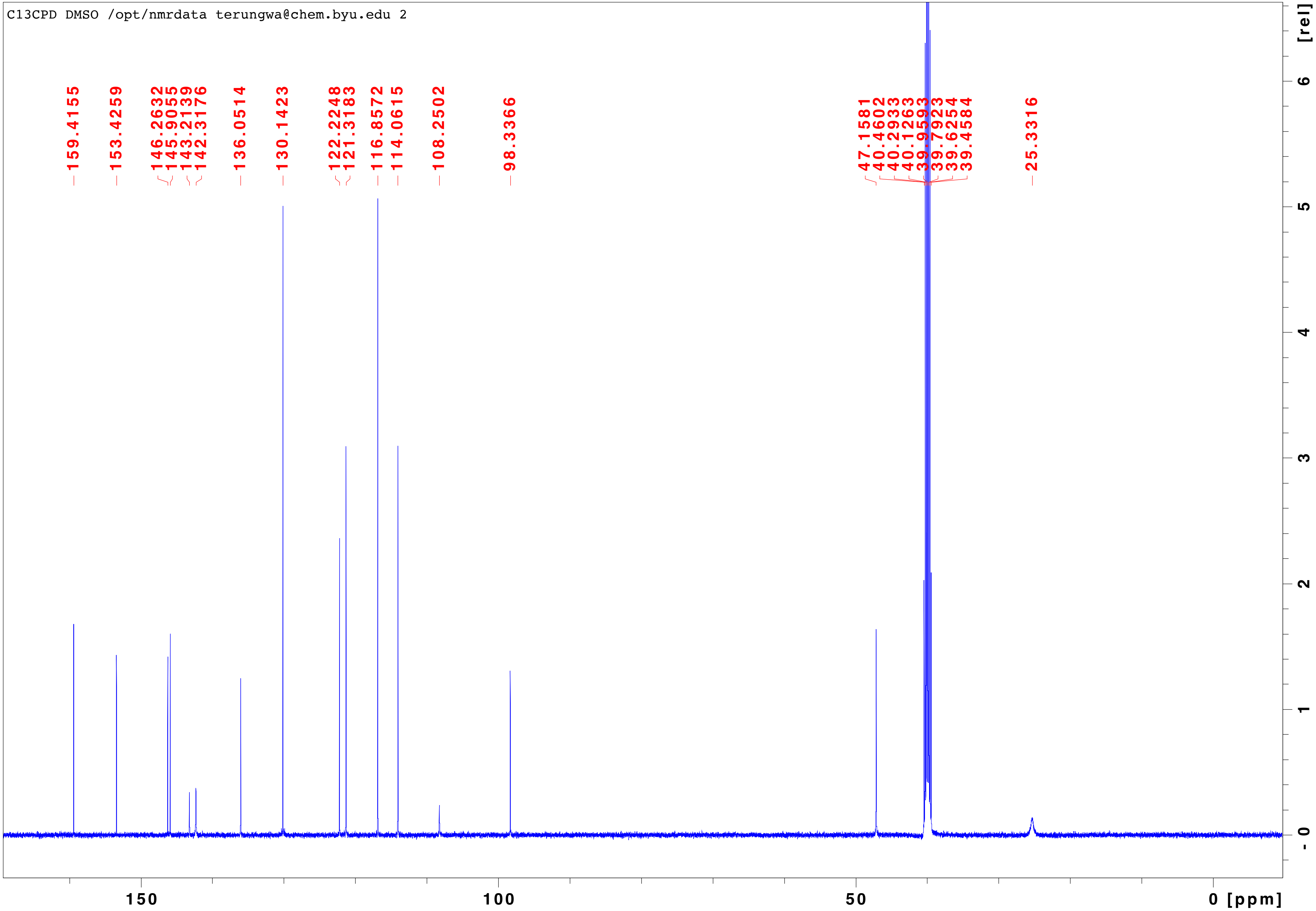

JI-II-117 10 1 /home/nmruser/networkdata/terungwa@chem.byu.edu

PROTON DMSO /opt/nmrdata terungwa@chem.byu.edu 9

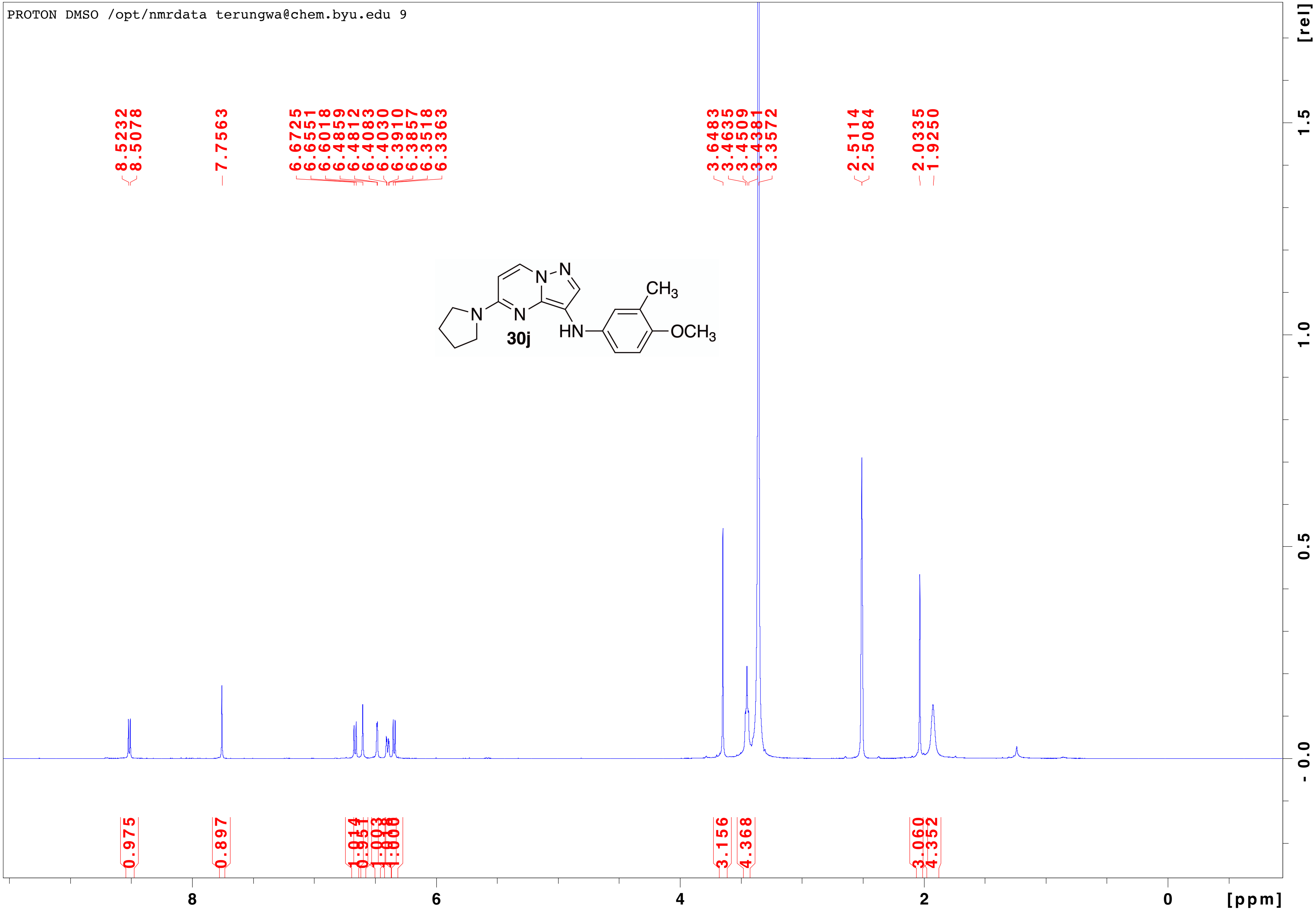

JI-II-117 10 1 /home/nmruser/networkdata/terungwa@chem.byu.edu

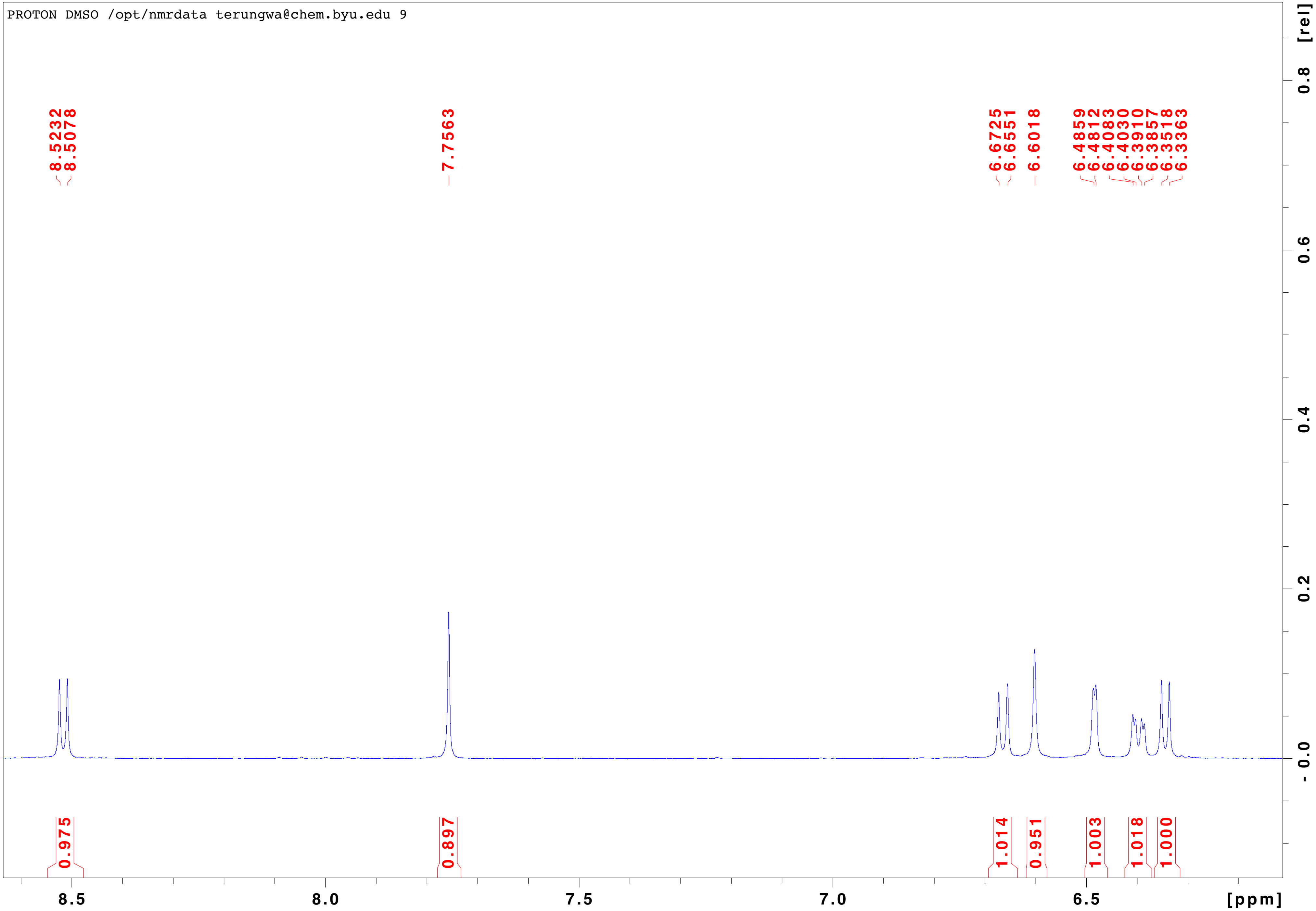

JI-II-117 10 1 /home/nmruser/networkdata/terungwa@chem.byu.edu

PROTON DMSO /opt/nmrdata terungwa@chem.byu.edu 9

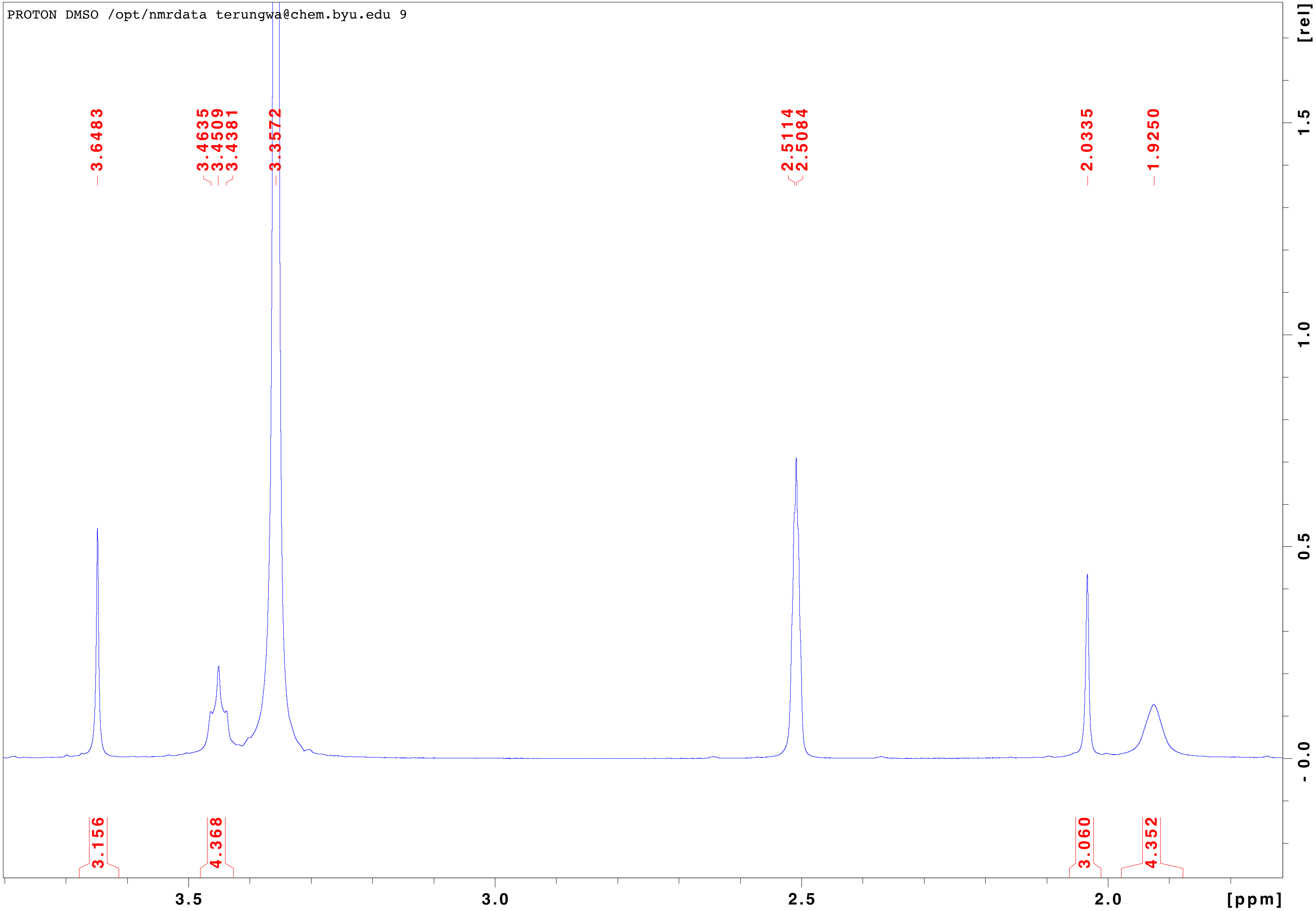

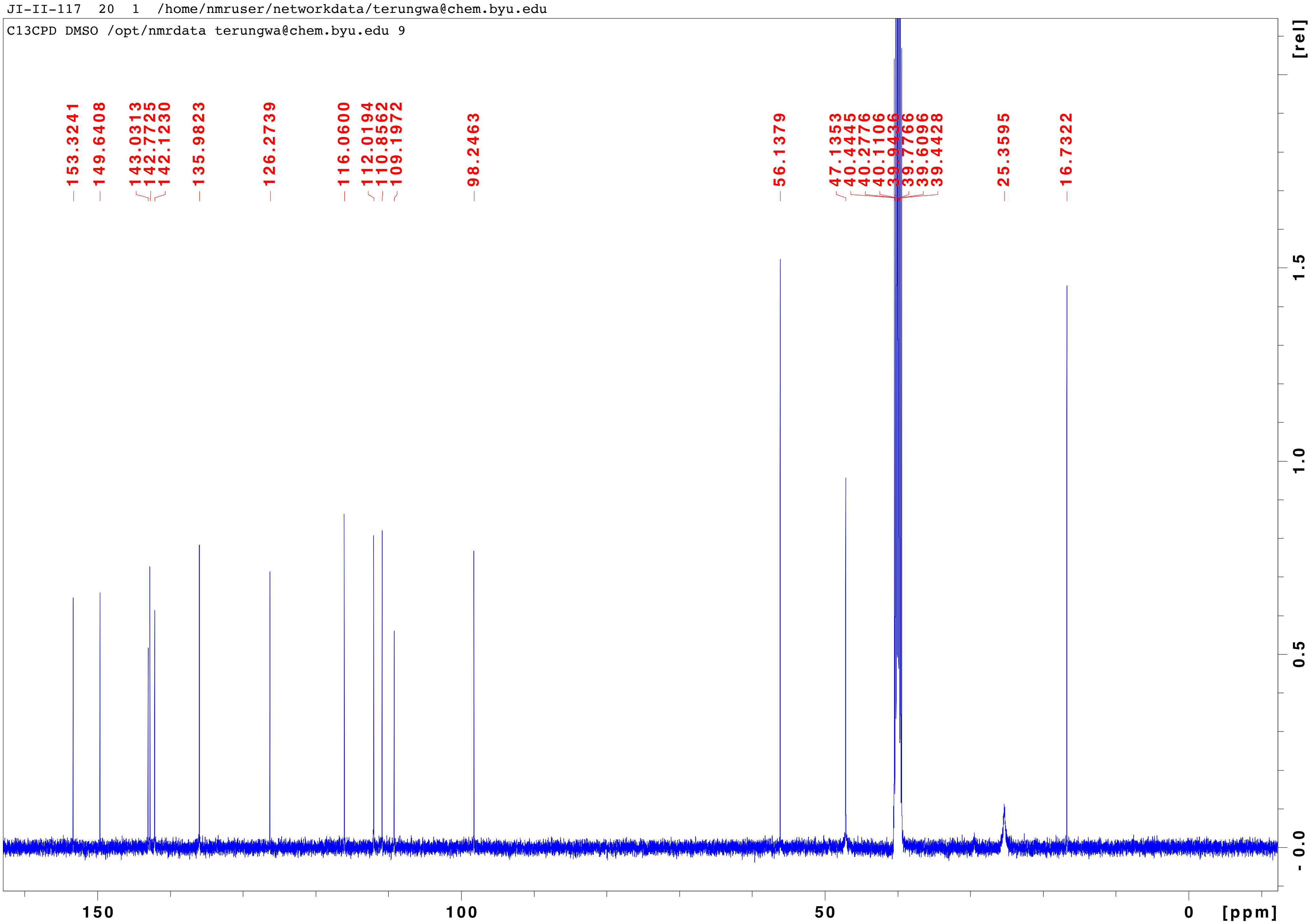
JI-II-117 20 1 /home/nmruser/networkdata/terungwa@chem.byu.edu
C13CPD DMSO /opt/nmrdata terungwa@chem.byu.edu 9
153.3241
149.6408
143.0313
142.7725
142.1230
135.9823
126.2739
116.0600
112.0194
110.8562
109.1972
98.2463
56.1379
47.1353
40.4445
40.2776
40.1106
39.9436
39.7766
39.6096
39.4428
25.3595
16.7322
[rel]
1.5
1.0
0.5
0.0
150
100
50
0
[ppm]

JI-II-123 20 1 /home/nmruser/networkdata/terungwa@chem.byu.edu

PROTON DMSO /opt/nmrdata terungwa@chem.byu.edu 5

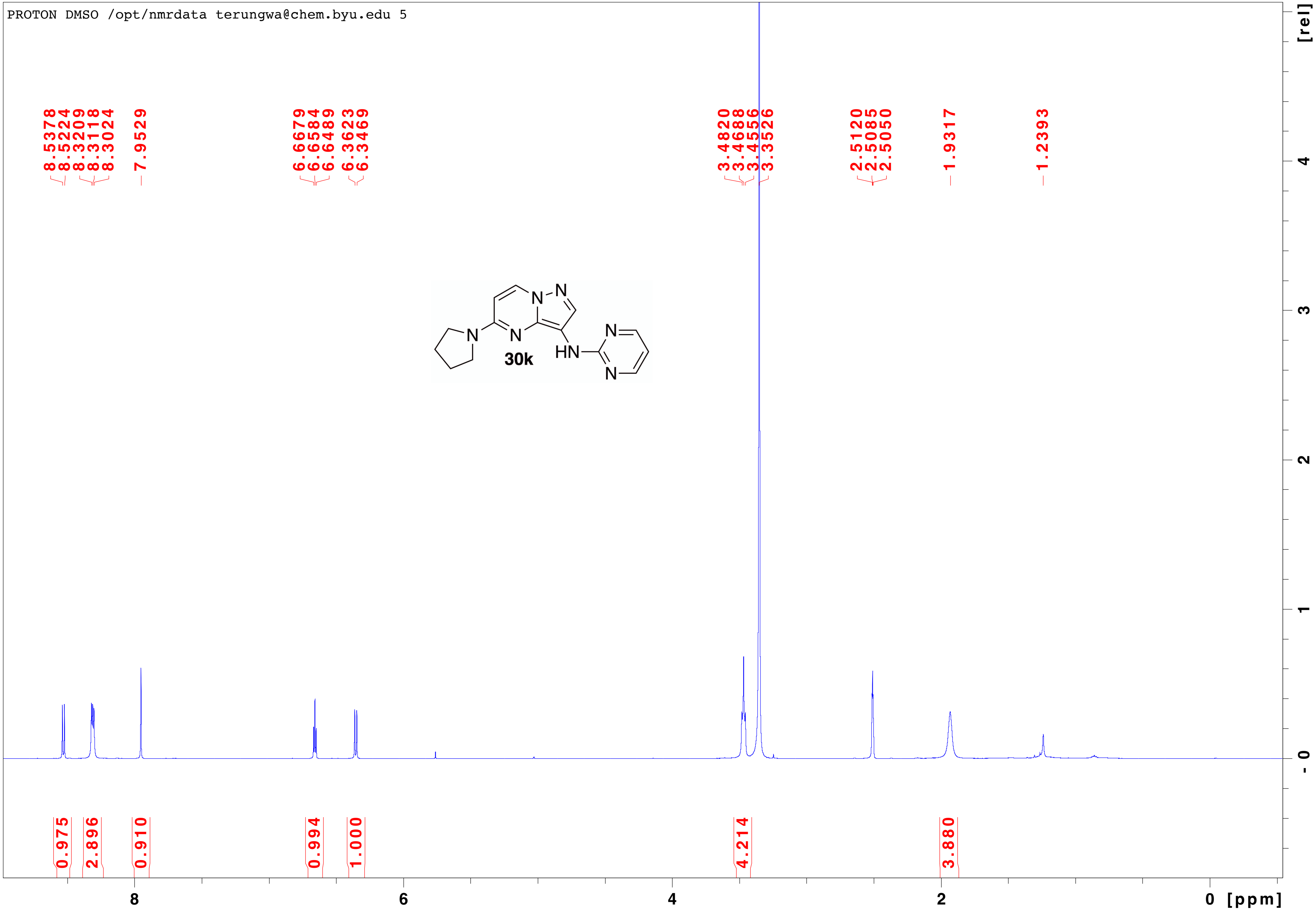

JI-II-123 20 1 /home/nmruser/networkdata/terungwa@chem.byu.edu

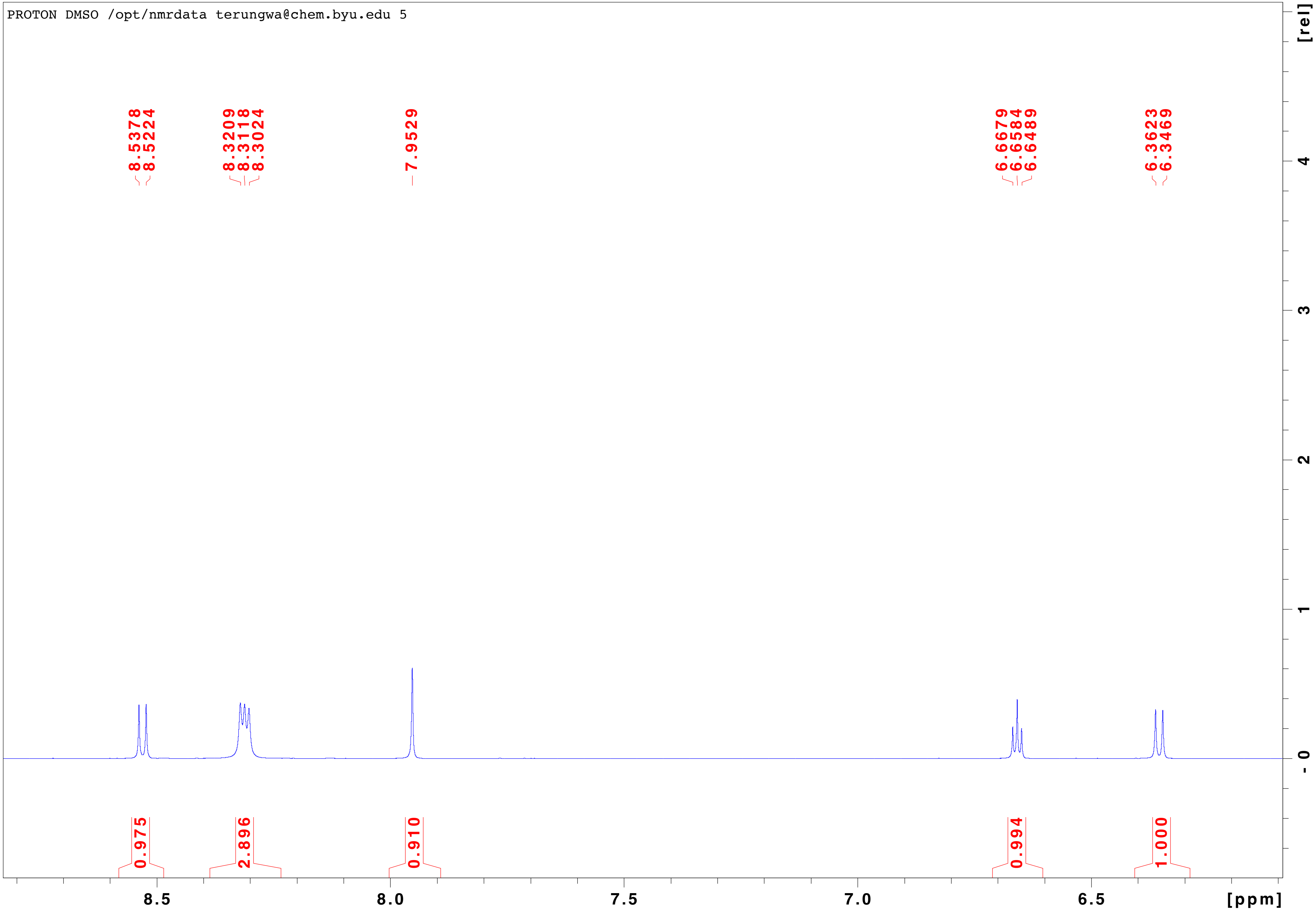

JI-II-123 20 1 /home/nmruser/networkdata/terungwa@chem.byu.edu

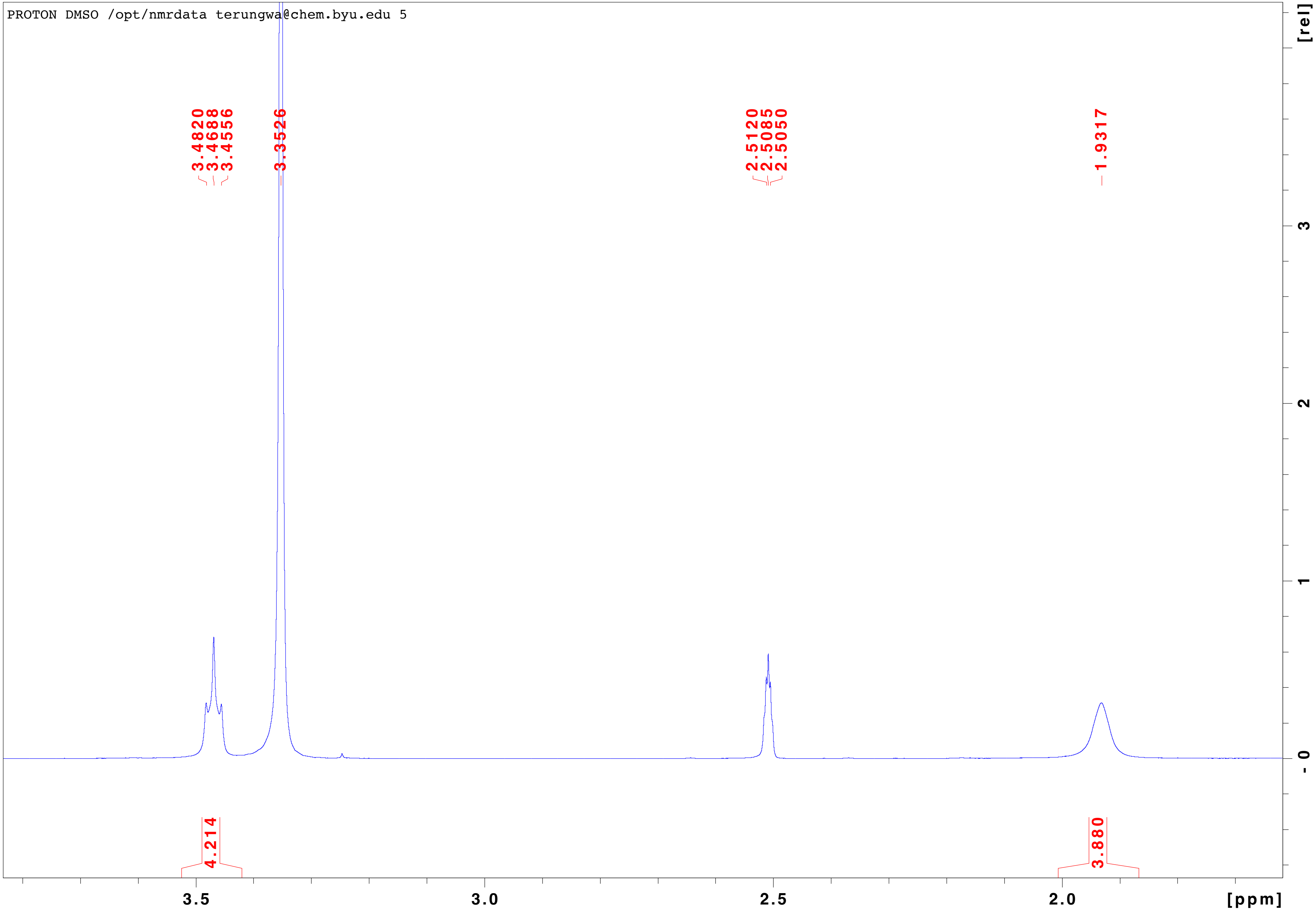

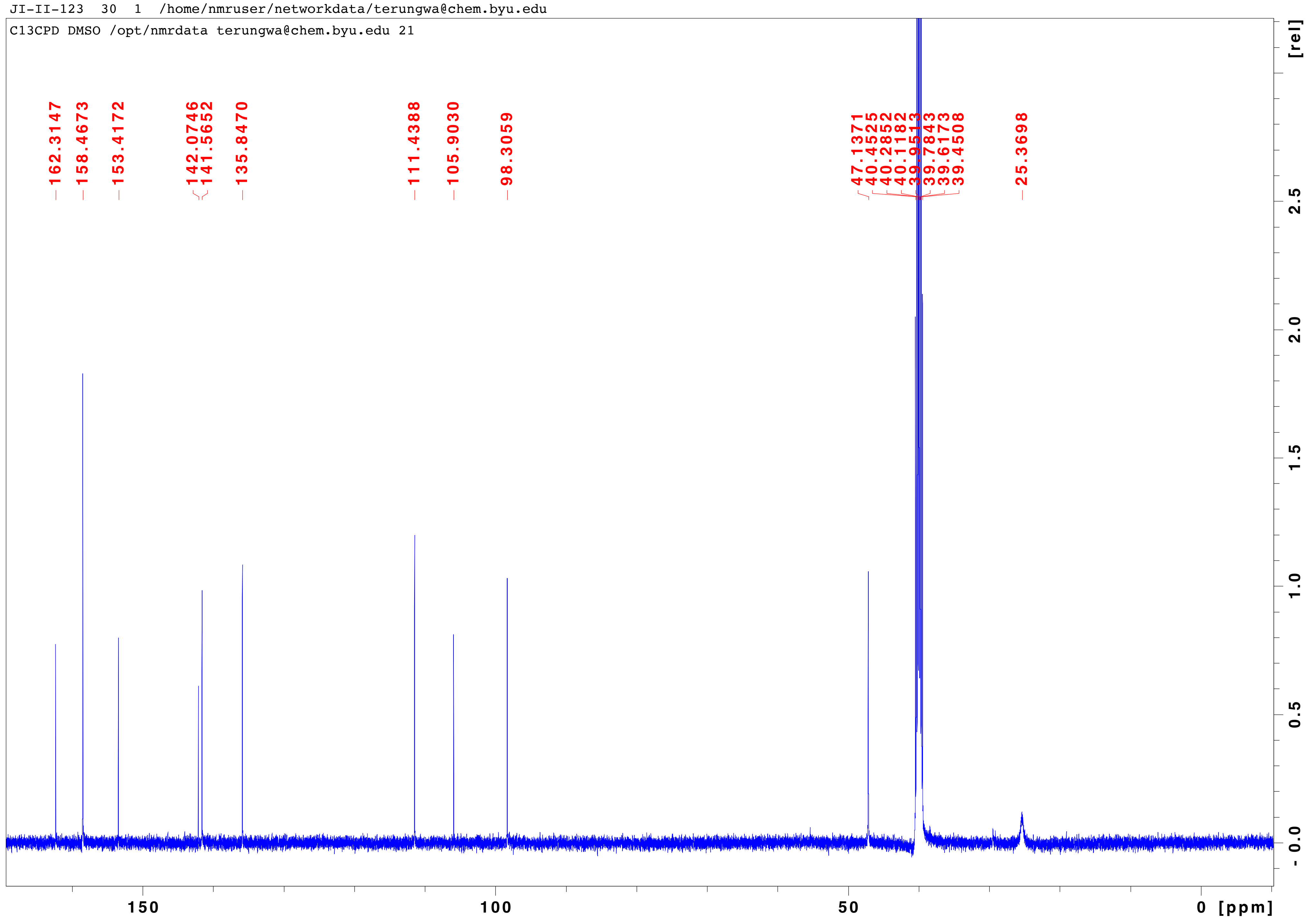

JI-II-123 30 1 /home/nmruser/networkdata/terungwa@chem.byu.edu
C13CPD DMSO /opt/nmrdata terungwa@chem.byu.edu 21
162.3147
158.4673
153.4172
142.0746
141.5652
135.8470
111.4388
105.9030
98.3059
47.1371
40.4525
40.2852
40.1182
39.9513
39.7843
39.6173
39.4508
25.3698
[rel]
2.5
2.0
1.5
1.0
0.5
0.0
150
100
50
0 [ppm]

JI-II-125 20 1 /home/nmruser/networkdata/terungwa@chem.byu.edu

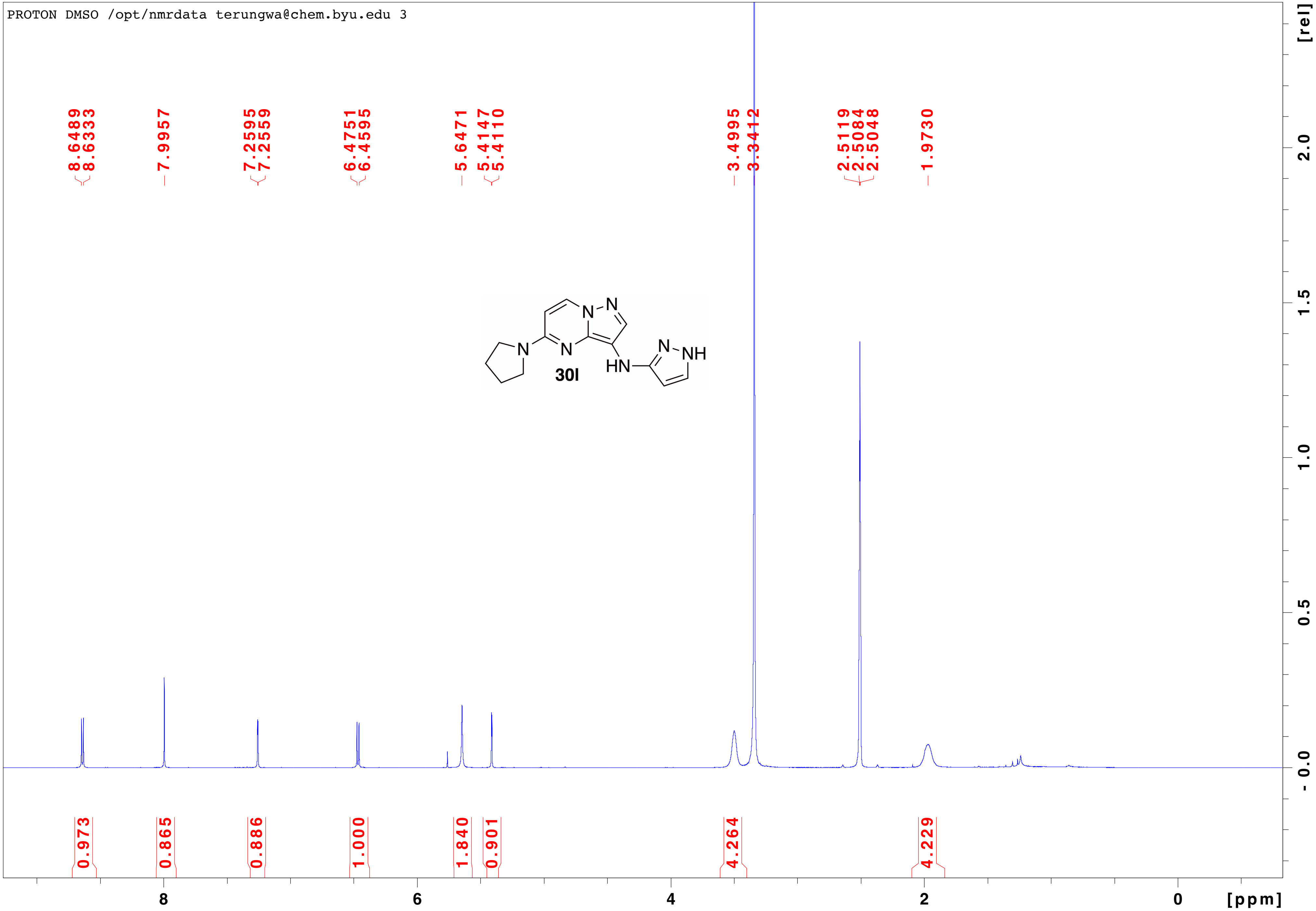

JI-II-125 20 1 /home/nmruser/networkdata/terungwa@chem.byu.edu

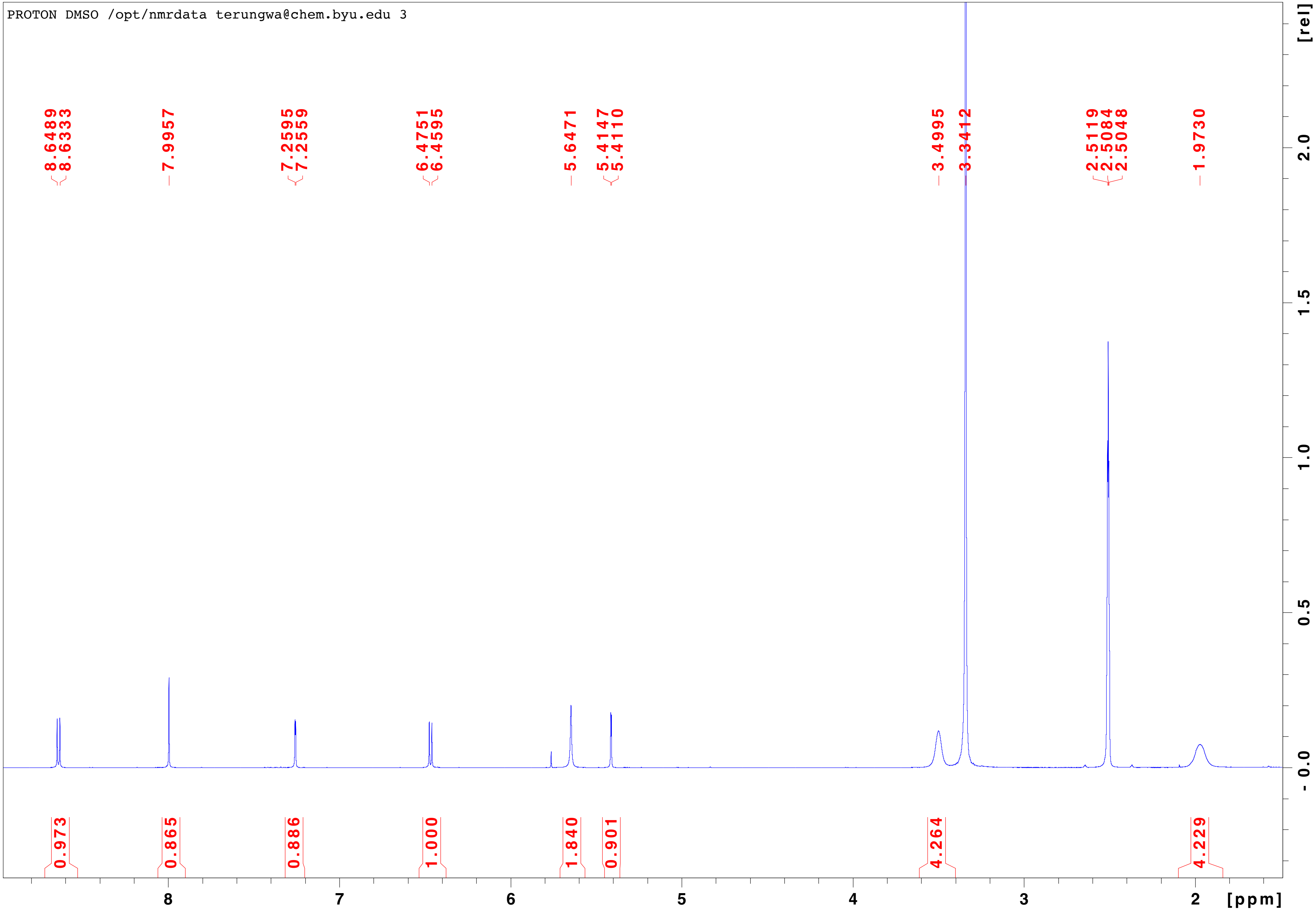

JI-II-125 40 1 /home/nmruser/networkdata/terungwa@chem.byu.edu

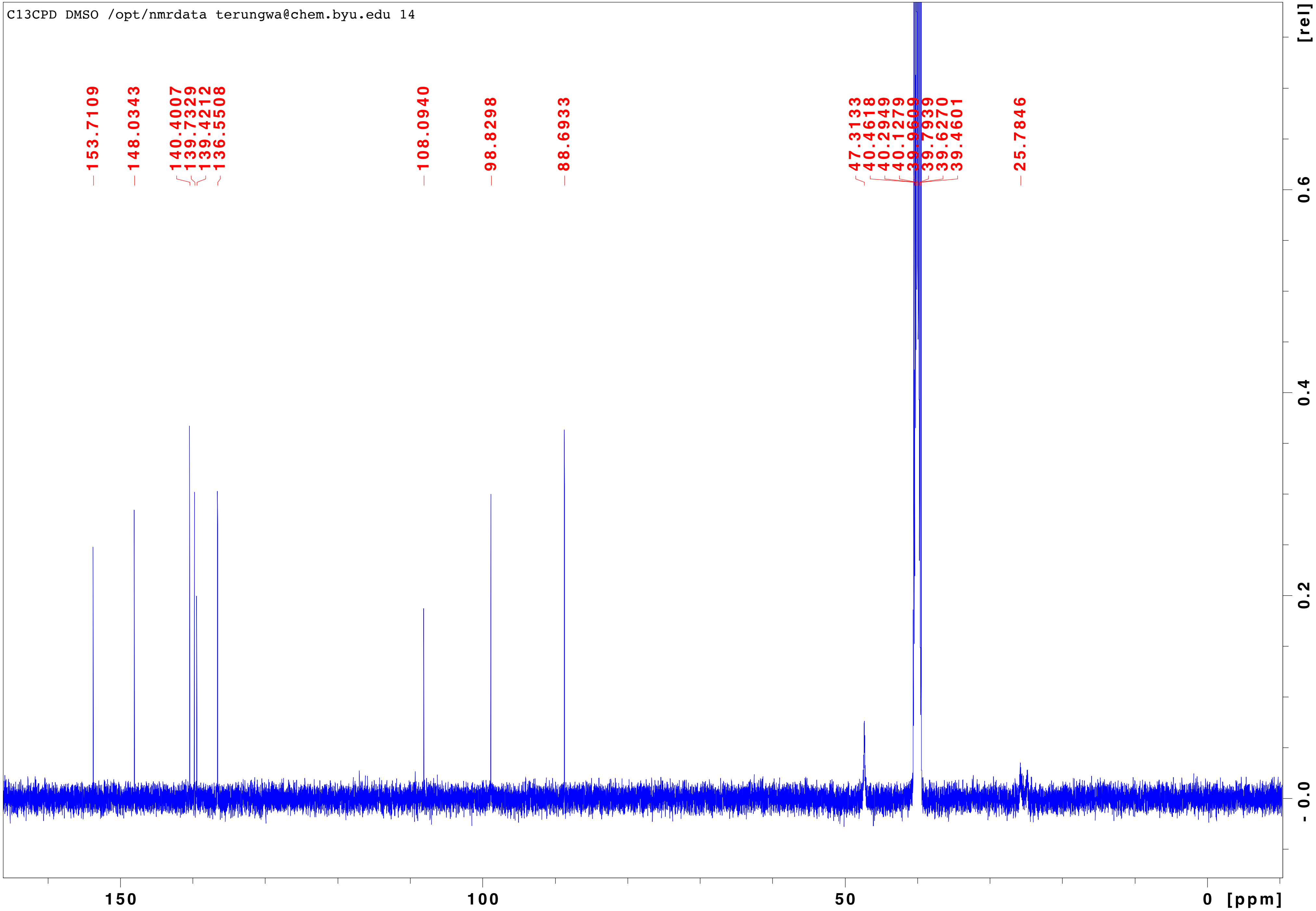

JI-II-125Br 10 1 /home/nmruser/networkdata/Bruker500Data/terungwa@chem.byu.edu

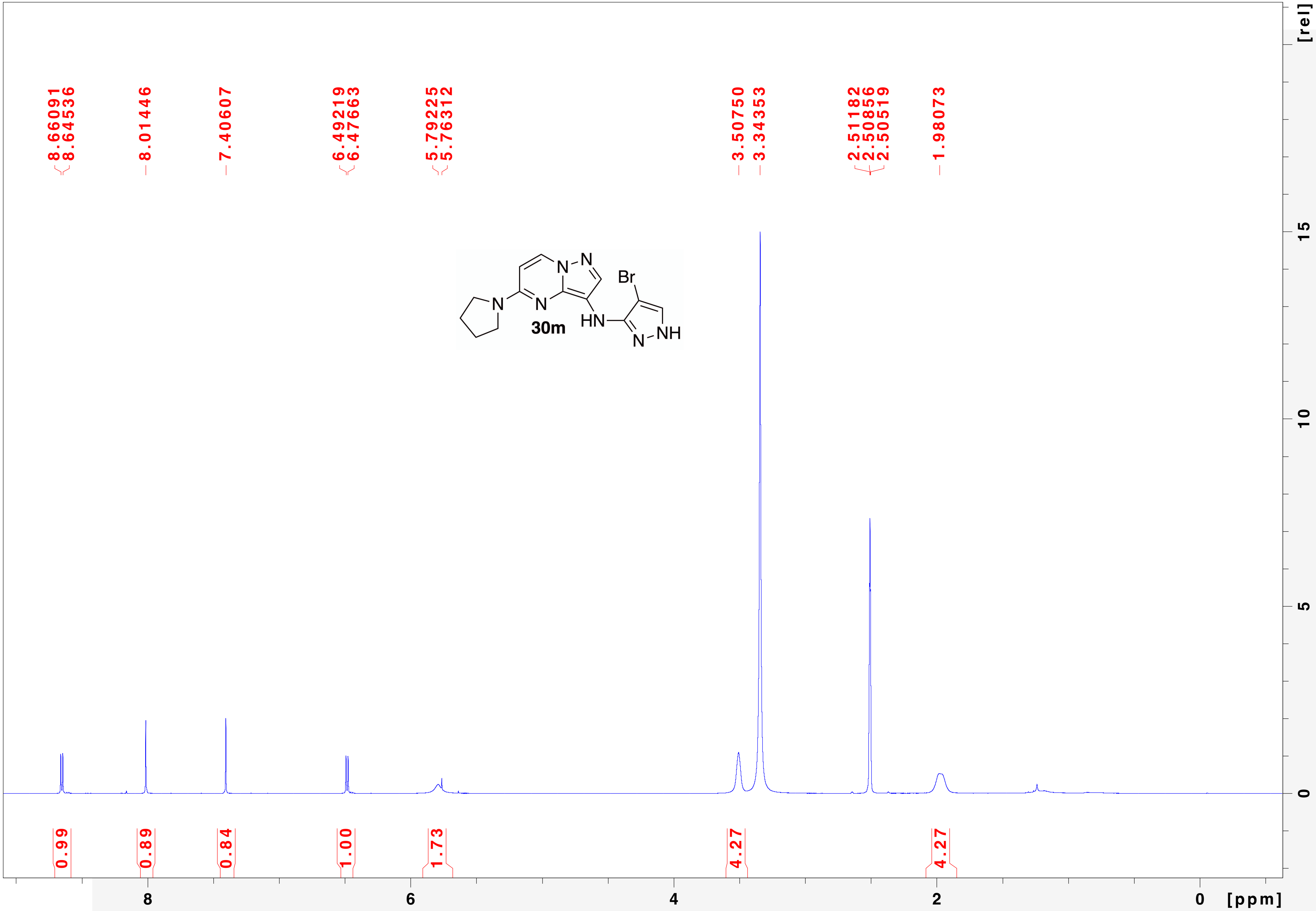

JI-II-125Br 10 1 /home/nmruser/networkdata/Bruker500Data/terungwa@chem.byu.edu

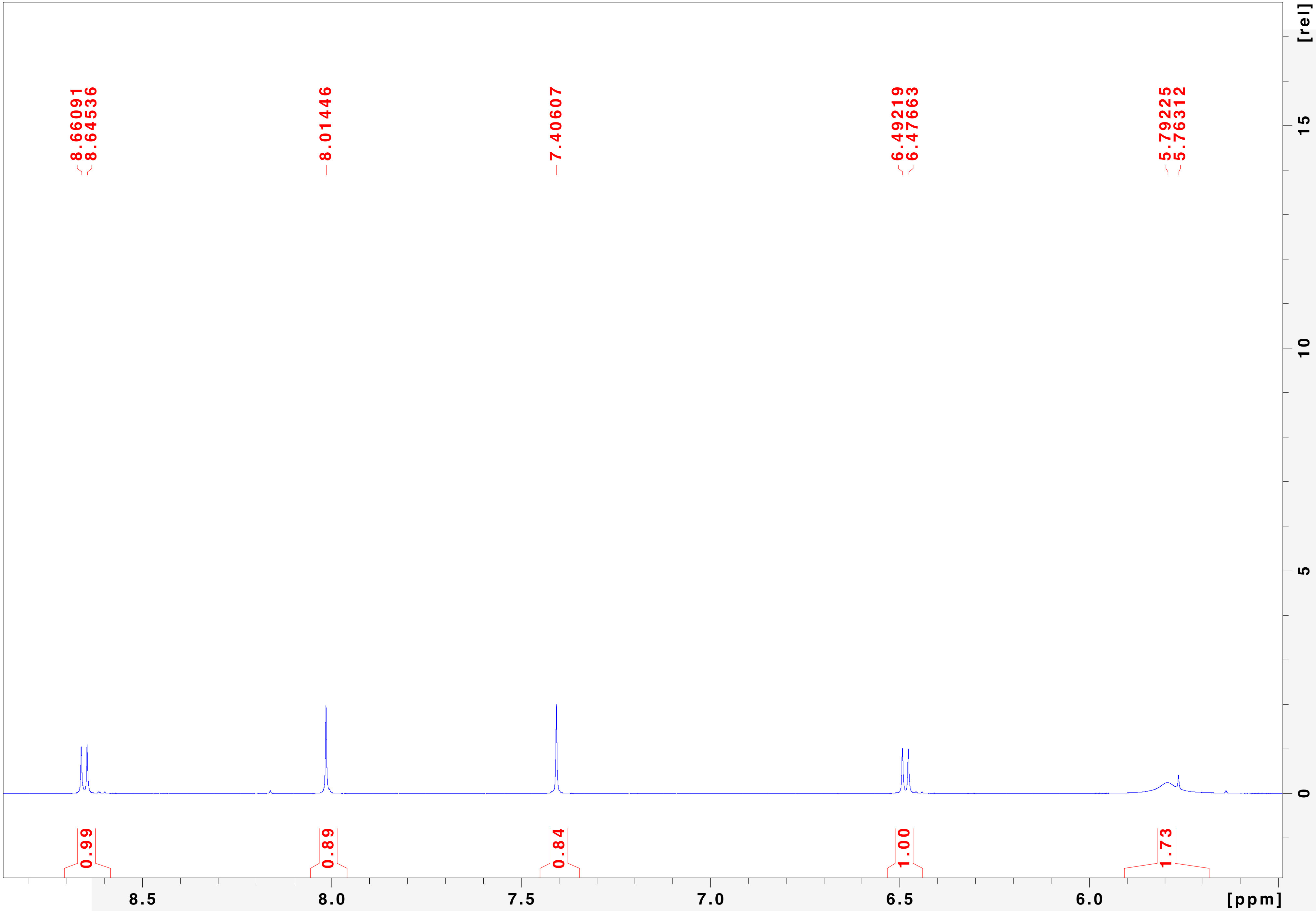

JI-II-125Br 30 1 /home/nmruser/networkdata/Bruker500Data/terungwa@chem.byu.edu

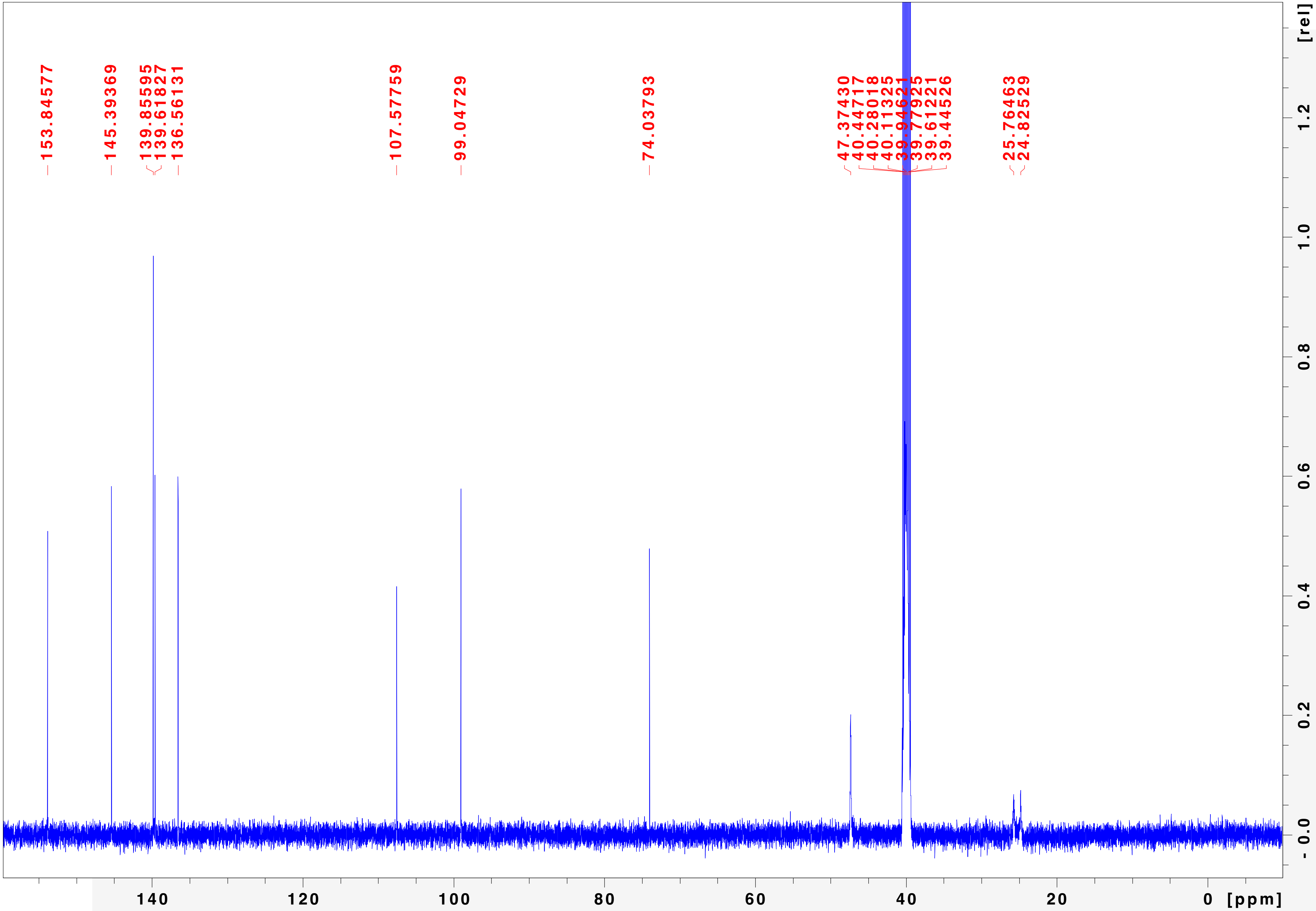

LG-I-43 40 1 /home/nmruser/networkdata/Bruker500Data/log96@chem.byu.edu

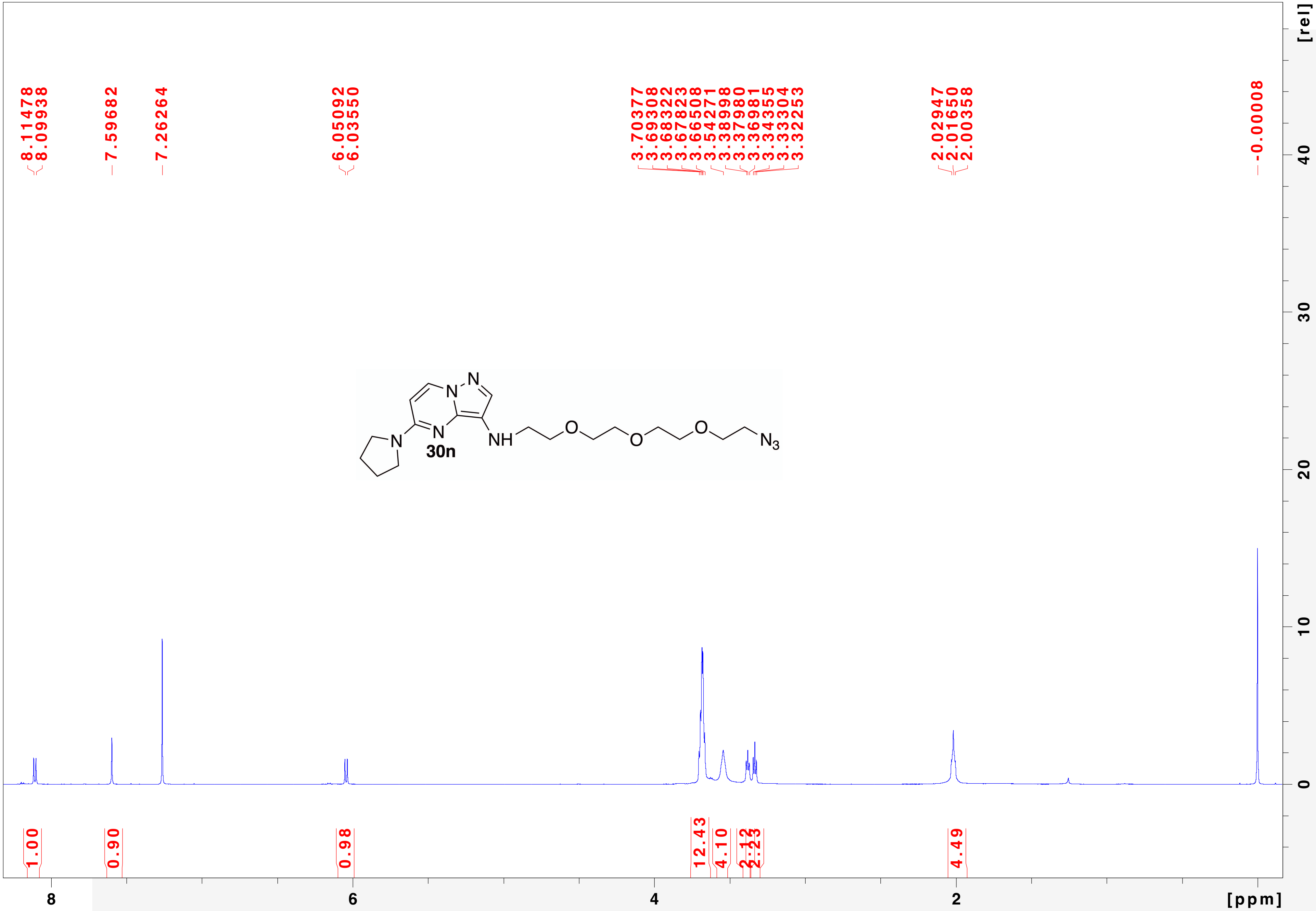

LG-I-43 40 1 /home/nmruser/networkdata/Bruker500Data/log96@chem.byu.edu

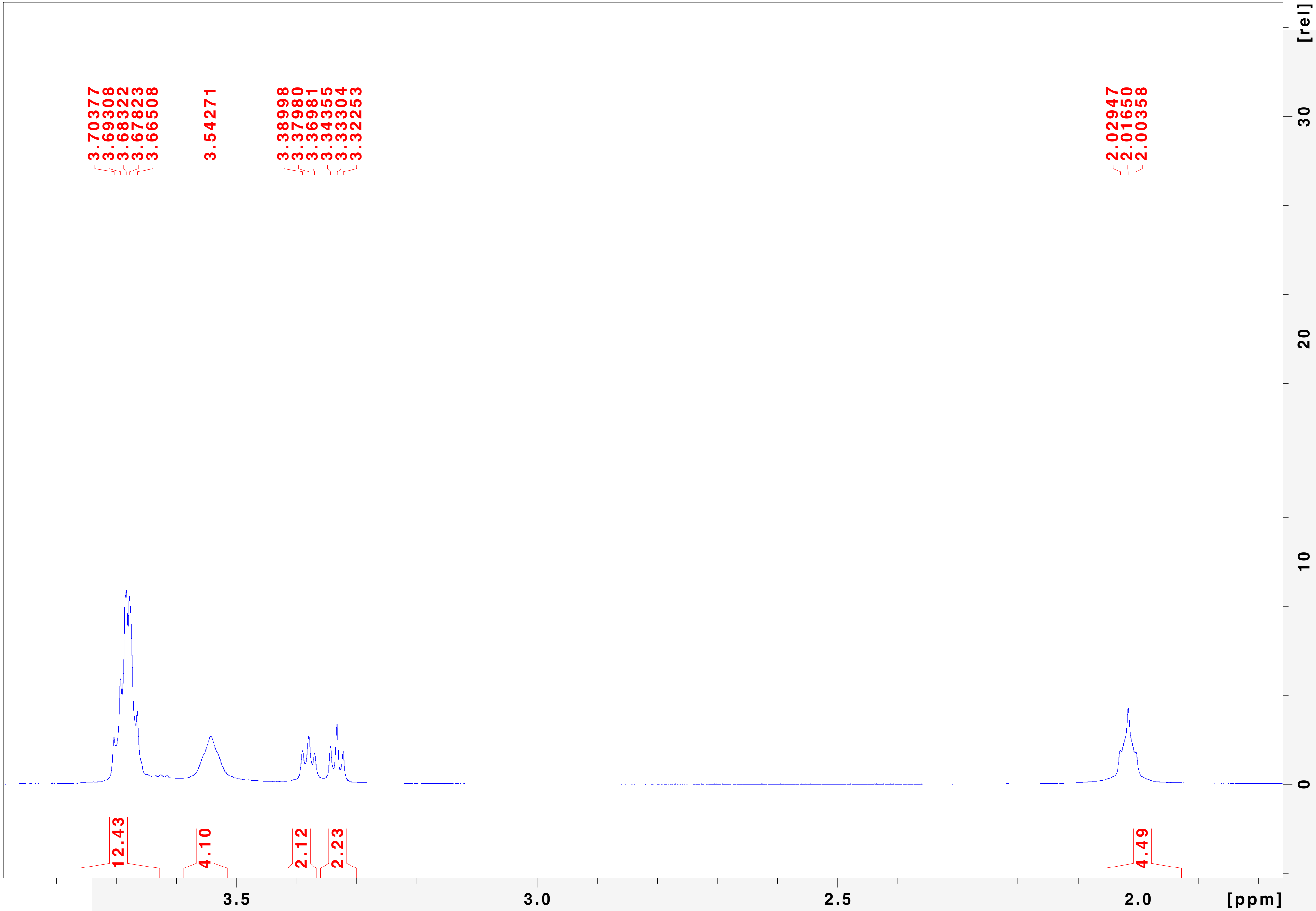

LG-I-43 50 1 /home/nmruser/networkdata/Bruker500Data/log96@chem.byu.edu

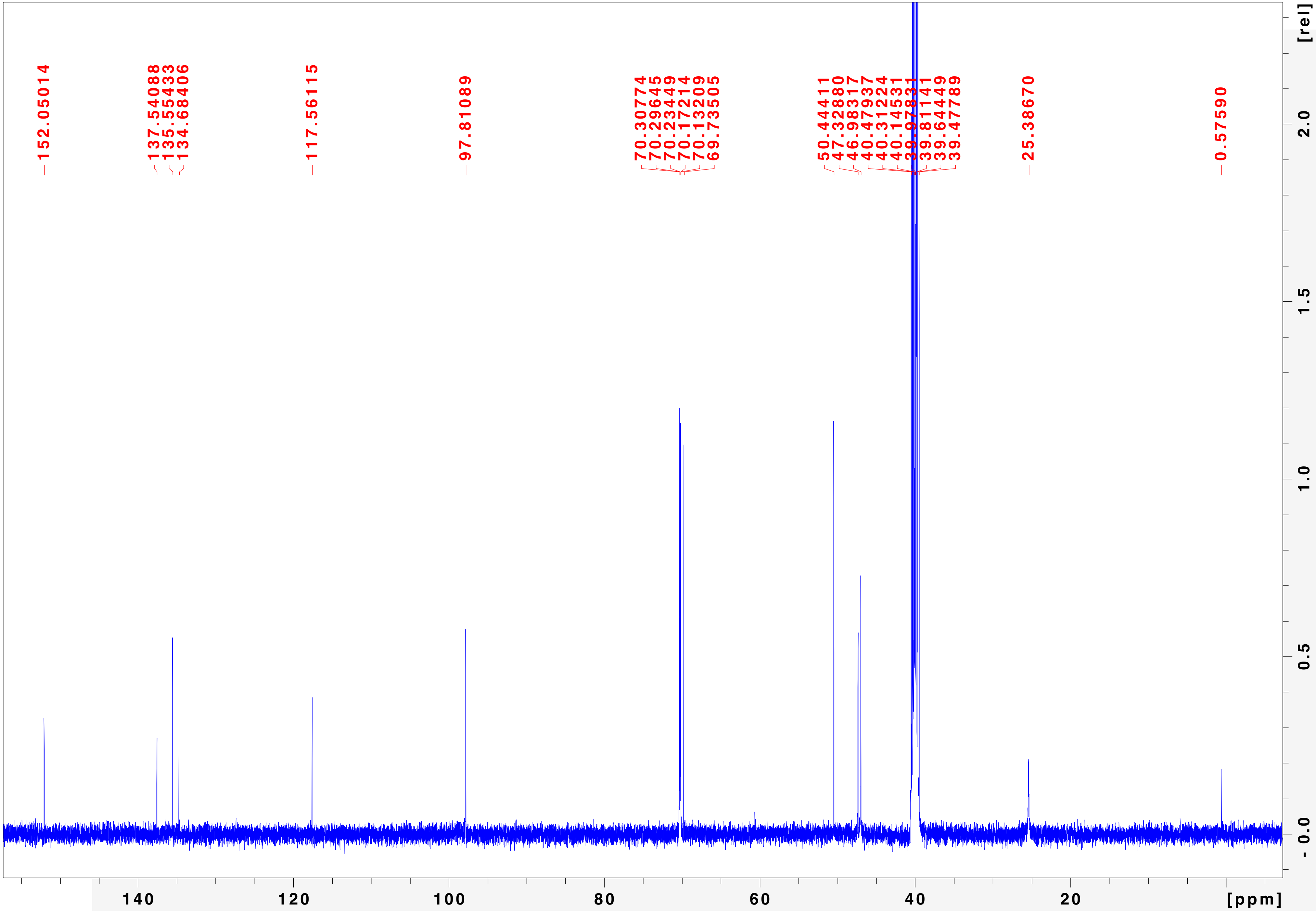

LG-I-43 50 1 /home/nmruser/networkdata/Bruker500Data/log96@chem.byu.edu

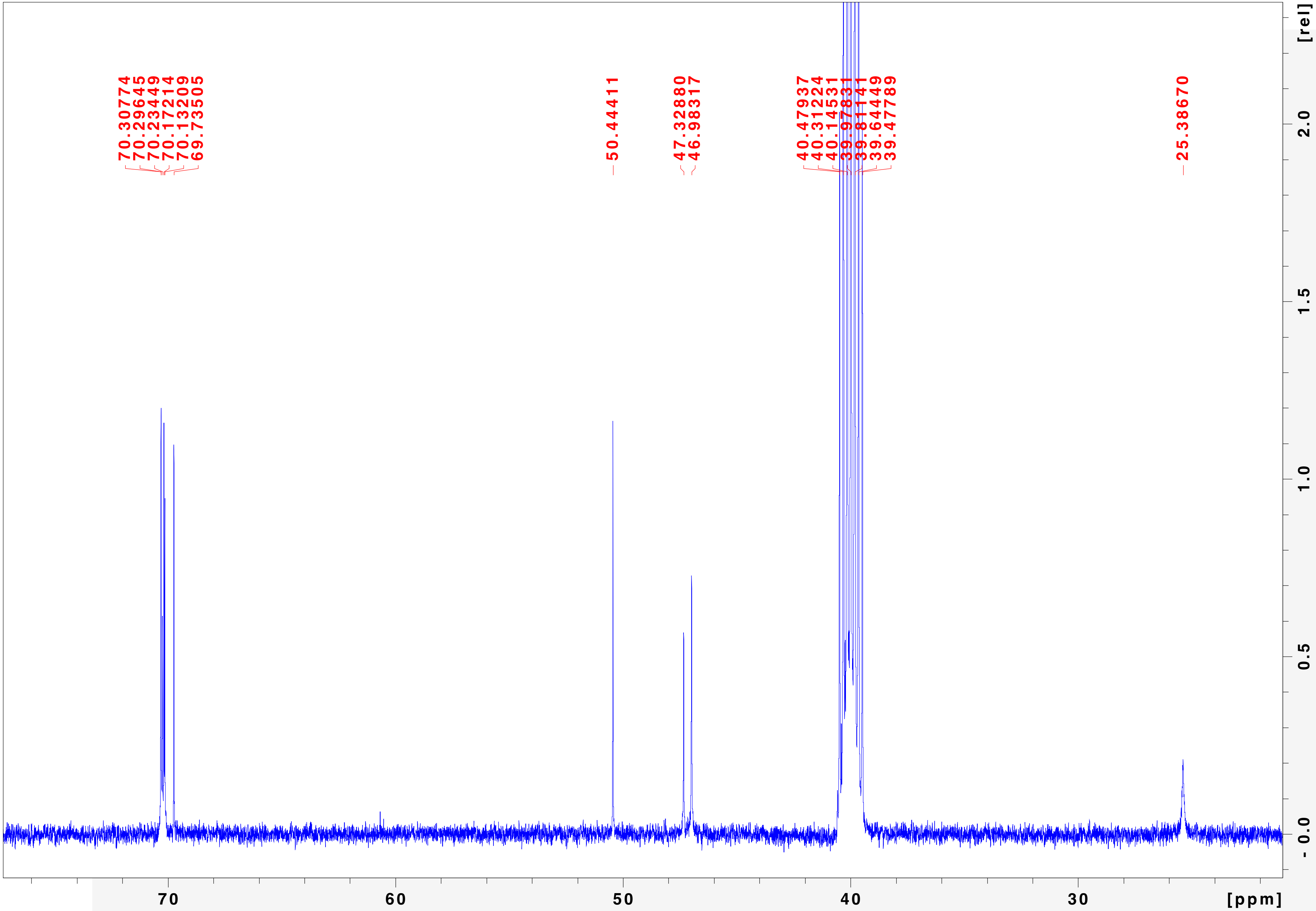

LG-I-43 50 1 /home/nmruser/networkdata/Bruker500Data/log96@chem.byu.edu

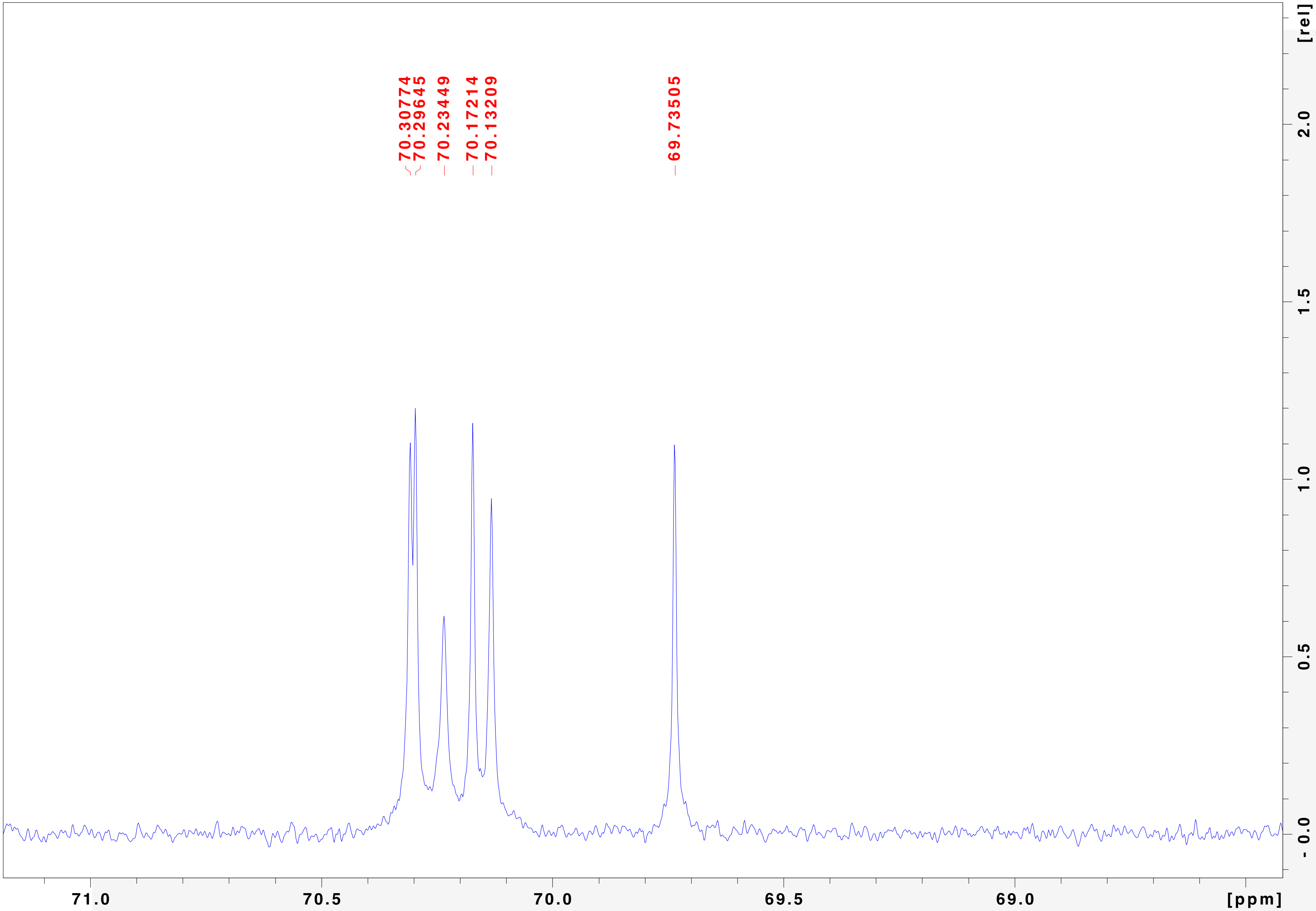

"BAT - II - 61" 1 1 /home/nmruser/networkdata/Bruker500Data/bat54@chem.byu.edu

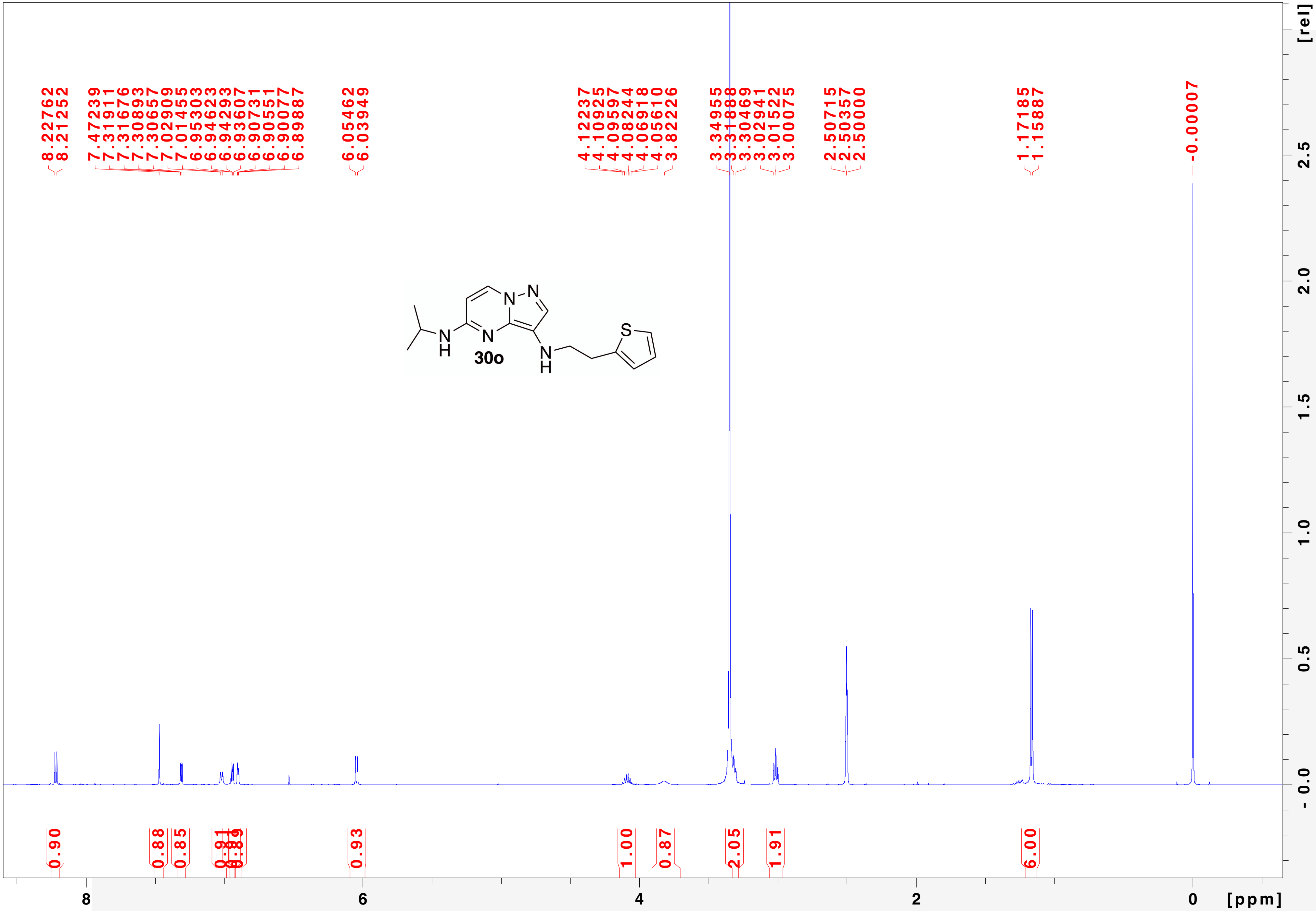

"BAT - II - 61" 1 1 /home/nmruser/networkdata/Bruker500Data/bat54@chem.byu.edu

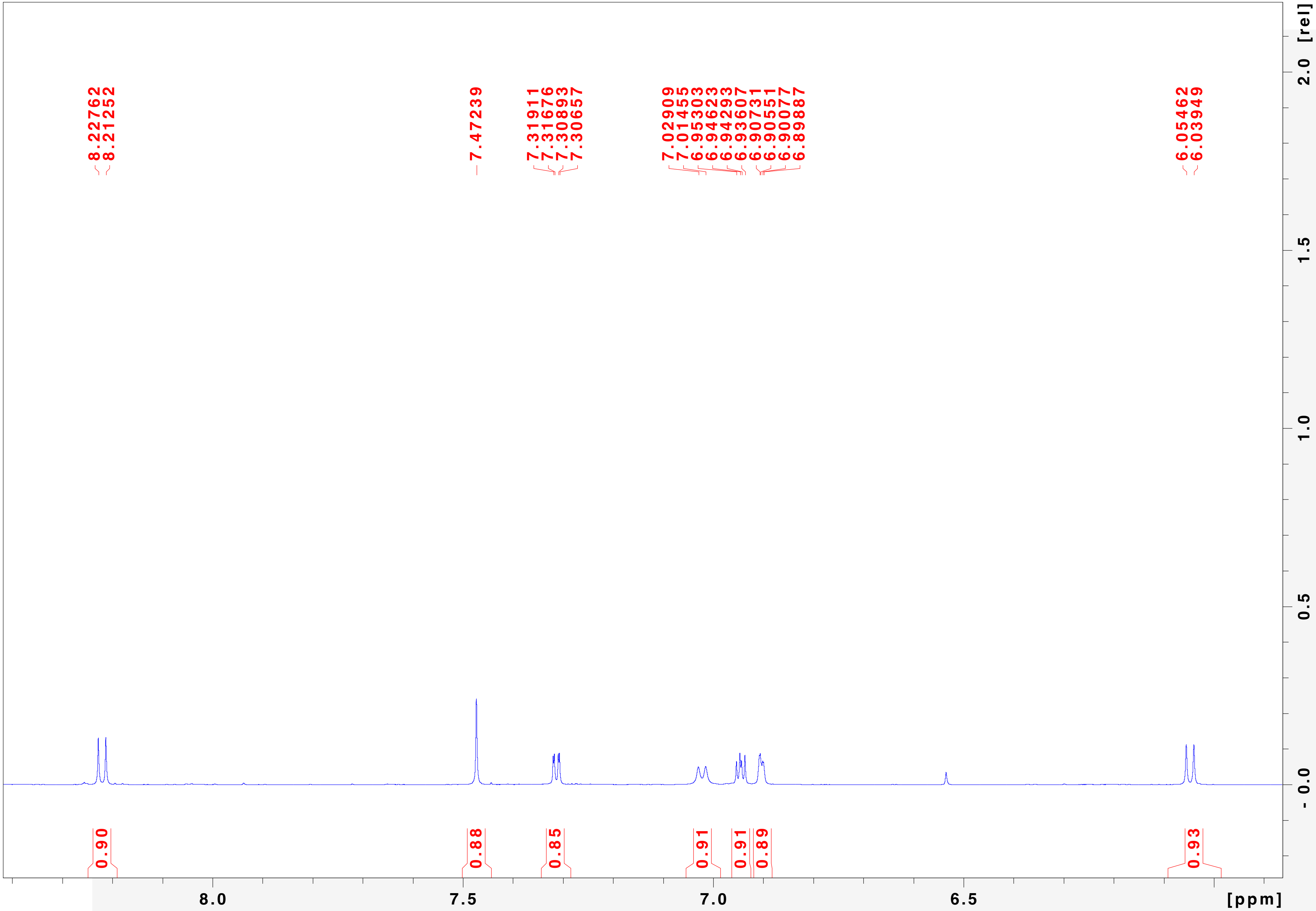

"BAT - II - 61" 1 1 /home/nmruser/networkdata/Bruker500Data/bat54@chem.byu.edu

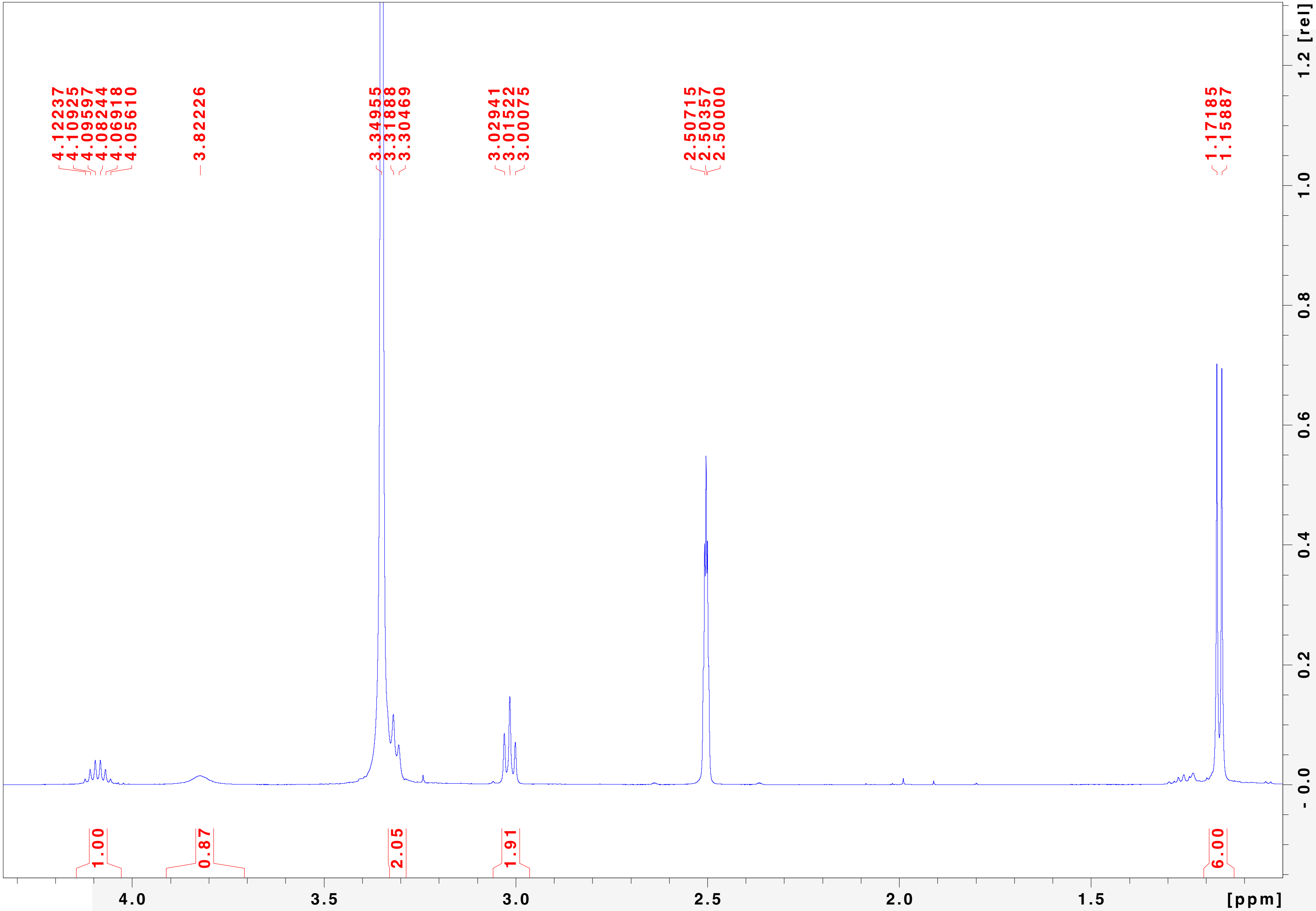

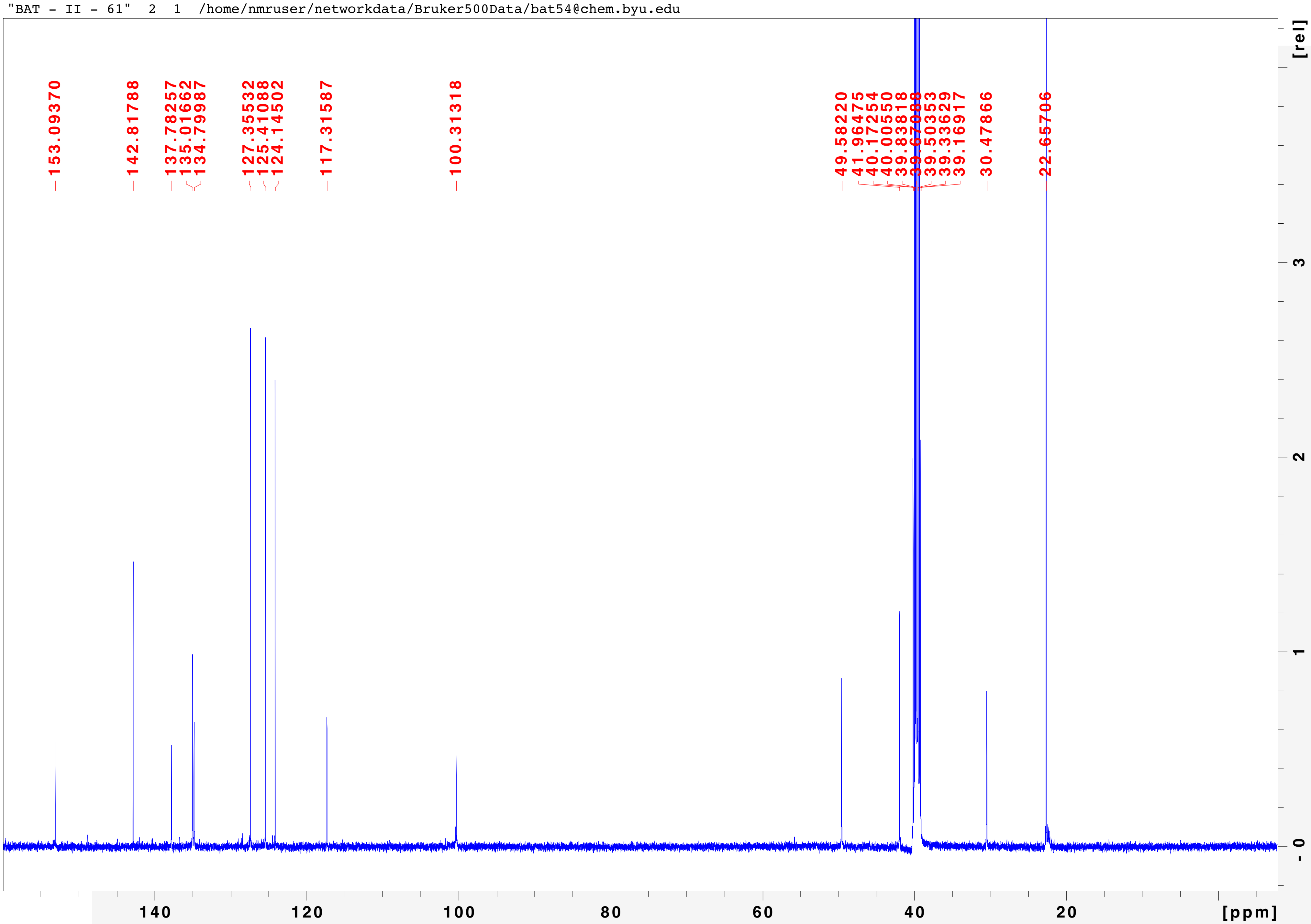

"BAT - II - 61" 2 1 /home/nmruser/networkdata/Bruker500Data/bat54@chem.byu.edu
153.09370
142.81788
137.78257
135.01662
134.79987
127.35532
125.41088
124.14502
117.31587
100.31318
49.58220
41.96475
40.17254
40.00550
39.83818
39.50353
39.33629
39.16917
30.47866
22.65706
[rel]
[ppm]

"BAT - II - 62" 1 1 /home/nmruser/networkdata/Bruker500Data/bat54@chem.byu.edu

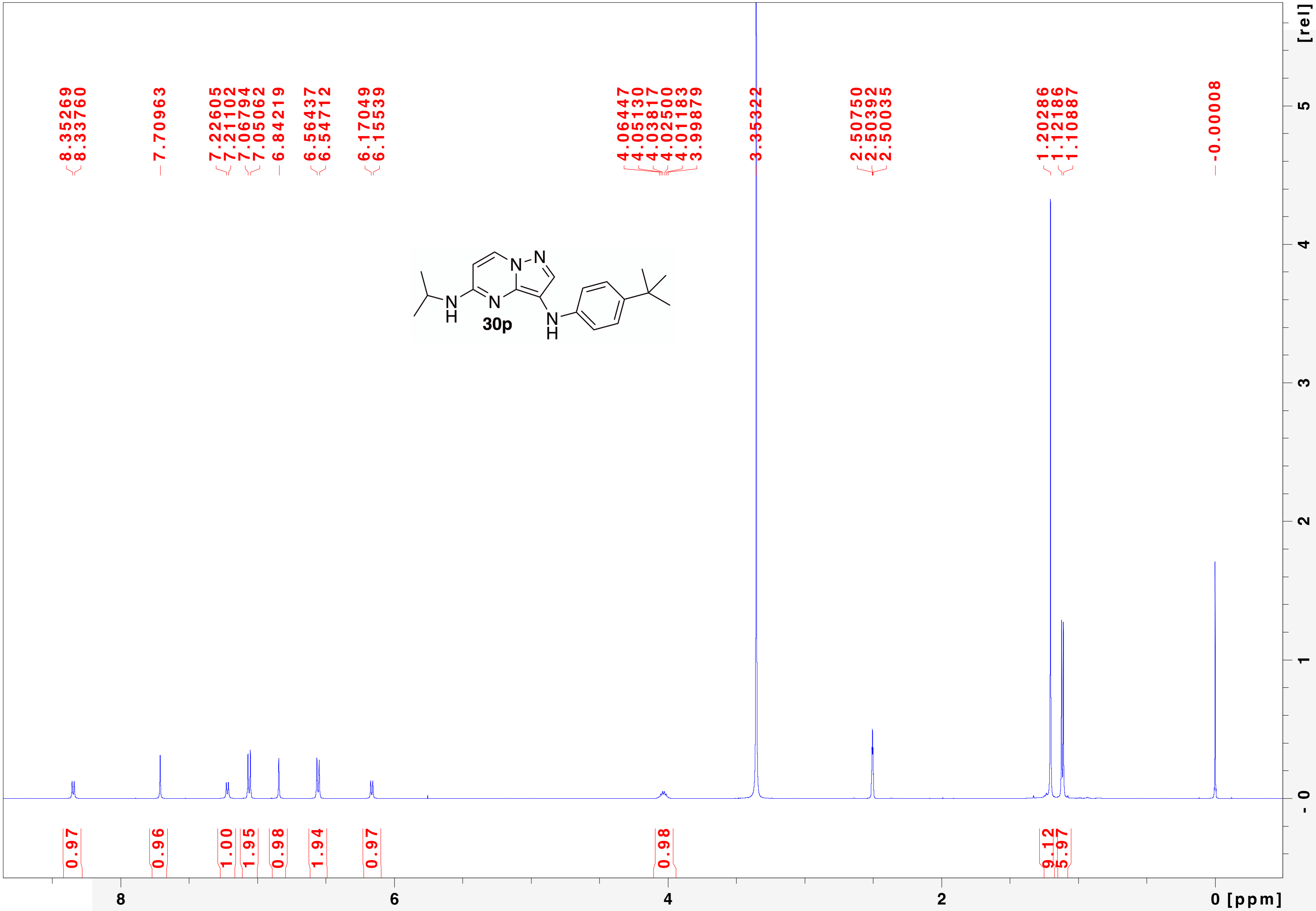

"BAT - II - 62" 1 1 /home/nmruser/networkdata/Bruker500Data/bat54@chem.byu.edu

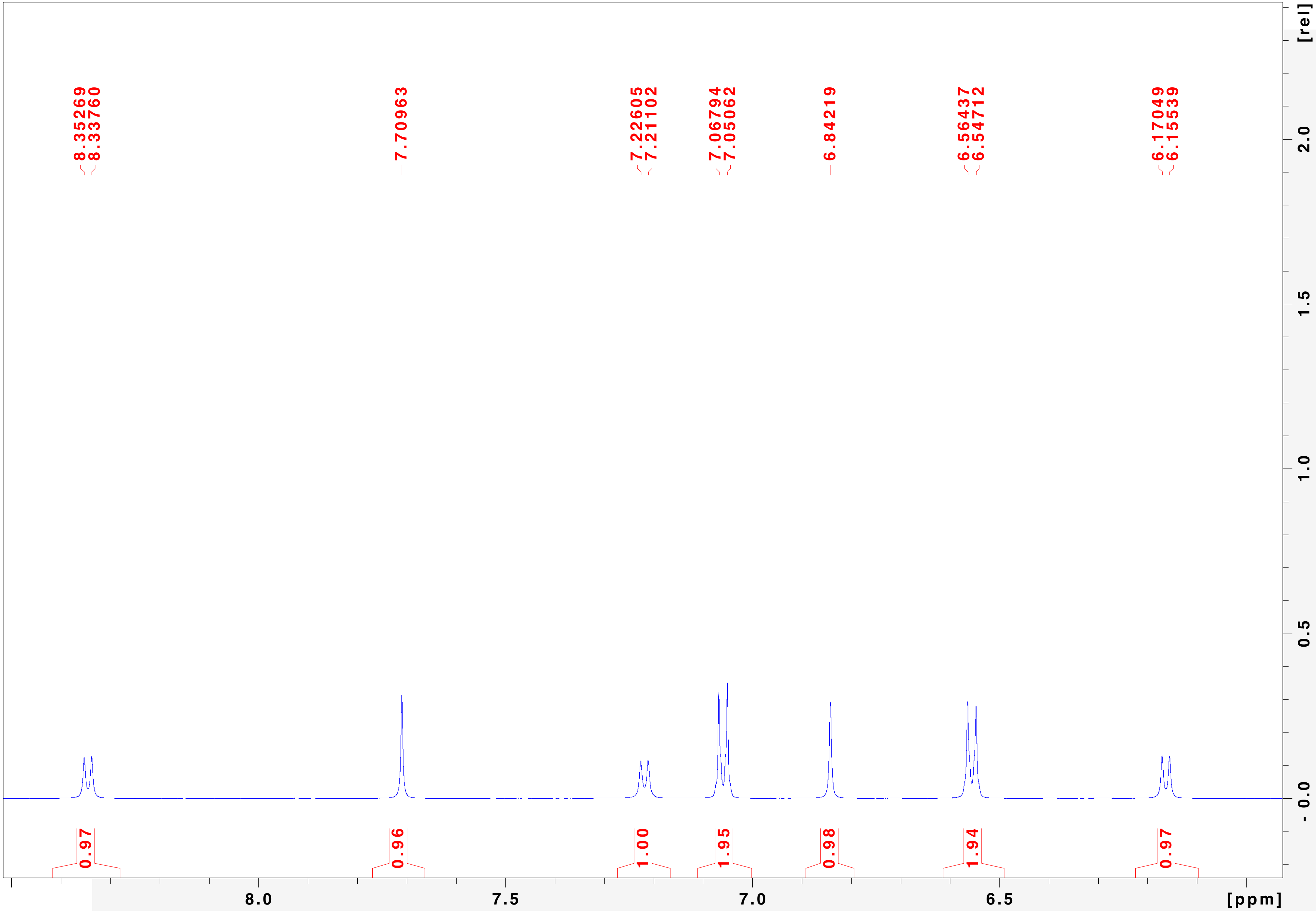

"BAT - II - 62" 1 1 /home/nmruser/networkdata/Bruker500Data/bat54@chem.byu.edu

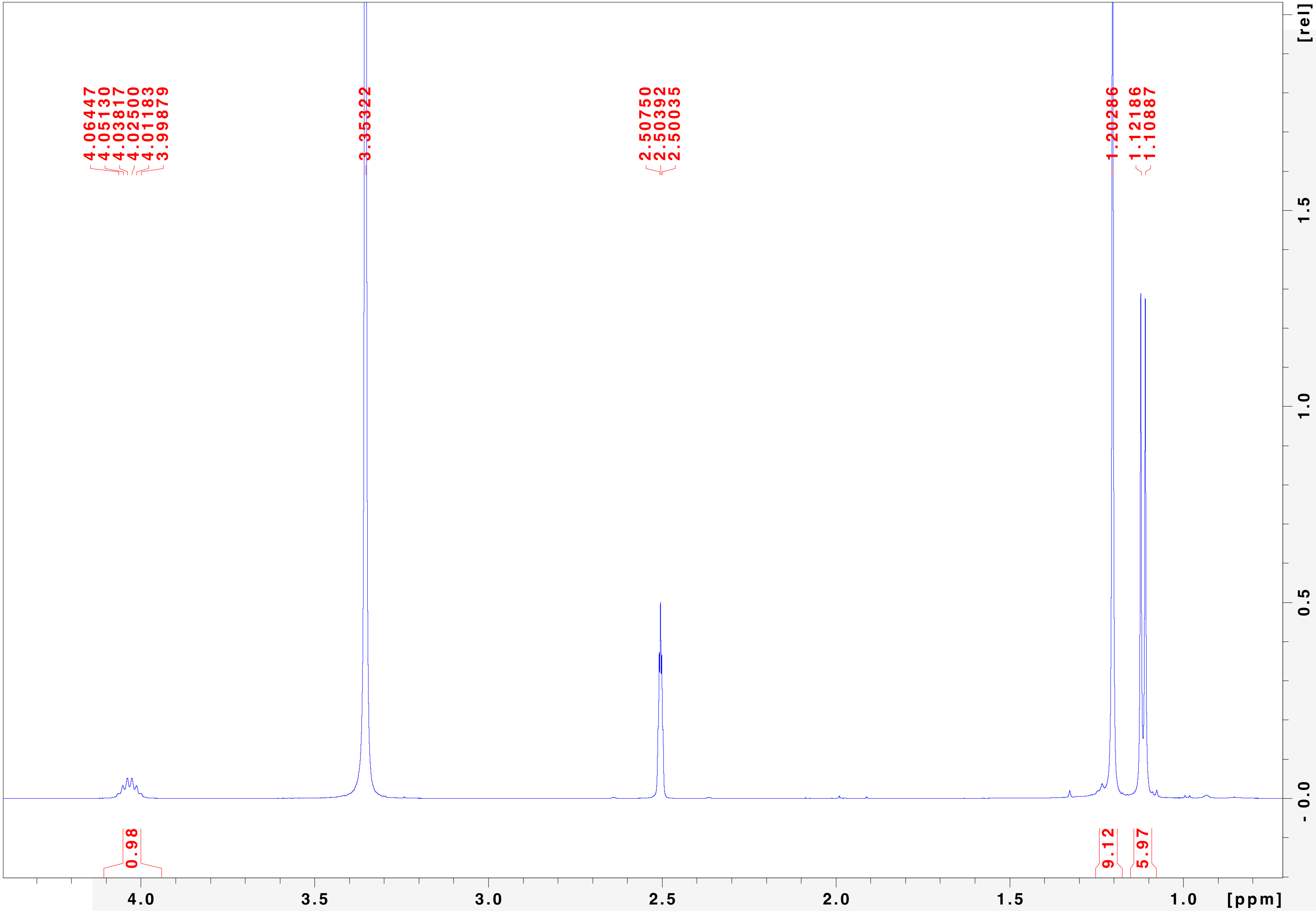

"BAT - II - 62" 2 1 /home/nmruser/networkdata/Bruker500Data/bat54@chem.byu.edu

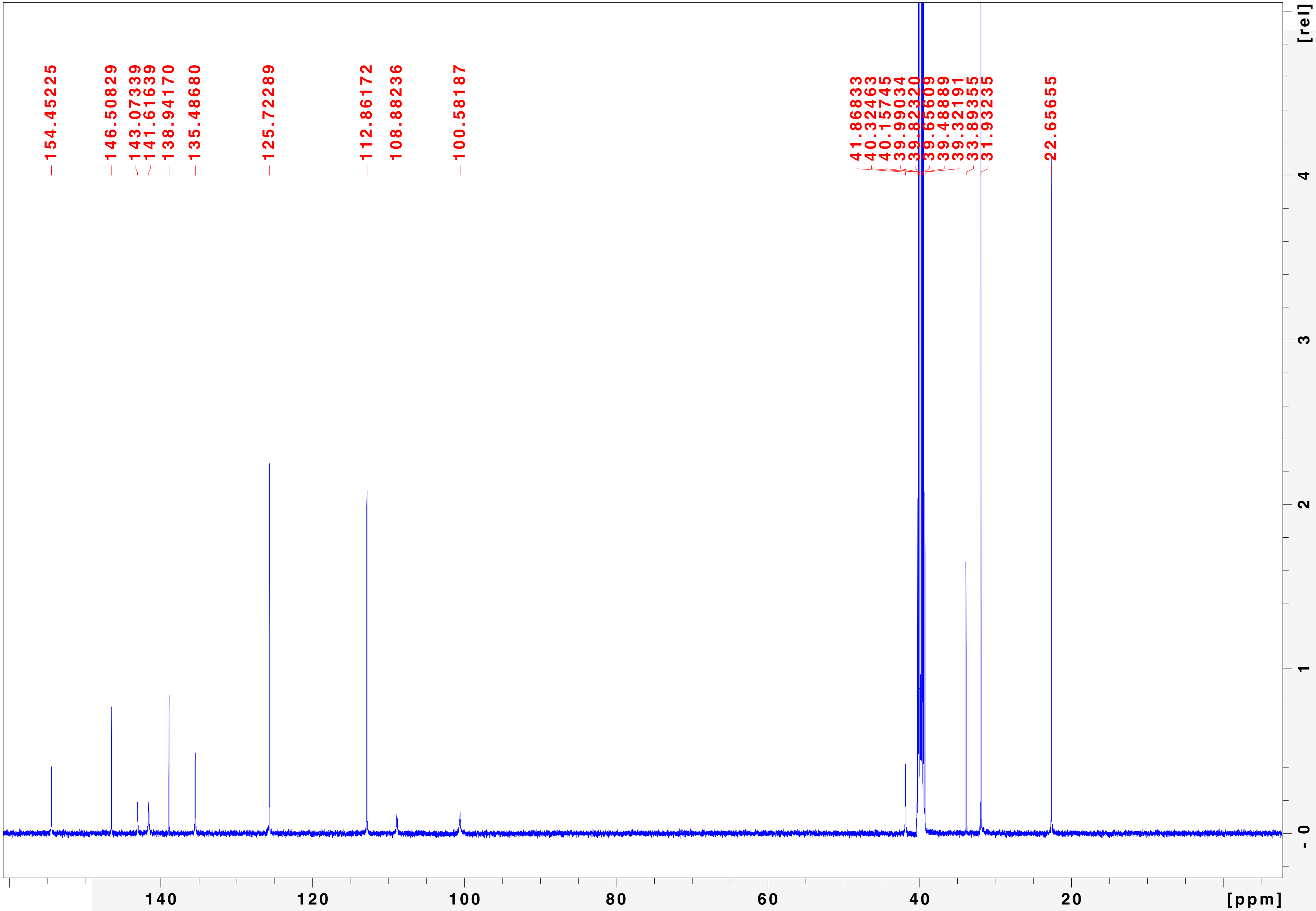

"BAT - II - 60" 1 1 /home/nmruser/networkdata/Bruker500Data/bat54@chem.byu.edu

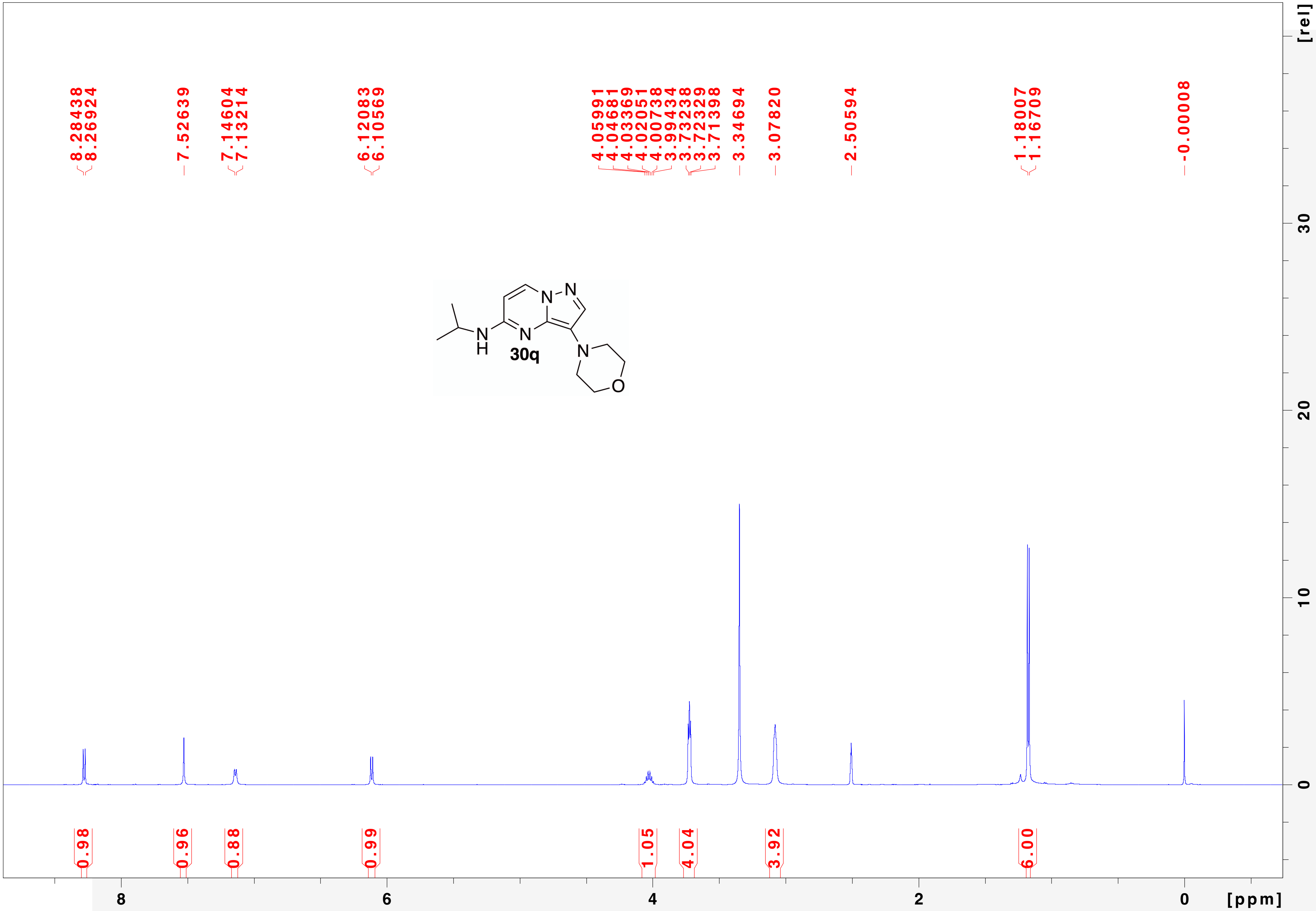

"BAT - II - 60" 1 1 /home/nmruser/networkdata/Bruker500Data/bat54@chem.byu.edu

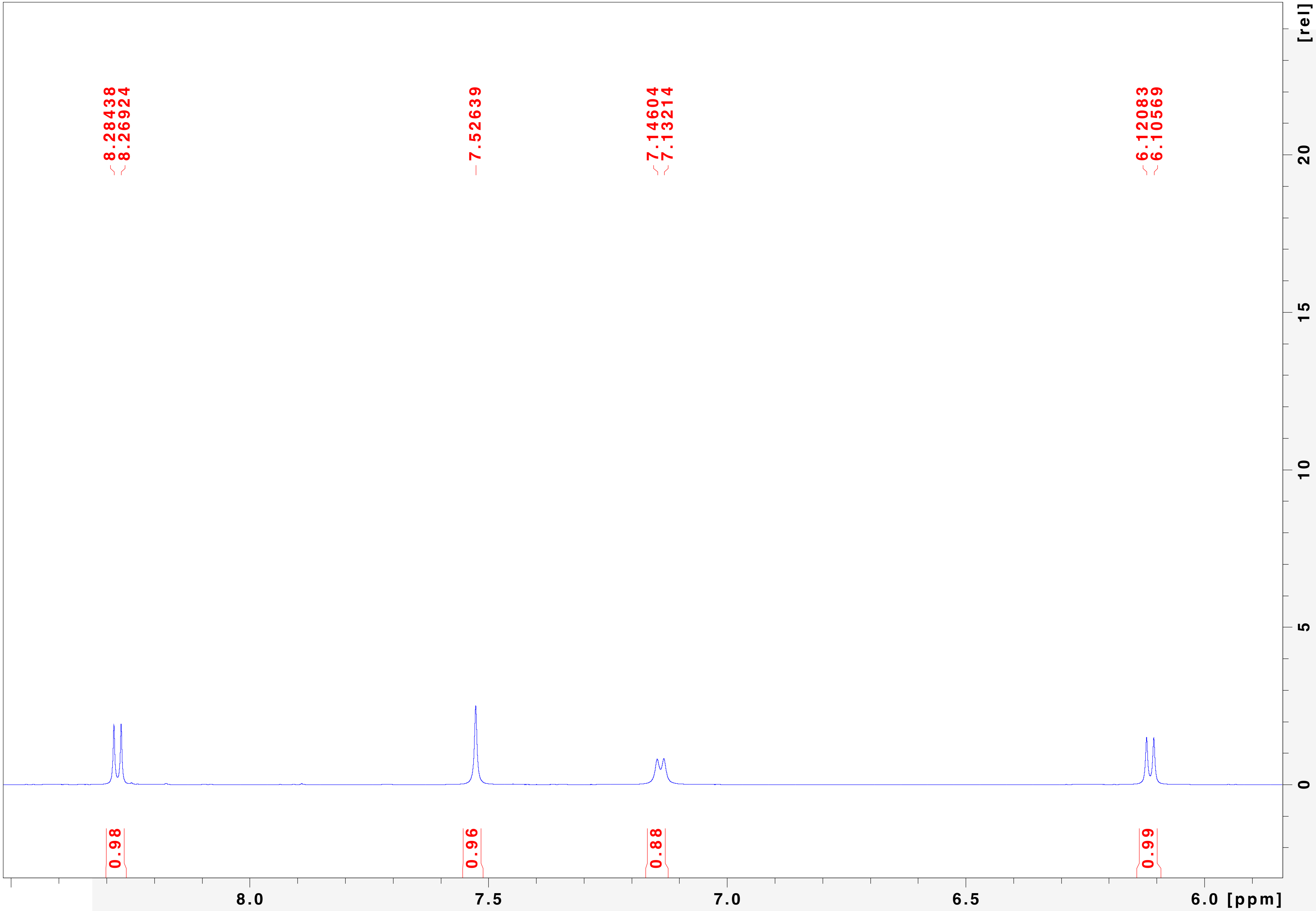

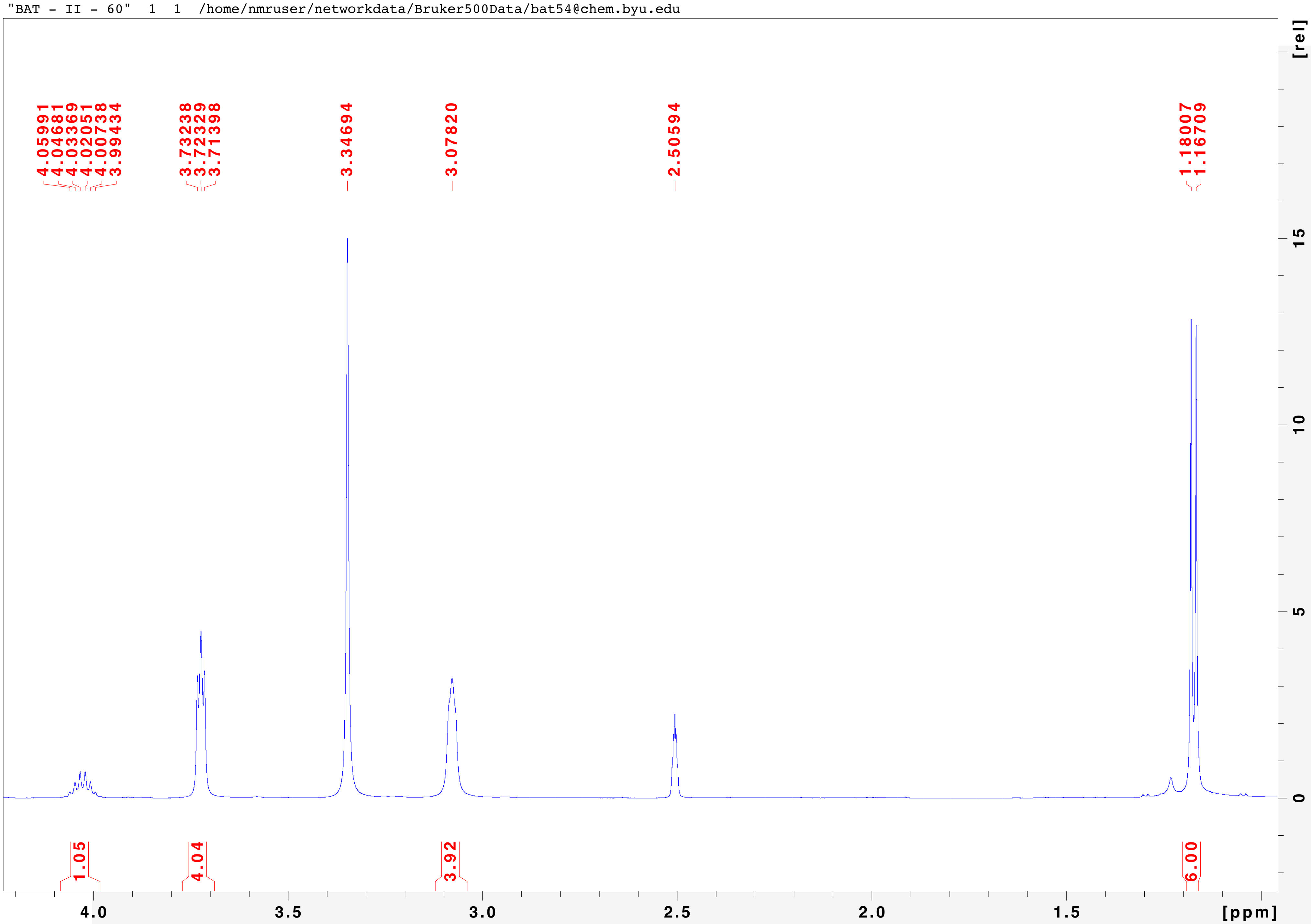

"BAT - II - 60" 1 1 /home/nmruser/networkdata/Bruker500Data/bat54@chem.byu.edu
4.05991
4.04681
4.03369
4.02051
4.00738
3.99434
3.73238
3.72329
3.71398
3.34694
3.07820
2.50594
1.18007
1.16709
1.05
4.04
3.92
6.00
[rel]
15
10
5
0
4.0
3.5
3.0
2.5
2.0
1.5
[ppm]

"BAT - II - 60" 2 1 /home/nmruser/networkdata/Bruker500Data/bat54@chem.byu.edu

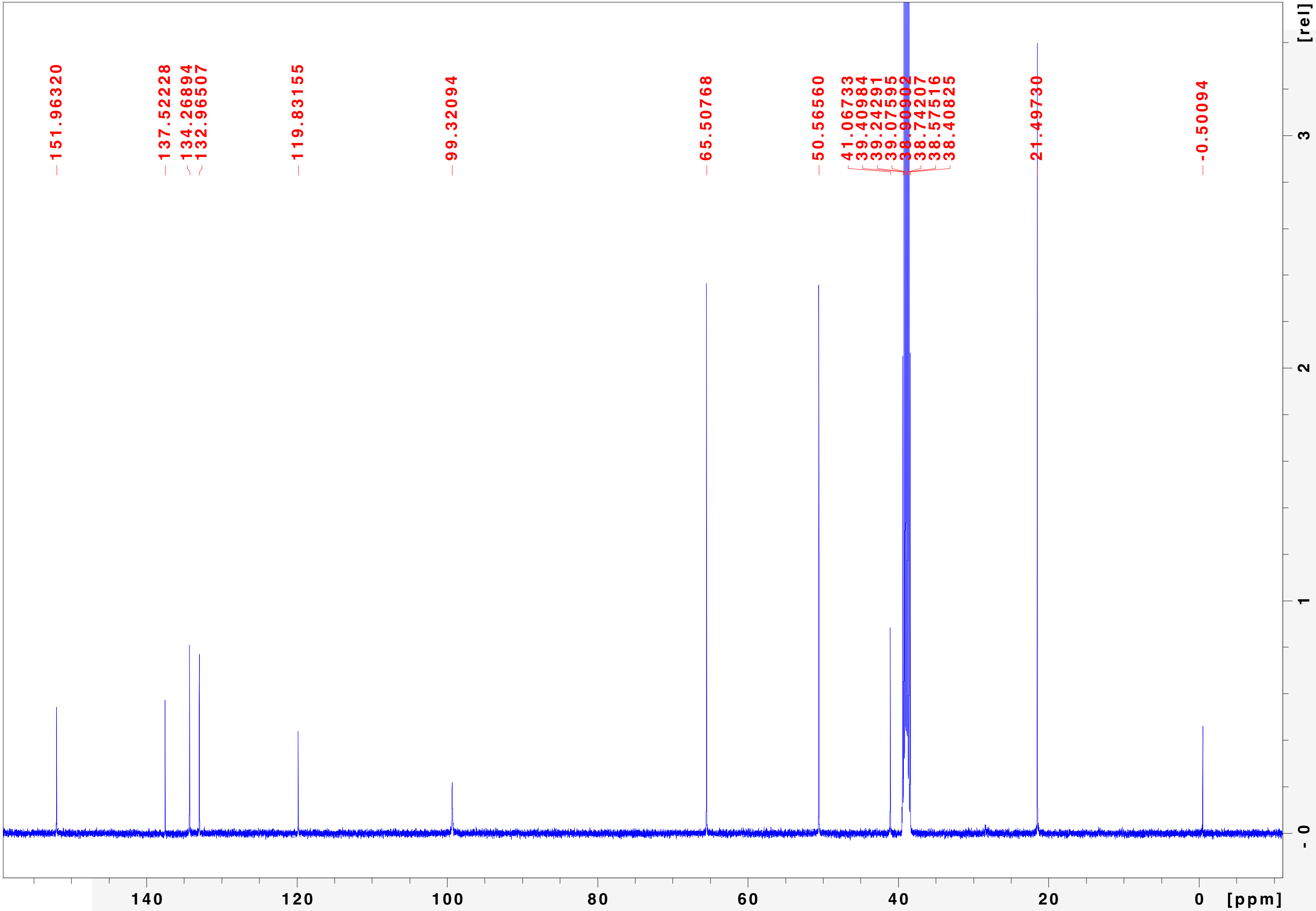

JI-III-53 10 1 /home/nmruser/networkdata/Bru500/terungwa@chem.byu.edu

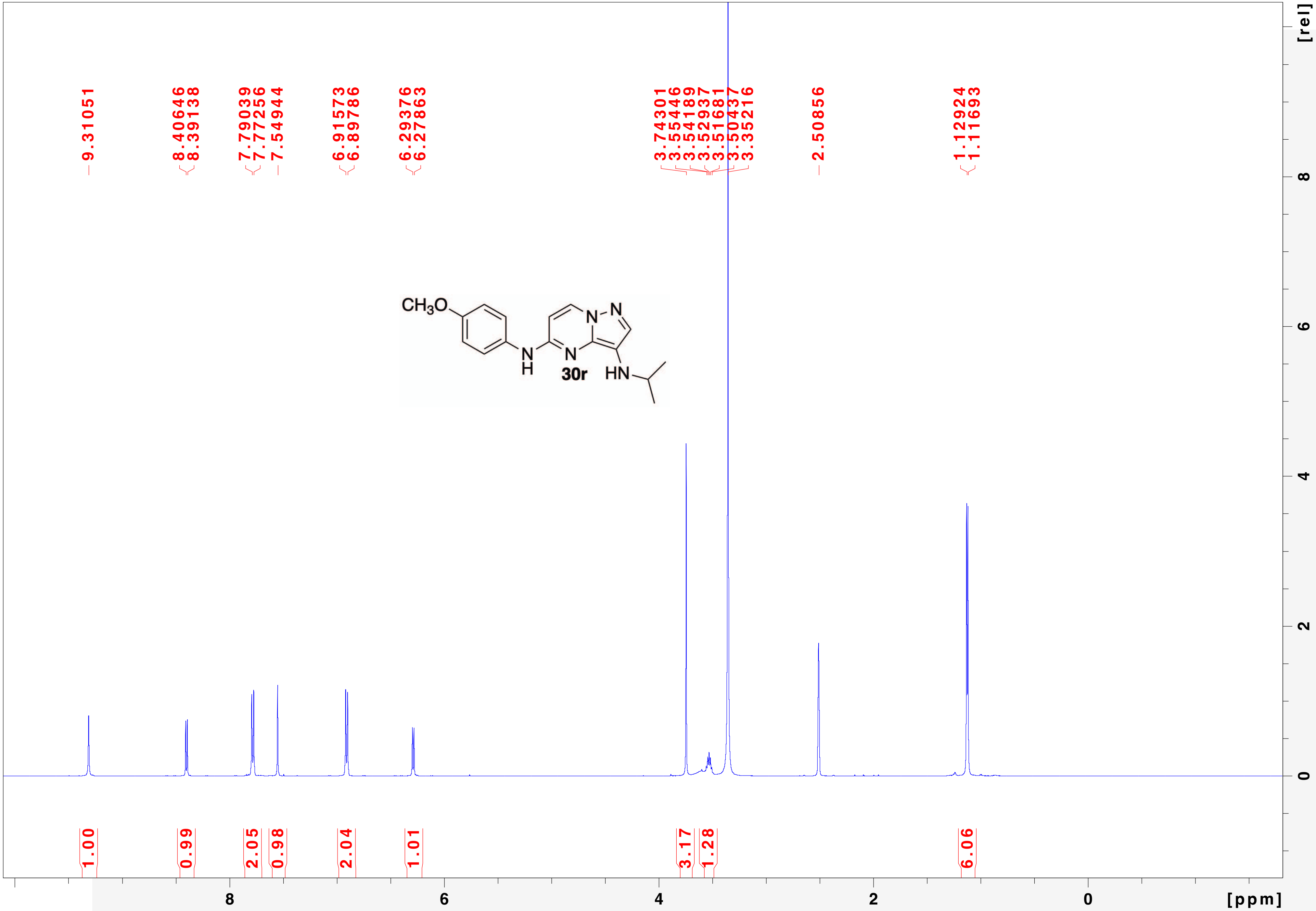

JI-III-53 10 1 /home/nmruser/networkdata/Bru500/terungwa@chem.byu.edu

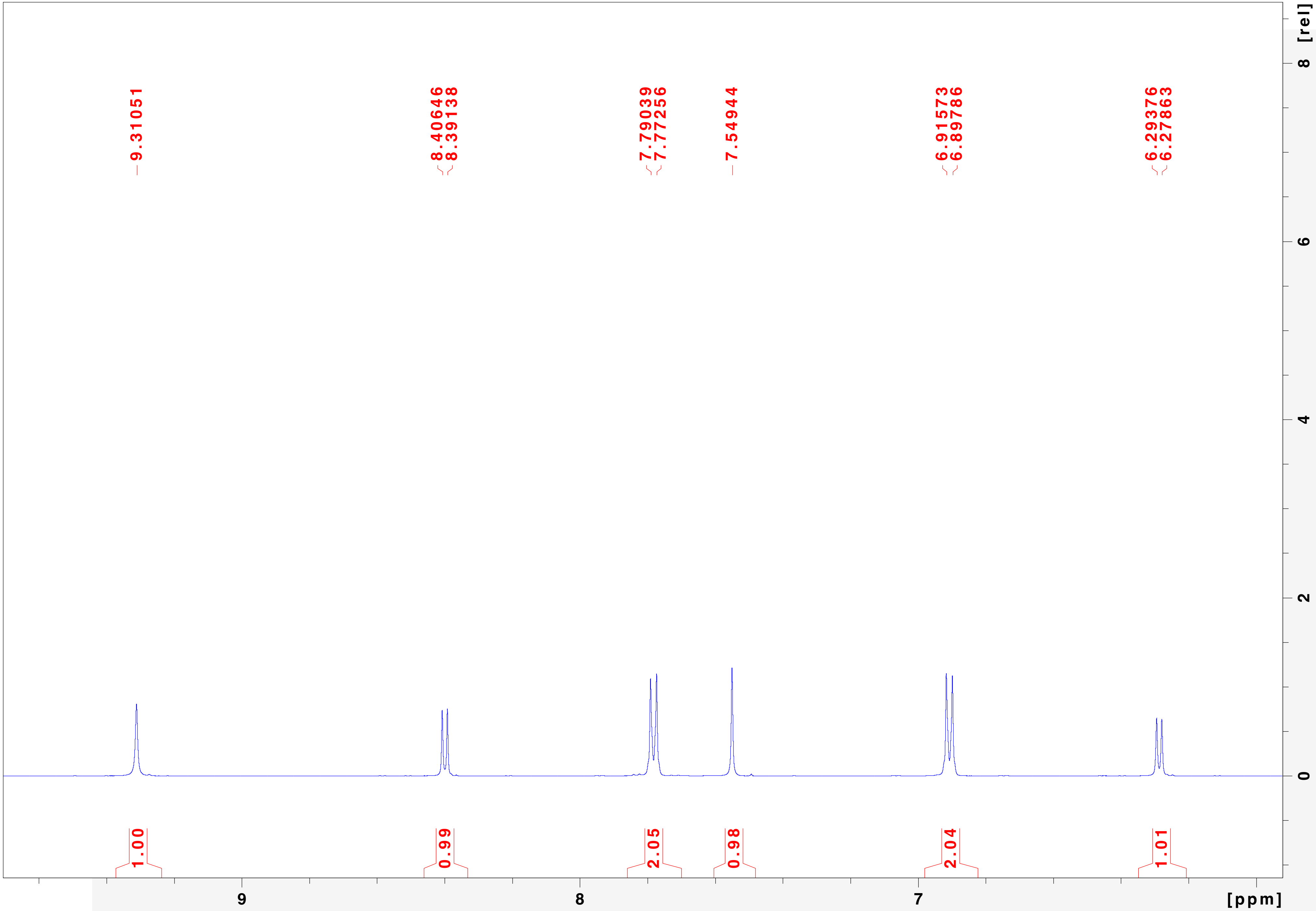

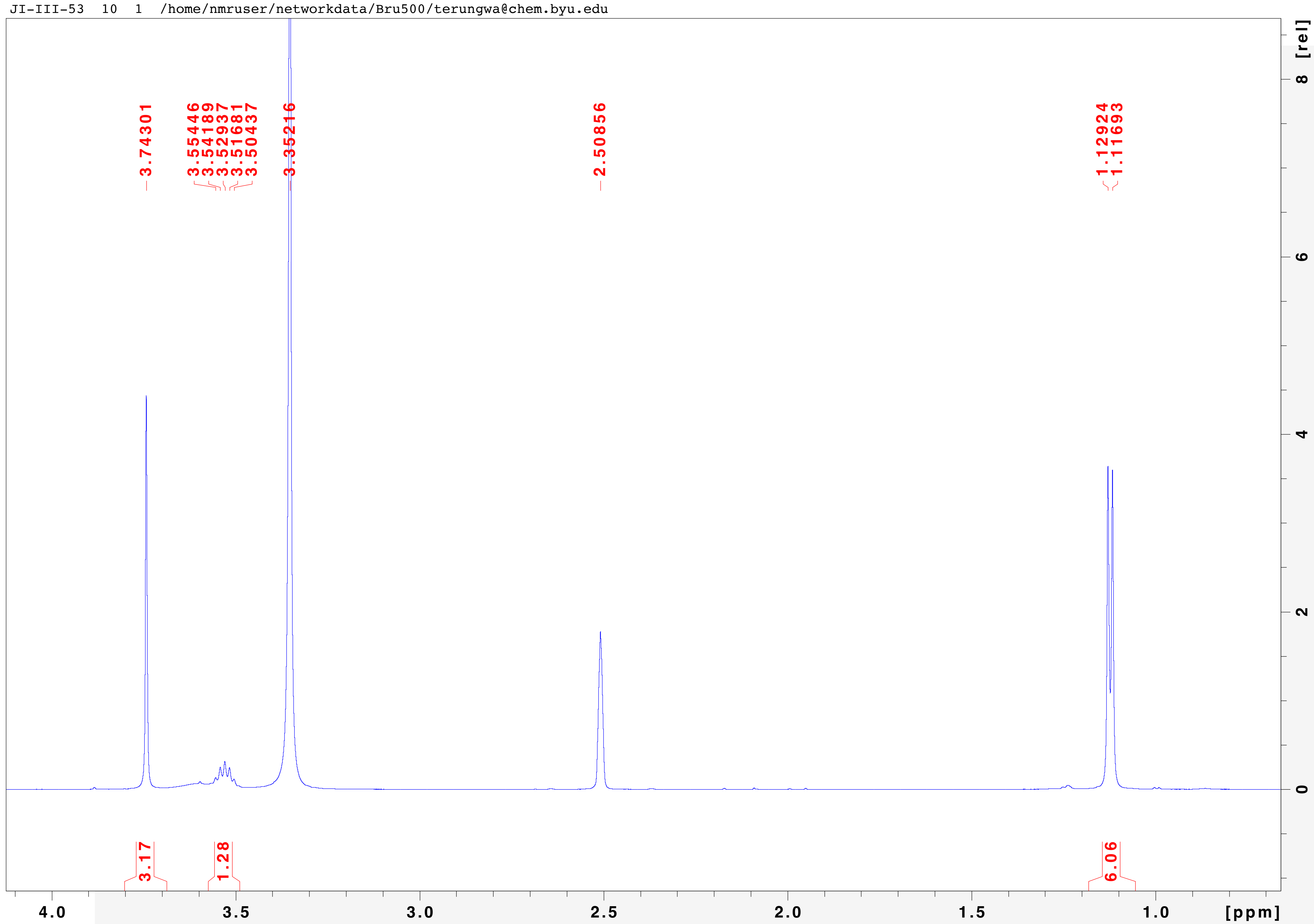
JI-III-53 10 1 /home/nmruser/networkdata/Bru500/terungwa@chem.byu.edu
3.74301
3.55446
3.54189
3.52937
3.51681
3.50437
3.35216
2.50856
1.12924
1.11693
3.17
1.28
6.06
4.0
3.5
3.0
2.5
2.0
1.5
1.0
[ppm]
8
6
4
2
0
[rel]

JI-III-53 11 1 /home/nmruser/networkdata/Bru500/terungwa@chem.byu.edu

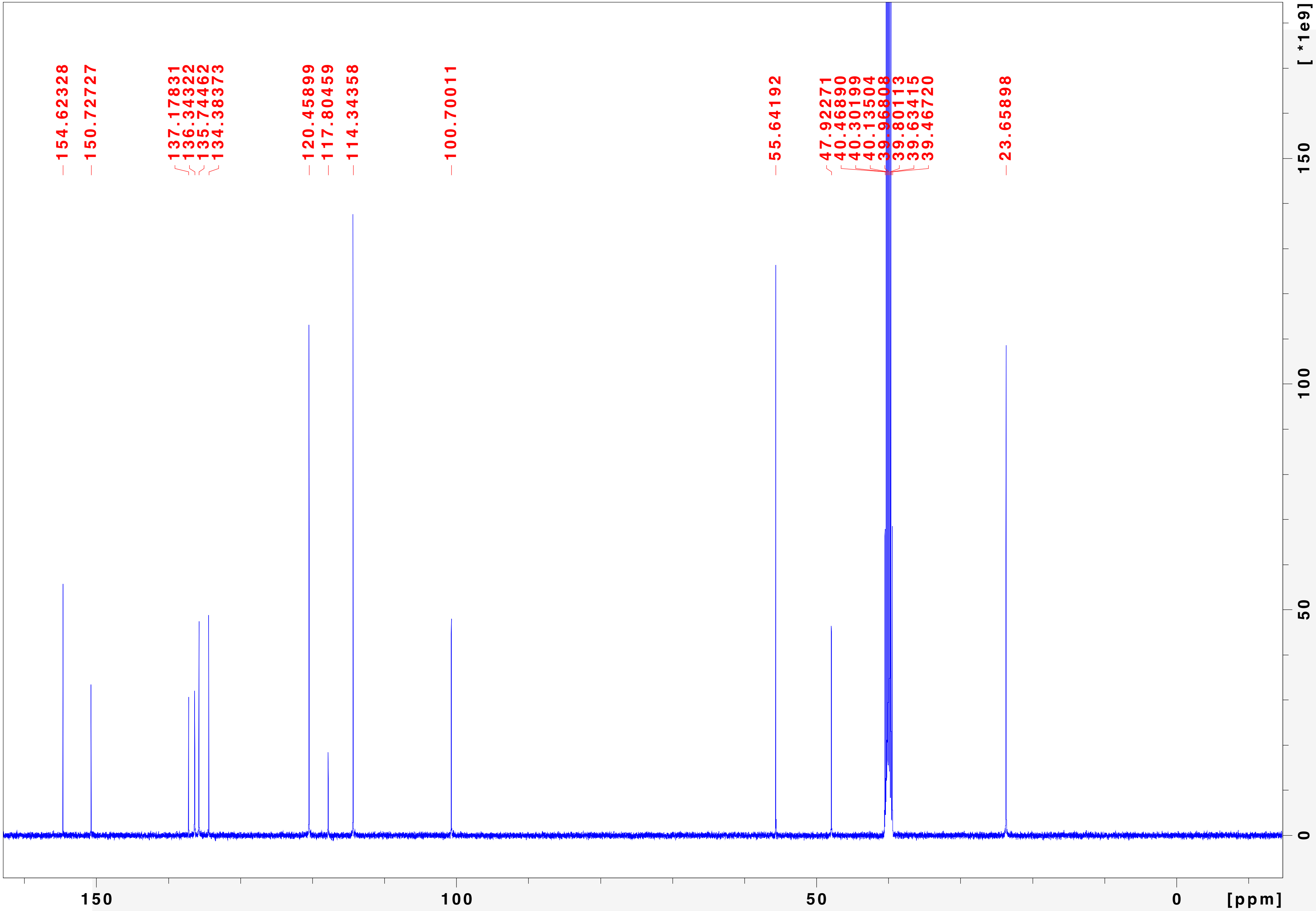

JI-III-47 10 1 /home/nmruser/networkdata/Bru500/terungwa@chem.byu.edu

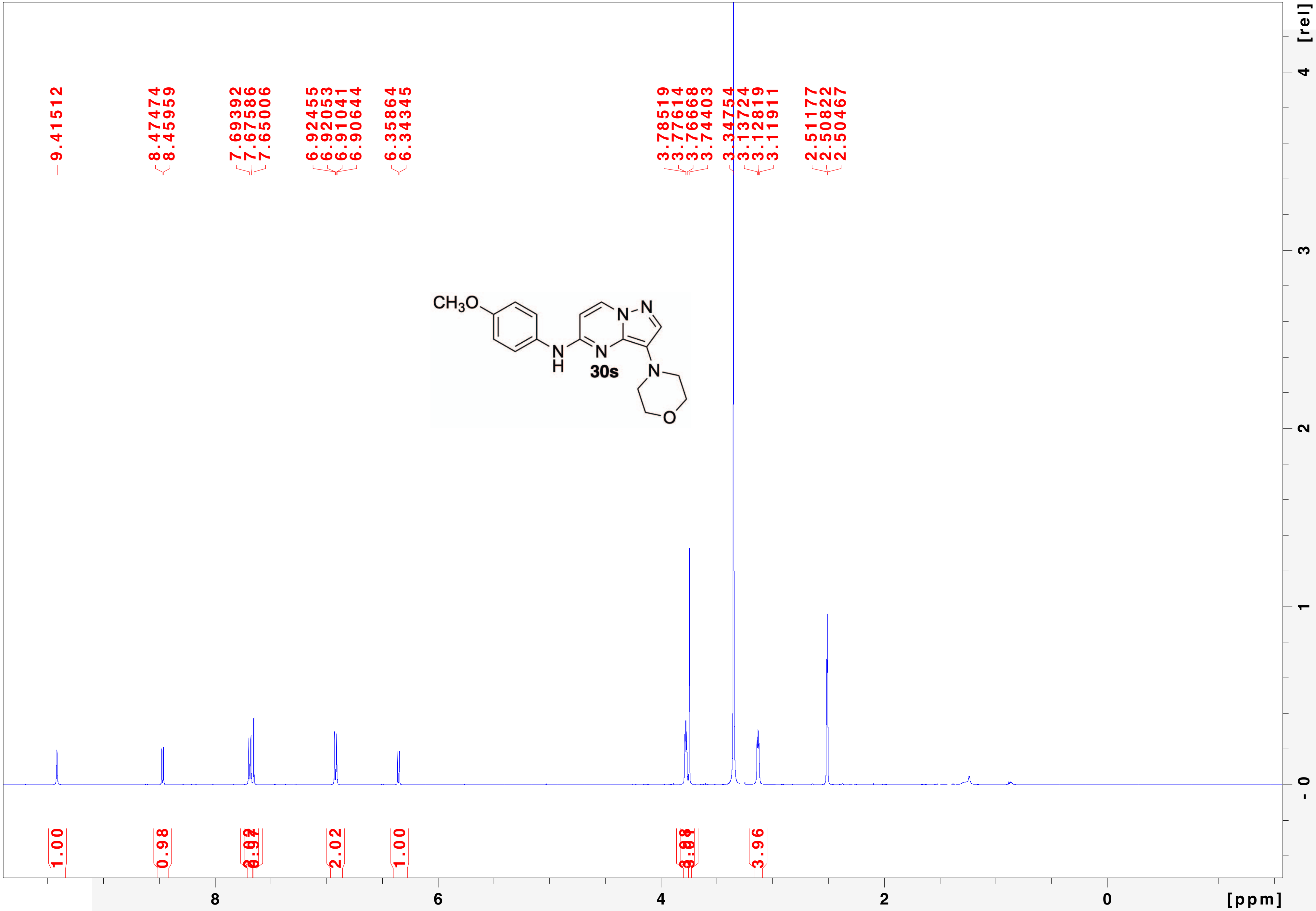

JI-III-47 10 1 /home/nmruser/networkdata/Bru500/terungwa@chem.byu.edu

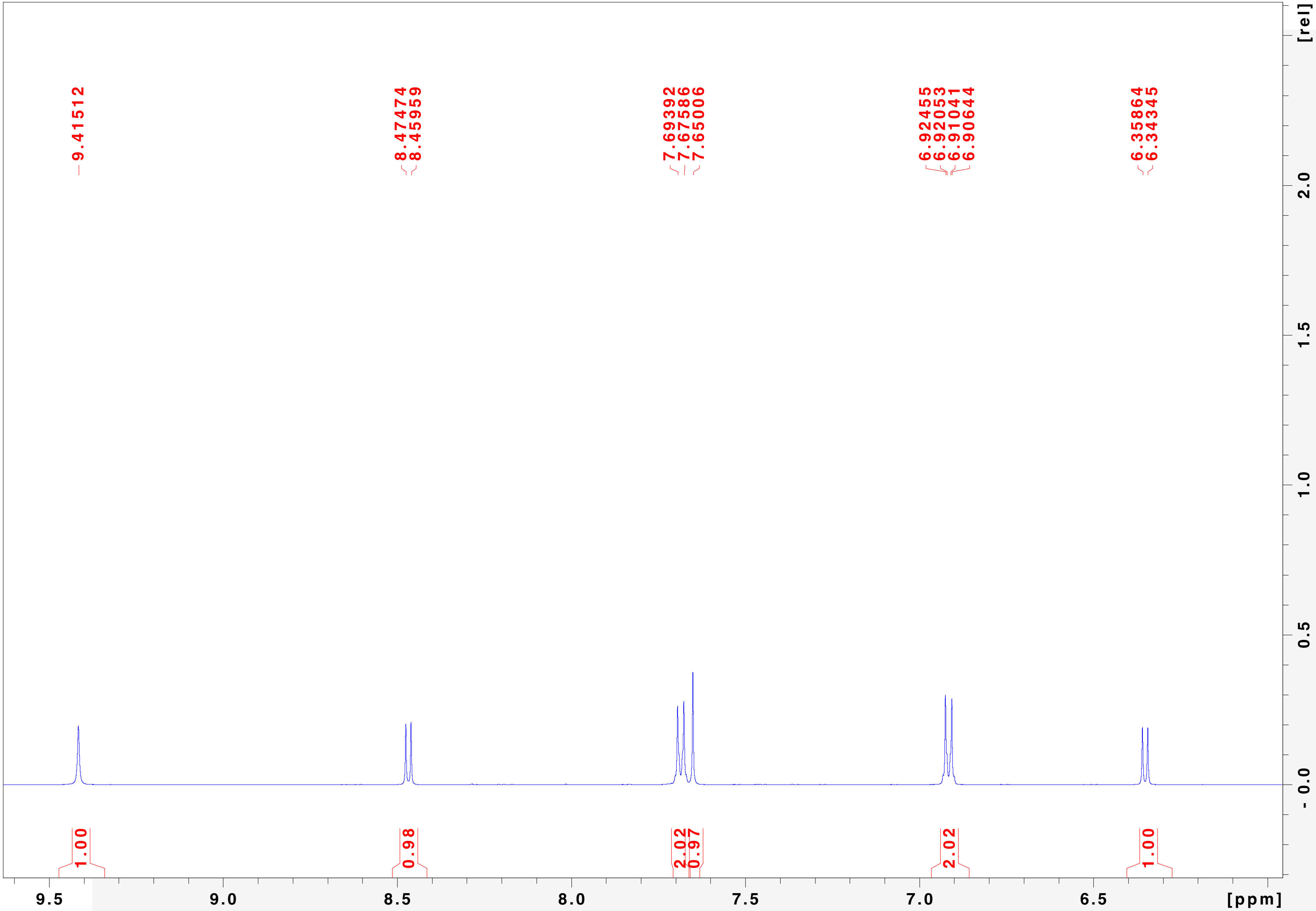

JI-III-47 10 1 /home/nmruser/networkdata/Bru500/terungwa@chem.byu.edu

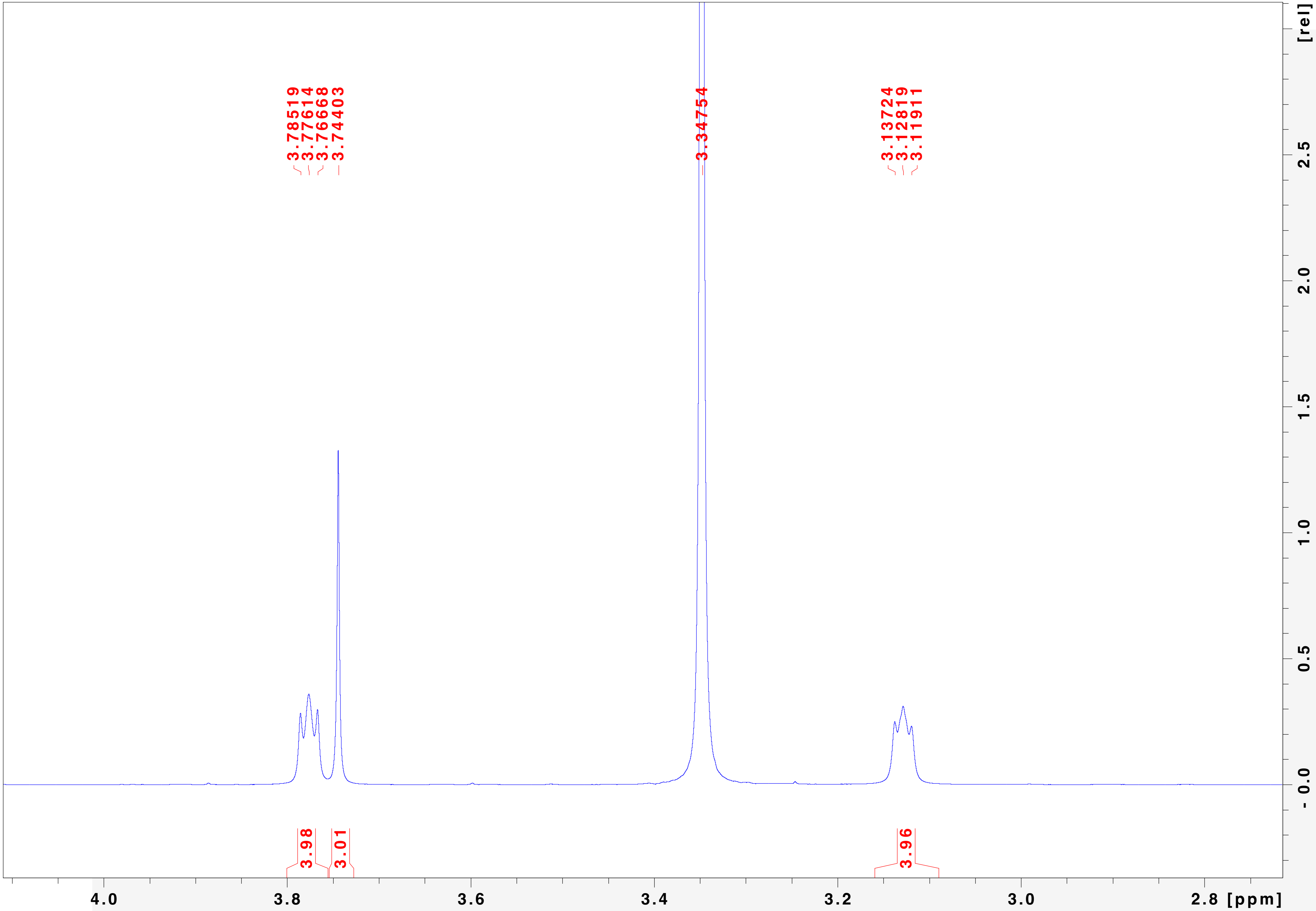

JI-III-47 11 1 /home/nmruser/networkdata/Bru500/terungwa@chem.byu.edu

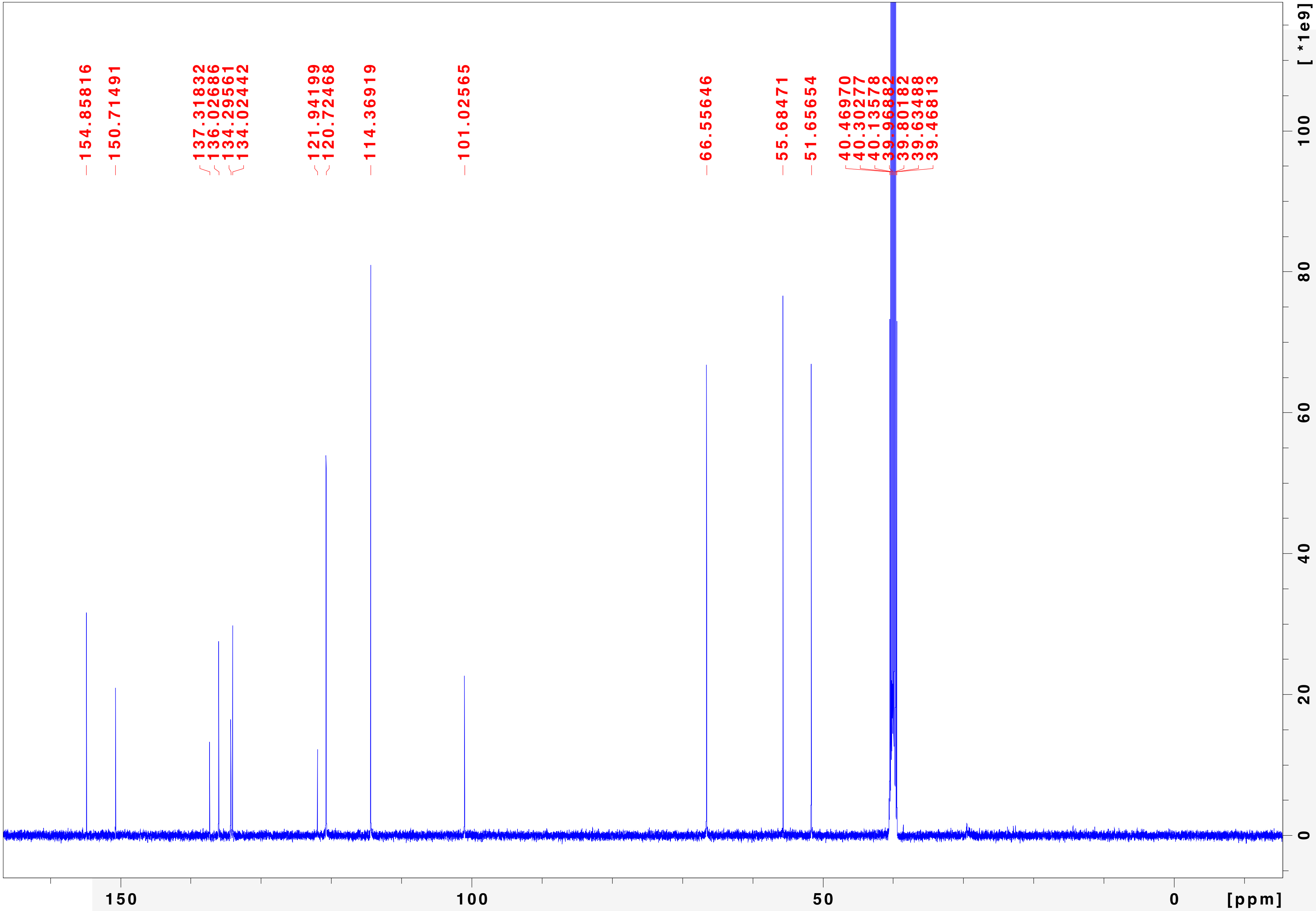

JI-III-54 10 1 /home/nmruser/networkdata/Bru500/terungwa@chem.byu.edu

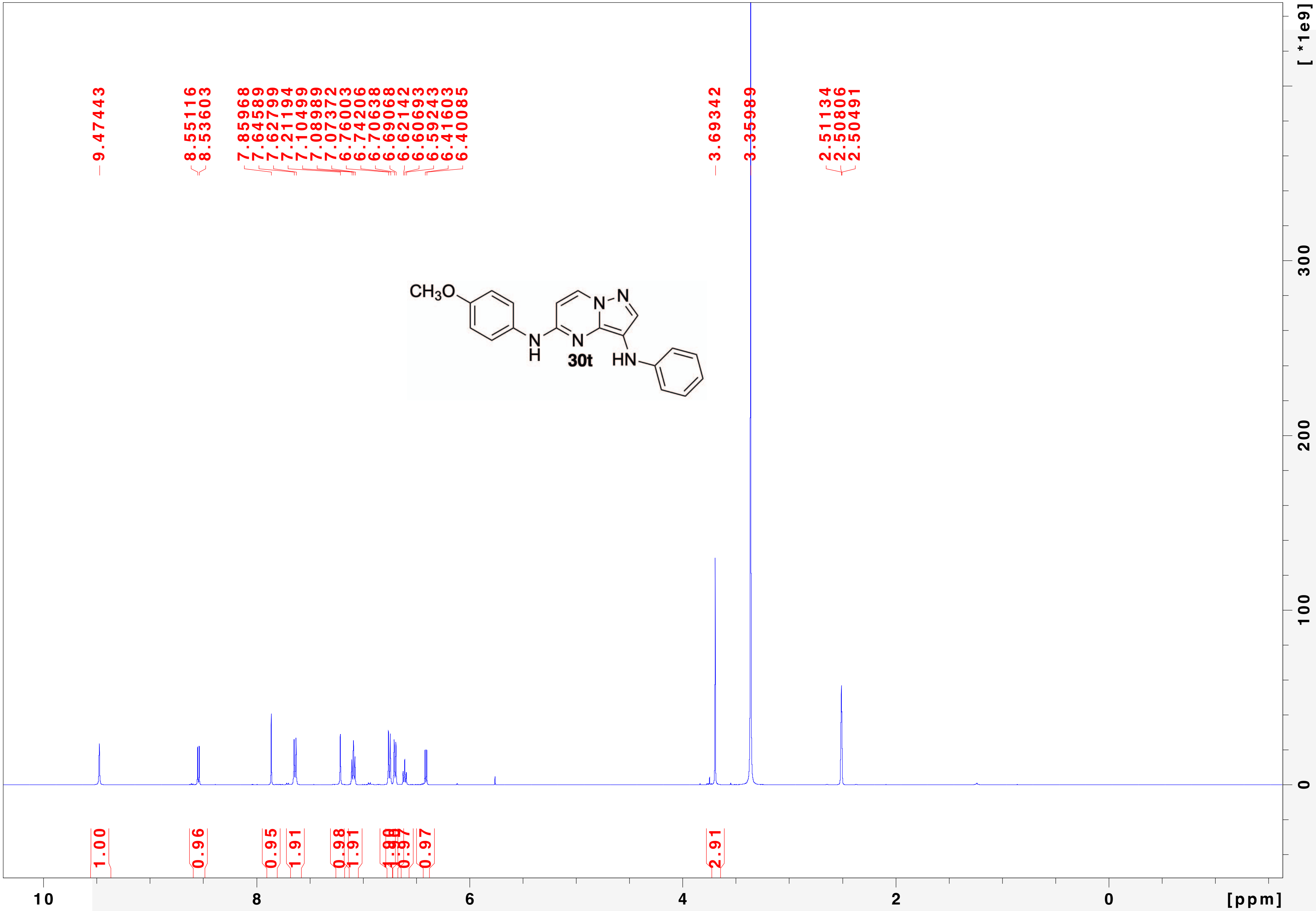

JI-III-54 10 1 /home/nmruser/networkdata/Bru500/terungwa@chem.byu.edu

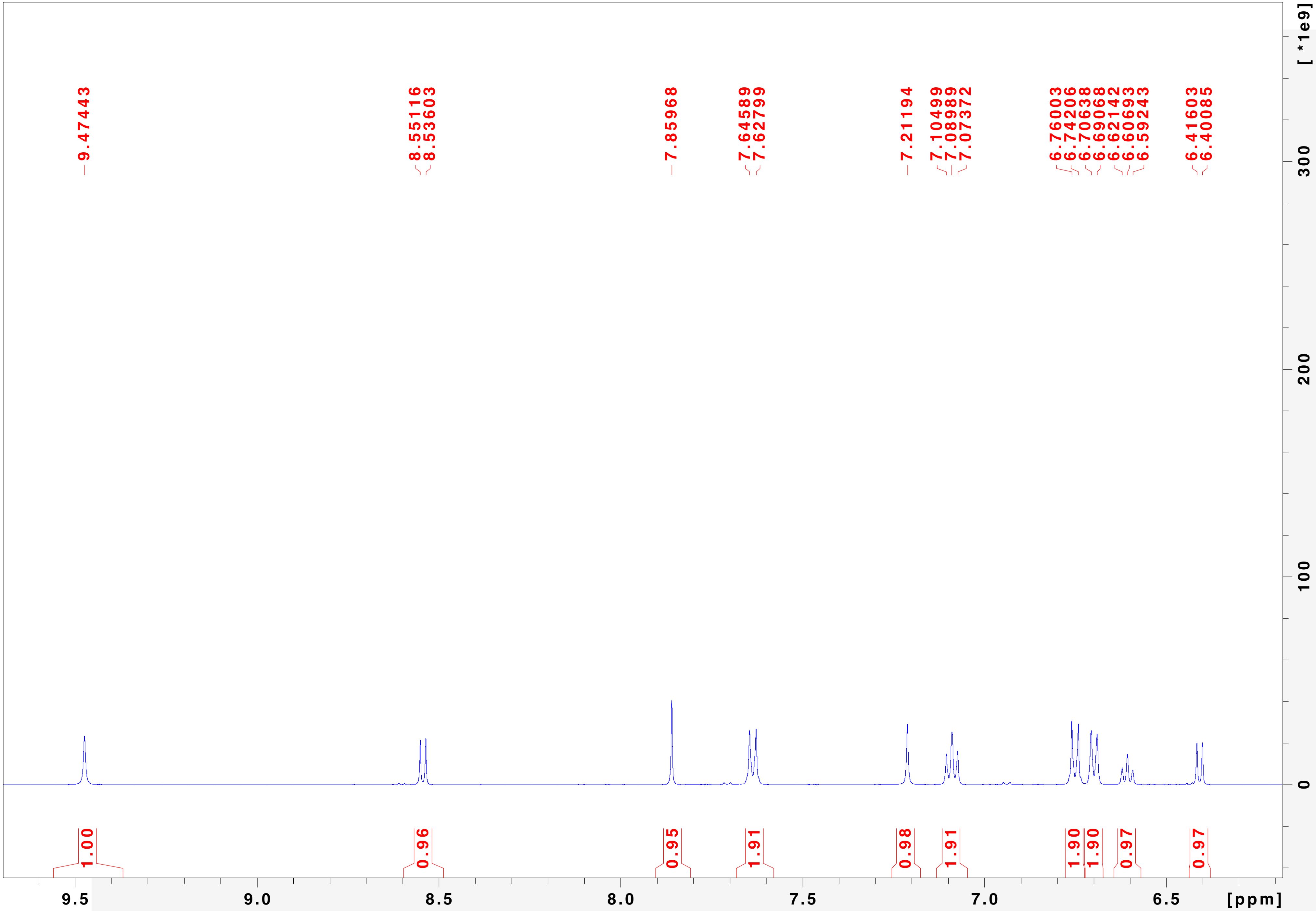

JI-III-54 20 1 /home/nmruser/networkdata/Bru500/terungwa@chem.byu.edu

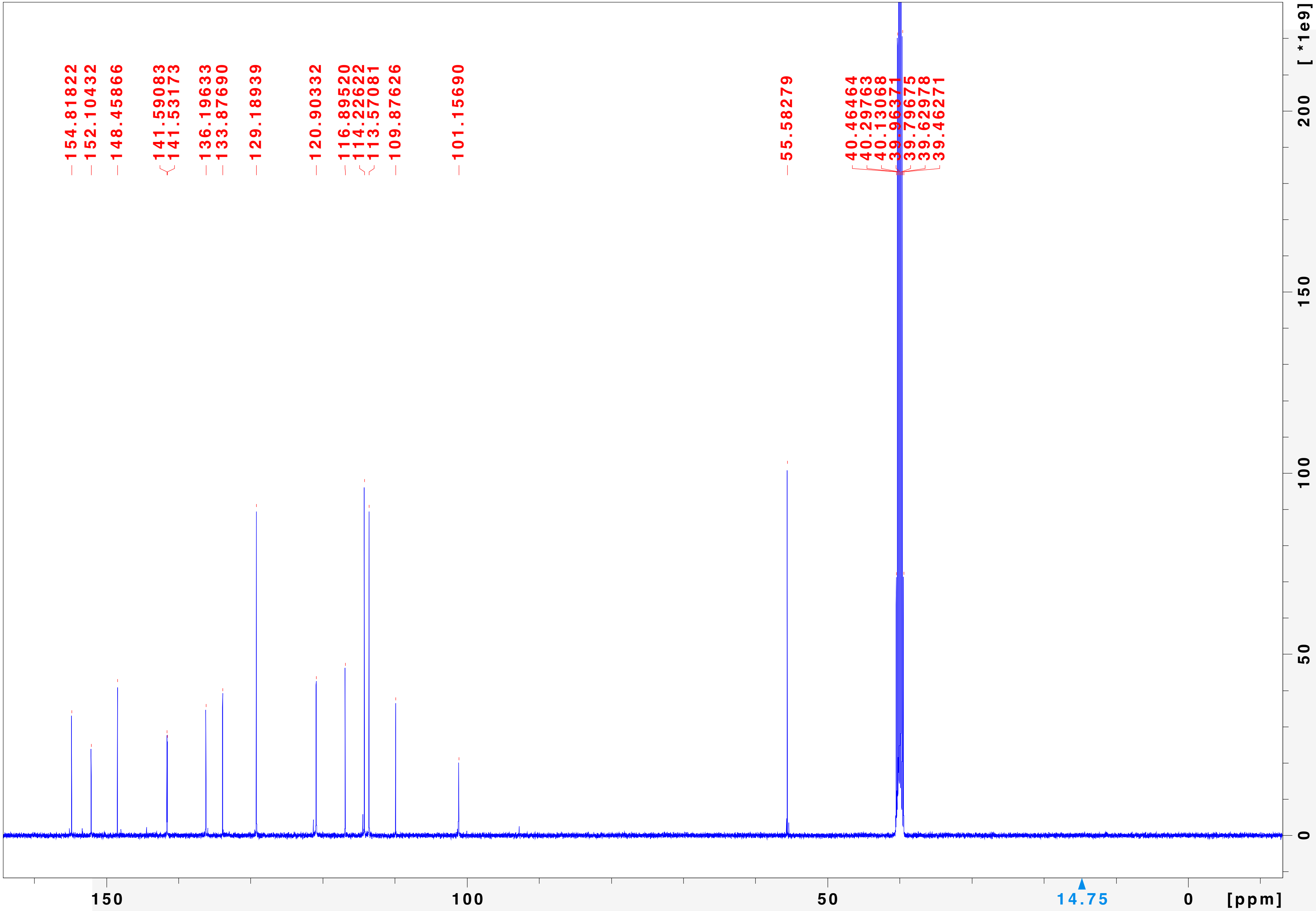

JI-III-55 10 1 /home/nmruser/networkdata/Bru500/terungwa@chem.byu.edu

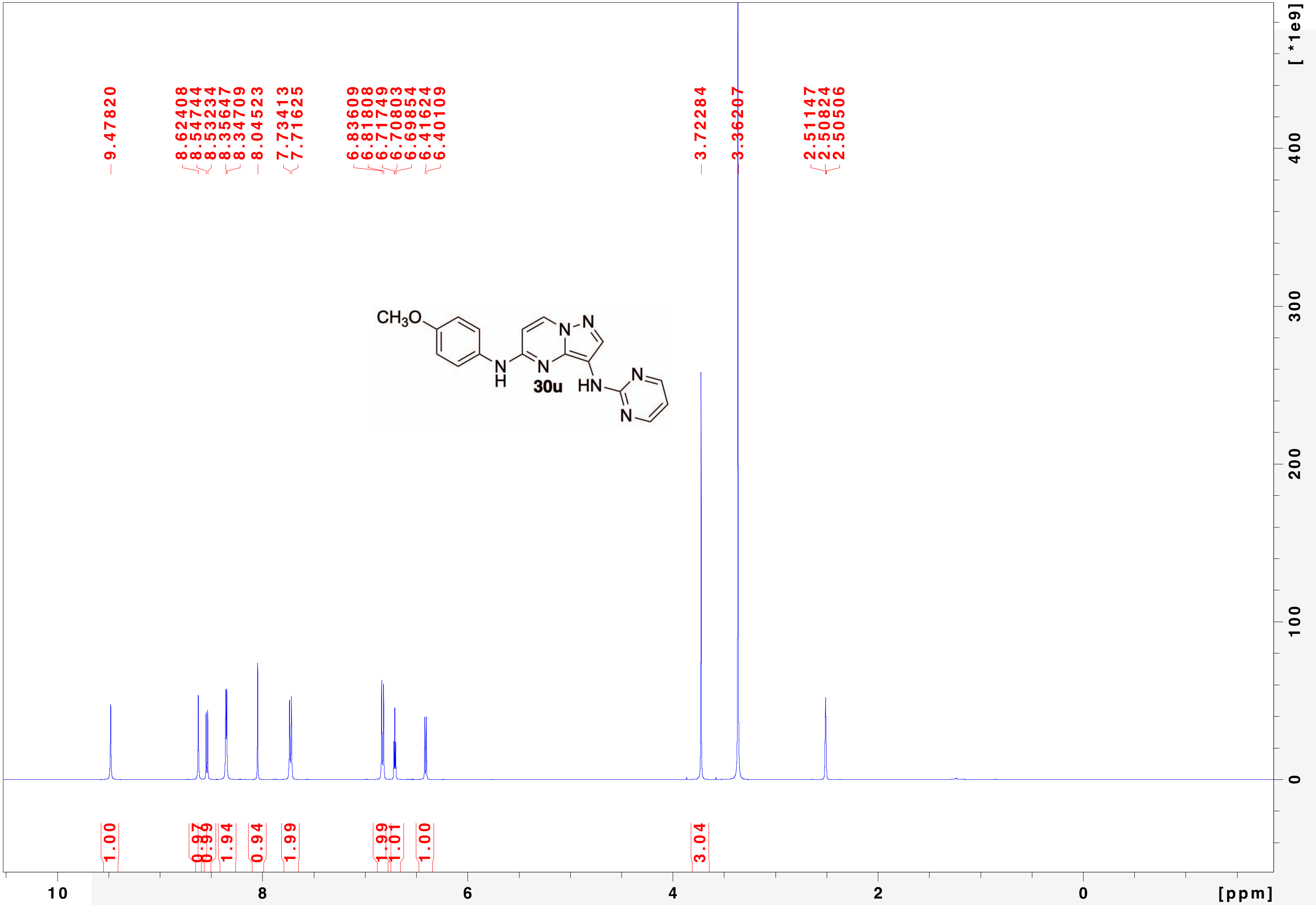

JI-III-55 10 1 /home/nmruser/networkdata/Bru500/terungwa@chem.byu.edu

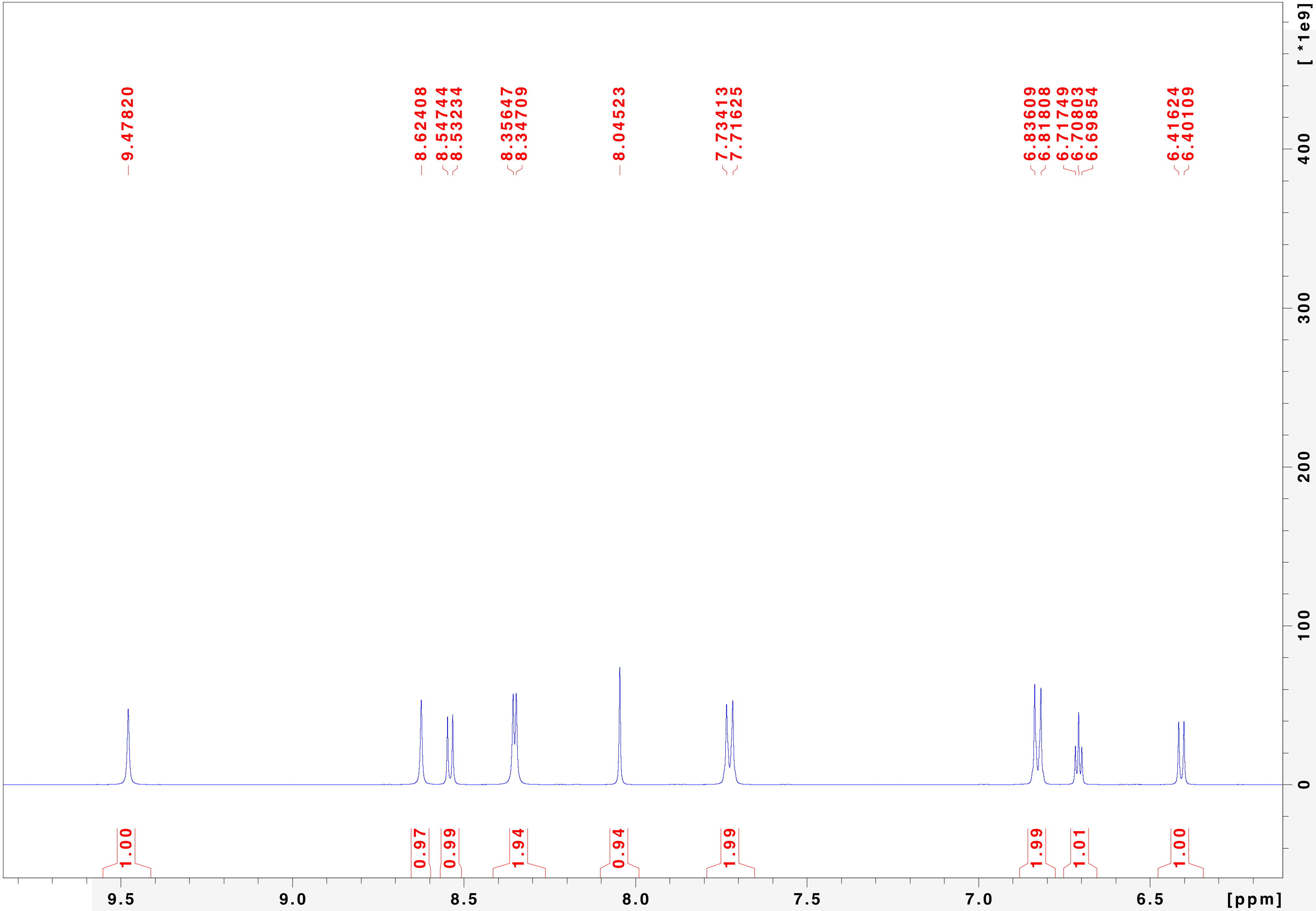

JI-III-55 20 1 /home/nmruser/networkdata/Bru500/terungwa@chem.byu.edu

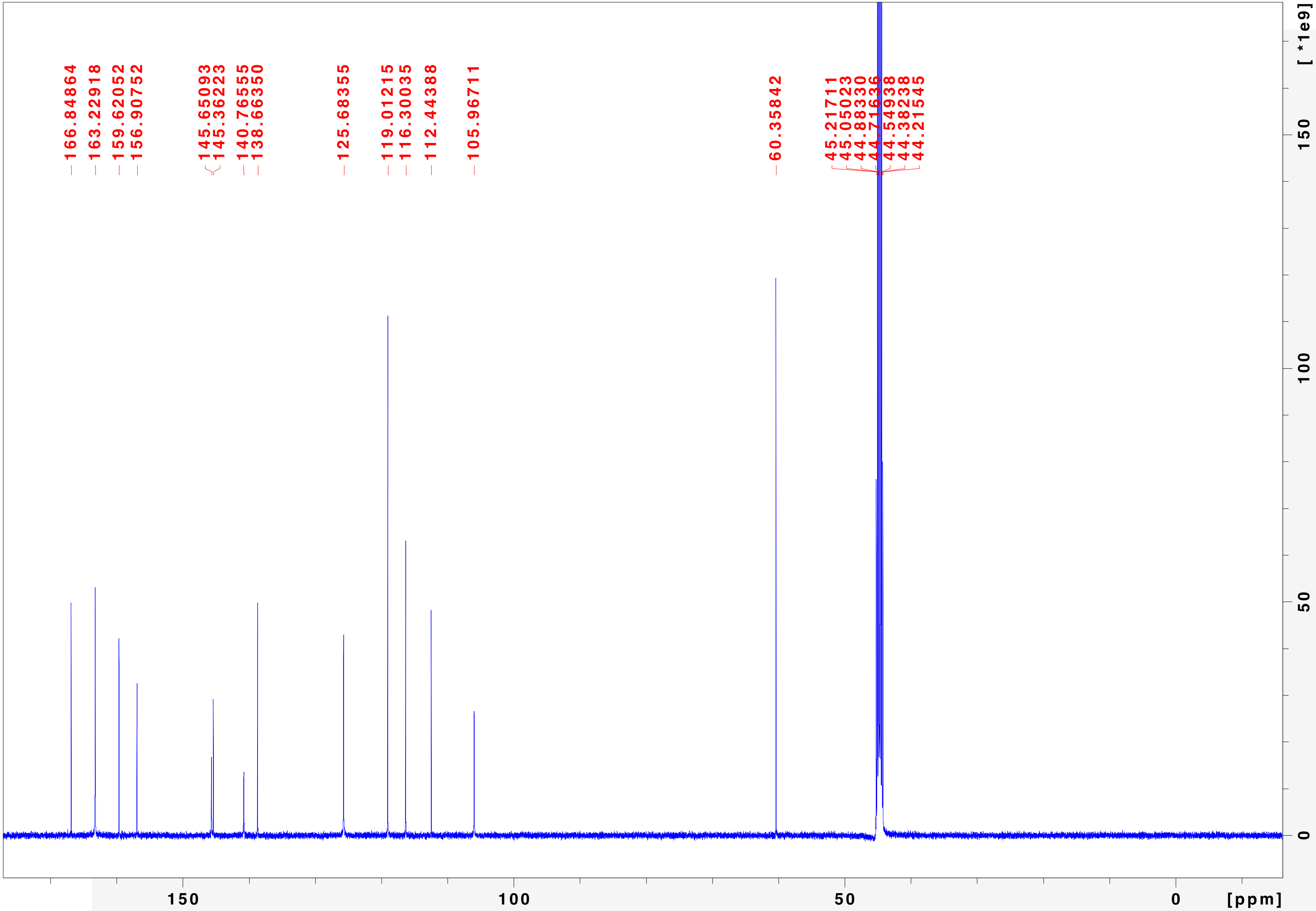

JI-III-43 30 1 /home/nmruser/networkdata/Bru500/terungwa@chem.byu.edu

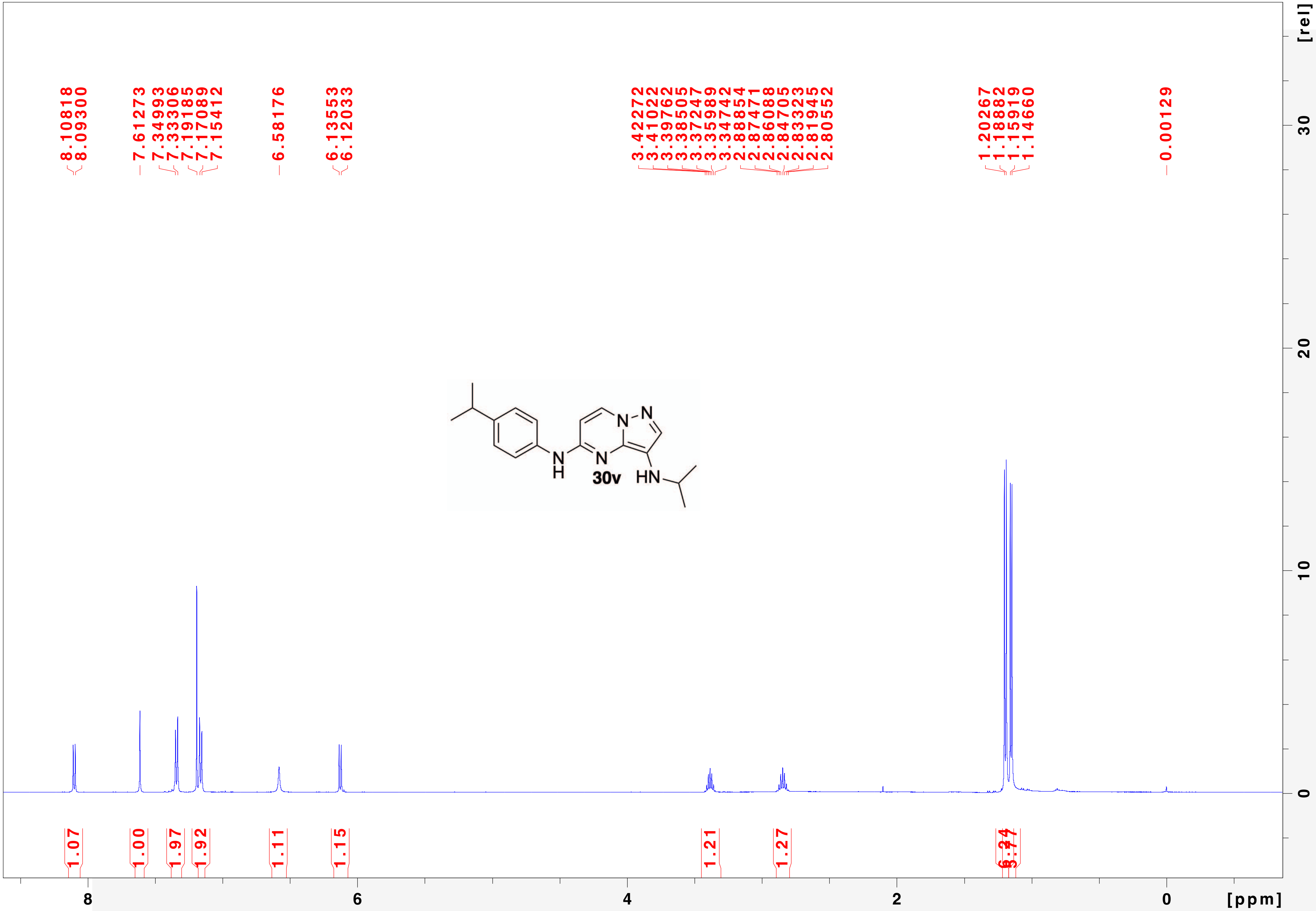

JI-III-43 30 1 /home/nmruser/networkdata/Bru500/terungwa@chem.byu.edu

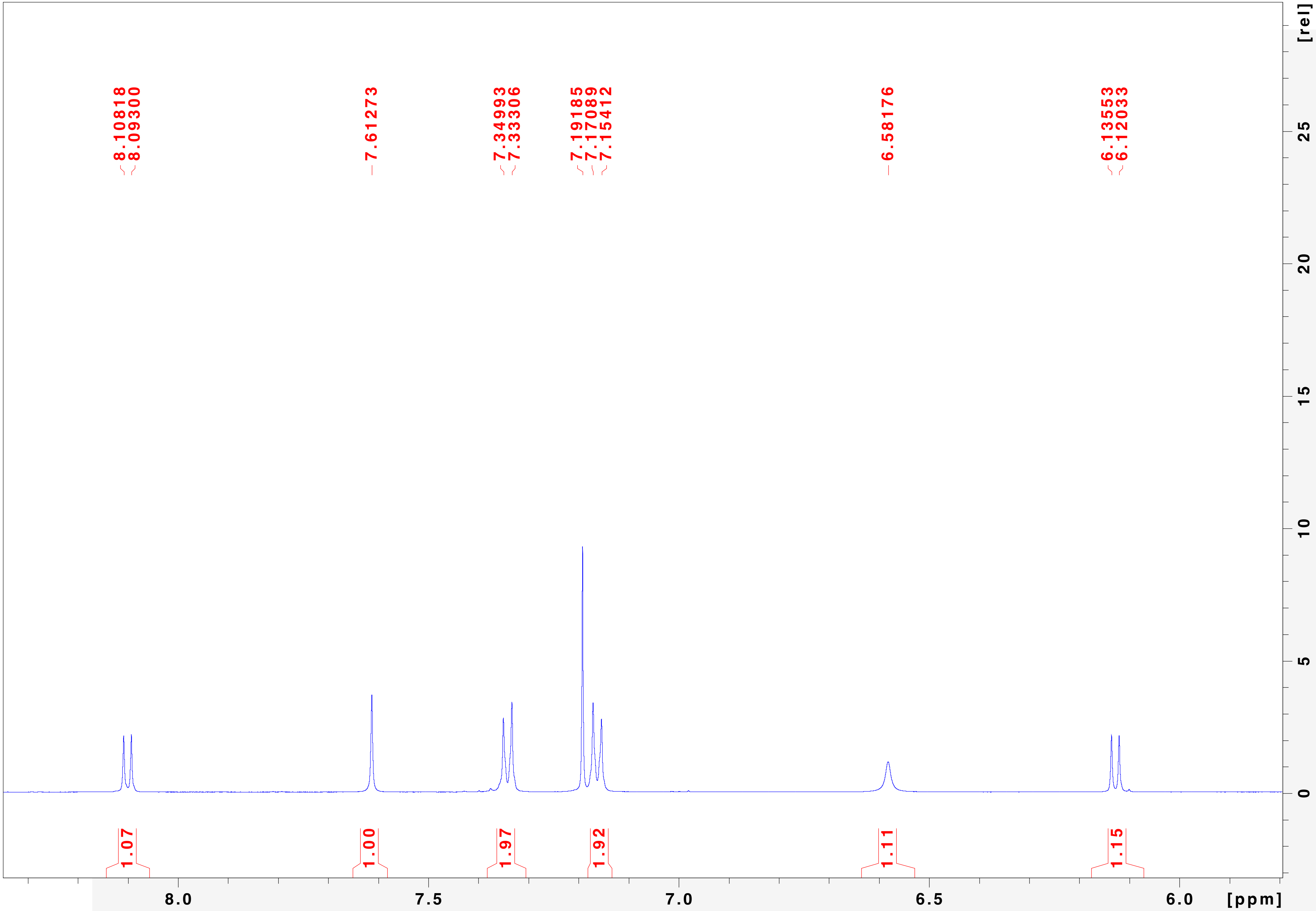

JI-III-43 30 1 /home/nmruser/networkdata/Bru500/terungwa@chem.byu.edu

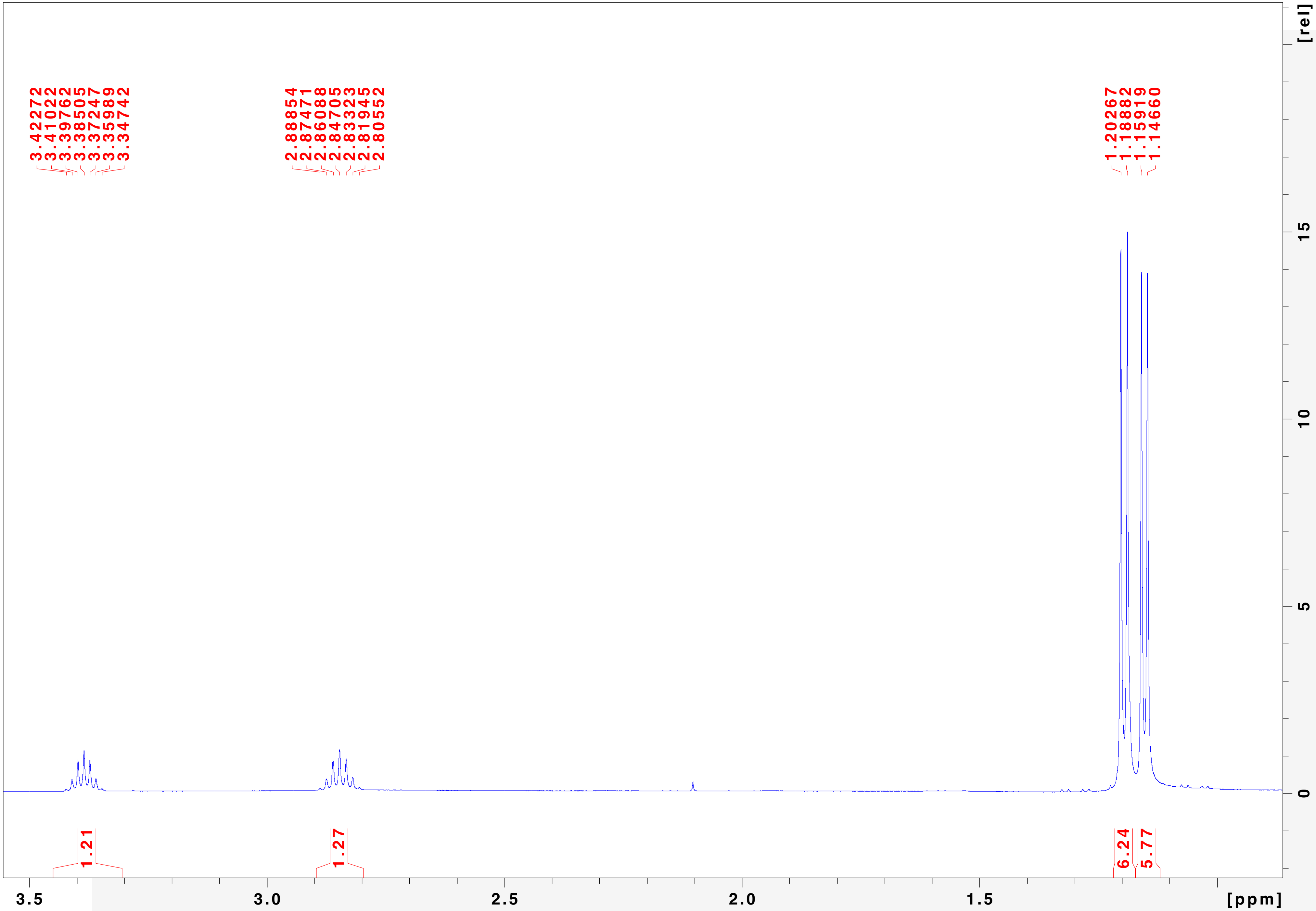

JI-III-43 20 1 /home/nmruser/networkdata/Bru500/terungwa@chem.byu.edu

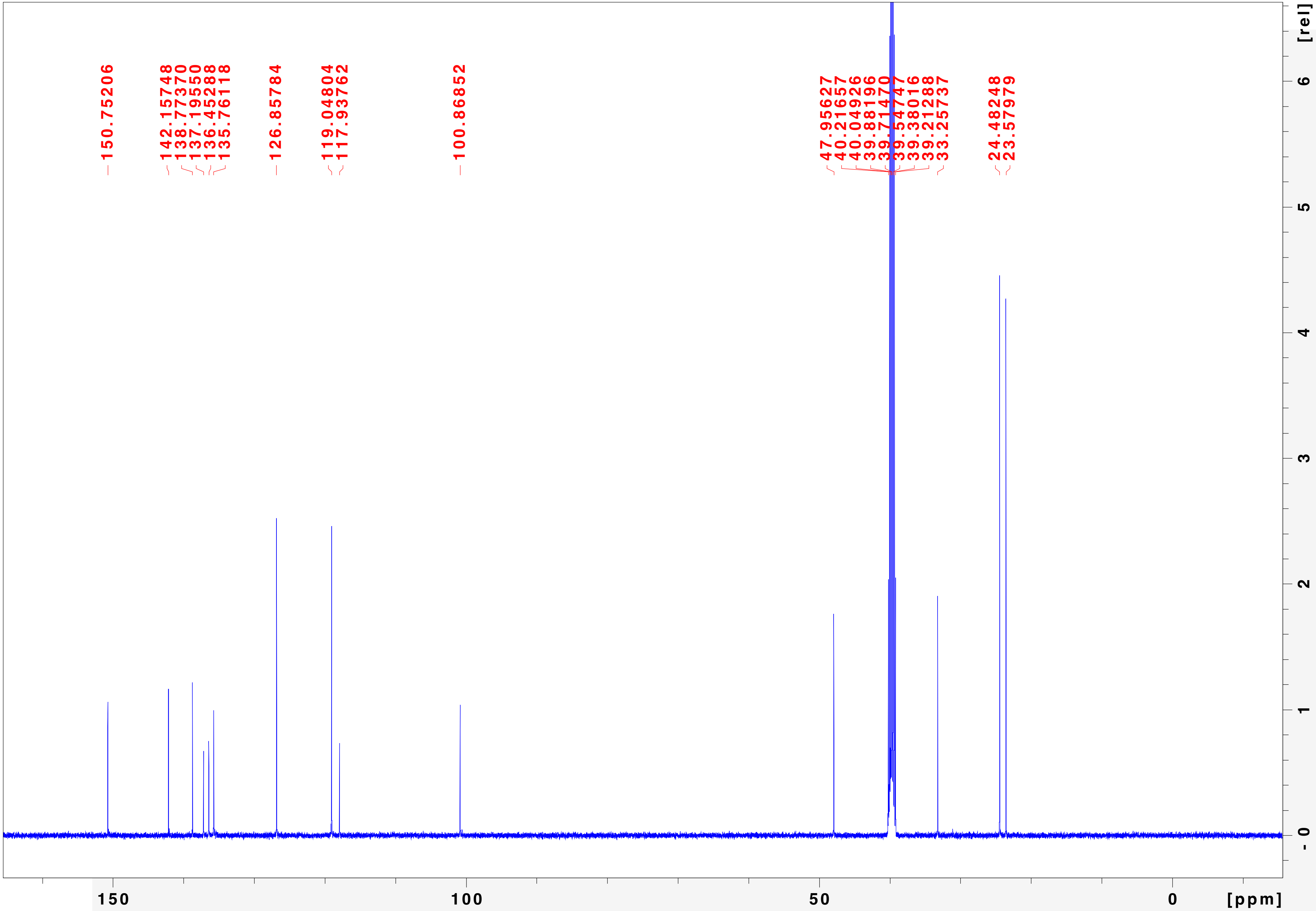

JI-III-42 10 1 /home/nmruser/networkdata/Bru500/terungwa@chem.byu.edu

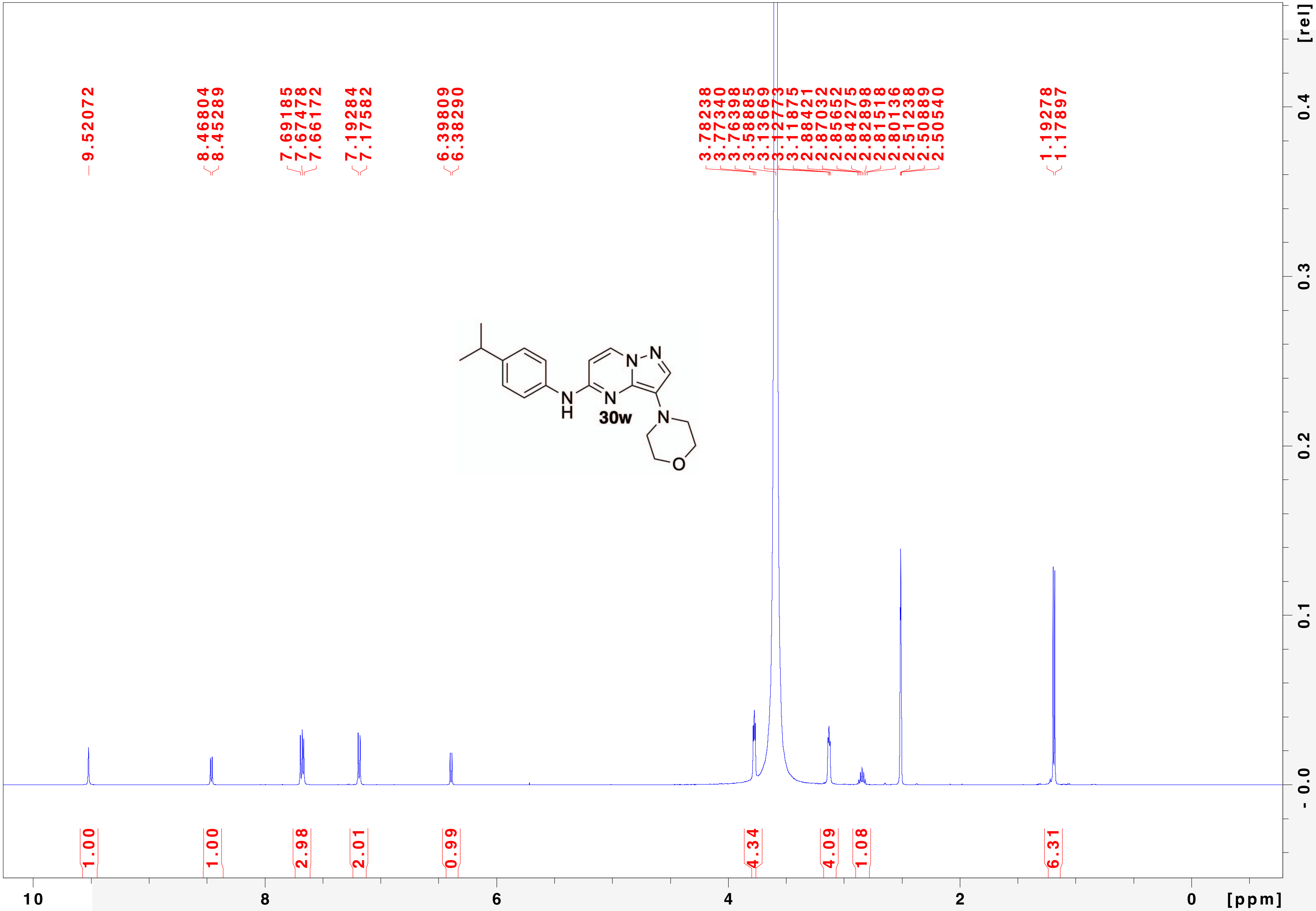

JI-III-42 10 1 /home/nmruser/networkdata/Bru500/terungwa@chem.byu.edu

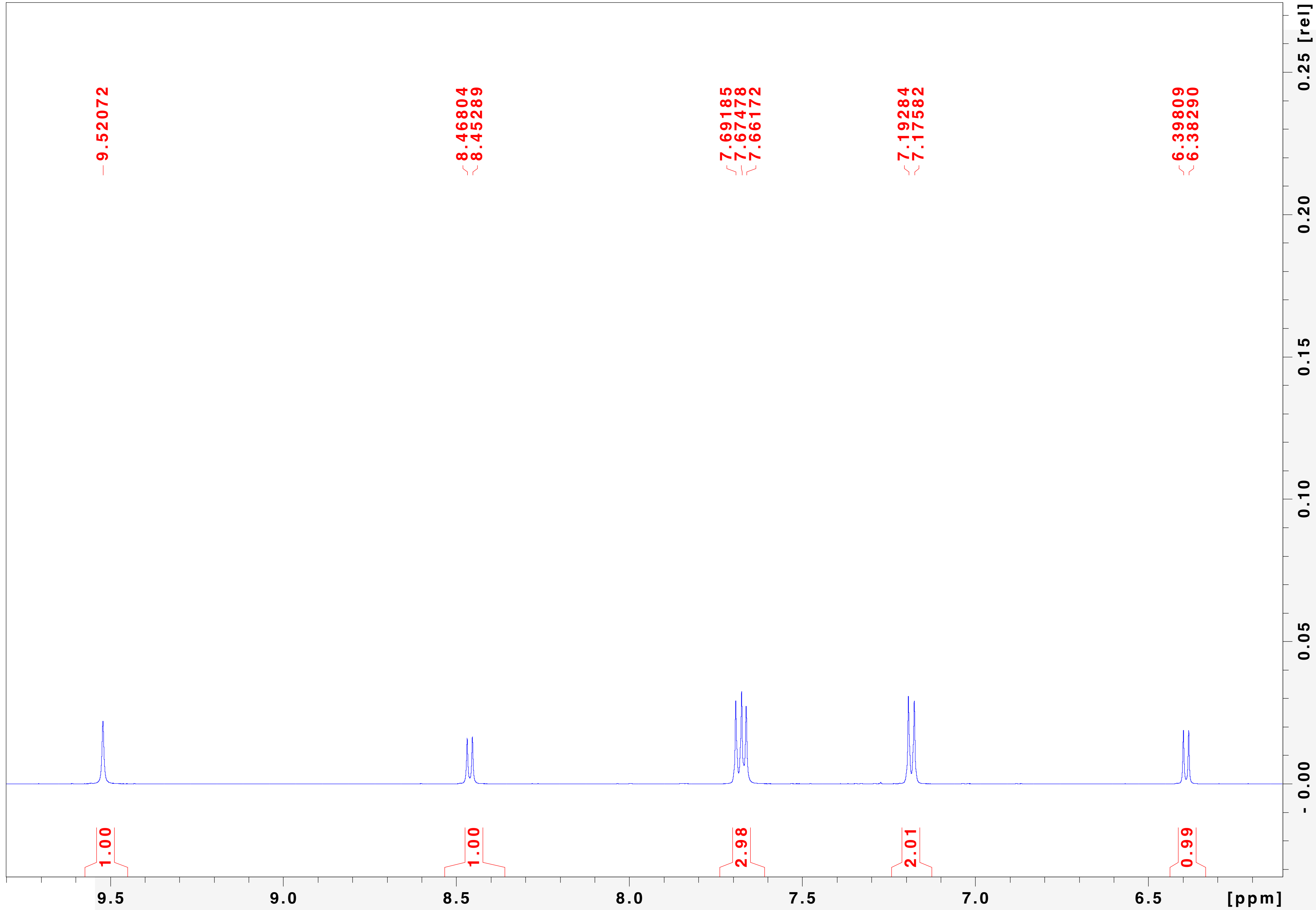

JI-III-42 10 1 /home/nmruser/networkdata/Bru500/terungwa@chem.byu.edu

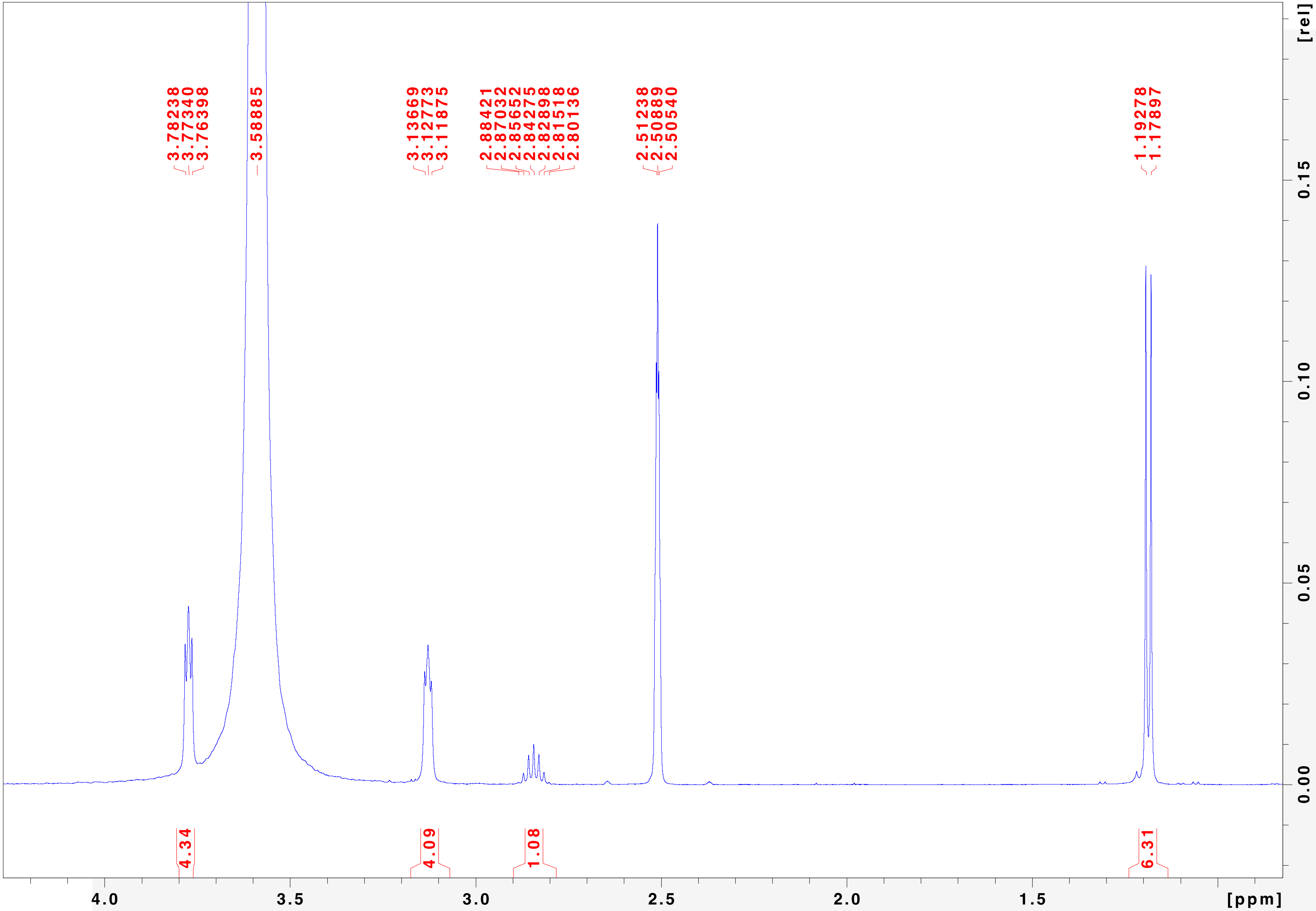

JI-III-42 20 1 /home/nmruser/networkdata/Bru500/terungwa@chem.byu.edu

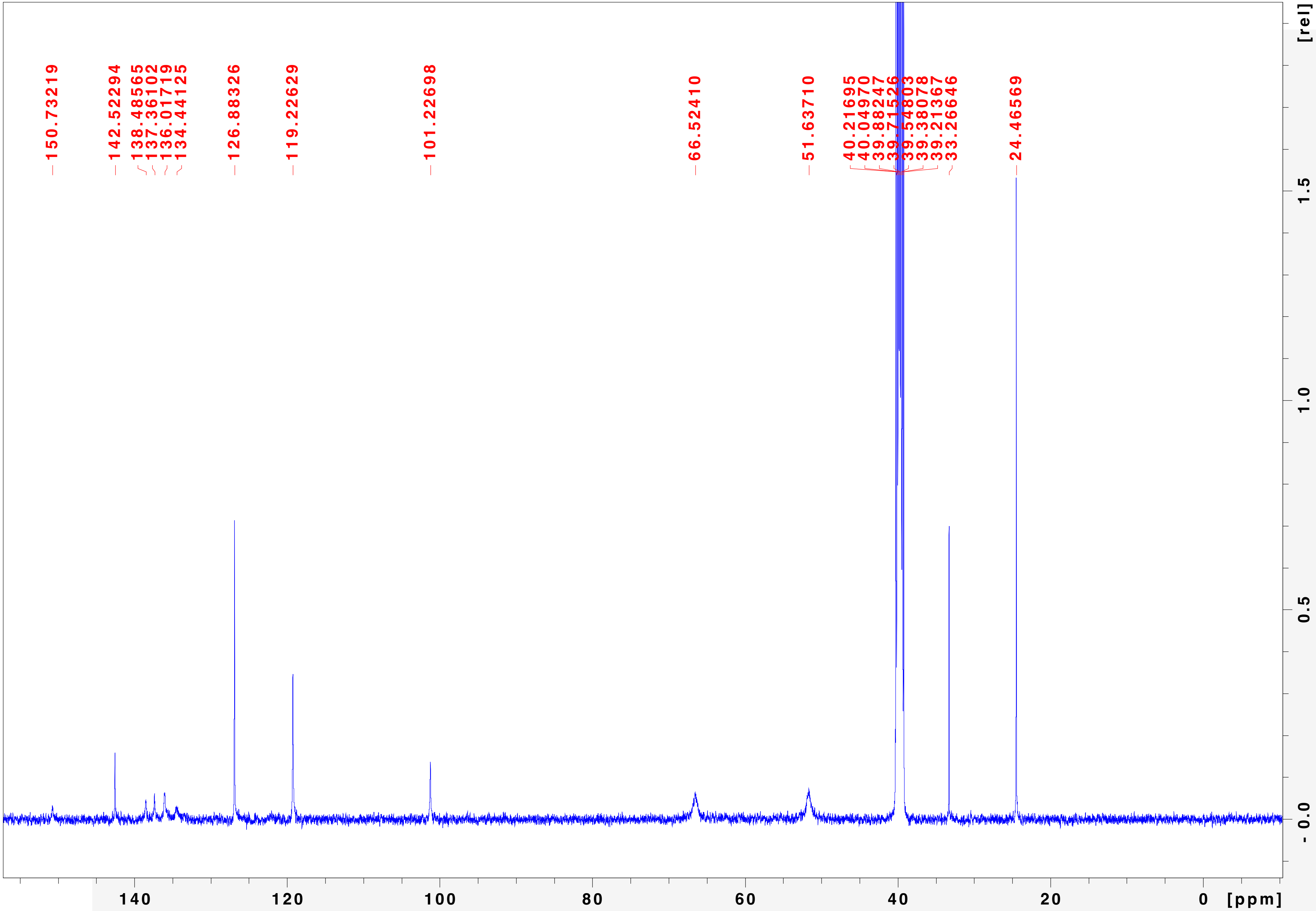

JI-III-42 20 1 /home/nmruser/networkdata/Bru500/terungwa@chem.byu.edu

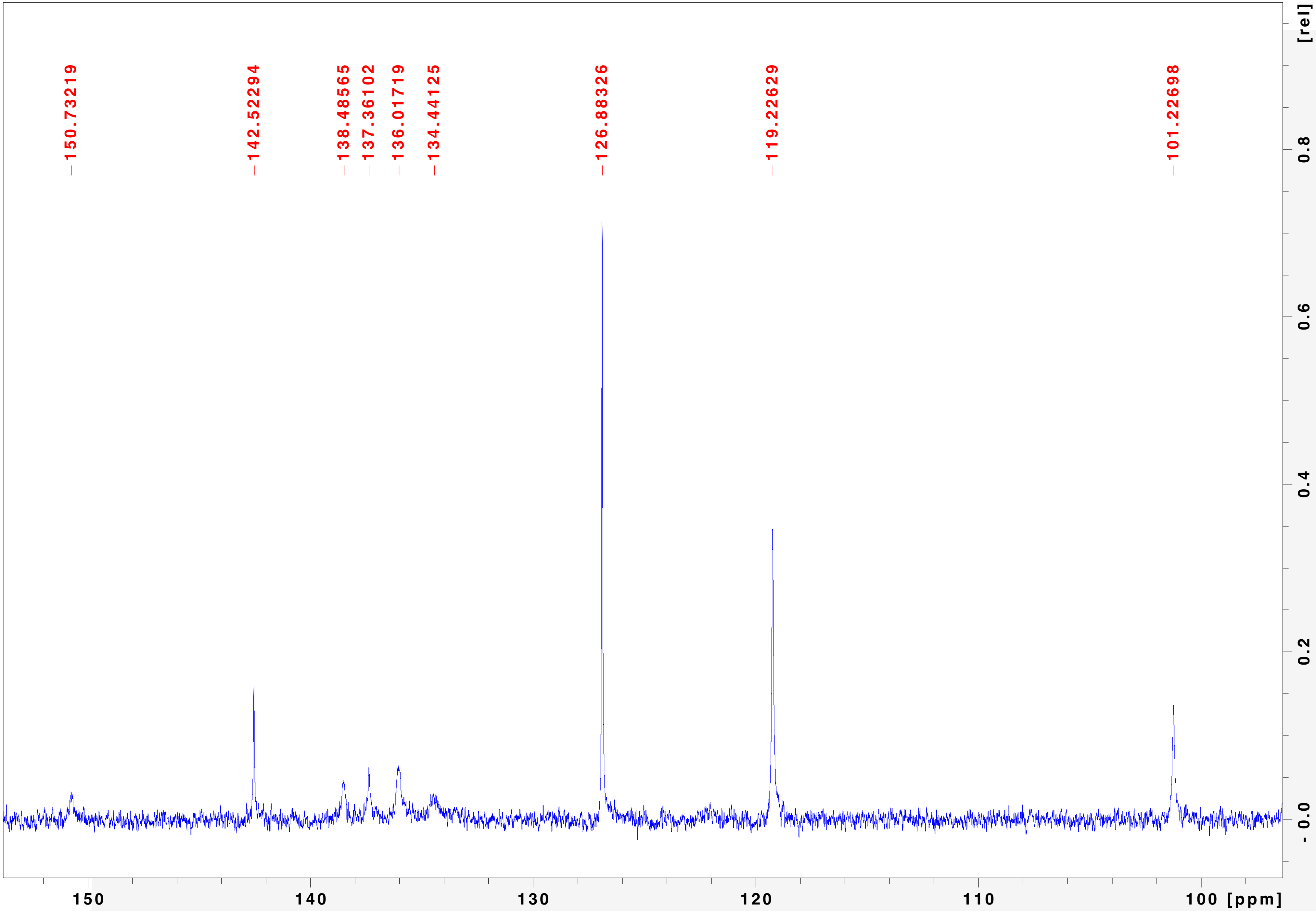

JI-III-42 20 1 /home/nmruser/networkdata/Bru500/terungwa@chem.byu.edu

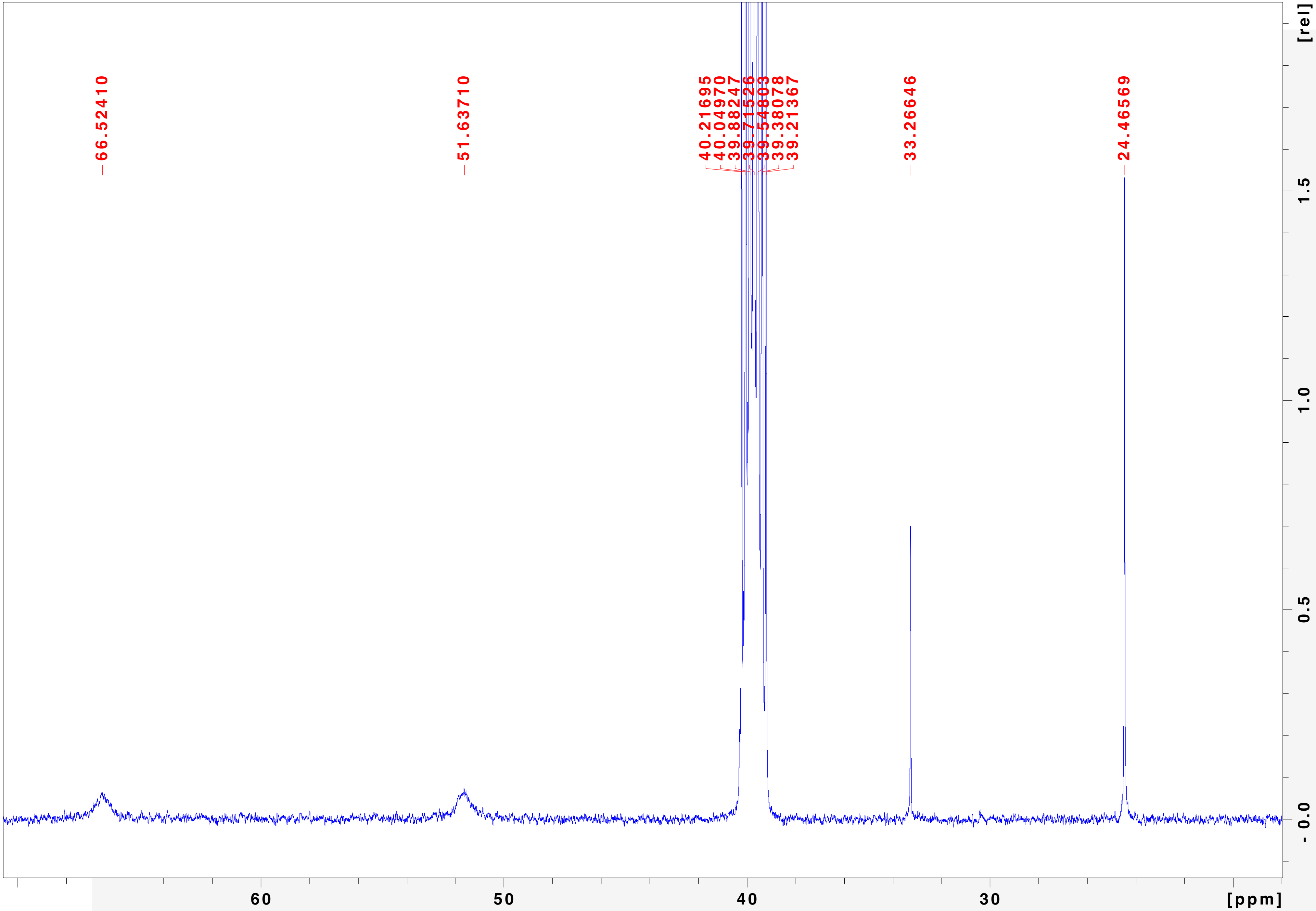

JI-III-46 10 1 /home/nmruser/networkdata/Bru500/terungwa@chem.byu.edu

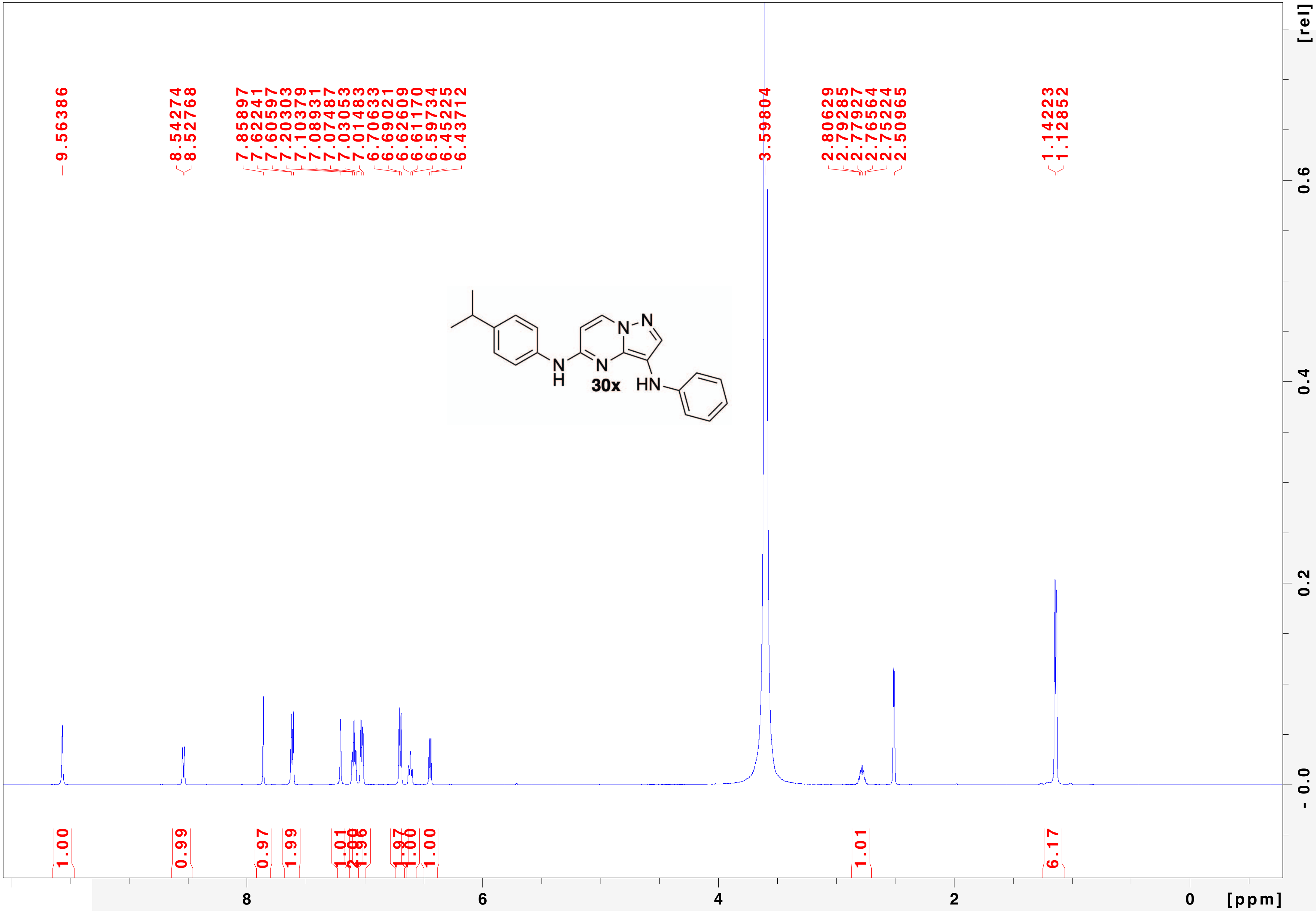

JI-III-46 10 1 /home/nmruser/networkdata/Bru500/terungwa@chem.byu.edu

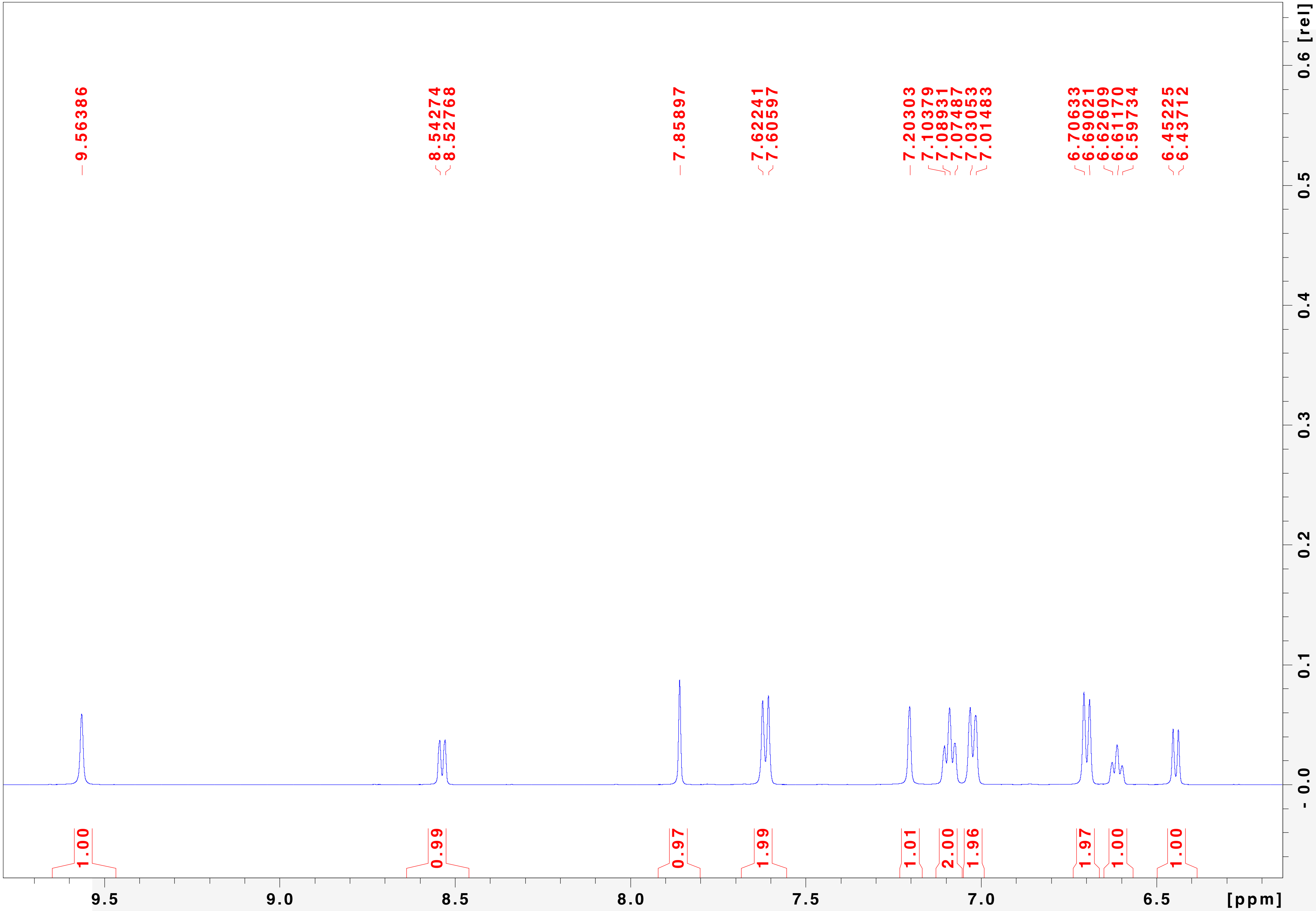

JI-III-46 10 1 /home/nmruser/networkdata/Bru500/terungwa@chem.byu.edu

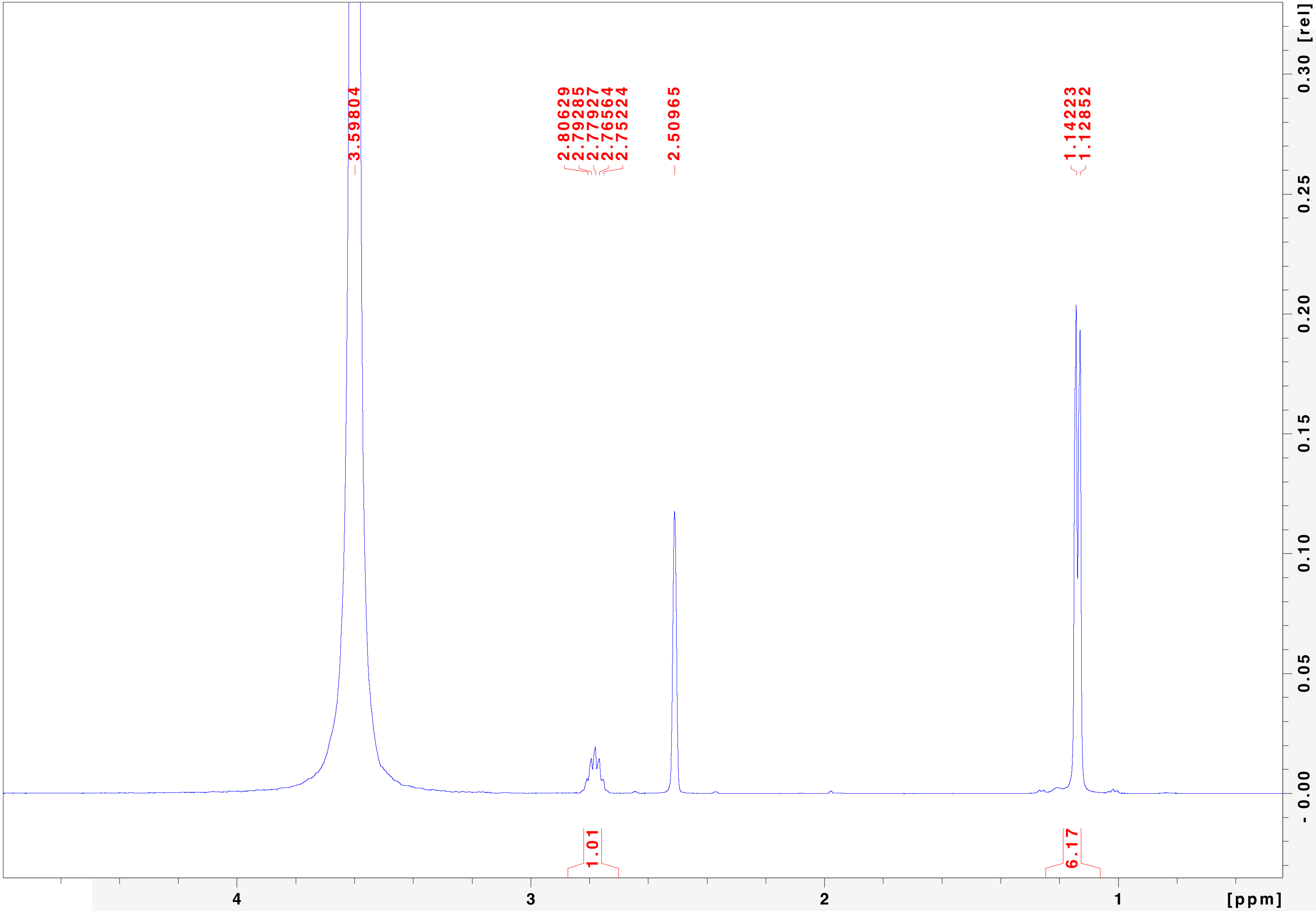

JI-III-46 20 1 /home/nmruser/networkdata/Bru500/terungwa@chem.byu.edu

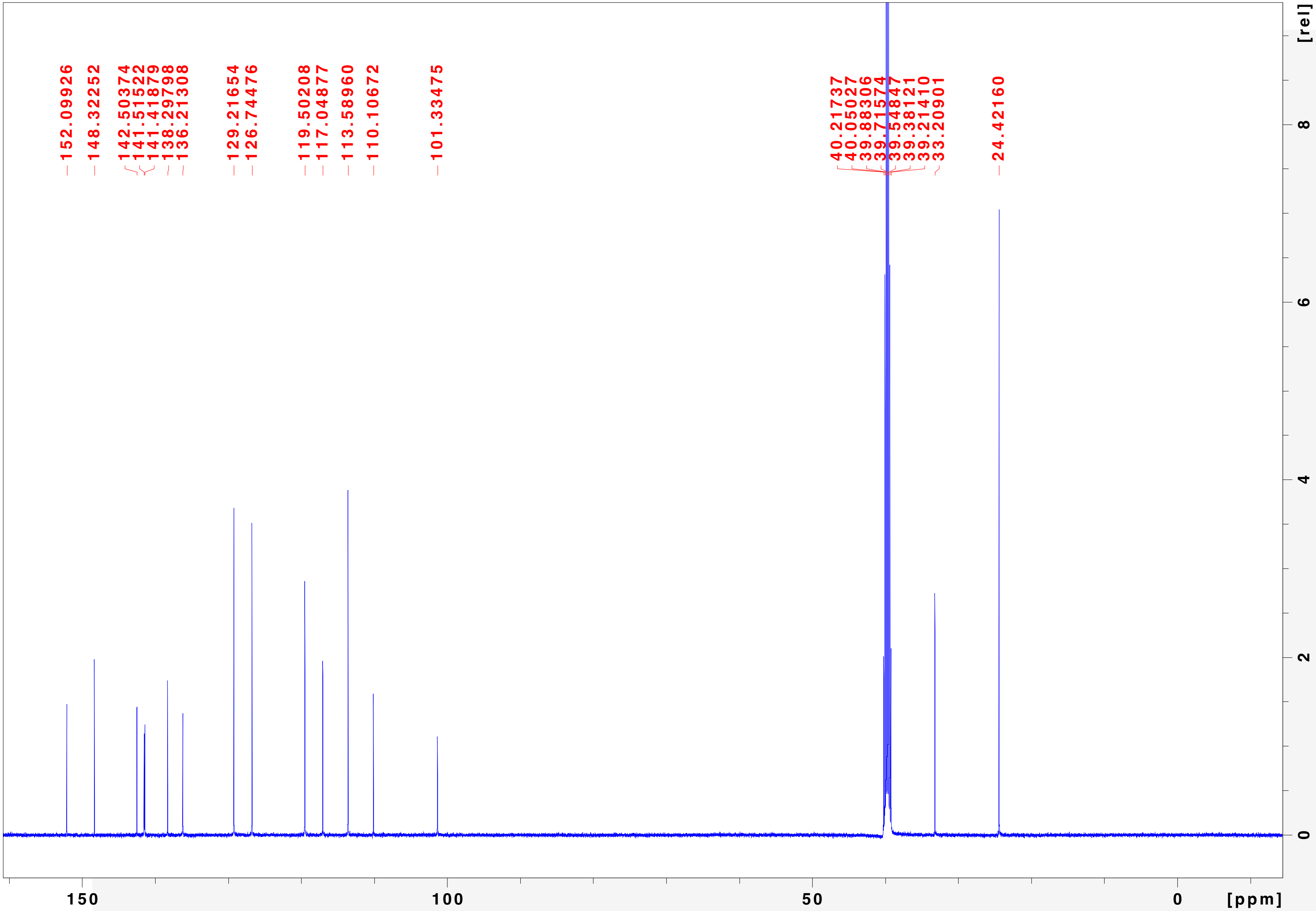

JI-III-51 20 1 /home/nmruser/networkdata/Bru500/terungwa@chem.byu.edu

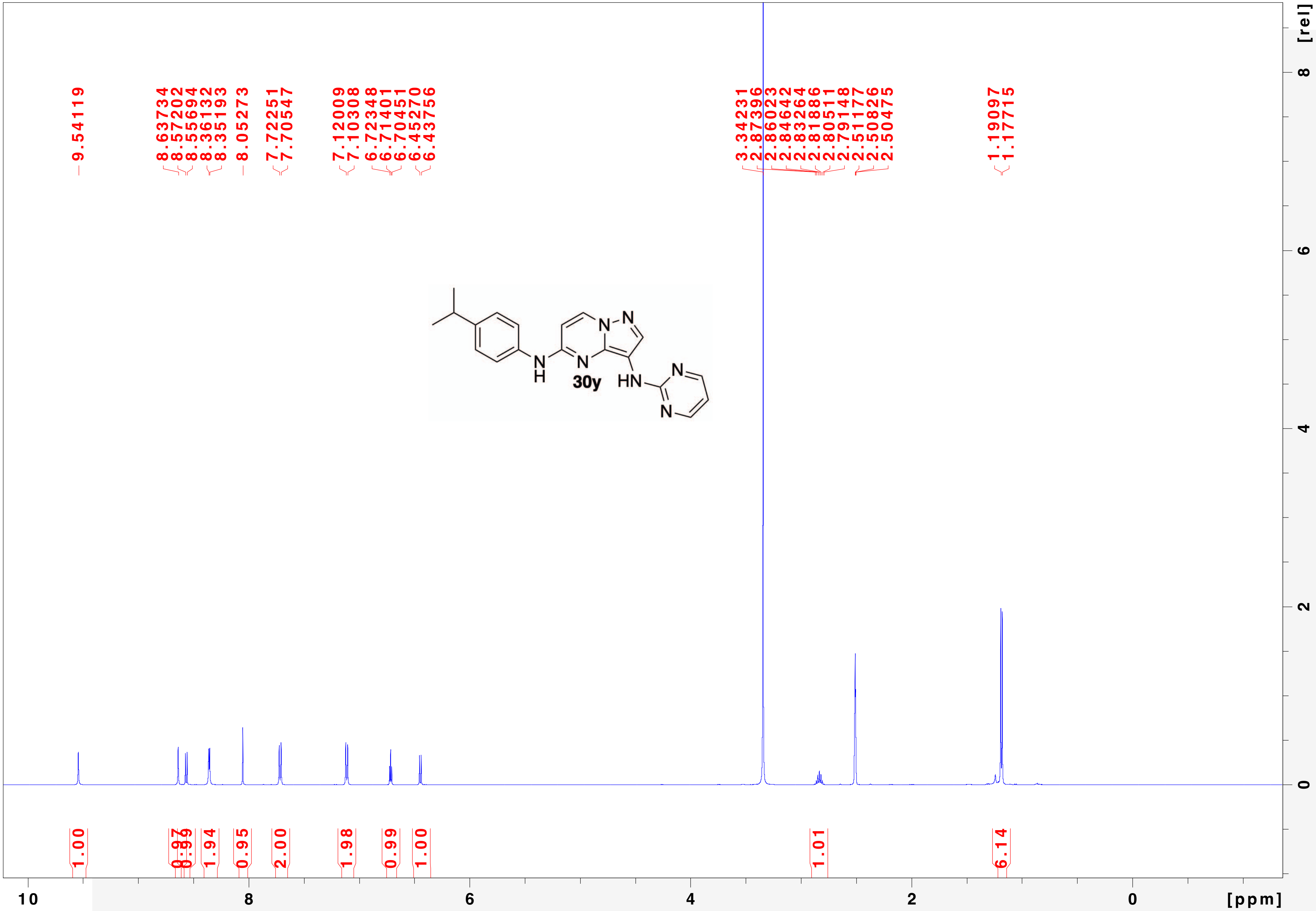

30y

JI-III-51 20 1 /home/nmruser/networkdata/Bru500/terungwa@chem.byu.edu

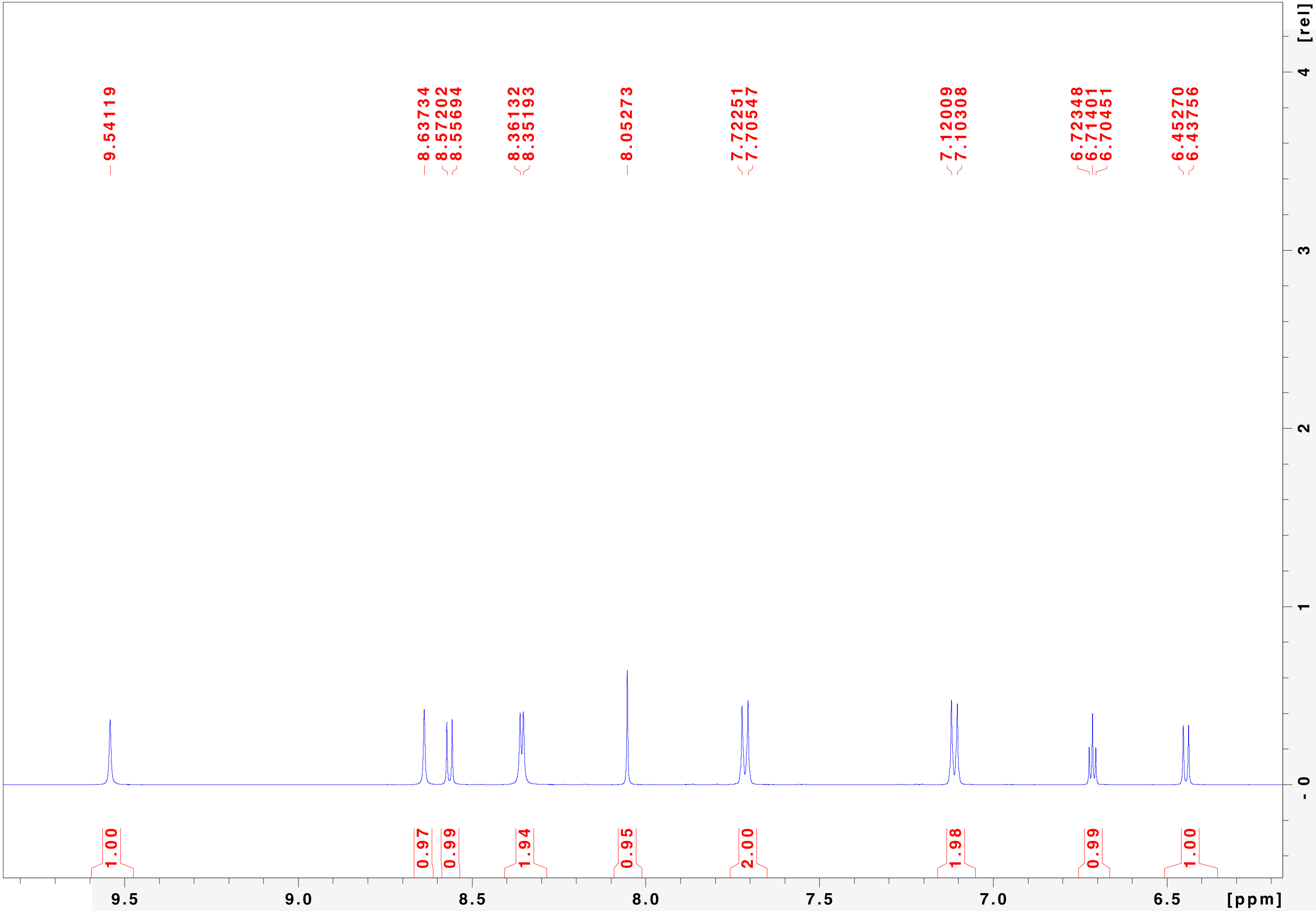

JI-III-51 20 1 /home/nmruser/networkdata/Bru500/terungwa@chem.byu.edu

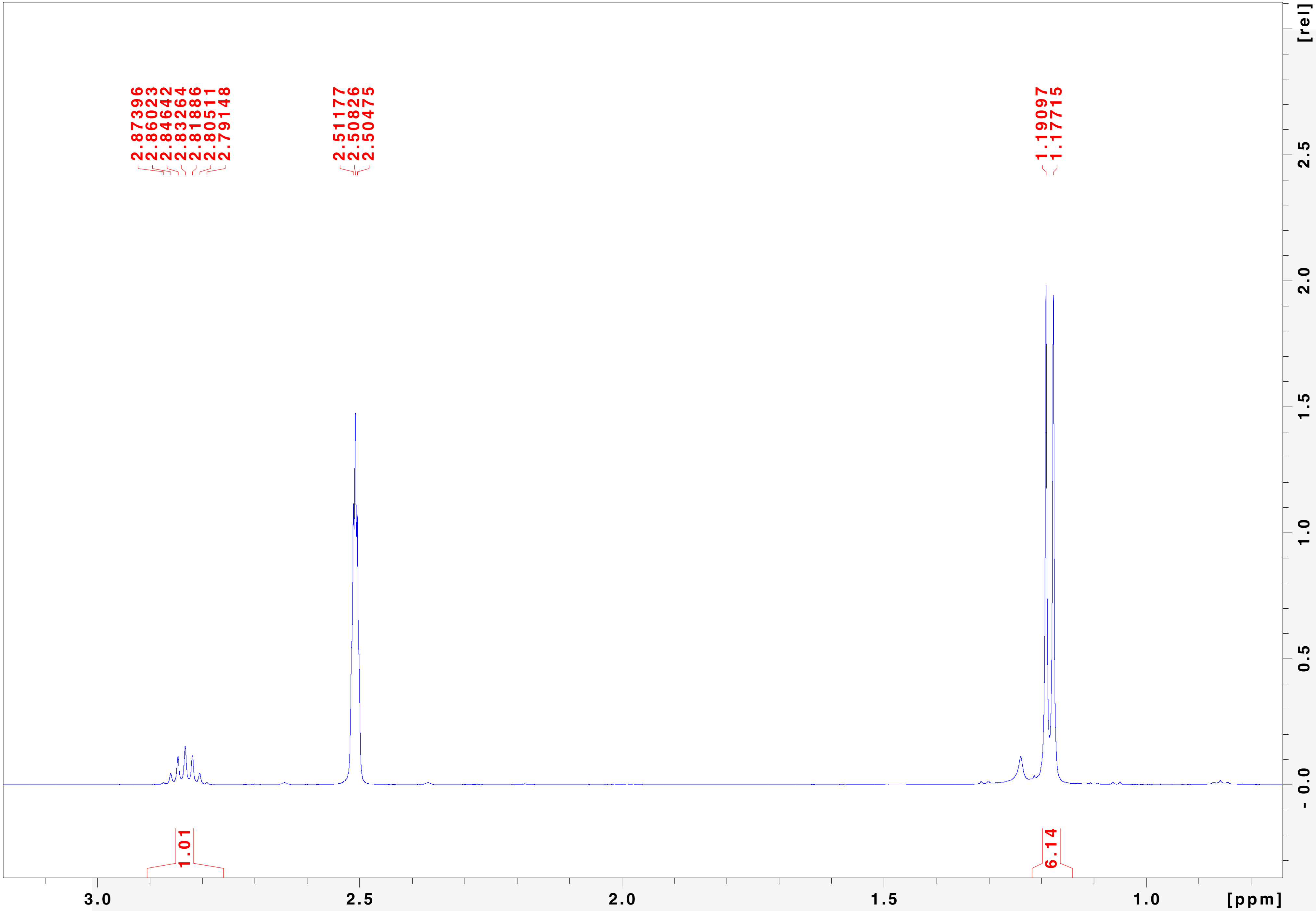

JI-III-51 30 1 /home/nmruser/networkdata/Bru500/terungwa@chem.byu.edu

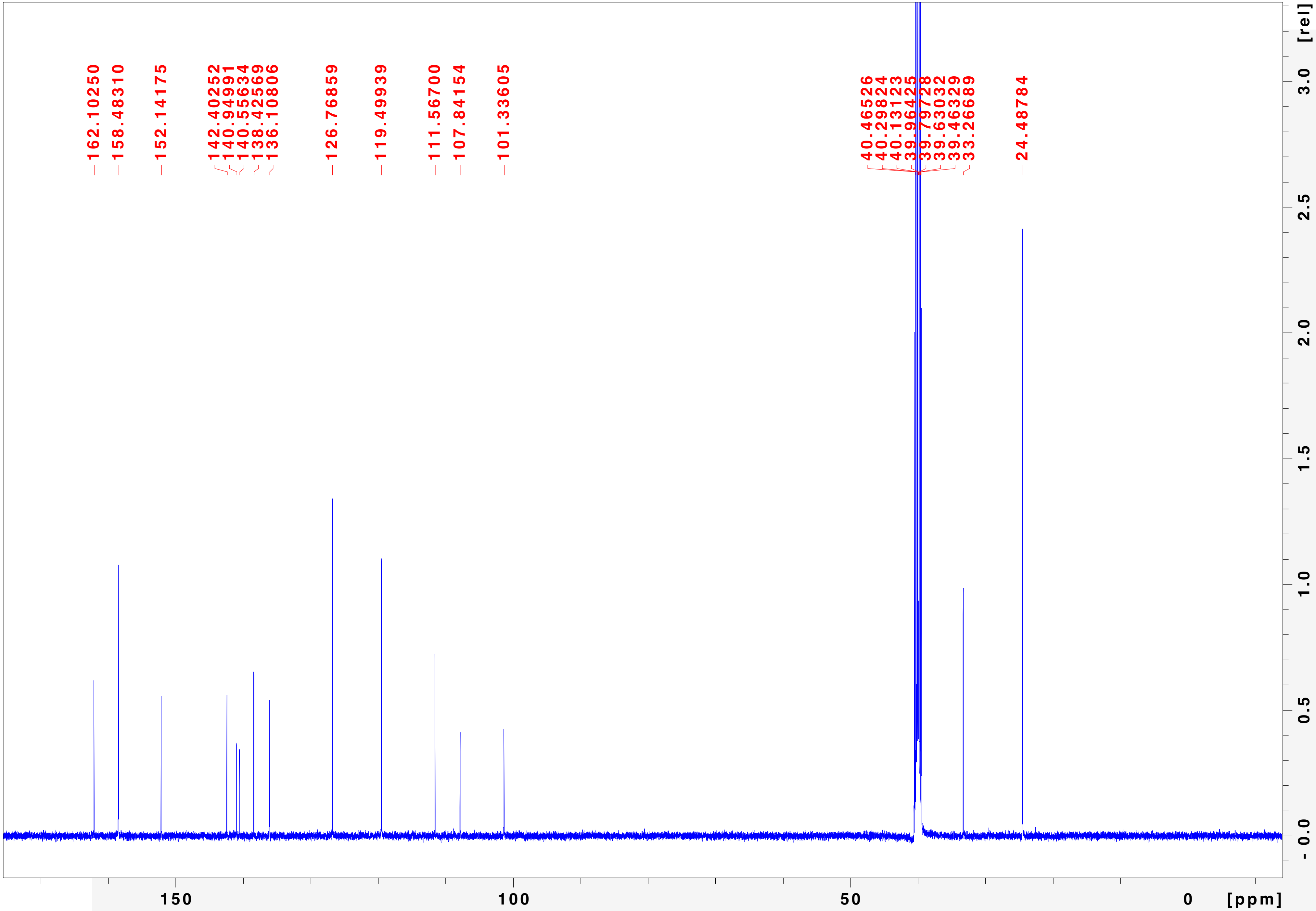

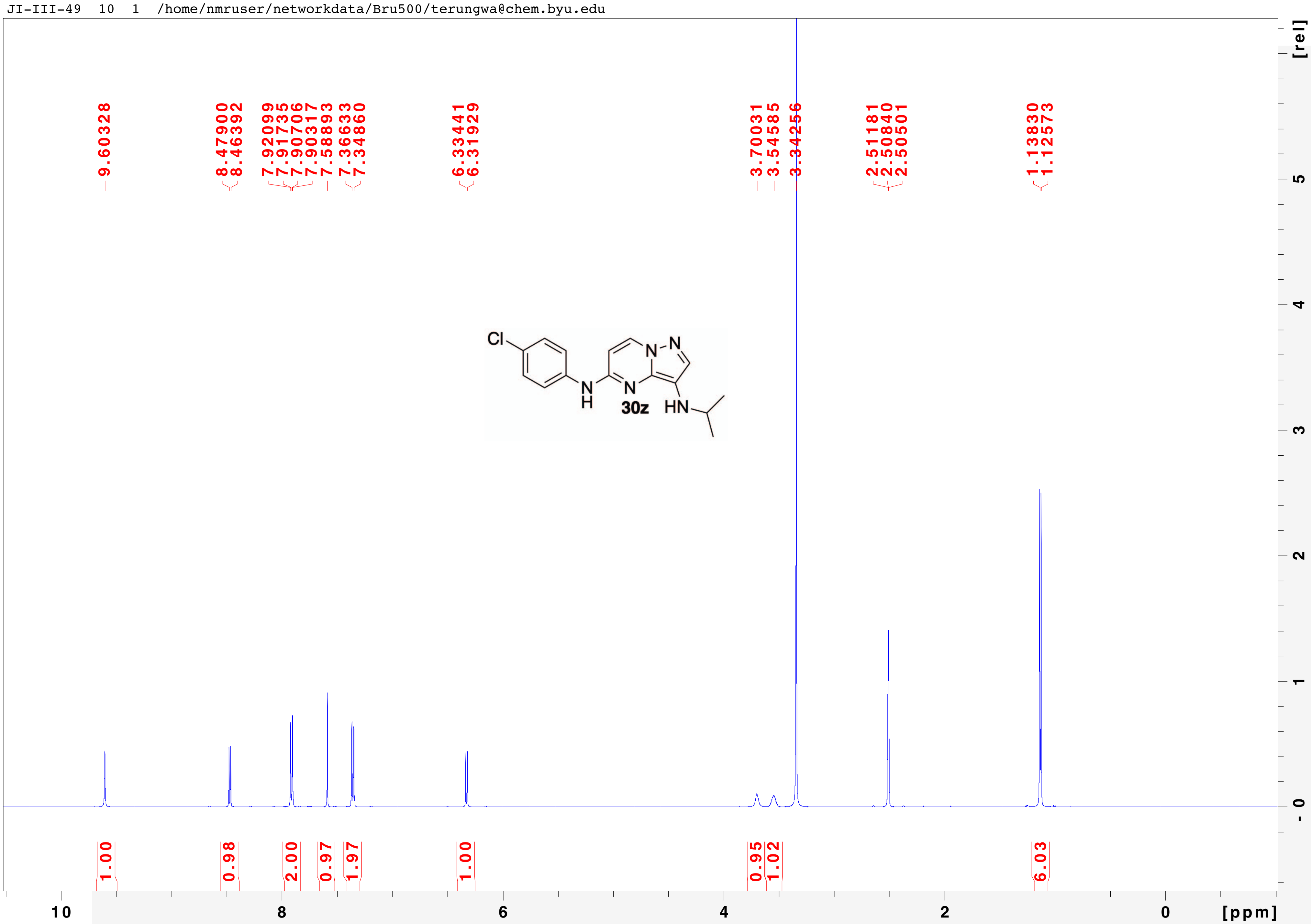
JI-III-49 10 1 /home/nmruser/networkdata/Bru500/terungwa@chem.byu.edu
9.60328
8.47900
8.46392
7.92099
7.91735
7.90706
7.90317
7.58893
7.36633
7.34860
6.33441
6.31929
3.70031
3.54585
3.34256
2.51181
2.50840
2.50501
1.13830
1.12573
Cl
N
H
N
N
N
30z
HN
1.00
0.98
2.00
0.97
1.97
1.00
0.95
1.02
6.03
10
8
6
4
2
0
[ppm]
[rel]
5
4
3
2
1
0

JI-III-49 10 1 /home/nmruser/networkdata/Bru500/terungwa@chem.byu.edu

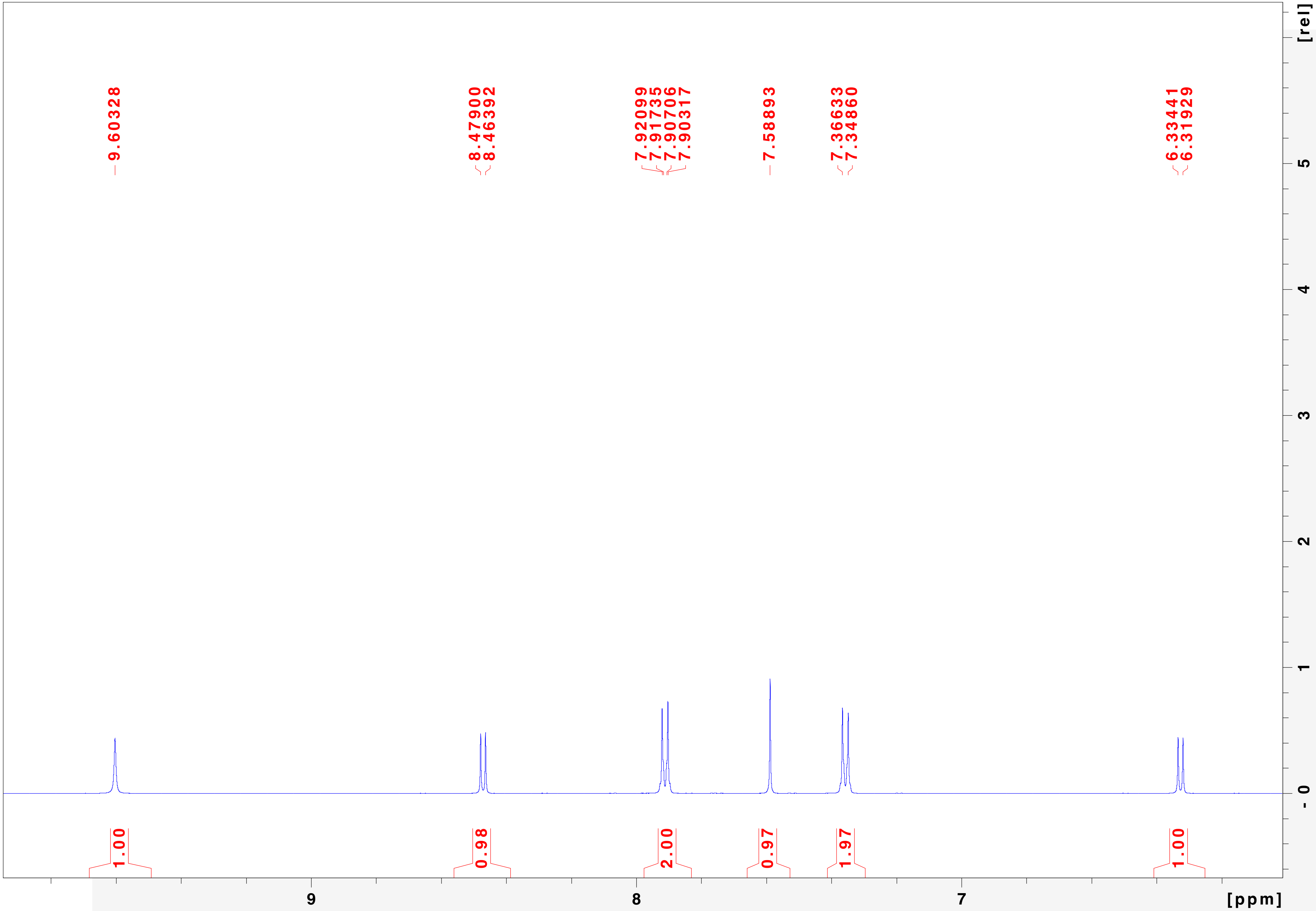

JI-III-49 10 1 /home/nmruser/networkdata/Bru500/terungwa@chem.byu.edu

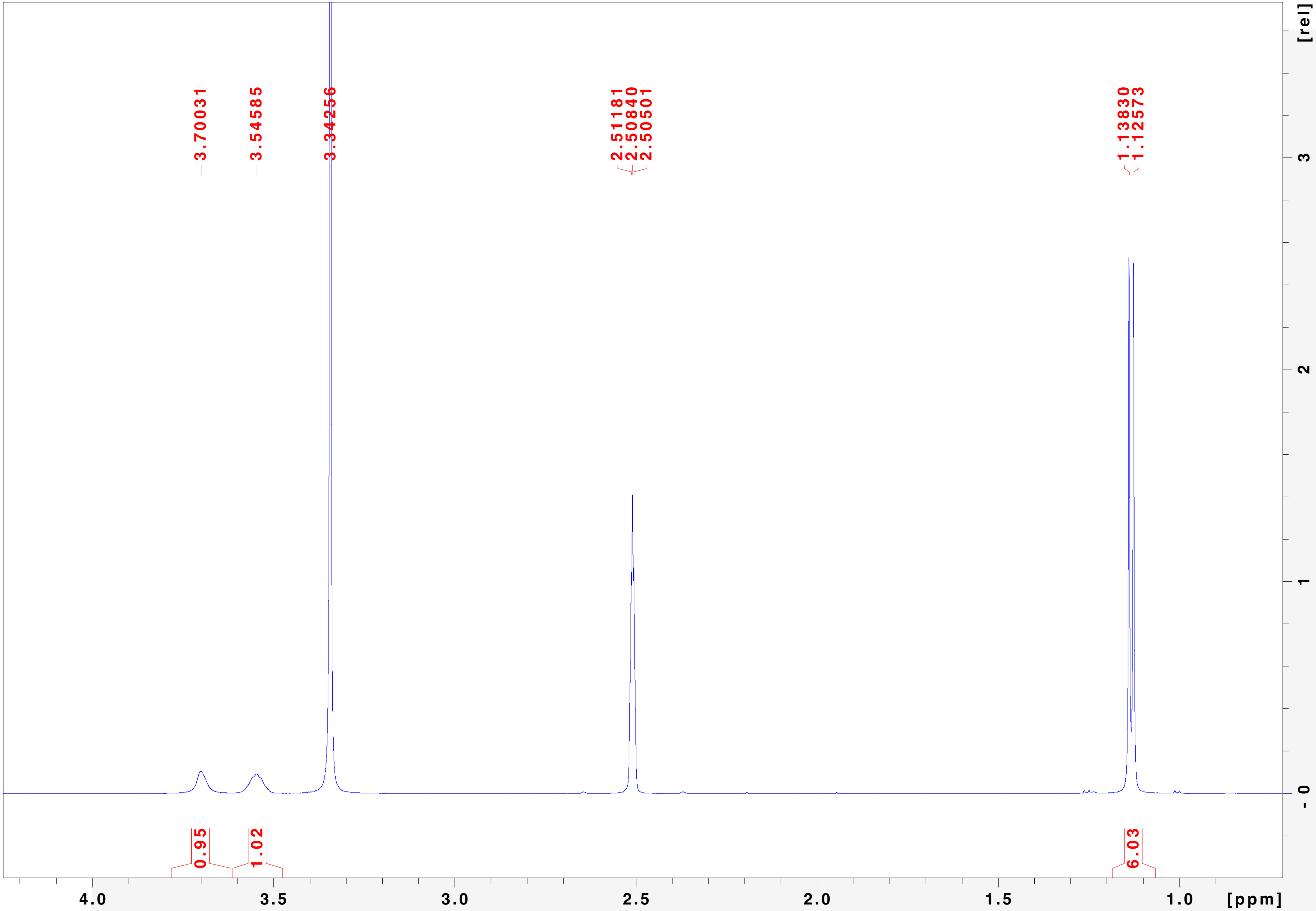

JI-III-49 20 1 /home/nmruser/networkdata/Bru500/terungwa@chem.byu.edu

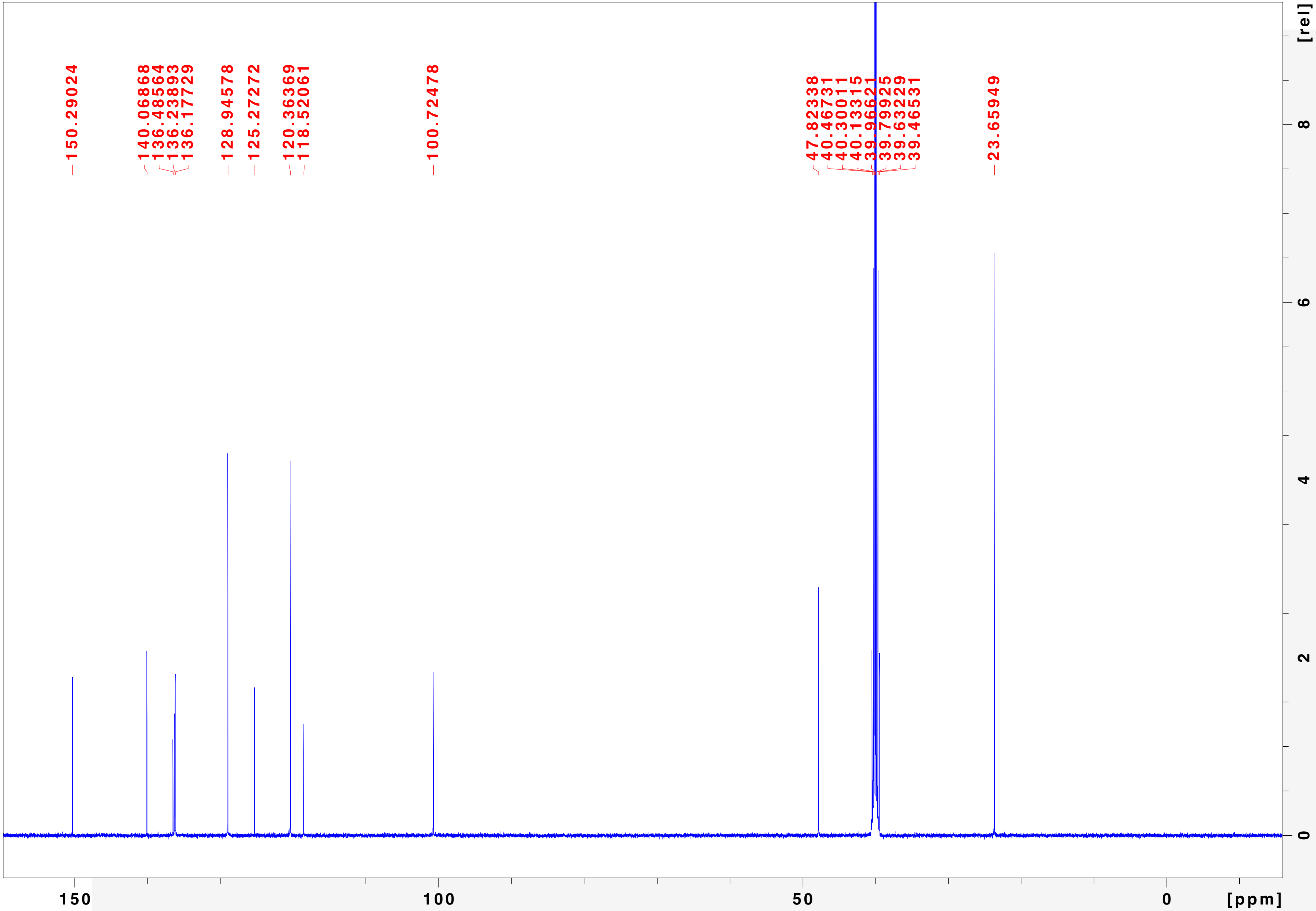

JI-III-48 10 1 /home/nmruser/networkdata/Bru500/terungwa@chem.byu.edu

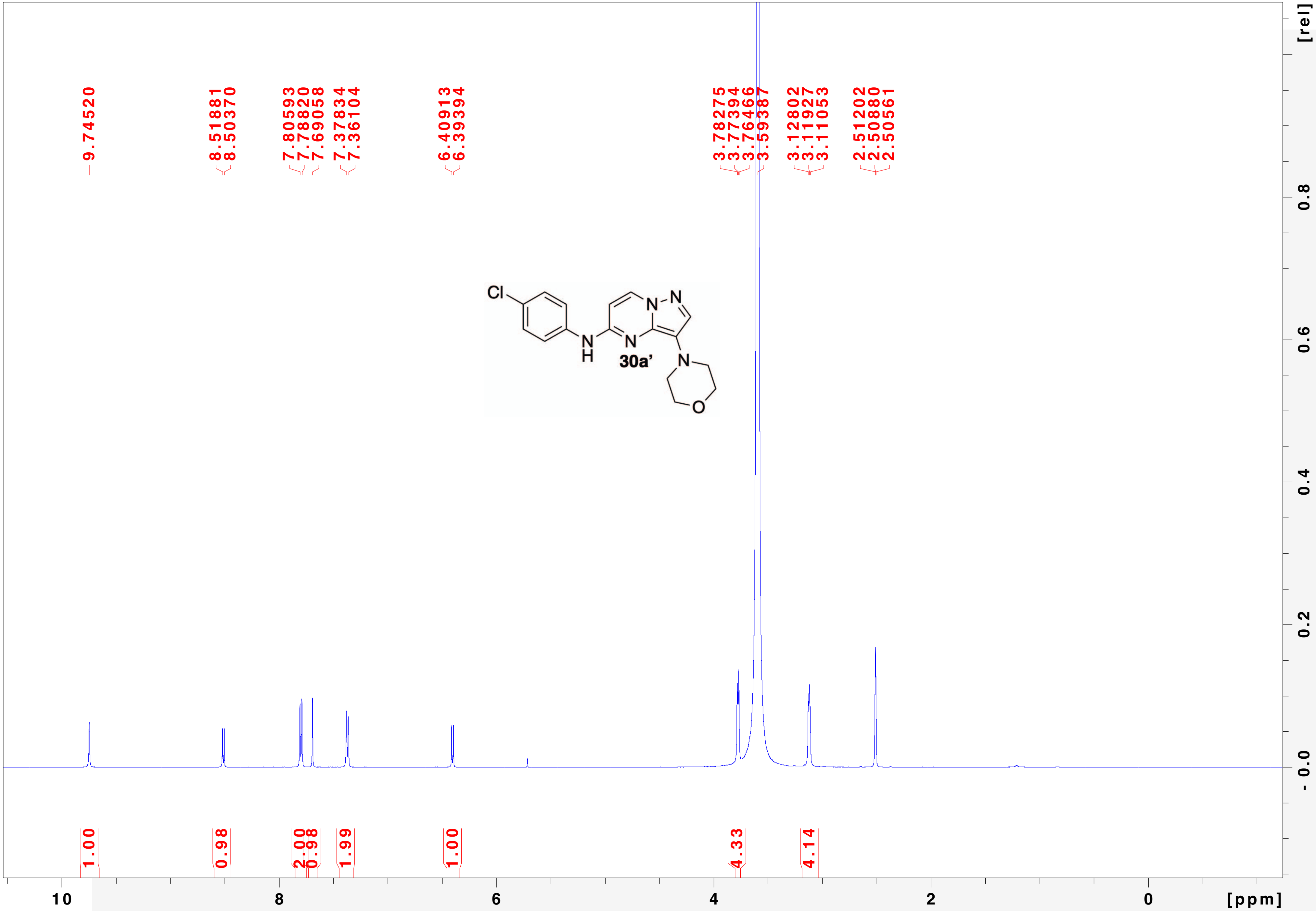

JI-III-48 10 1 /home/nmruser/networkdata/Bru500/terungwa@chem.byu.edu

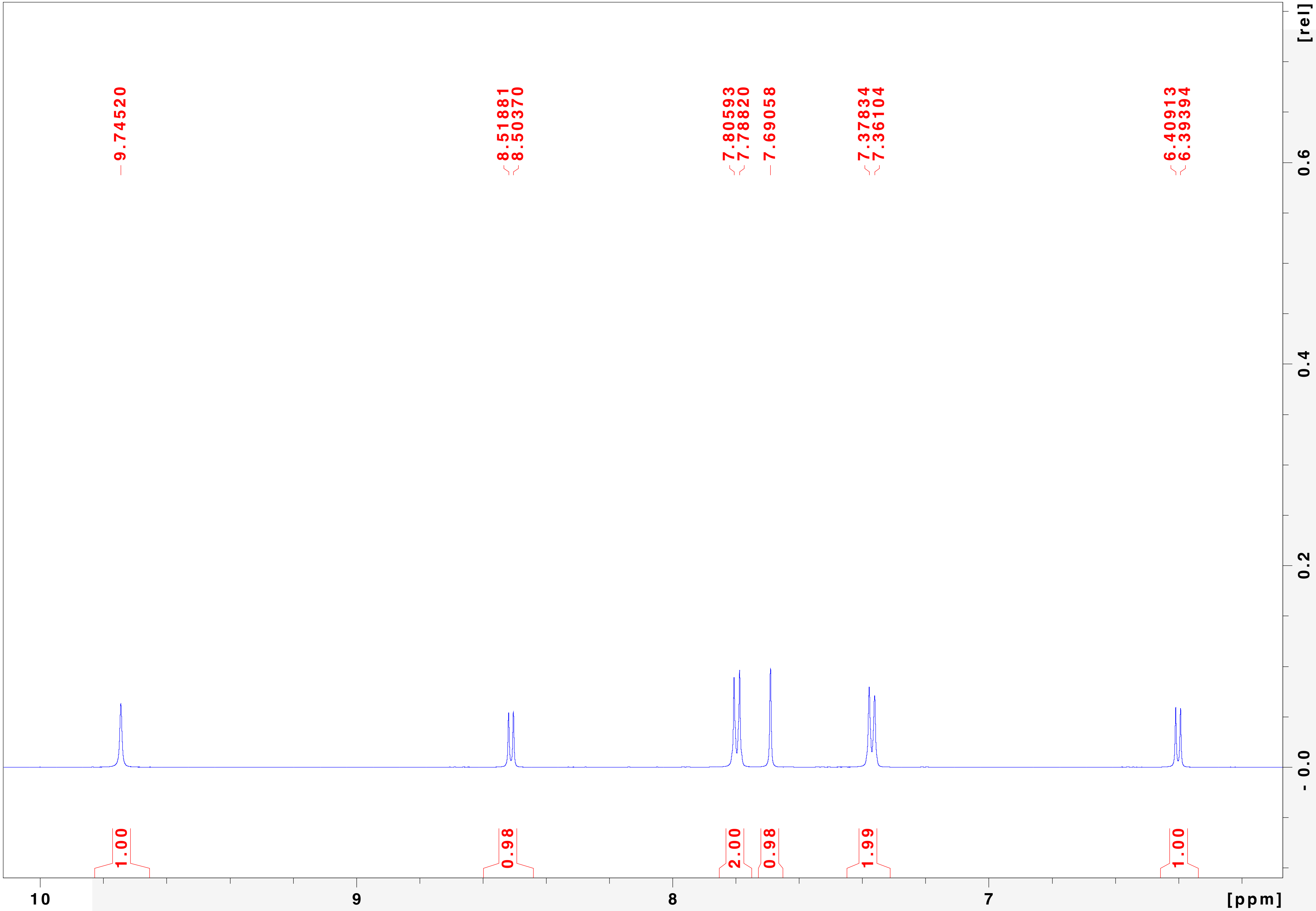

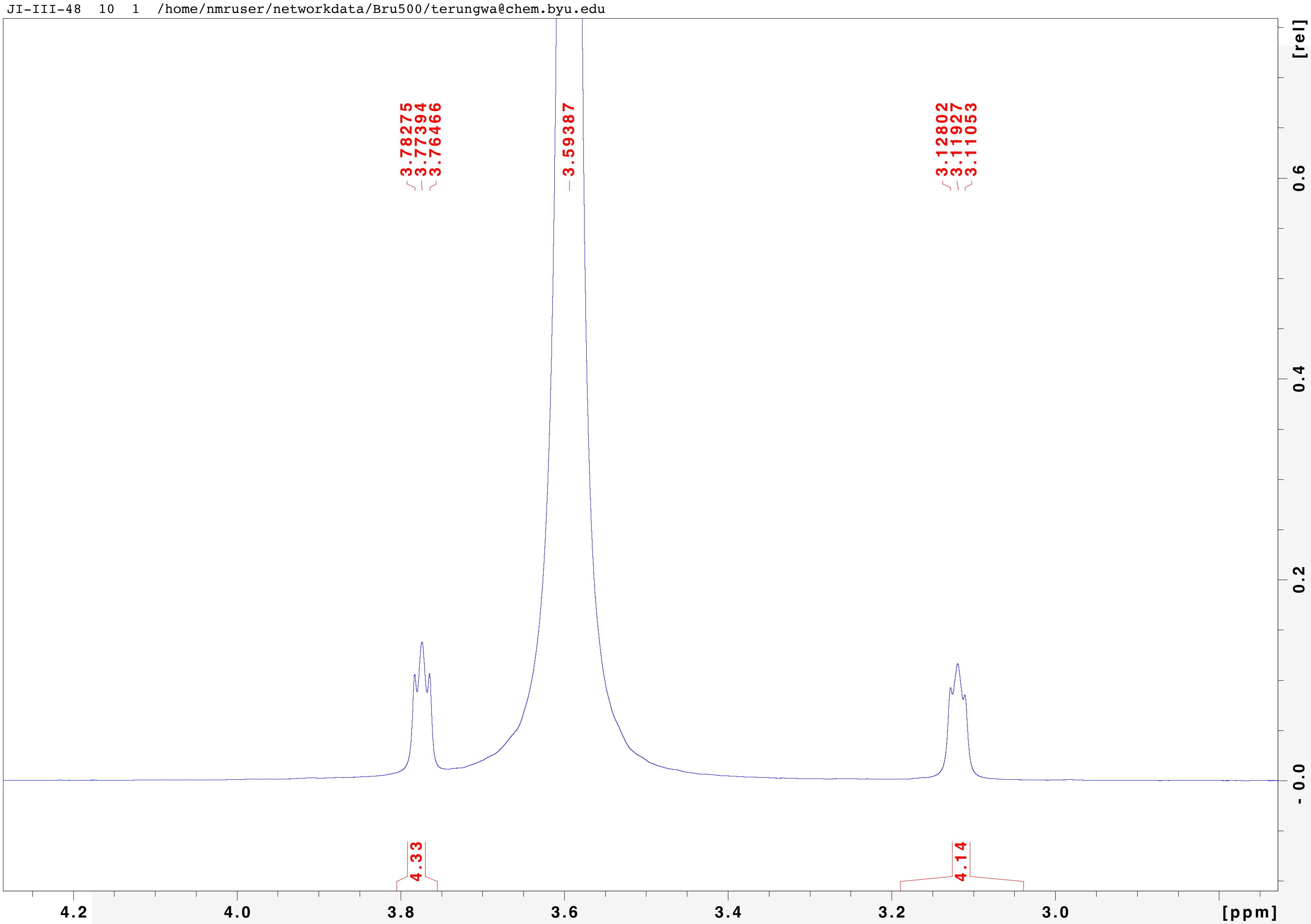
JI-III-48 10 1 /home/nmruser/networkdata/Bru500/terungwa@chem.byu.edu
3.78275
3.77394
3.76466
3.59387
3.12802
3.11927
3.11053
4.33
4.14
4.2
4.0
3.8
3.6
3.4
3.2
3.0
[ppm]
[rel]
0.6
0.4
0.2
- 0.0

JI-III-48 20 1 /home/nmruser/networkdata/Bru500/terungwa@chem.byu.edu

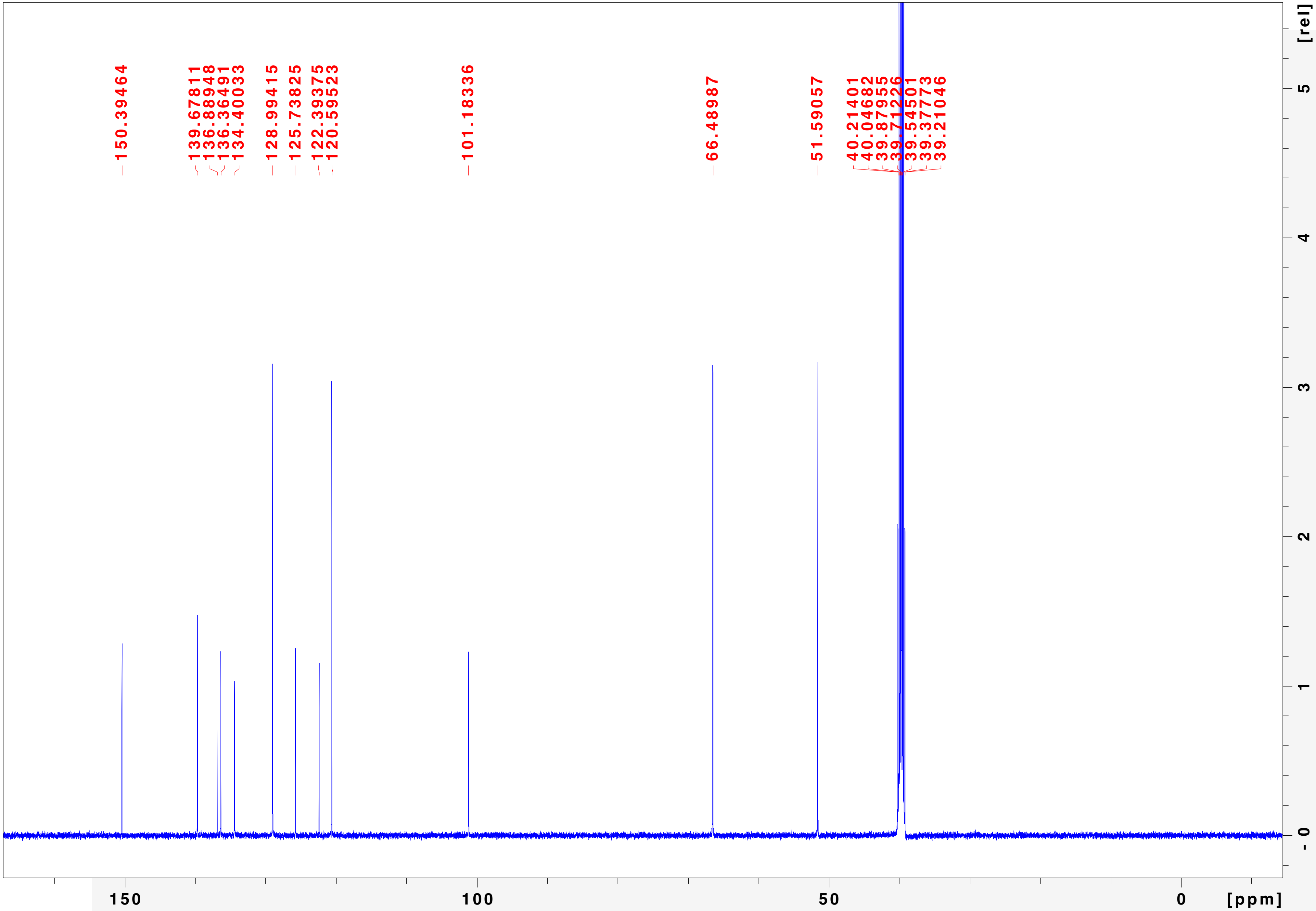

JI-III-50 20 1 /home/nmruser/networkdata/Bru500/terungwa@chem.byu.edu

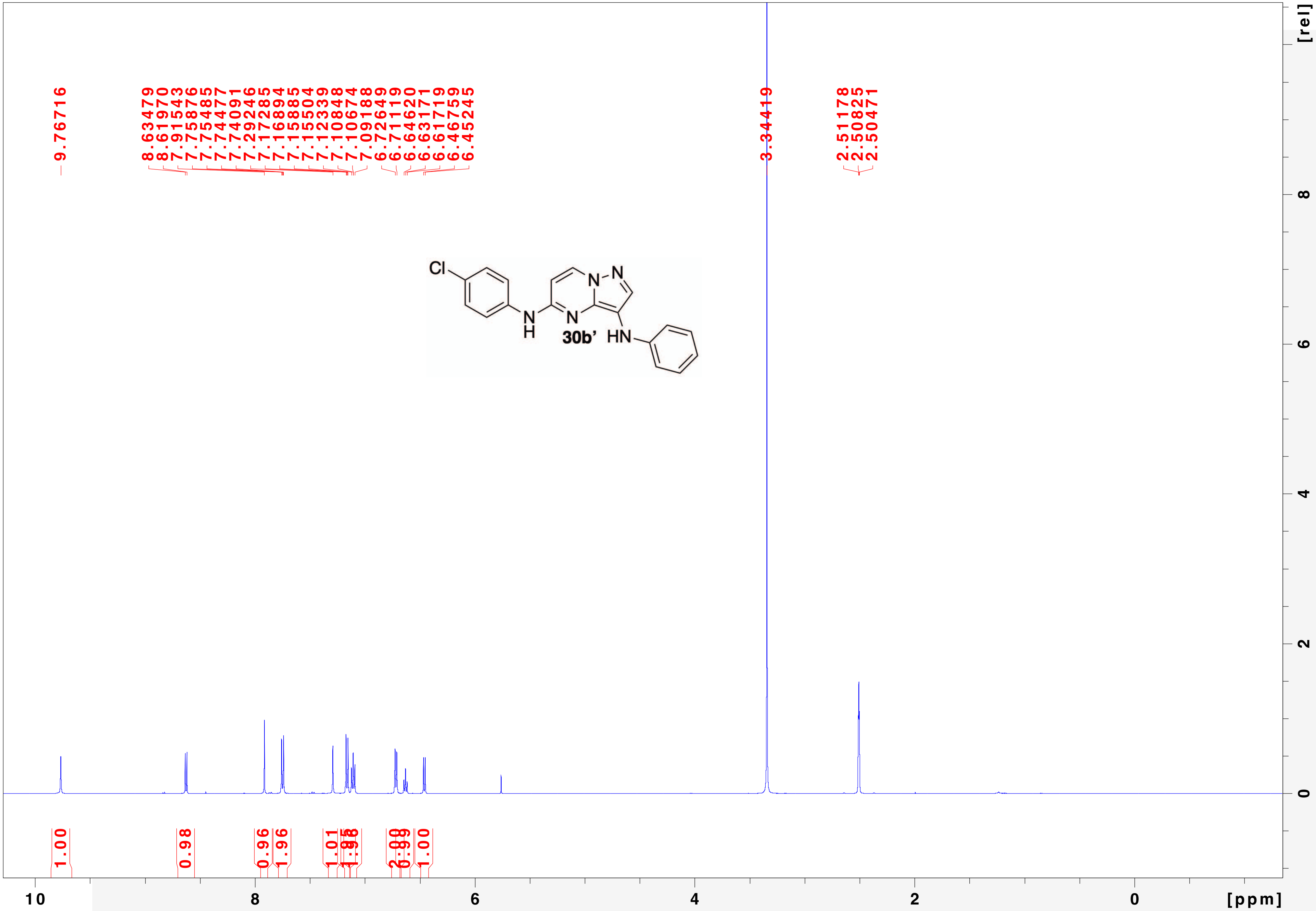

JI-III-50 20 1 /home/nmruser/networkdata/Bru500/terungwa@chem.byu.edu

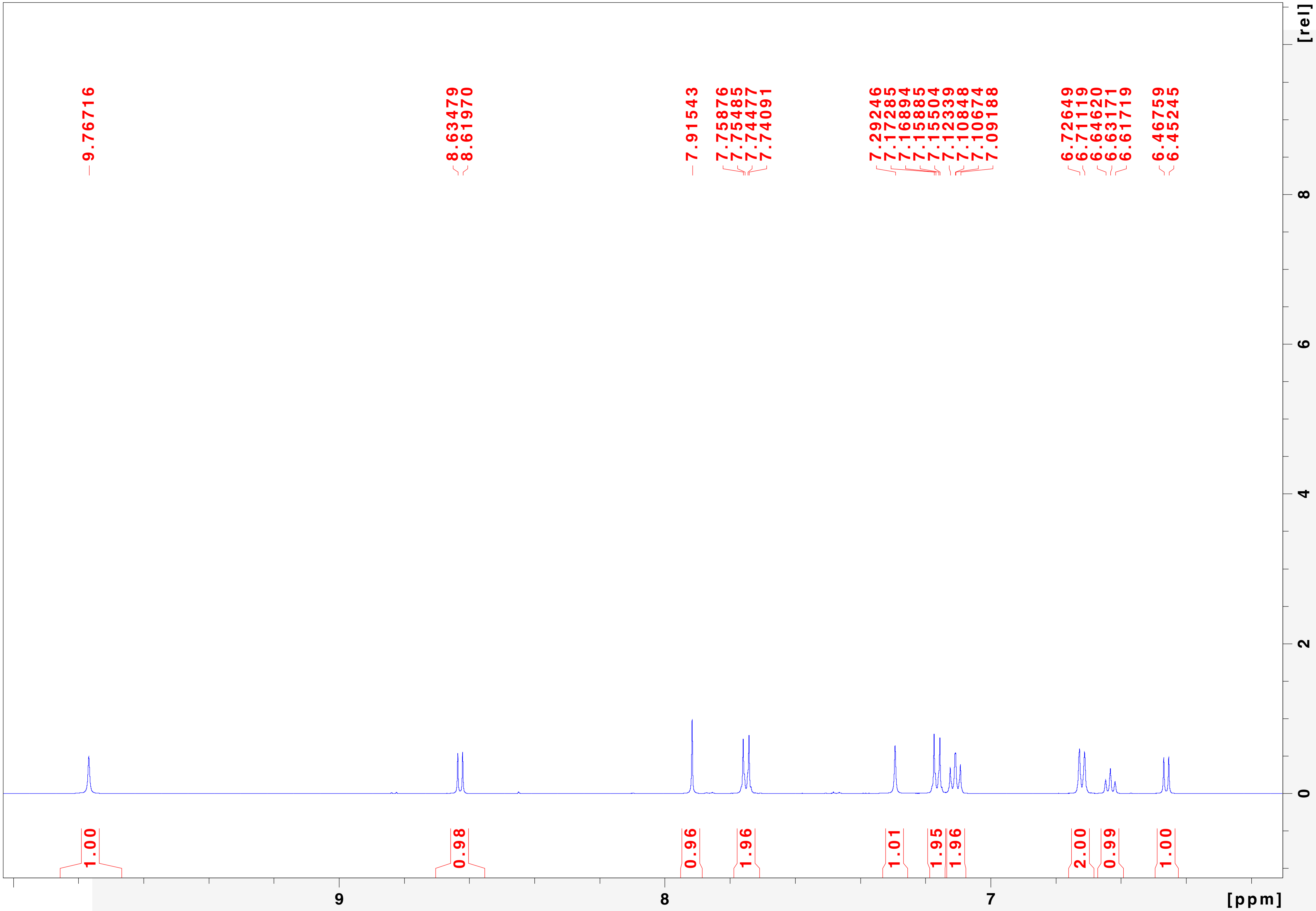

JI-III-50 30 1 /home/nmruser/networkdata/Bru500/terungwa@chem.byu.edu

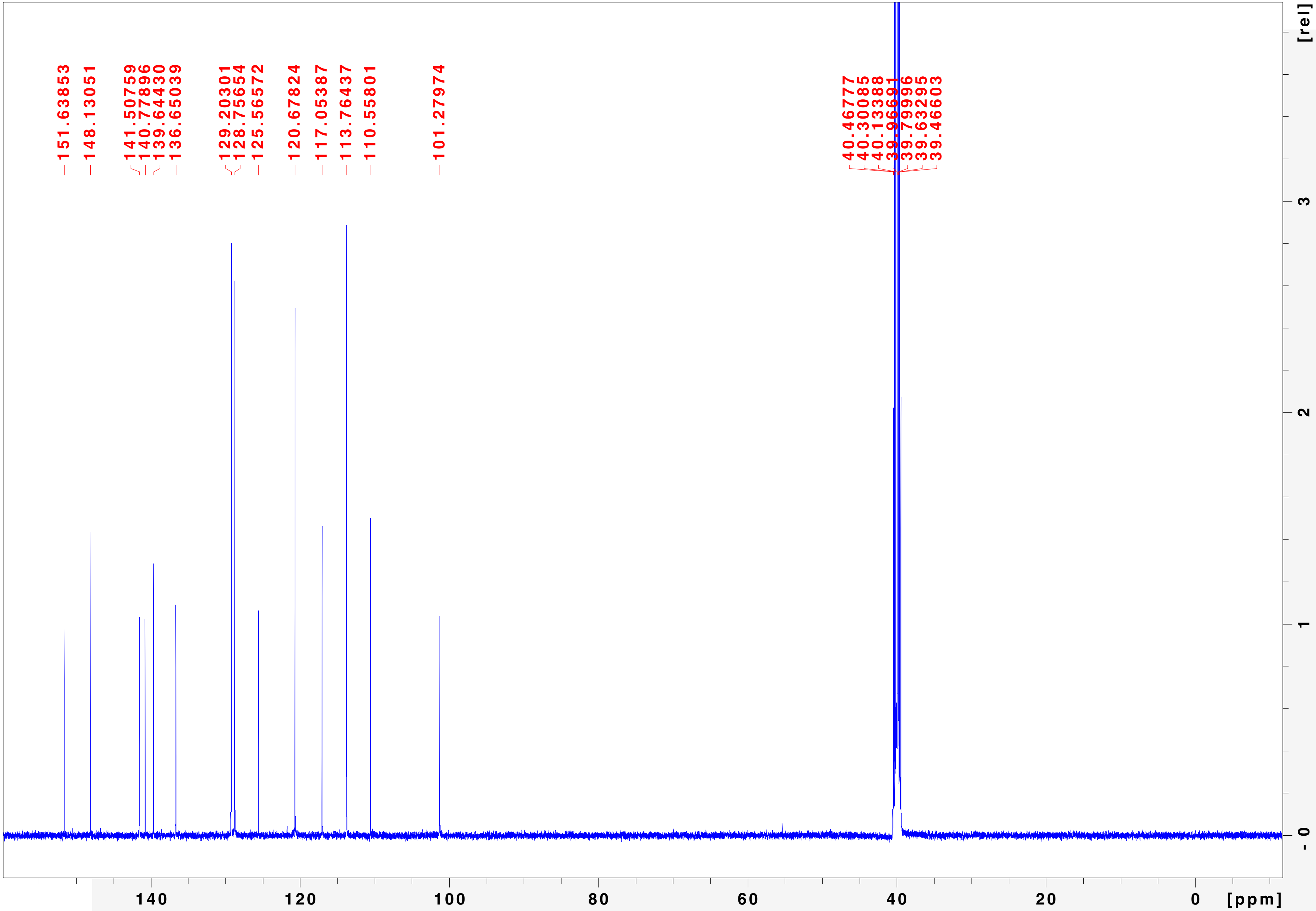

JI-III-52 40 1 /home/nmruser/networkdata/Bru500/terungwa@chem.byu.edu

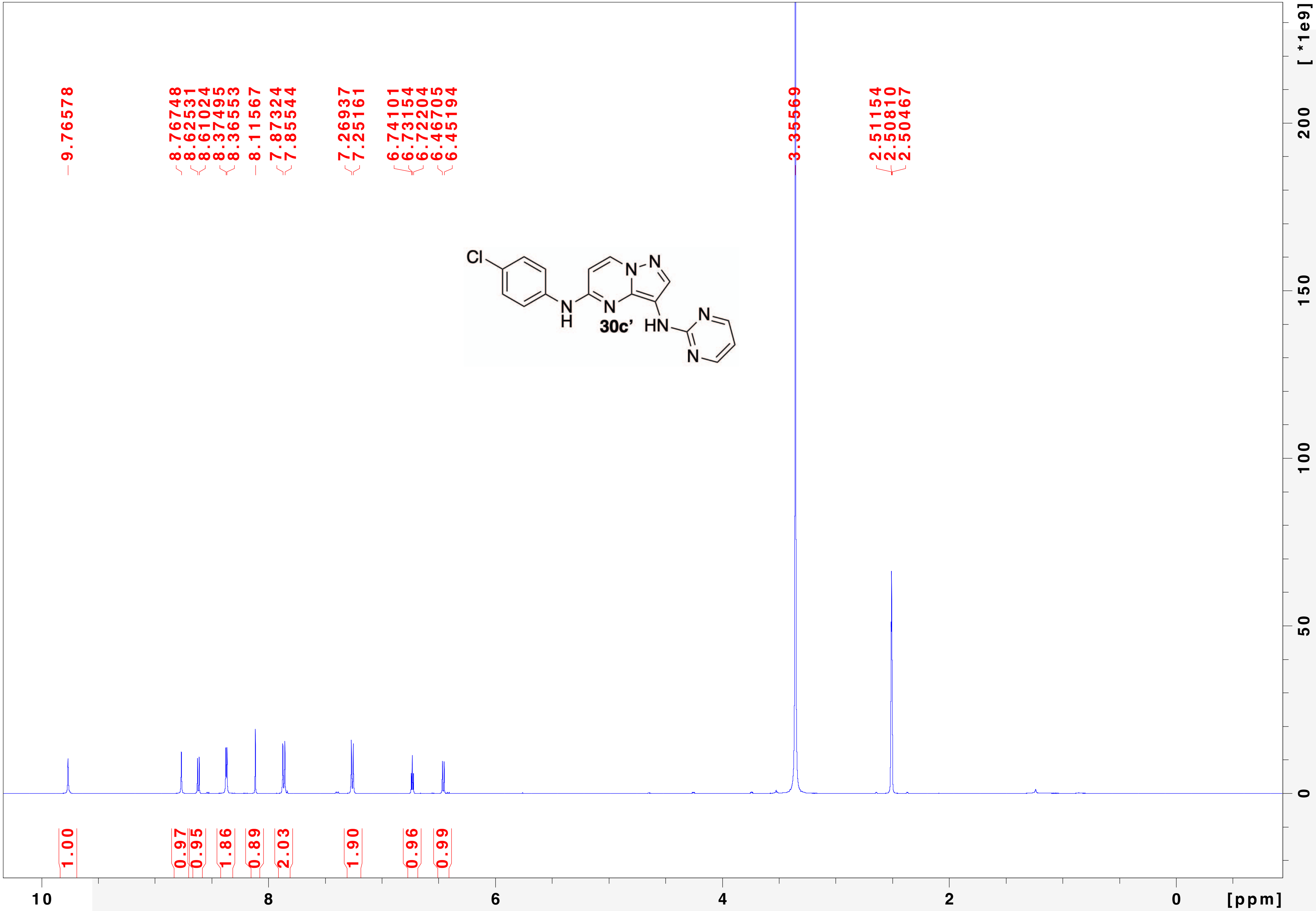

JI-III-52 40 1 /home/nmruser/networkdata/Bru500/terungwa@chem.byu.edu

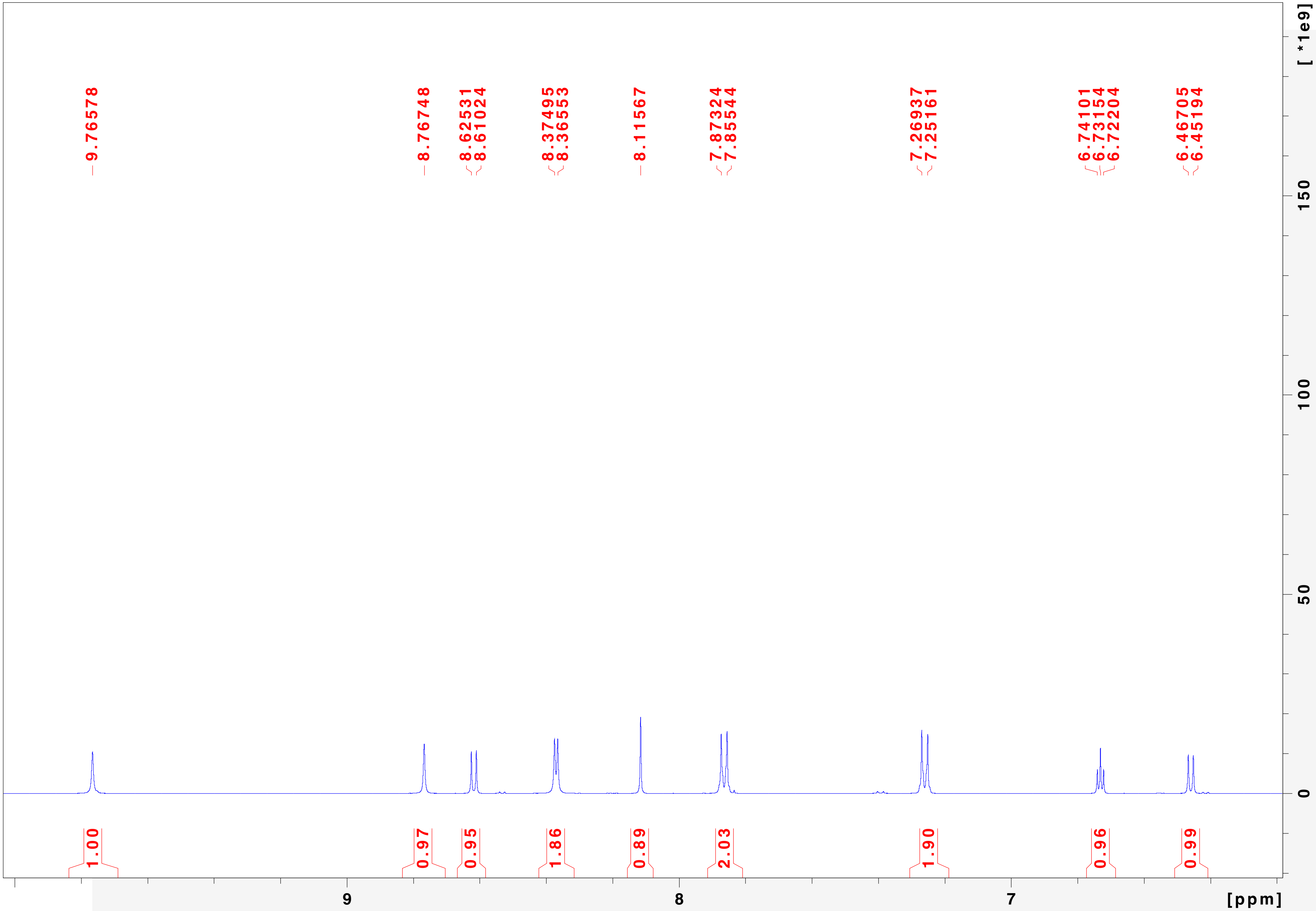

JI-III-52 50 1 /home/nmruser/networkdata/Bru500/terungwa@chem.byu.edu

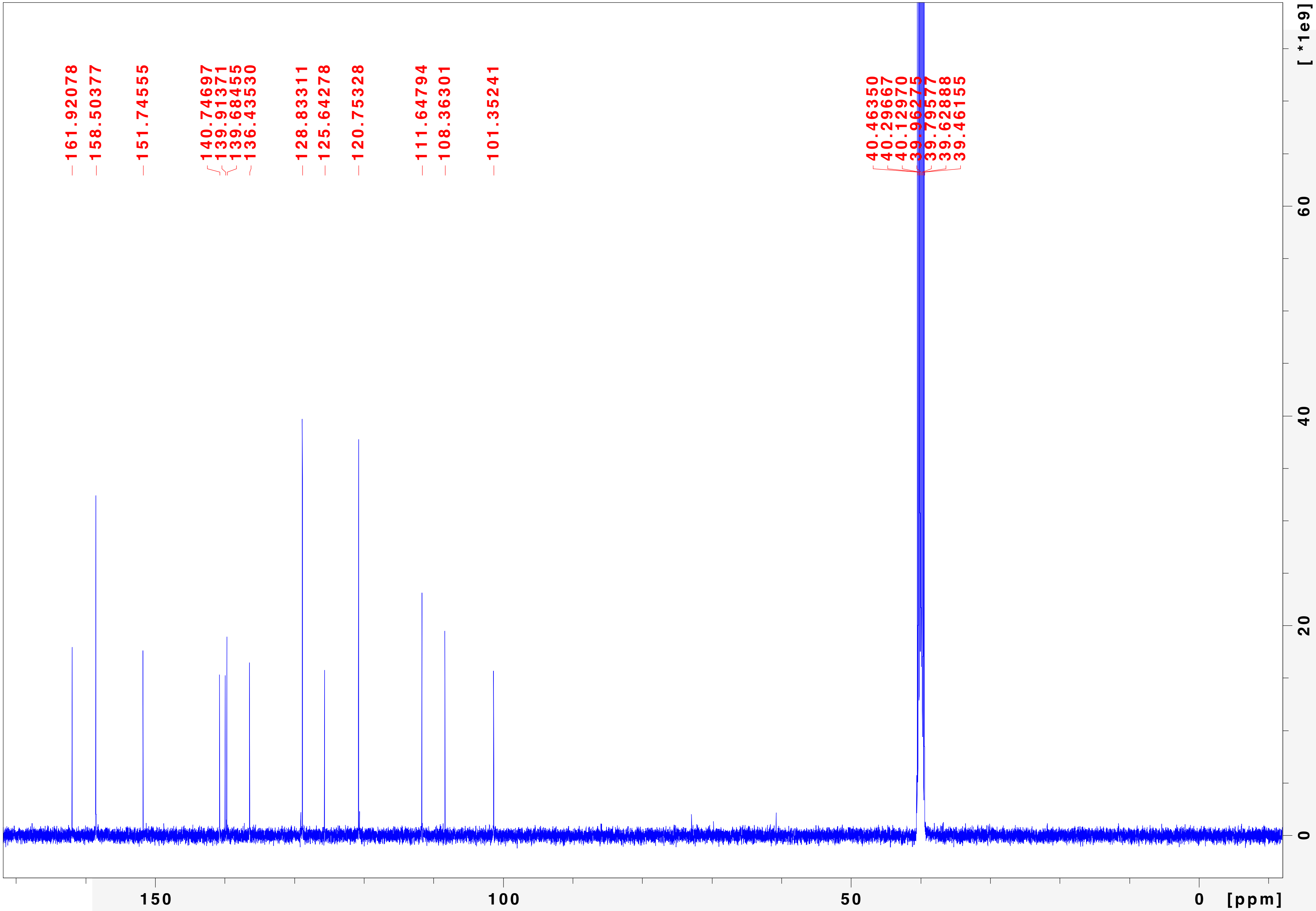

JI-II-97 10 1 /home/nmruser/networkdata/Bruker500Data/terungwa@chem.byu.edu

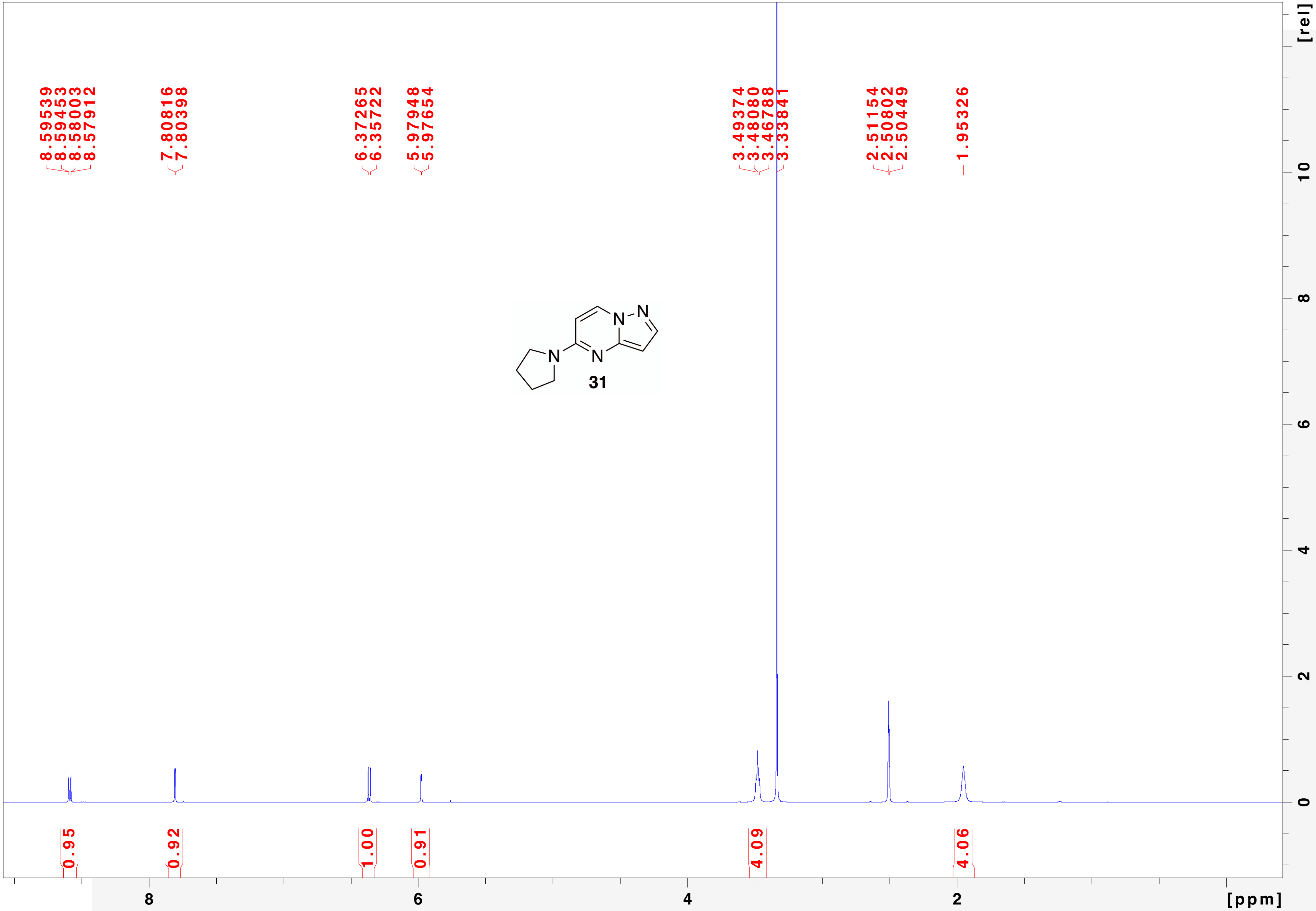

JI-II-97 10 1 /home/nmruser/networkdata/Bruker500Data/terungwa@chem.byu.edu

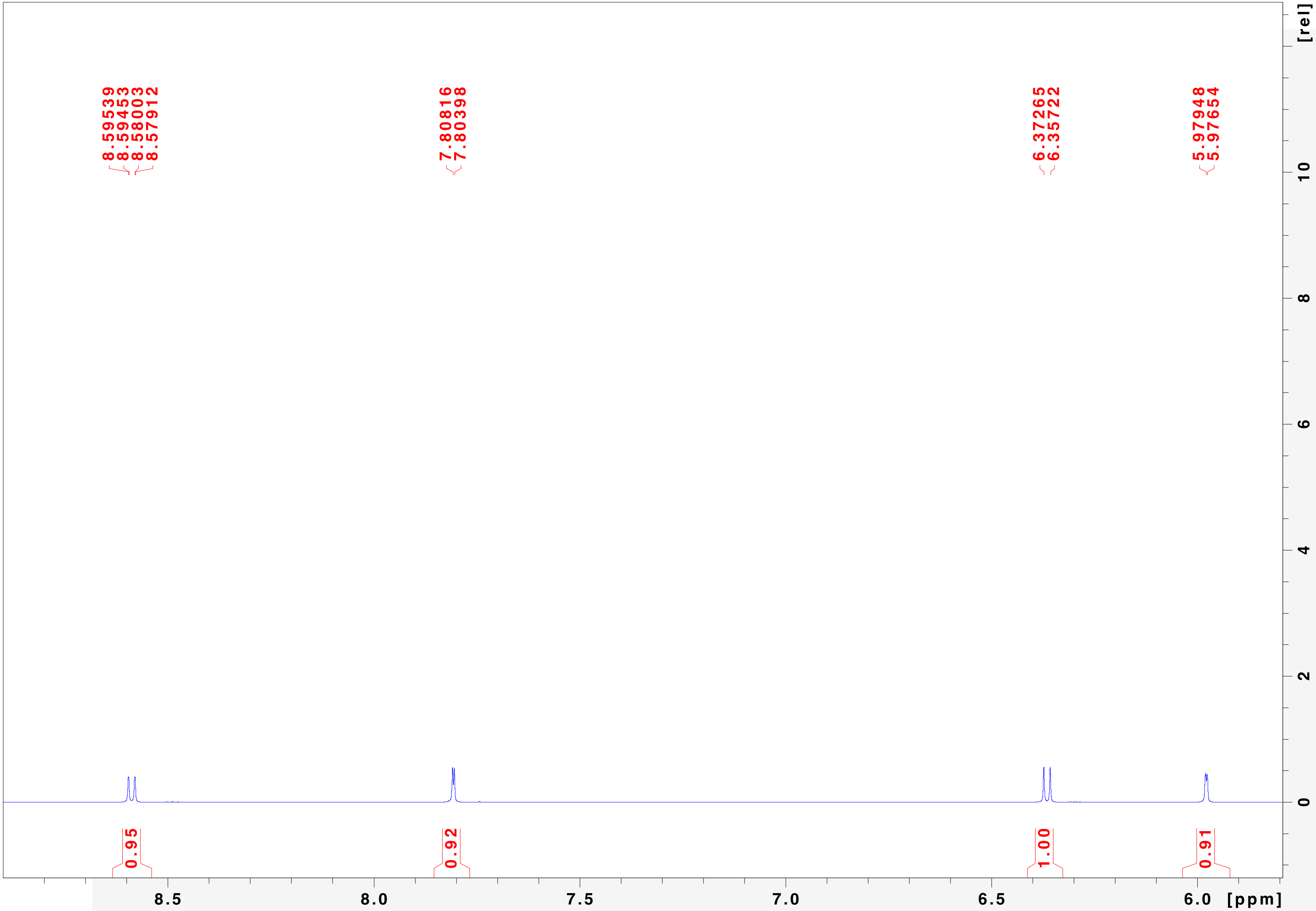

JI-II-97 20 1 /home/nmruser/networkdata/Bruker500Data/terungwa@chem.byu.edu

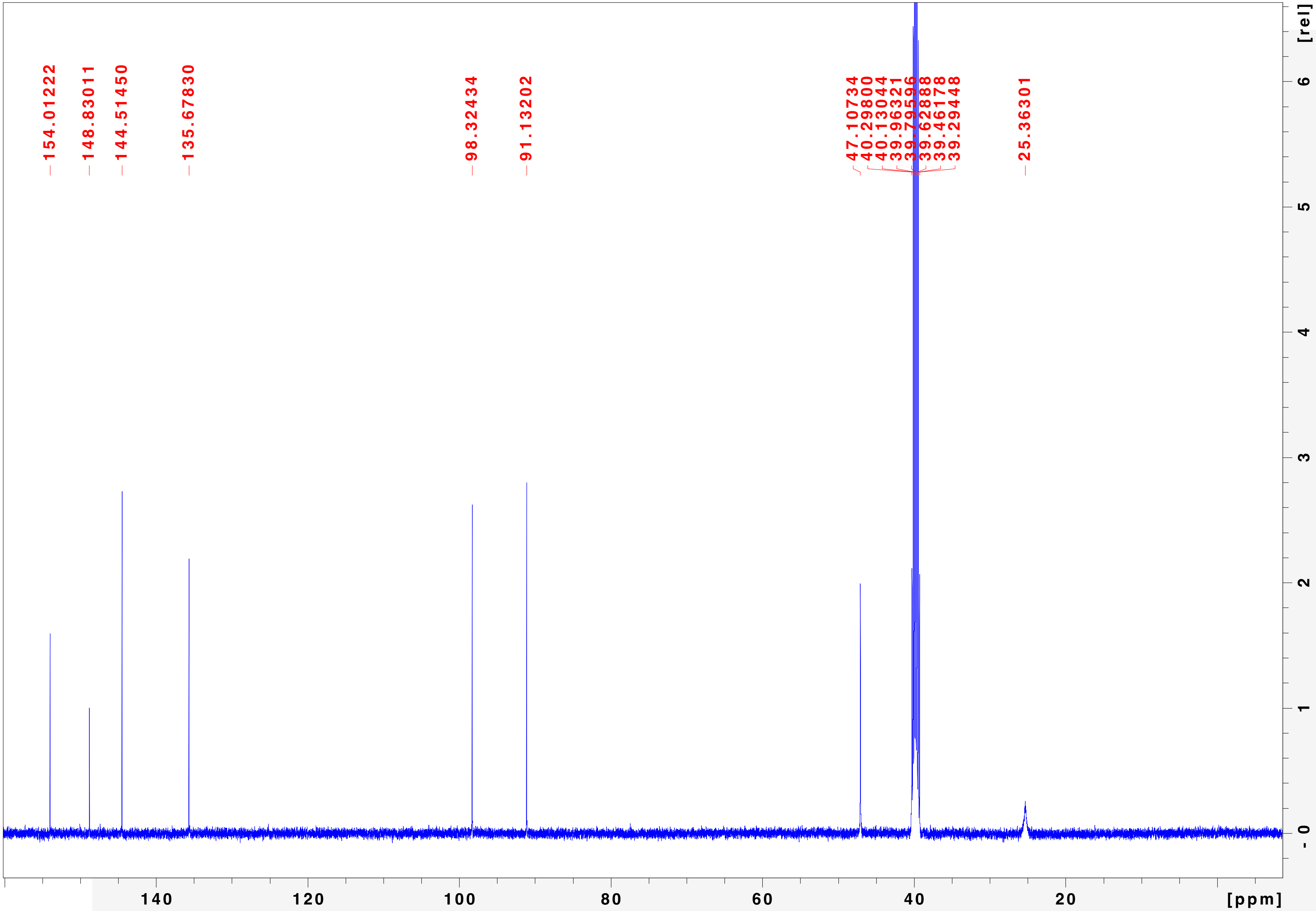

JI-II-111 10 1 /home/nmruser/networkdata/terungwa@chem.byu.edu

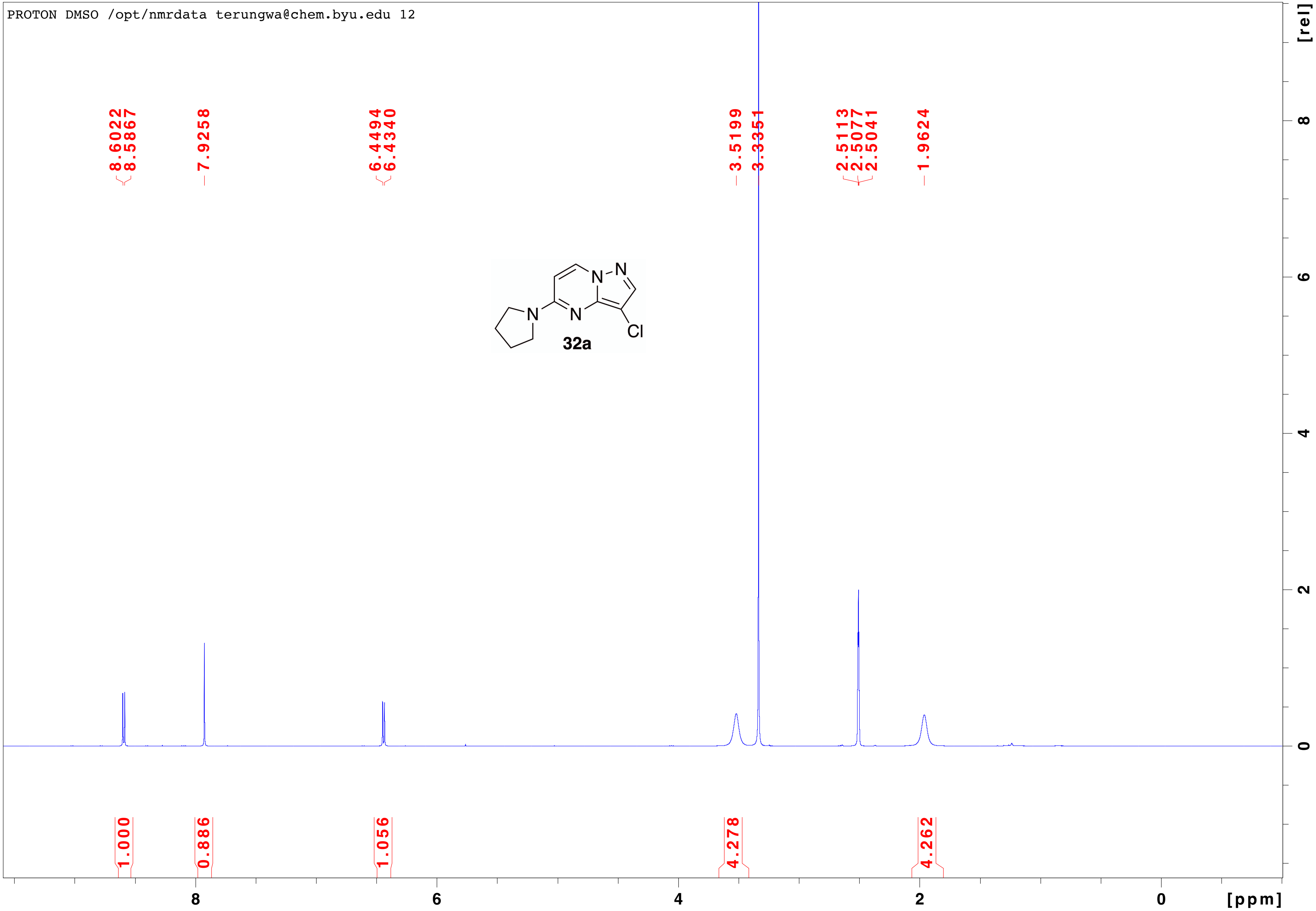

JI-II-111 20 1 /home/nmruser/networkdata/terungwa@chem.byu.edu

C13CPD DMSO /opt/nmrdata terungwa@chem.byu.edu 19

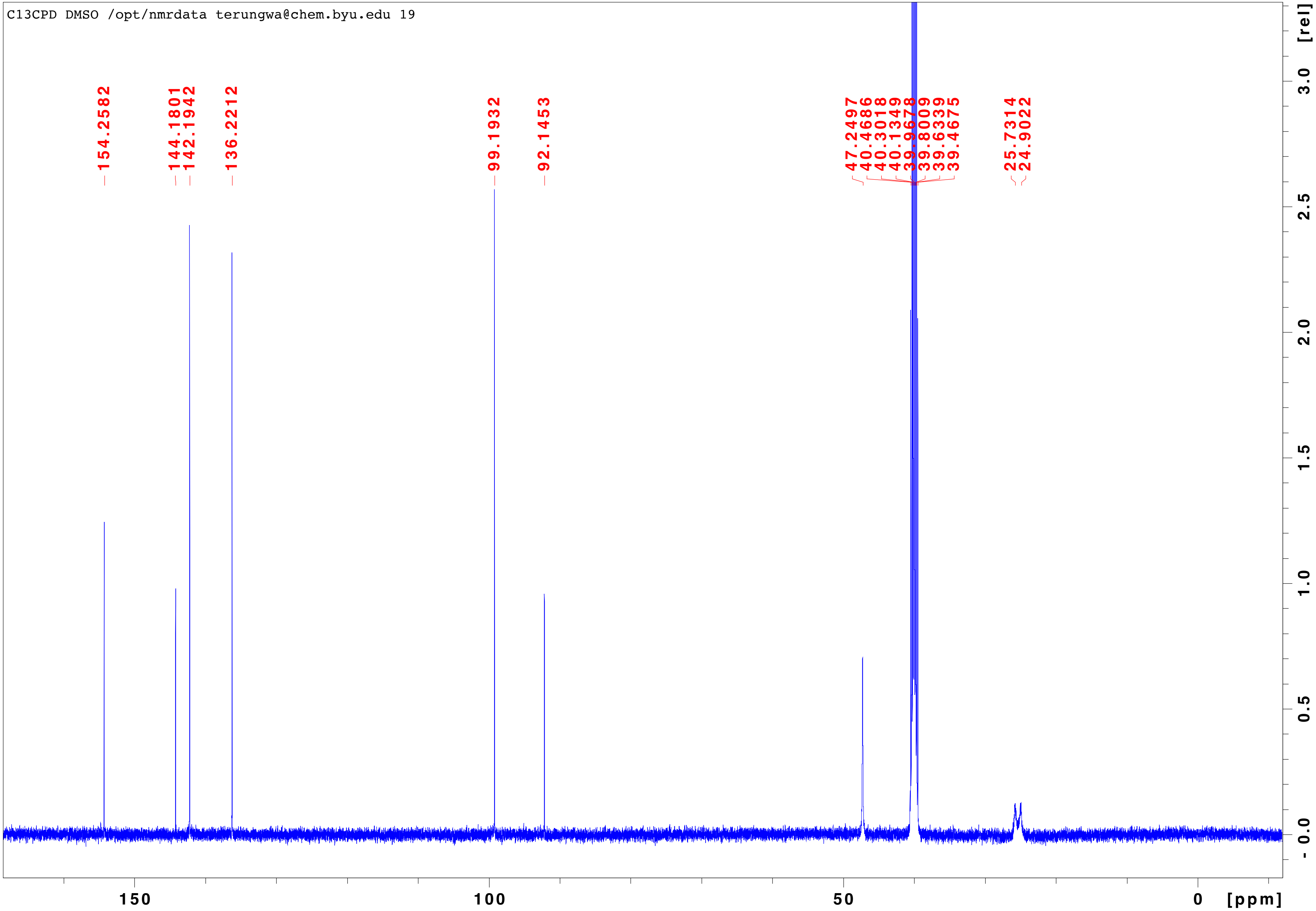

JI-II-112 10 1 /home/nmruser/networkdata/terungwa@chem.byu.edu

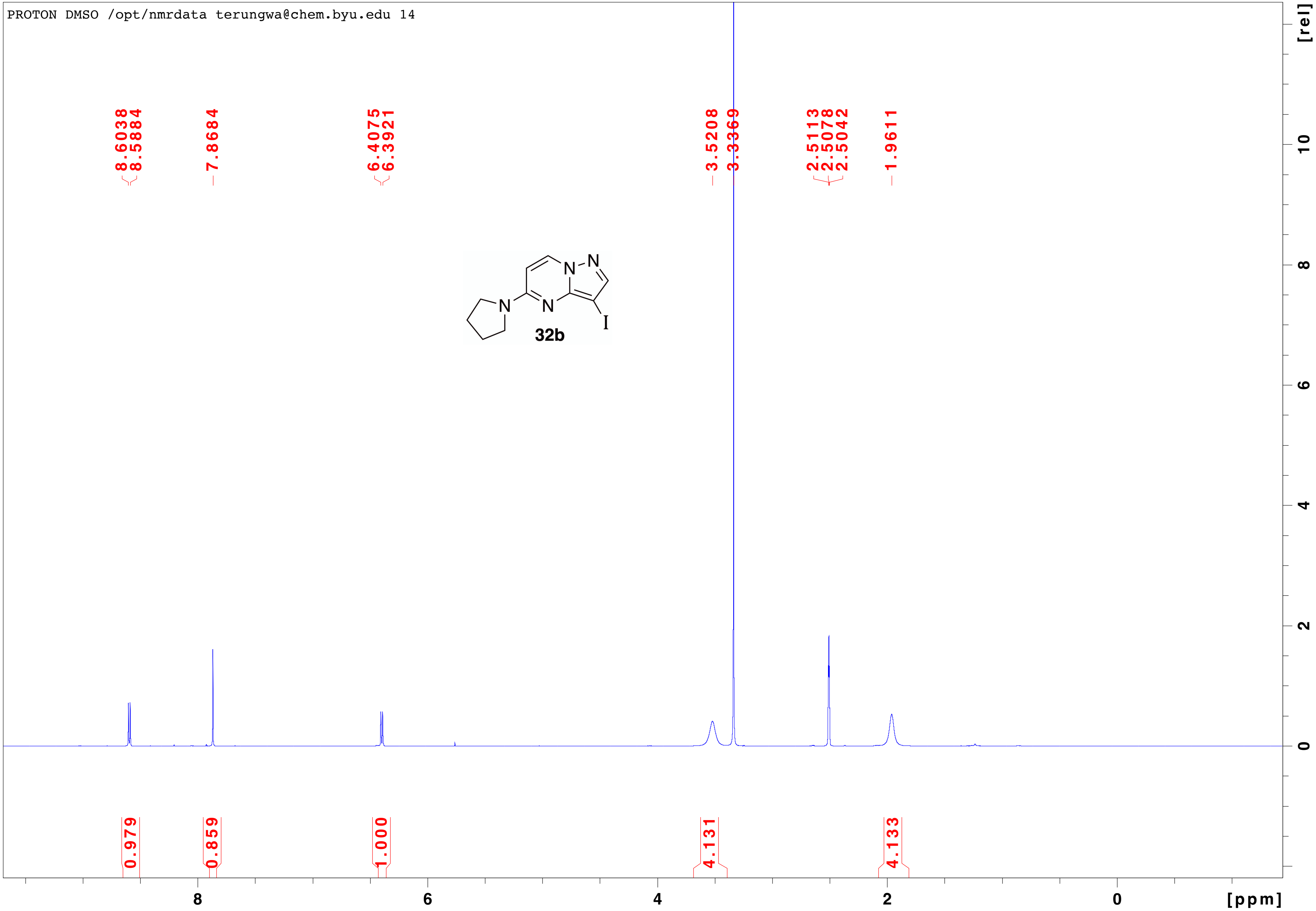

JI-II-112 20 1 /home/nmruser/networkdata/terungwa@chem.byu.edu

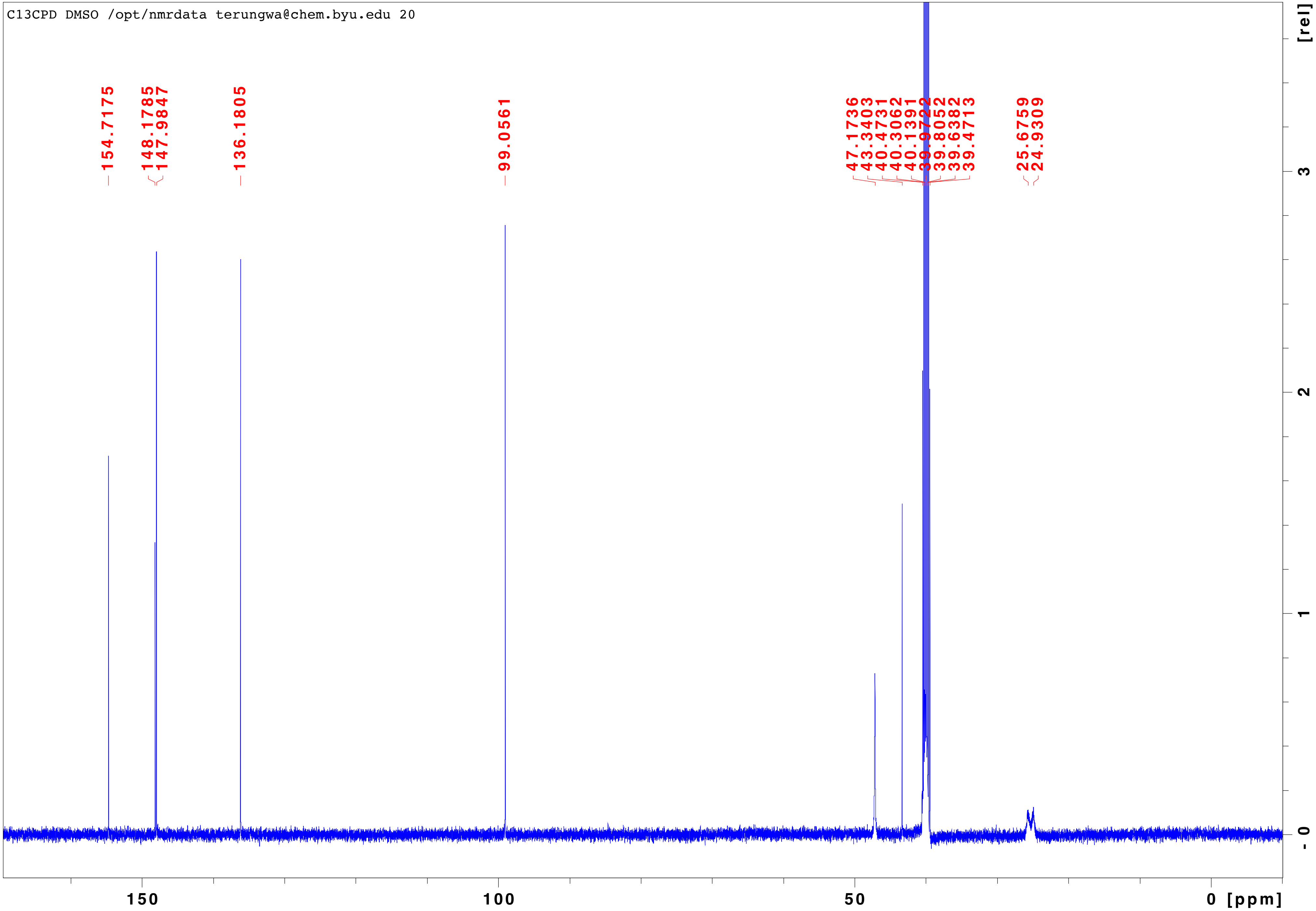

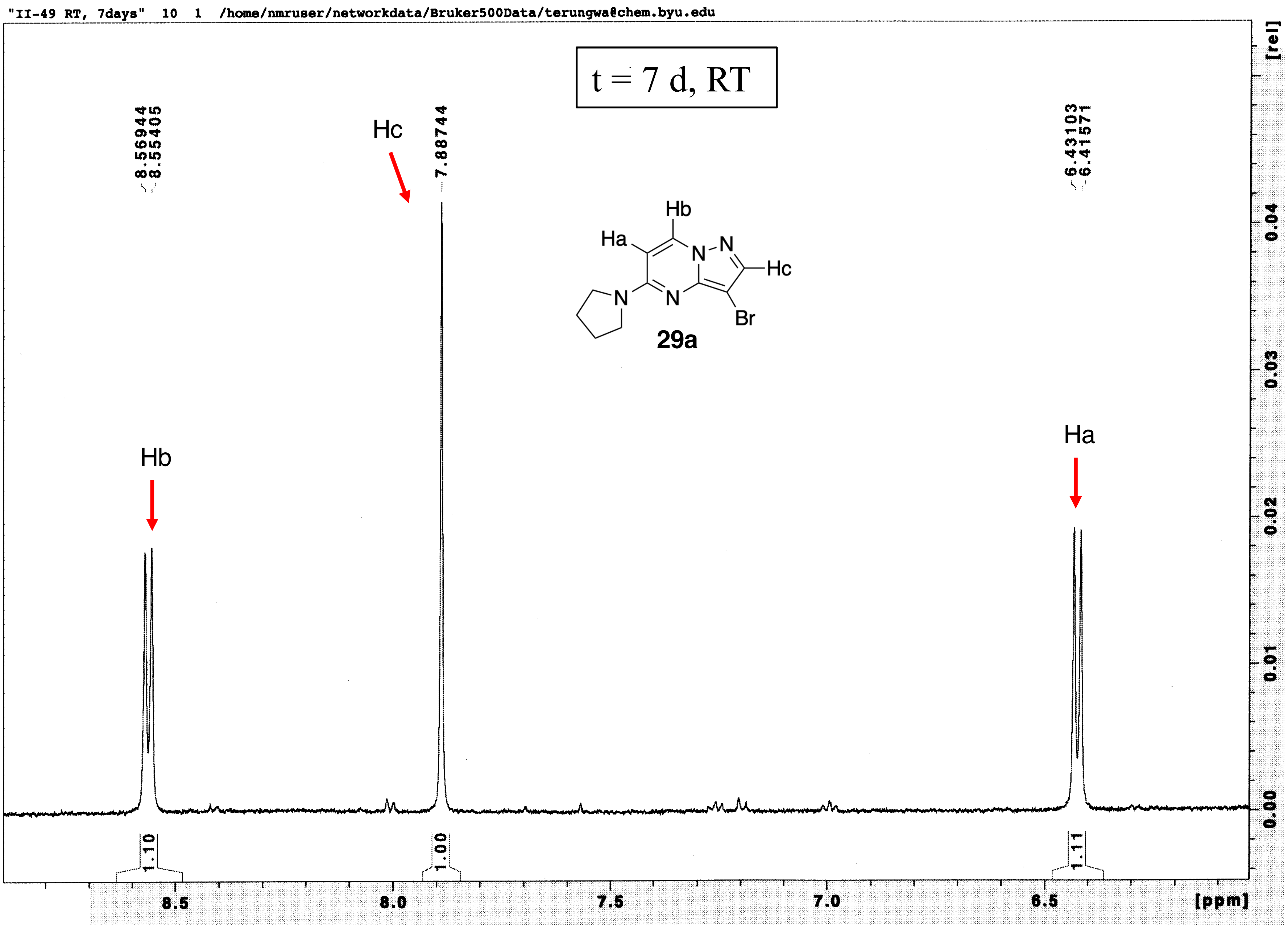
"II-49 RT, 7days" 10 1 /home/nmruser/networkdata/Bruker500Data/terungwa@chem.byu.edu
t = 7 d, RT
8.56944
8.55405
Hc
7.88744
6.43103
6.41571
Hb
Ha
N
N
N
N
Hc
Br
29a
Hb
Ha
1.10
1.00
1.11
8.5
8.0
7.5
7.0
6.5
[ppm]
[rel]
0.04
0.03
0.02
0.01
0.00

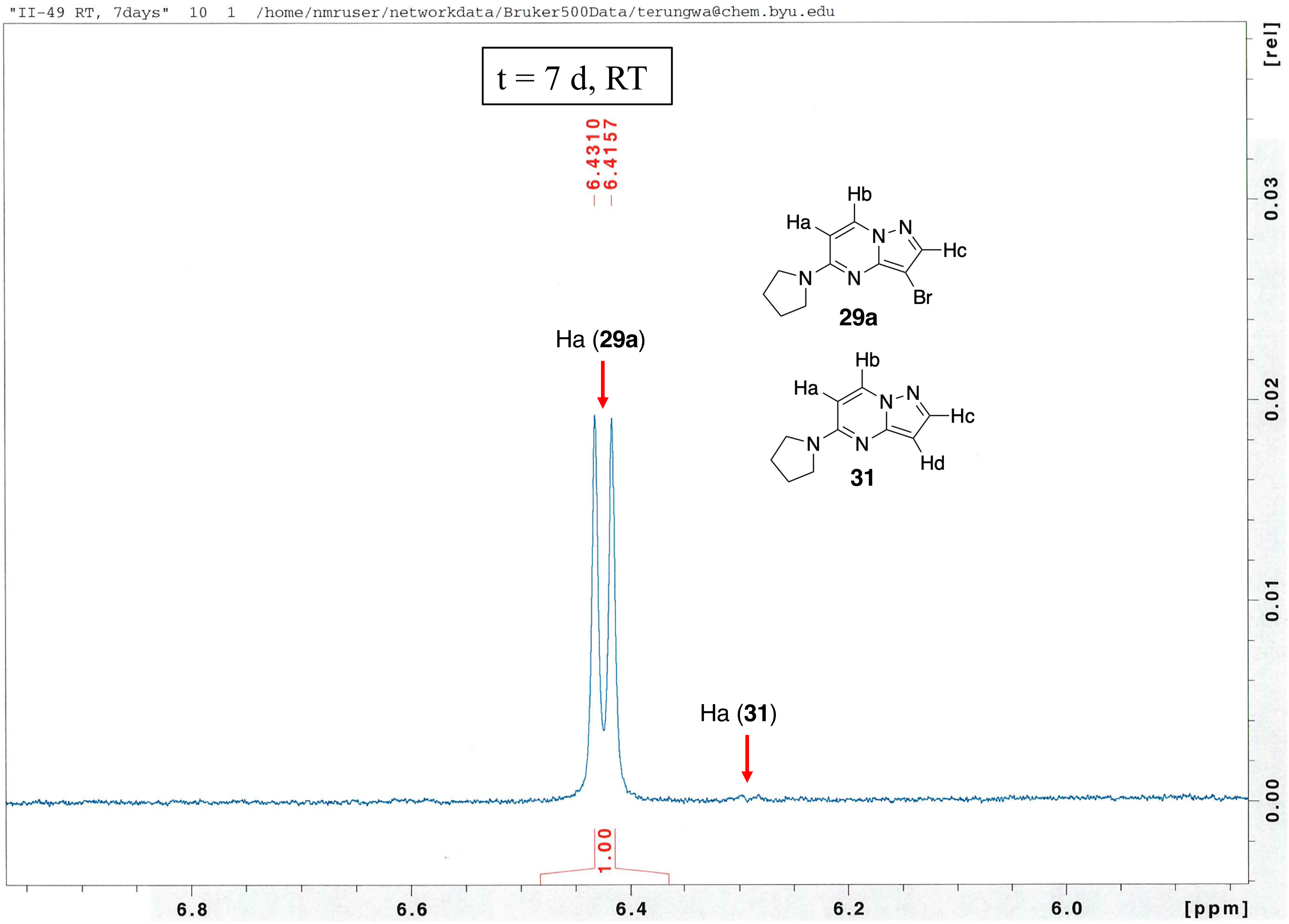
"II-49 RT, 7days" 10 1 /home/nmruser/networkdata/Bruker500Data/terungwa@chem.byu.edu
t = 7 d, RT
6.4310
6.4157
Ha (29a)
Ha (31)
Hb
Ha
Hc
Br
29a
Hd
31
1.00
6.8
6.6
6.4
6.2
6.0
[ppm]
[rel]
0.03
0.02
0.01
0.00

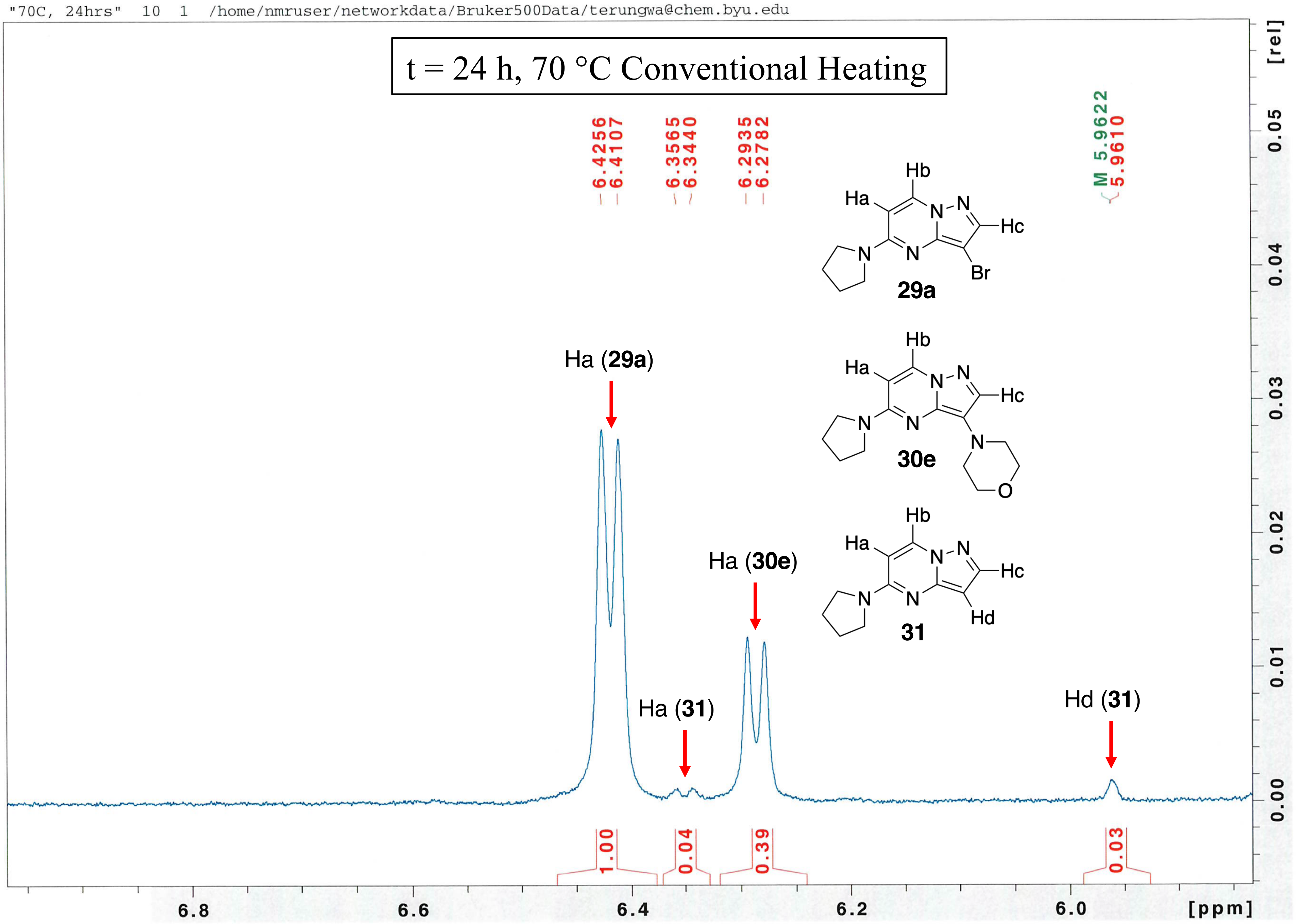
"70C, 24hrs" 10 1 /home/nmruser/networkdata/Bruker500Data/terungwa@chem.byu.edu
t = 24 h, 70 °C Conventional Heating
6.4256
6.4107
6.3565
6.3440
6.2935
6.2782
M 5.9622
5.9610
Hb
Ha
N-N
Hc
N
N
Br
29a
Hb
Ha
N-N
Hc
N
N
N
O
30e
Hb
Ha
N-N
Hc
N
N
Hd
31
Ha (29a)
Ha (30e)
Ha (31)
Hd (31)
1.00
0.04
0.39
0.03
6.8
6.6
6.4
6.2
6.0
[ppm]
0.00
0.01
0.02
0.03
0.04
0.05
[rel]

"II-49 70C 30min"   10   1   /home/nmruser/networkdata/Bruker500Data/terungwa@chem.byu.edu

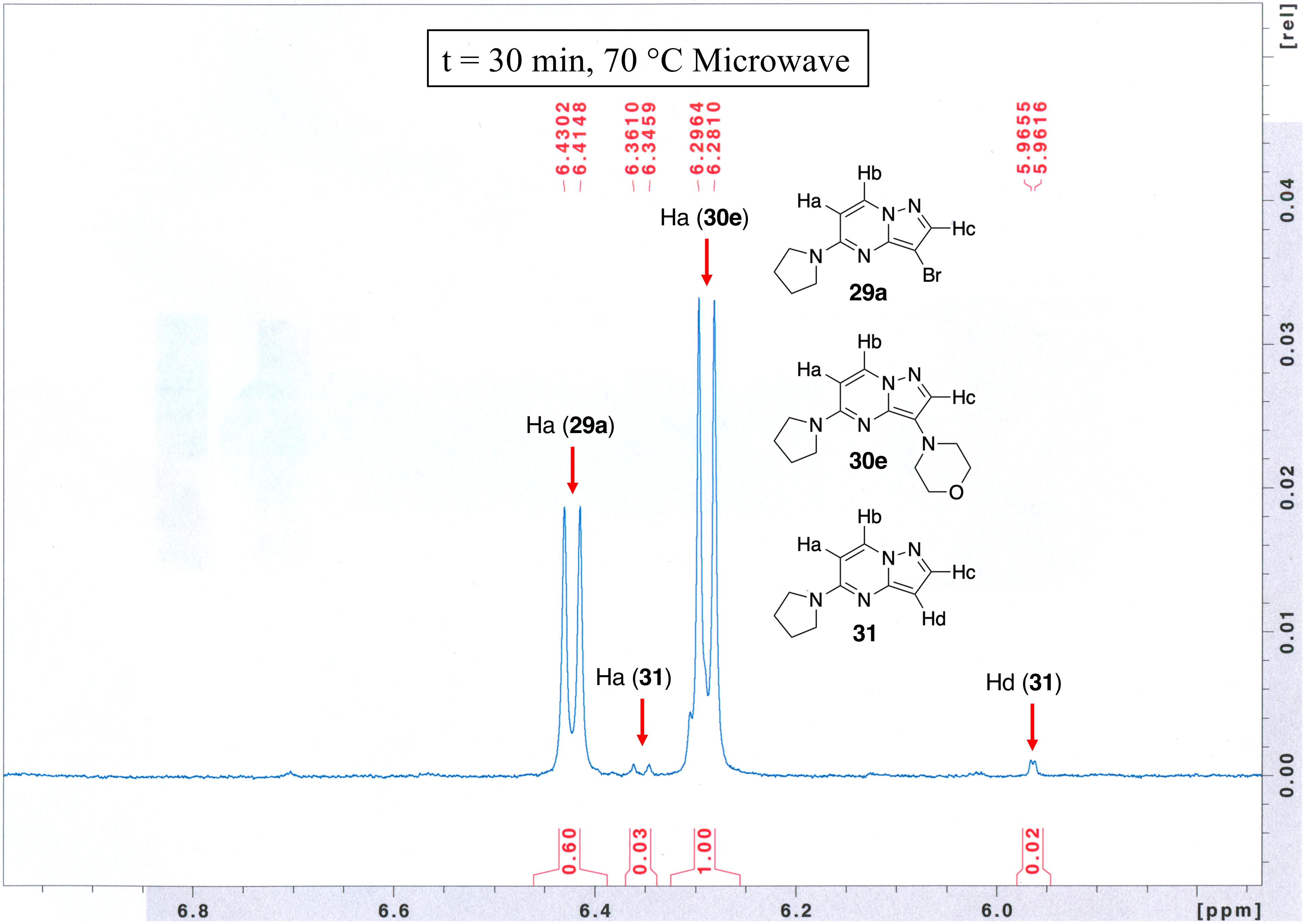

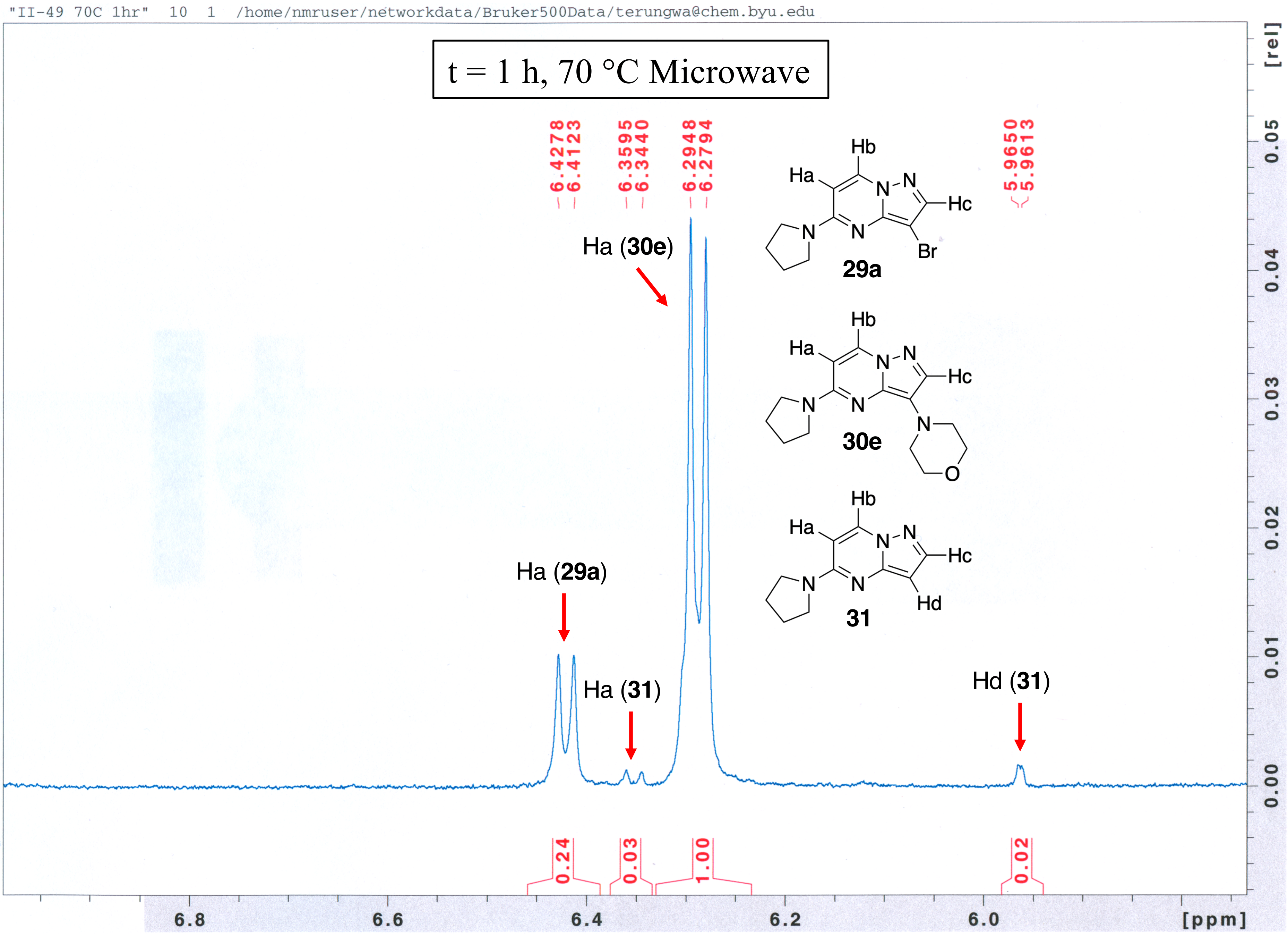
"II-49 70C 1hr" 10 1 /home/nmruser/networkdata/Bruker500Data/terungwa@chem.byu.edu
t = 1 h, 70 °C Microwave
6.4278
6.4123
6.3595
6.3440
6.2948
6.2794
5.9650
5.9613
Ha (30e)
Ha (29a)
Ha (31)
Hd (31)
Hb
Ha
Hc
Br
29a
30e
31
Hd
0.24
0.03
1.00
0.02
6.8
6.6
6.4
6.2
6.0
[ppm]
[rel]
0.05
0.04
0.03
0.02
0.01
0.00

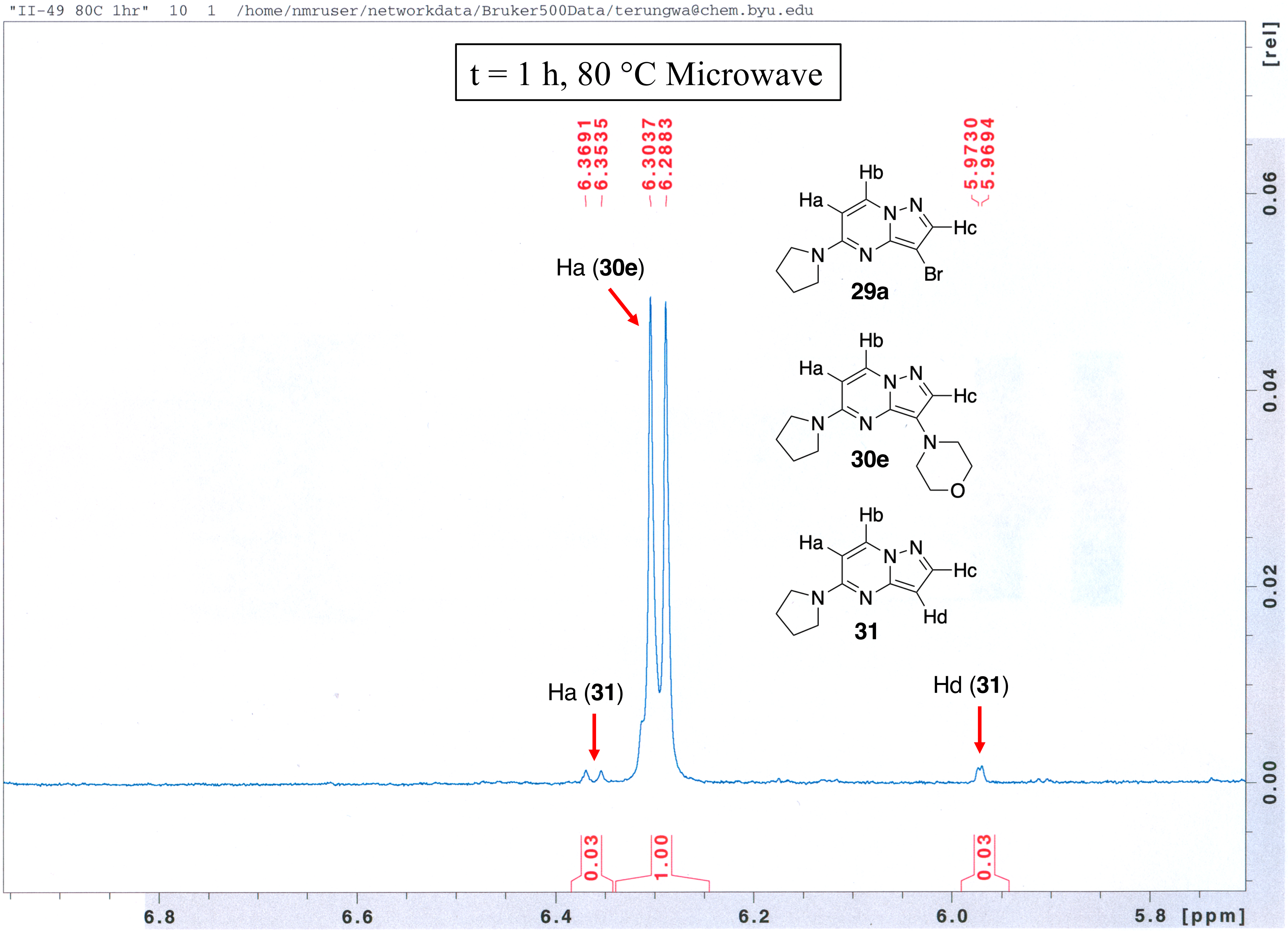

"II-49 80C 1hr" 10 1 /home/nmruser/networkdata/Bruker500Data/terungwa@chem.byu.edu
t = 1 h, 80 °C Microwave
6.3691
6.3535
6.3037
6.2883
5.9730
5.9694
Ha (30e)
Ha (31)
Hd (31)
29a
30e
31
Ha
Hb
Hc
Hd
Br
N
O
0.03
1.00
0.03
6.8
6.6
6.4
6.2
6.0
5.8
[ppm]
[rel]
0.06
0.04
0.02
0.00
